# A Vinyl Cyclopropane Ring Expansion and Iridium‐Catalyzed Hydrogen Borrowing Cascade

**DOI:** 10.1002/anie.202003614

**Published:** 2020-05-07

**Authors:** Simon Wübbolt, Choon Boon Cheong, James R. Frost, Kirsten E. Christensen, Timothy J. Donohoe

**Affiliations:** ^1^ Chemistry Research Laboratory University of Oxford Oxford OX1 3TA UK

**Keywords:** catalysis, cyclopentane, hydrogen borrowing, iridium, rearrangement

## Abstract

A vinyl cyclopropane rearrangement embedded in an iridium‐catalyzed hydrogen borrowing reaction enabled the formation of substituted stereo‐defined cyclopentanes from Ph* methyl ketone and cyclopropyl alcohols. Mechanistic studies provide evidence for the ring‐expansion reaction being the result of a cascade based on oxidation of the cyclopropyl alcohols, followed by aldol condensation with the pentamethyl phenyl‐substituted ketone to form an enone containing the vinyl cyclopropane. Subsequent single electron transfer (SET) to this system initiates a rearrangement, and the catalytic cycle is completed by reduction of the new enone. This process allows for the efficient formation of diversely substituted cyclopentanes as well as the construction of complex bicyclic carbon skeletons containing up to four contiguous stereocentres, all with high diastereoselectivity.

## Graphical abstract

**A vinyl cyclopropane/cyclopentene** rearrangement process has been embedded in an iridium‐catalyzed hydrogen borrowing reaction, enabling the formation of substituted, stereo‐defined cyclopentanes containing up to four contiguous stereogenic centres—all from reaction of pentamethylphenyl (Ph*) methylketone and a variety of cyclopropyl alcohols.

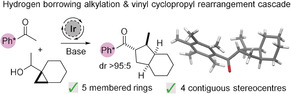

The transition metal‐catalyzed α‐alkylation of ketones using hydrogen borrowing chemistry has proven to be a powerful tool for the generation of carbon–carbon bonds.^1^ Our investigations have revealed the advantageous behavior of pentamethyl phenyl (Me_5_C_6_=Ph*) ketones, in which the aromatic ring is twisted out of conjugation with the carbonyl; this means that the *ortho*‐methyl groups shield the ketone from unwanted nucleophilic attack in situ.^2^ Exploiting this feature, we reported the iridium‐catalyzed α‐alkylation of ketones using primary and secondary alcohols to give α‐ and β‐branched compounds, respectively (Scheme 1 top).^2^, ^3^ In addition our group, and others, have shown that this strategy can be used to build ring systems of different sizes (especially six membered) by sequential hydrogen borrowing alkylation with diols (Scheme 1 top).^4^ It is important to note that after alkylation the Ph* group can be readily cleaved using a Br_2_‐mediated retro Friedel–Crafts acylation, with the resulting acid bromide converted into a variety of different carboxylic acid derivatives, aldehydes or alcohols.^2^, ^3^

**Scheme 1:**
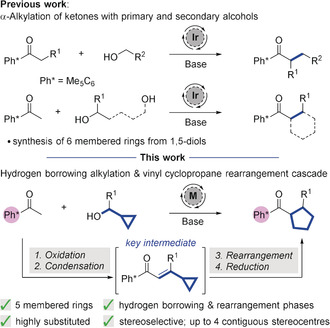
Previous work (top and middle) and proposed hydrogen borrowing sequence with embedded vinyl cyclopropane rearrangement (bottom).

Recently, we have considered the exciting possibility of promoting additional transformations of reactive intermediates generated during the hydrogen borrowing sequence (oxidation, condensation and reduction).^5^ In this context we proposed that a key cyclopropyl‐substituted enone intermediate, arising from a hydrogen borrowing process with a cyclopropyl alcohol, would undergo an in situ vinyl cyclopropane rearrangement to give 5‐membered ring systems (Scheme 1, bottom).^6^ This four‐stage, one‐pot method could generate stereochemically complex cyclopentanes that are not easily accessible with high yields and diastereoselectivity using our previous methodology utilizing 1,4‐diols under hydrogen borrowing catalysis.4b

Herein, we investigate the stereoselectivity of the rearrangement/hydrogen borrowing cascade, together with the scope for introducing substituents on the cyclopropyl alcohol component.^4^ To the best of our knowledge this attractive approach of integrating a rearrangement into a hydrogen borrowing cascade has not been previously described. With our recently developed systems in mind, we recognized that the reaction design would rely on a delay of the enone reduction to allow rearrangement and thus minimize the formation of directly alkylated product.^5^, ^7^

We began by investigating the reaction of Ph* methyl ketone (**1**) with 1‐cyclopropylpropane‐1‐ol (**2 a**). This was a suitable alcohol for the optimization process because it gave mixtures of the desired cyclopentane **4 a**, together with side‐products that were not rearranged (**3 a**, major) or ring‐opened (minor, not shown). First, we screened different catalyst and ligand combinations with KO*t*‐Bu as base at 85 °C in toluene, and found that [Ir(cod)Cl]_2_ in combination with the bulky ligand cataCXium A (L1) produced the desired cyclopentane **4 a** in 15 % yield with 7 % unrearranged side‐product **3 a** (Table 1, entries 1–4). An extended ligand screen (see Supporting Information, SI) revealed that 8 mol % of cataCXium A gave the highest yield of rearranged product, which is consistent with its ability to retard the hydrogen returning reduction step on the enone intermediate, providing time for the rearrangement to occur.^5^ After eventual reduction of the rearranged enone, vide infra, the cyclopentane was formed with high *trans*‐diastereoselectivity, presumably as a result of reversible deprotonation at the α‐position to the carbonyl of the product (Scheme 2 shows an X‐ray structure of **4 a**).4b The yield could be improved by increasing the reaction temperature (110 °C and then 125 °C) providing **4 a** in 29 % yield, while suppressing the formation of side‐product **3 a** (Table 1, entries 5,6). Pleasingly, a decrease in the amount of base from 3 equiv. to 1 equiv. resulted in a higher yield of 69 % (Table 1, entries 7,8). However, the use of substoichiometric amounts of base gave a poor yield (Table 1, entry 9). Seeking to improve the process, we also screened a variety of bases such as NaO*t*‐Bu, KOH, NaOH and KHMDS and found that KO*t*‐Bu gave the best results (Table 1, entries 10–13). These results show the extreme sensitivity of the reaction to the nature and amount of the base; presumably this is related to the efficiency of the aldol reaction which precedes rearrangement. Thus, we identified the conditions producing 69 % yield of the desired product **4 a** as being optimal (Table 1, entry 8).

**Scheme 2:**
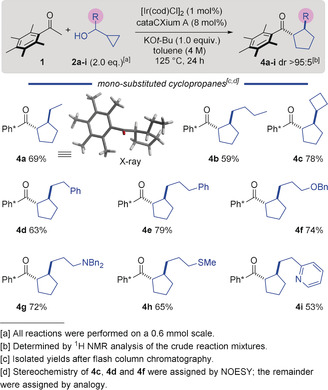
Scope of the vinyl cyclopropane/hydrogen borrowing cascade.

**Table 1:** Optimization of a vinyl cyclopropane/hydrogen borrowing cascade.^[a]^ 
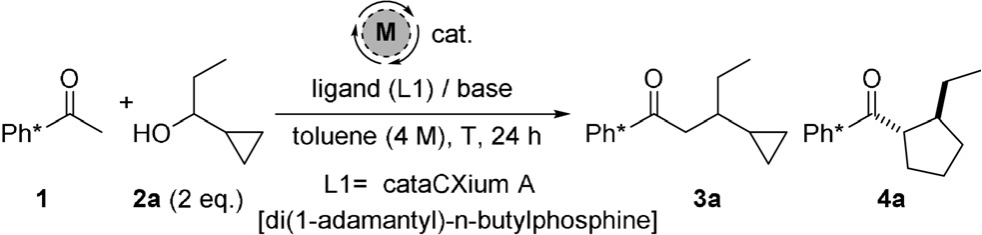

Entry	Catalyst (1 mol %)	L1 (mol %)	Base (equiv.)	*T* [°C]	Yield [%] **3 a** ^[b]^	Yield [%] **4 a** ^[b,c]^
1^[d]^	[RhCp*Cl_2_]_2_	–	KO*t*‐Bu (3)	85	–	trace
2^[d]^	[Cp*IrCl_2_]_2_	–	KO*t*‐Bu (3)	85	3	4
3^[e]^	[Rh(cod)Cl]_2_	8	KO*t*‐Bu (3)	85	–	8
4^[d]^	[Ir(cod)Cl]_2_	8	KO*t*‐Bu (3)	85	7	15
5^[e]^	[Ir(cod)Cl]_2_	8	KO*t*‐Bu (3)	110	trace	19
6^[e]^	[Ir(cod)Cl]_2_	8	KO*t*‐Bu (3)	125	trace	29
7^[e]^	[Ir(cod)Cl]_2_	8	KO*t*‐Bu (2)	125	1	40
**8**	**[Ir(cod)Cl]_2_**	**8**	**KO*t*‐Bu (1)**	**125**	**3**	**69^[f]^**
9^[d]^	[Ir(cod)Cl]_2_	8	KO*t*‐Bu (0.5)	125	trace	5
10^[d]^	[Ir(cod)Cl]_2_	8	NaO*t*‐Bu (1)	125	–	–
11^[d]^	[Ir(cod)Cl]_2_	8	NaOH (1)	125	trace	trace
12^[d]^	[Ir(cod)Cl]_2_	8	KOH (1)	125	1	7
13^[d]^	[Ir(cod)Cl]_2_	8	KHMDS (1)	125	trace	1

[a] All reactions were performed on a 0.6 mmol scale. [b] Determined by reverse phase HPLC analysis vs. durene as an internal standard. [c] dr >95:5. [d] The remaining mass balance mainly comprised of ketone **1**. [e] The remaining mass balance comprised of a complex mixture. [f] Isolated yield.

Using the optimized protocol, we investigated the substrate scope of the reaction and found that a wide range of functional groups were tolerated on the cyclopropyl alcohol carbinol center (**2 a**–**i**). Starting with the parent compound **4 a** which was isolated in 69 % yield (Scheme 2) an increase of the length of the sidechain gave **4 b** in 59 % yield. Next, we tested cyclopropyl alcohols substituted with cycloalkanes of different ring sizes and found that a 4‐membered ring was tolerated, giving **4 c** (78 %). Interestingly, cyclopropyl alcohols substituted with 5‐ and 6‐membered rings at the alcohol carbinol did not react (not shown), presumably because increased steric hindrance retarded the aldol reaction. Pleasingly, side chains of different sizes were tolerated and gave the products **4 d** and **4 e** in 63 % and 79 % yield, respectively. Furthermore, cyclopropyl alcohols decorated with protected heteroatoms were viable in the reaction. For example, cyclopropyl substrates bearing an OBn or NBn_2_ group furnished cyclopentanes **4 f** and **4 g** in >70 % yield. In addition, a SMe‐substituent was tolerated, giving **4 h** in 65 % yield. Finally, a cyclopropyl alcohol decorated with a pyridine resulted in the formation of **4 i** in 53 % yield. In all cases the 1,2‐*trans* diastereoisomer of the product was observed (dr >95:5). Note that the corresponding cyclopropyl alcohol **2** with R equal to a methyl group did rearrange but the product could not be purified from alkylated side‐product.^8^

Next, we investigated the role of substituents placed on the cyclopropane ring. Adding a distal phenyl‐substituent gave compounds **4 j**/**4 j′** in 54 % yield and 77:23 dr (Scheme 3). In this reaction, the more substituted carbon of the cyclopropane ring has migrated selectively. However, use of starting alcohol **2 k** with a phenyl disubstituted cyclopropane was unsuccessful; only traces of the product **4 k** were observed in the crude mixture.

**Scheme 3:**
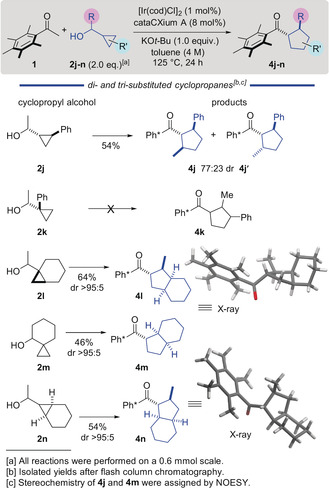
Scope of the vinyl cyclopropane/hydrogen borrowing cascade.

The behavior of other substituted bicyclic cyclopropyl alcohols was then investigated and these formed valuable bicyclic cyclopentanes of high complexity as single diastereoisomers. Cyclopropane **2 l** bearing a quaternary center gave octahydro‐indene derivative **4 l** in 64 % yield, while spiro compound **2 m** delivered **4 m** in 46 % yield (Scheme 3). Interestingly, neither of these two reactions gave any trace of the corresponding unrearranged alkylated side‐product. Finally, cyclopropyl‐annulated cyclohexane derivative **2 n** furnished the resulting bicycle **4 n** in 54 % yield. The structure and relative stereochemistry of bicyclic compounds **4 l** and **4 n** was proven unambiguously by X‐ray crystallography (Scheme 3).^9^

Using a series of Ph* substituted cyclopentanes we demonstrated the versatility of the Ph* group by cleavage with Br_2_. Subsequent in situ transformation of the resulting acid bromide intermediate allowed the synthesis of stereochemically complex cyclopentane carboxylic acid derivatives in high yields (Scheme 4).^2^ It is well established that Ph* cleavage reactions do not lead to erosion of stereochemistry adjacent to the carbonyl group.4a, 4b The lactone product **5 f** is noteworthy because the acid bromide formed in situ is capable of reacting intramolecularly with the ether, resulting in formation of a seven membered lactone after debenzylation.

**Scheme 4:**
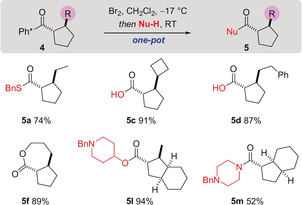
Release of the Ph* group and in situ coupling.

To investigate the mechanism for rearrangement, we synthesized the proposed intermediate (*E*/*Z*)‐**6** (55:45 dr) which would normally arise in a hydrogen borrowing sequence, and resubmitted it to the reaction conditions (Scheme 5). From this reaction, we isolated the rearranged product **4 a** in 35 % yield, along with the alkylated product **3 a** in 14 % yield (Scheme 5 A).

**Scheme 5:**
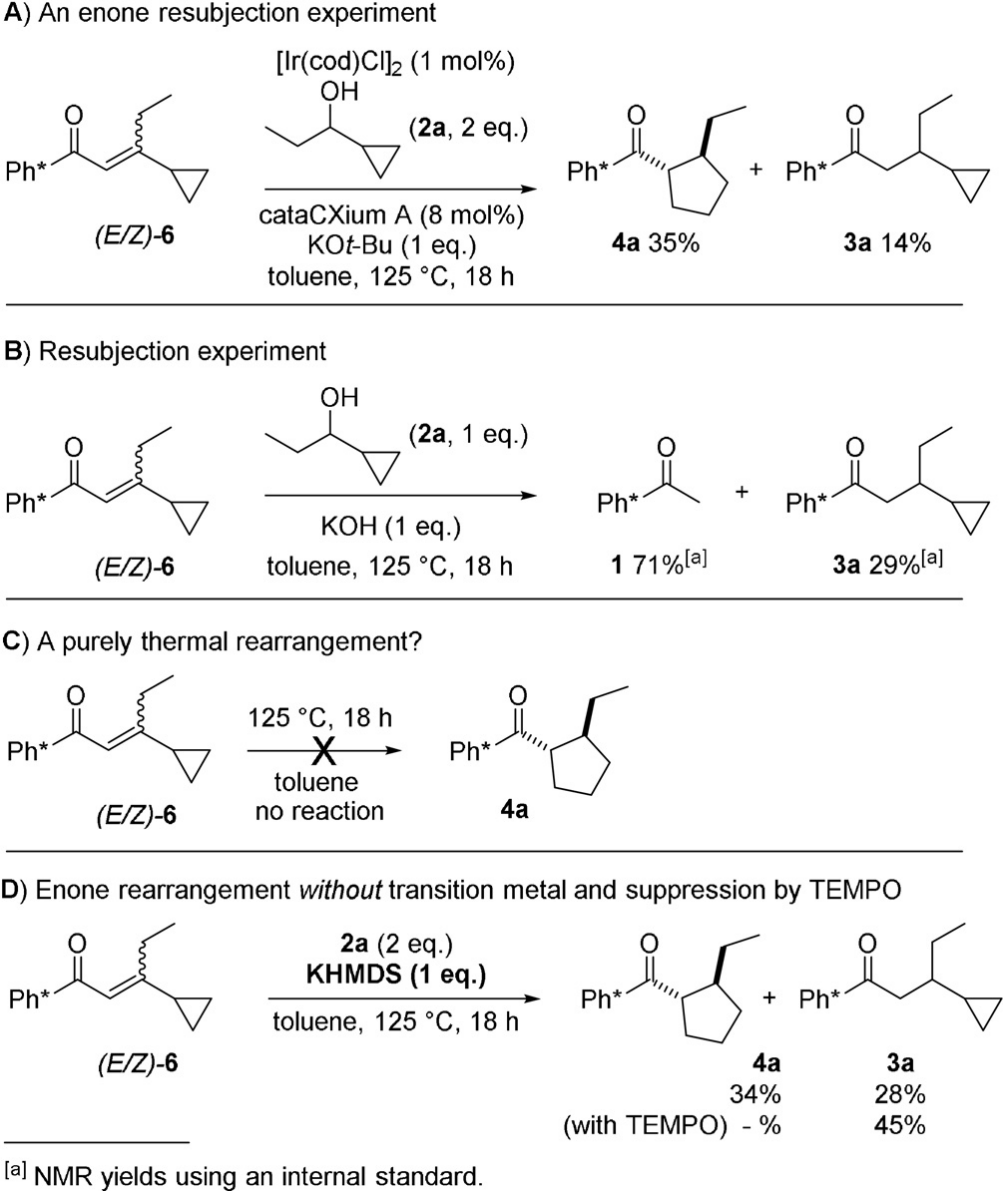
Mechanistic experiments.

We also attempted to gather evidence for nucleophilic opening of the cyclopropane ring by homo‐conjugate addition.^10^ Interestingly, when we added KOH to a mixture of (*E*/*Z*)‐**6** and **2 a**, without iridium catalyst, both starting ketone **1** and reduced product **3 a** were formed, and no ring opened or rearranged products were observed (Scheme 5 B).^11^, ^12^ The isolation of **1** illustrates how easily the aldol process that forms enone (*E*/*Z*)‐**6** can be reversed by hydroxide. In addition, the formation of reduced compound **3 a** implicates a transition metal free pathway for enone reduction, presumably related to a Meerwein–Ponndorf–Verley (MPV) type reaction; this is quite a common background reaction in hydrogen borrowing catalysis.^2^, ^13^

Further work at studying the rearrangement of (*E*/*Z*)‐**6** confirmed that the enone was sensitive to the presence of water and, under basic conditions, underwent a facile *retro* aldol reaction returning ketone **1**. Therefore, experiments examining the rearrangement of (*E*/*Z*)‐**6** were conducted under anhydrous conditions. Initially, we heated (*E*/*Z*)‐**6** in toluene at 125 °C overnight, and no reaction was observed, thus ruling out a purely thermal rearrangement process (Scheme 5 C).^6^, ^14^ Attempts to promote the rearrangement of (*E*/*Z*)‐**6** using metal catalysts alone (eg Rh, Ni) were also unsuccessful, even though there have been literature reports on metal‐catalyzed rearrangements of related cyclopropyl enones.^15^

Finally, when (*E*/*Z*)‐**6** and alcohol **2 a** were reacted with a solution of KHMDS in dry toluene (in order to make the potassium alkoxide under anhydrous conditions; without iridium catalyst) we were able to directly convert (*E*/*Z*)‐**6** into the rearranged and reduced (MPV) product **4 a** in 34 % yield, along with the reduced compound **3 a** in 28 % yield (Scheme 5 D; similar results were observed with NaHMDS).^16^ Removing the cyclopropyl alcohol **2 a** from the same reaction resulted only in deprotonation of (*E*/*Z*)‐**6** at the ethyl group by the KHMDS, followed by kinetic protonation of the extended enolate at the α‐position; no rearrangement was observed (see SI).^17^ Based on these results we reasoned that the rearrangement process does not involve the transition metal catalyst and that the presence of an alkoxide base is crucial.^18^ To investigate whether the rearrangement involves radicals, we added TEMPO (1 equiv.) to the rearrangement of (*E*/*Z*)‐**6**, which effectively suppressed the reaction, resulting in the formation of **3 a** in 45 % yield (Scheme 5 D). Furthermore, the addition of TEMPO to the parent reaction of ketone **1** and alcohol **2 a** in the presence of iridium dramatically lowered the yield of **4 a** to 6 % (see SI). These results suggest a (transition metal free) radical pathway for ring expansion.

Based on these experiments, we propose the mechanism shown (Scheme 6). In the first hydrogen borrowing phase, the alcohol **2** is oxidized by the iridium catalyst to the ketone **A**, and subsequently undergoes a condensation reaction with **1** to give enone **B** (Scheme 6). Then, a single electron transfer (SET), which may be initiated from an alkoxide or enolate to intermediate **B**,^19^ results in formation of radical‐anion **C**. The species **C** can then enter a ring opening/ring closing rearrangement phase^20^ to form a 5‐membered enone radical anion (**C**→**E**). Clearly, intermediate **D** must have the necessary *cis*‐alkene geometry in order to cyclise, and this may arise via a reversible reaction from **C**.20e Intermediate **E** could then conceivably propagate the SET chain by donating an electron to another molecule of **B** (Scheme 6). Under basic conditions, the alkene in **F** will isomerize into conjugation with the carbonyl group to give **G**, which can complete the hydrogen borrowing cycle and be reduced by the iridium hydride formed in step 1 to give the product **4**. This type of mechanism would be consistent with the formation of **4 j**/**4 j′** in which the more substituted end of the cyclopropane is subject to rearrangement, presumably via formation of a benzylic radical. The stereochemistry of bicyclic compounds **4 l**–**n** can also be explained via this mechanism, with the Ir−H adding to the convex face of a *cis*‐6,5 bicyclo enone intermediate analogous to **G**, and epimerization under basic conditions then fixing the stereochemistry of the carbonyl group into an *exo*‐position.

**Scheme 6:**
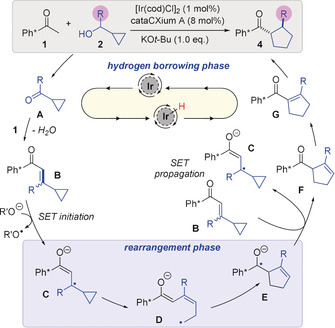
Proposed mechanism of the vinyl cyclopropyl rearrangement.

In conclusion, we have developed an efficient synthesis of cyclopentanes via a ring expansion reaction of cyclopropanes embedded into a hydrogen borrowing catalysis cycle. The methodology tolerates a wide range of functional groups and allows for the construction of complex bicyclic carbon skeletons containing up to four contiguous stereogenic centres, with high control over the relative stereochemistry. Control experiments suggest that the rearrangement is not mediated by the transition metal catalyst but is the result of a radical pathway. This strategy represents a powerful example of rearrangement becoming part of a hydrogen borrowing cascade and this concept offers many possibilities for development.

## Conflict of interest

The authors declare no conflict of interest.

## Supporting information

As a service to our authors and readers, this journal provides supporting information supplied by the authors. Such materials are peer reviewed and may be re‐organized for online delivery, but are not copy‐edited or typeset. Technical support issues arising from supporting information (other than missing files) should be addressed to the authors.

Supplementary
