# Supplementary material for: A Vinyl Cyclopropane Ring Expansion and Iridium‐Catalyzed Hydrogen Borrowing Cascade
Source: Angew Chem Int Ed Engl. 2020 May 7;59(28):11339–44. doi: 10.1002/anie.202003614 (PMC7463175; doi:10.1002/anie.202003614)
Supplement: Supplementary file 1 — Supplementary [file ANIE-59-11339-s001.pdf]

Angewandte Chemie
Eine Zeitschrift der Gesellschaft Deutscher Chemiker

Supporting Information

A Vinyl Cyclopropane Ring Expansion and Iridium-Catalyzed Hydrogen Borrowing Cascade

*Simon Wübbolt[+], Choon Boon Cheong[+], James R. Frost, Kirsten E. Christensen, and Timothy J. Donohoe**

anie_202003614_sm_miscellaneous_information.pdf

Supporting Information

Table of Contents

1. General Remarks……………………………...……………………2

2. General Procedures……………………………………………….….7

3. Experimental Details………………………………………………….8

4. NMR Spectra……………………………………………………..…43

5. Single crystal X-ray diffraction…………………………………..117

6. References………………………………………………..……120

1. General Remarks

Reaction Setup: Microwave vials and vial caps (containing a resealing Silicone/PTFE septum) were purchased from Kinesis (Cole-Palmer) and were used without flame-drying. Unless otherwise stated, all other reactions were performed in flame-dried glassware equipped with a stir bar using standard Schlenk techniques under an atmosphere of Ar dried over passage through Chameleon® silica gel, before replacing with a balloon of Ar. Room temperature (RT) is defined to be 25 °C (298 K).

Reagents and solvents: Unless detailed below or otherwise stated, all reagents and solvents were purchased and used as supplied from Sigma-Aldrich (now Merck KGaA), Thermo Fisher Scientific (including Alfa Aesar and Acros Organics), Fluorochem, Honeywell, Tokyo Chemical Industry, Apollo Scientific, Manchester Organics (part of Navin Fluorine Int. Ltd.) and Strem Chemicals.

Sublimed grade KO*t*-Bu (99.9%) was purchased from Sigma-Aldrich, stored under Ar and used as supplied.

$[Ir(COD)Cl]_2$ and $[Cp^*IrCl_2]_2$ were purchased from Sigma-Aldrich and/or Alfa Aesar and used as supplied.[*] All other transition metal catalysts and phosphine ligands were purchased from Sigma-Aldrich, Alfa Aesar, Fluorochem or Strem Chemicals, stored under Ar and used as supplied.

Anhydrous DMSO and Et_3N were purchased from Sigma-Aldrich in Sure/Seal™ bottles. Anhydrous Et_2O, DCM, MeOH, THF and toluene were obtained from MBRAUN SPS-5 solvent purification system by passage through double filtration columns under N_2.

1-Cyclopropylpentan-1-ol[1], 1-cyclopropyl-3-phenylpropan-1-ol[2], 1-(*cis*-bicyclo[4.1.0]heptan-7-yl)ethan-1-ol[3], 4-(dibenzylamino)butan-1-ol[4] and (3-bromopropyl)(methyl)sulfane[5] were synthesised according to literature procedures.

Chromatography: Thin layer chromatography (TLC) was performed on pre-coated aluminium- (200 μm) or glass-backed (250 μm) Merck Kieselgel 60 F_{254} plates, or polyester-backed Macherey-Nagel Polygram® SIL G/UV_{254} plates, visualised using UV irradiation (λ = 254 nm) and/or staining with potassium permanganate ($KMnO_4$), vanillin, 2,4-dinitrophenylhydrazine (2,4-DNPH) or cerium ammonium molybdate (CAM, or Hanessian's

[*] Negligible variation was observed between either suppliers across multiple batches.

stain) solutions. Preparative TLC was performed using the abovementioned TLC plates for samples within 5–25 mg of crude mass, or Merck Kieselgel 60 F_{254} preparative TLC plates (500 µm) for samples within 25–100 mg of crude mass.

Purification by flash column chromatography was performed with Merck Kieselgel 60 (40–63 µm) or (15–40 µm) silica gel according to the method by W. C. Still et al.[6] All solvents used for chromatography were HPLC grade or equivalent and supplied by Honeywell, Sigma-Aldrich or Thermo Fisher Scientific.

Nomenclature: Systematic names were generated by the software ChemDraw according to the guidelines specified by the International Union of Pure and Applied Chemistry (IUPAC). Conventional names are also provided for reference where applicable. All compounds reported are racemates, and stereodescriptors drawn in the structures are relative. For *meso* and other symmetric achiral cyclic compounds, the prefixes *cis* and *trans* are used for clarity where appropriate.

Compound Numbering: Unless otherwise stated, compounds are numbered such that only unique carbons are labelled based on their systematic names analogous to IUPAC nomenclature guidelines.

Melting points: Melting points (m.p.) were obtained using a Leica VMTG heated-stage microscope equipped with a Testo 720 thermometer and are uncorrected.

Infrared spectroscopy: Fourier-transform infrared (FT-IR) spectra were recorded from evaporated films on a Bruker Tensor 27 spectrometer equipped with a Pike Miracle Attenuated Total Reflectance (ATR) sampling accessory. Absorption maxima are quoted in wavenumbers (ν_{max}) with units of cm^{-1} and for the range of 3600–600 cm^{-1}.

NMR spectroscopy: All spectra were acquired on a Bruker DPX 200 equipped with a 5 mm $^{13}C/^{1}H$ dual probe, a Bruker AVIII HD 400 equipped with a 5 mm *z*-gradient BBFO probe, Bruker AVIII HD 400 equipped with a 5 mm *z*-gradient BBFO "SMART" probe, Bruker AVII 500 equipped with a 5 mm BBFO "SMART" probe or 5 mm *z*-gradient TFI probe, or Bruker AVIII HD equipped with a 5 mm triple resonance TBO probe. All NMR acquisition was performed using the software Bruker TopSpin®. The deuterated solvent acted as internal deuterium lock. ^{1}H NMR experiments were recorded at 400 or 500 MHz, ^{13}C NMR at 101 or 126 MHz with broadband proton decoupling. Unless otherwise stated, all NMR experiments were performed at 298 K.

NMR data processing and analyses were performed with the software MestReNova. Chemical shifts (δ) are quoted in parts per million (ppm) to the nearest 0.01 ppm for ^{1}H and 0.1 ppm for ^{13}C and ^{15}N NMR; for cases where signals in ^{13}C NMR spectra are within 0.1 ppm, only the signals involved are reported to 0.01 ppm. Chemical shifts for ^{15}N nuclei, where available, were obtained by direct detection from ^{15}N NMR or by indirect detection from ^{1}H-^{15}N HMBC experiments; the chemical shifts thus recorded have a deviation of within 0.1 ppm from either experiments.

Residual protic solvent signal acted as an internal reference for ^{1}H NMR, and deuterated solvent carbon signal acted as an internal reference for ^{13}C NMR ($CDCl_3$: ^{1}H NMR = 7.26 ppm; ^{13}C NMR = 77.16 ppm. In accordance to IUPAC guidelines, the chemical shifts for ^{15}N NMR spectra reported are based on the secondary frequencies (Ξ) of the respective nuclei in the unified scale relative to ^{1}H in tetramethylsilane.[7*]

Every attempt was made to obtain *J*-coupling information through multiplet analysis with the aid of line fitting. The multiplicity of a signal is reported as such: s – singlet, d – doublet, t – triplet, q – quartet, quint. – quintet, sext. – sextet, sept. – septet, oct. – octet, non. – nonet, q_{AB} – AB quartet, m – multiplet, m_c centrosymmetric multiplet, app. – apparent or approximate, br. – broad, v. – very, or combinations thereof. Coupling constants and Δ_{AB} are quoted in Hz to the nearest 0.1 Hz. Structural assignments were made with the aid of DEPTQ, COSY, HSQC, HMBC, H2BC, NOESY, ROESY and TOCSY experiments.

[*] For ^{15}N NMR, the compound of reference is liq. NH_3. The chemical shift reference can be converted to a nitromethane reference by subtracting 380.5 ppm.

Mass spectrometry (MS): Low resolution MS were recorded on an Agilent 6120 Quadrupole mass spectrometer equipped with an Agilent 1260 Infinity LC pump, or a Waters LCT Premier XE mass spectrometer equipped with a Waters 1525μ Binary HPLC pump, both with a flow rate of 0.2 mL min^{-1} using H_2O:MeOH:HCOOH (10:89.9:0.1) as eluent. The mass reported is the mass-to-charge ratio (*m*/*z*) containing the most abundant isotopes under the conditions of electrospray ionisation (ESI), with each value to 1 decimal place.

High resolution mass spectrometry (HRMS) under ESI conditions were recorded on a Thermo Exactive Orbitrap mass spectrometer equipped with a Waters Equity LC system, a Bruker MicroToF mass spectrometer equipped with an Agilent 1100 HPLC pump and autosampler, or on a Waters Xevo Quadrupole Time of Flight (Q-ToF) mass spectrometer. The Thermo Exactive system employs a flow rate of 0.2 mL min^{-1} using H_2O:MeOH:HCOOH (10:89.9:0.1) as eluent, with a heated electrospray ionisation (HESI-II) probe and has a resolution of 50,000 FWHM. The Bruker system uses the built-in electrospray source, while the Waters system runs on a lock-mass mode with ESI performed by a secondary electrospray source, both using conditions identical to the Thermo Exactive system. Instrument control and data processing were performed using the softwares Thermo Xcalibur for the Thermo Exactive system, Compass DataAnalysis 4.0 for the Bruker system, and MassLynx for the Waters system.

Atmospheric pressure chemical ionisation (APCI) HRMS were recorded on the abovementioned Thermo Exactive spectrometer under identical conditions using N_2 as the reagent gas.

Chemical ionisation (CI) and electron impact ionisation (EI) HRMS were performed on an Agilent 7200 Quadrupole Q-ToF mass spectrometer equipped with a direct insertion probe supplied by Scientific Instrument Manufacturer (SIM) GmbH. Instrument control and data processing were performed using the software Agilent MassHunter. The reagent gas used for CI was NH_3 or CH_4.

All HRMS conditions were adjusted for maximum sensitivity, with an accuracy of better than 5 ppm for 24 h following external calibration on the day of analysis. Unless otherwise specified, the mass reported for HRMS is the mass-to-charge ratio containing the most abundant isotopes, with each value to 4 or 5 decimal places and within 5 ppm of the calculated mass.

Reverse phase HPLC: Reverse phase HPLC separations were achieved using an Dionex Ultimate 3000 HPLC unit and Chromeleon software. Agilent Poroshell 120 EC-C18 4μm column (150 × 4.6 mm), fitted with matching Agilent Guard Cartridges (10 × 4 mm), were used

as specified in the text. Solvents used were of HPLC grade (Fisher Scientific, Sigma Aldrich or Rathburn); all eluent systems were isocratic.

X-Ray Crystallography: Single crystal X-ray diffraction was performed by Dr. Kirsten E. Christensen on a (Rigaku) Oxford Diffraction/Agilent Supernovae A diffractometer using Cu-K_α radiation (λ = 1.54184 Å) and a graphite monochromator. Samples were mounted on perfluoropoly-ethyl ether oil and cooled by a Cryostream N_2 open-flow cooling device[8] to 150 K throughout the data collection process. For very small crystals, diffraction data was collected on beamline I-19 at the Diamond Light Source (λ = 0.68890 Å) at a temperature of 100 K. The diffraction patterns were integrated and reduced using the software CrysAlisPro. The software CRYSTALS for Microsoft Windows was used to obtain *ab initio* solutions (using SuperFlip[9] embedded within CRYSTALS[10]) and carry out structure refinement.[11] The images of the solved structures were generated with the software Mercury, and represent displacement ellipsoid plots of the best-fit model drawn at 50% probability level.

2. General Procedures

General Procedure A: Hydrogen Borrowing of Cyclopropyl Secondary Alcohols

To a 2–5 mL microwave vial equipped with a stirrer bar was added alcohol (2.0 equiv), ketone **1** (1.0 equiv), $[Ir(COD)Cl]_2$ (1 mol%), cataCXium® A (8 mol%), KO*t*-Bu (99.9%, 1.0 equiv) and toluene (4.0 M) sequentially in the open atmosphere. The vial was sealed with a microwave vial cap, then evacuated and backfilled with Argon. The vial was then heated to 125 °C in a preheated oil bath for 24 h. The reaction mixture was cooled to RT, diluted with Et_2O and washed with sat. aq. NH_4Cl. The aqueous layer was extracted with Et_2O (3x) and the combined organic layers were concentrated *in vacuo*. Purification by column chromatography provided the desired compound. Purification of the rearranged cyclopentane products generally required the use of Merck Silica gel 60 (15–40 µm).

General Procedure B: Br_2-mediated Cleavage of Pentamethylbenzene

To a 2–5 mL microwave vial equipped with a stirrer bar was added substrate (1.0 equiv) and DCM (0.2 M) sequentially in the open atmosphere. The vial was sealed with a microwave vial cap, cooled to –17 °C (ice/NaCl bath), then Br_2 (2.0 equiv) was added and stirred at –17 °C. Once TLC analysis indicated complete consumption of the substrate (typically 15 min), the appropriate nucleophile (3.0 equiv) was added at –17 °C and the reaction allowed to warm up to RT over 16 h. To the reaction mixture was added Et_2O (15 mL per mmol substrate), sat. aq. Na_2SO_3 (10 mL per mmol substrate) and brine (10 mL per mmol) sequentially. The layers were separated, the aqueous layer extracted with Et_2O (3 × 15 mL per mmol), and the combined organics dried (Na_2SO_4) and concentrated *in vacuo*. Purification by column chromatography provided the desired compound.

3. Experimental Details

Table S1. Ligand Screen

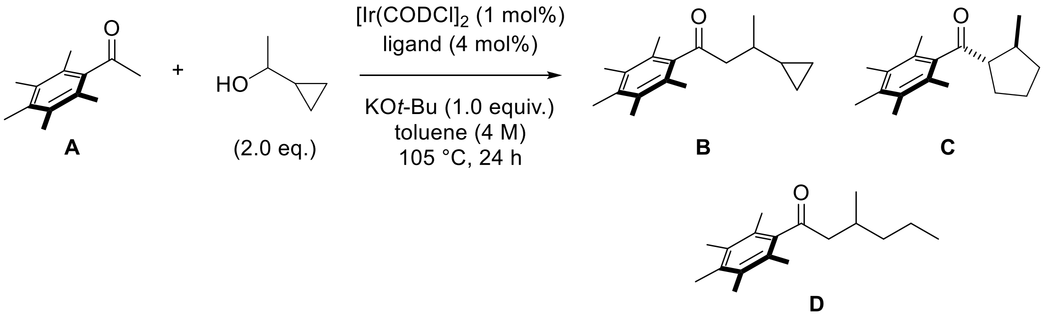

Entry[a]	Ligand	Ligand loading (mol%)	NMR ratio[b] **A**	**B**	**C**	**D**
1	cataCXium ABn	4	0.12	1	0.82	0.26
2	PAd_3	4	0.09	0.79	1	Traces
3	cataCXium FBn•HI	4	0.22	0.9	1	Traces
4	cataCXium FBu•HI	4	0.13	0.83	1	Traces
5	PCy_3	4	0.13	0.85	1	0.23
6	P*t*-Bu_3•HBF_4	4	0.27	0.93	1	–
7	P*n*-Bu_3•HBF_4	4	0.2	1	0.55	0.41
8	CyJohnPhos	4	0.18	1	1	0.24
9	TrixiePhos	4	0.12	1	0.97	Traces
10	PPh_3	4	Trace	1	0.76	0.29
11	P(*p*-Anisyl)$_3$	4	0.7	1	0.48	0.19
12	$P(Ar^F)_3$	4	0.65	1	0.33	0.21
13	$PMes_3$	4	0.15	0.87	1	Traces
14	$P(C_6F_5)_3$	4	1.21	1	0.67	0.15
15	$P(OPh)_3$	4	0.43	0.87	1	Traces
16	(*S*)-SDP	4	0.08	0.85	1	0.11
17	DPPBz	4	–	1	0.34	0.22
18	DPEPhos	4	–	1	0.12	0.22
19	(±)-BINAP	4	0.06	1	0.75	0.16
20	DPPF	4	–	1	0.34	0.19
21	DTBF	4	0.29	0.89	1	–
22	dppe	4	–	1	0.11	0.15
23	dppp	4	–	1	0.19	0.23
24	dppb	4	–	1	0.32	0.17
25	dpph	4	Trace	1	0.29	0.21
26	(2*S*,4*S*)-1,3-Me_2dppp	4	–	1	0.23	0.22
27	Triphos A	4	Trace	1	1	Traces
28	Triphos B	4	0.05	1	0.81	0.23
29	Tetraphos	4	0.31	1	–	–
30	cataCXium A (**L1**)	4	–	0.7 ≈36%[c]	1 ≈50%[c]	Traces
31	cataCXium A (**L1**)	8	–	0.46 ≈28%[c]	1 ≈60%[c]	Traces

[a] All reactions were performed on a 0.6 mmol scale. [b] Ratios were obtained by quantitative ^{1}H NMR analysis of the crude mixture [c] Calculated yield after column chromatography of obtained mixtures by quantitative ^{1}H NMR analysis.

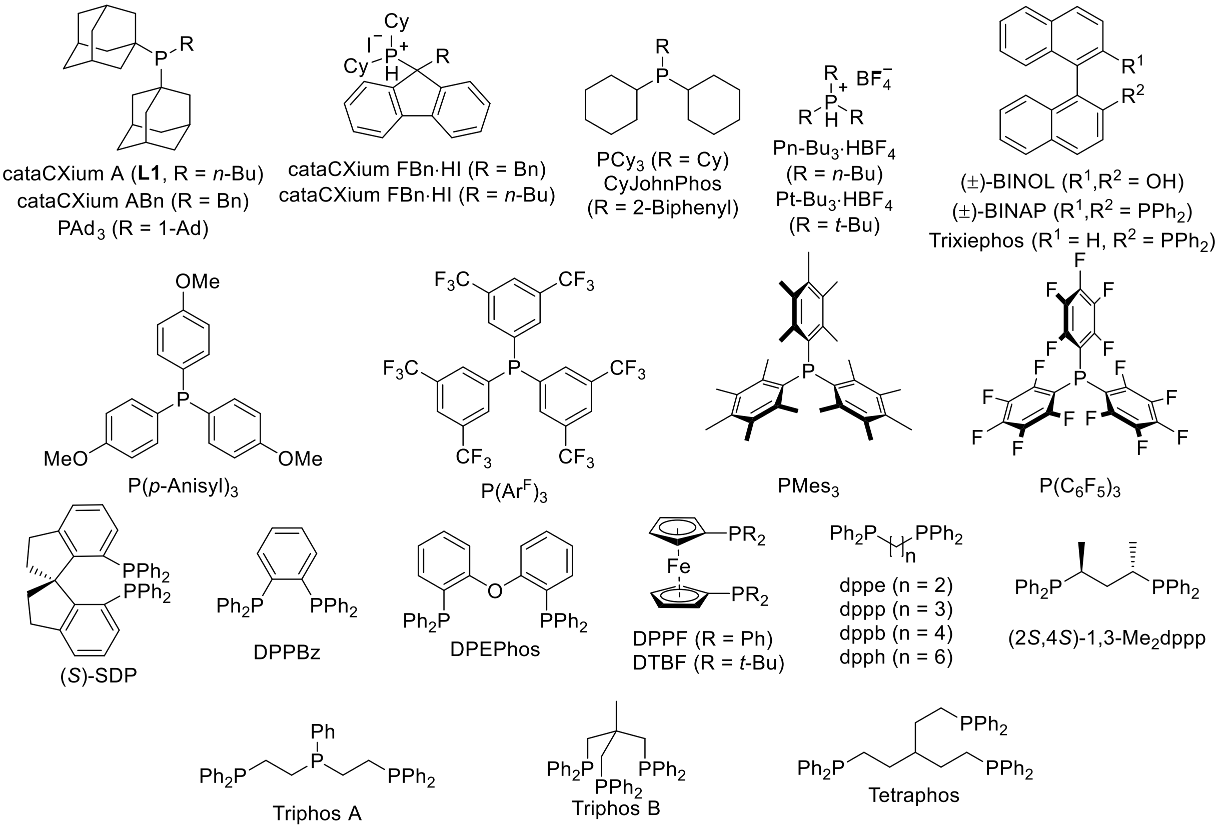

Scheme S1. Ligands used in the Table S1.

Initial screening experiments, including the ligand screen was performed using 1-cyclopropylethan-1-ol: however, separation of the alkylated and rearranged product could not be achieved, therefore we optimized further using 1-cyclopropylpropan-1-ol.

1-Cyclopropylpropan-1-ol (2a)

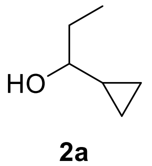

2a
$C_6H_{12}O$
100.16 g mol^{-1}

To a solution of EtMgBr (3 M in Et_2O, 6.20 mL, 18.6 mmol, 1.30 equiv) at RT was added a solution of the cyclopropylcarboxaldehyde (1.00 g, 14.3 mmol, 1.00 equiv) in THF (5 mL + 2 × 2 mL rinse) dropwise over 10 min (a gentle reflux was observed). The reaction was stirred for 1 h, and then quenched by the addition of sat. aq. NH_4Cl (15 mL). The layers were separated and the aqueous layer extracted with Et_2O (2 × 20 mL). The combined organics were dried ($MgSO_4$) and carefully concentrated using a stream of N_2. Purification by column

chromatography (SiO_2, eluent load, Pentane:Et_2O, 80:20) afforded alcohol **2a** (927 mg, 9.26 mmol, 65%) as a colourless oil.

IR (film) ν_{max}/cm^{-1} 3333, 3081, 3005, 2975, 2961, 2936, 2924, 2878, 2852, 1464, 1431, 1412, 1378, 1343, 1306, 1279, 1248, 1173, 1151, 1112, 1096, 1073, 1043, 1019, 967.
^{1}H NMR ($CDCl_3$, 400 MHz) δ = 2.81–2.74 (1H, m), 1.81–1.65 (1H, br. s), 1.69–1.53 (2H, m), 0.98 (3H, t, J = 7.5 Hz), 0.93–0.83 (1H, m), 0.57–0.42 (2H, m), 0.29–0.17 (2H, m) ppm.
^{13}C NMR ($CDCl_3$, 101 MHz) δ = 78.4, 30.2, 17.7, 10.2, 2.9, 2.4 ppm.
HRMS (ESI$^+$) Found $[M+H]^+$ = 101.0964; $C_6H_{13}O$ requires 101.0966.
The spectroscopic data is in good agreement with previous reports.[12]

Cyclobutyl(cyclopropyl)methanol (2c)

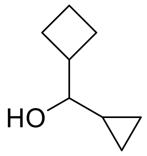

2c
$C_8H_{14}O$
126.20 g mol^{-1}

To a solution of cyclopropylMgBr (0.5 M in THF, 37.2 mL, 18.6 mmol, 1.30 equiv) at RT was added a solution of the cyclobutylcarboxaldehyde (1.20 g, 14.3 mmol, 1.00 equiv) in THF (5 mL + 2 × 2 mL rinse) dropwise over 15 min (a gentle reflux was observed). The reaction was stirred for 1 h, and then quenched by the addition of sat. aq. NH_4Cl (15 mL). The layers were separated and the aqueous layer extracted with Et_2O (2 × 50 mL). The combined organics were dried ($MgSO_4$) and carefully concentrated using a stream of N_2. Purification by column chromatography (SiO_2, eluent load, Pentane:Et_2O, 80:20→70:30) afforded alcohol **2c** (1.41 g, 11.2 mmol, 78%) as a colourless oil.

IR (film) ν_{max}/cm^{-1} 3371, 3080, 3004, 2973, 2939, 2863, 1464, 1443, 1430, 1414, 1342, 1272, 1243, 1204, 1173, 1121, 1080, 1042, 1018, 1002, 947.
^{1}H NMR ($CDCl_3$, 400 MHz) δ = 2.82 (1H, app. t, J = 7.8 Hz), 2.56–2.45 (1H, m), 2.08–1.72 (6H, m), 1.46 (1H, br. s, OH), 0.85–0.75 (1H, m), 0.54–0.39 (2H, m), 0.30–0.19 (2H, m) ppm.
^{13}C NMR ($CDCl_3$, 101 MHz) δ = 80.1, 41.6, 24.7, 24.6, 18.4, 15.7, 2.7, 1.6 ppm.
A stable molecular ion could not be detected by ESI, APCI or EI. The molecule loses H_2O too quickly

1-Cyclopropyl-4-phenylbutan-1-ol (2e)

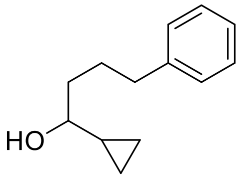

2e
$C_{13}H_{18}O$
190.29 g mol^{-1}

A suspension of magnesium turnings (316 mg, 13.0 mmol, 1.30 equiv) in Et_2O (15 mL) was stirred at RT and 1,2-dibromoethane (2–5 drops) was added followed by addition of (3-bromopropyl)benzene (1.52 mL, 10.0 mmol, 1.00 equiv) at such a rate as to maintain gentle reflux. After the addition was complete the resulting suspension was heated to reflux for a further 2 h and then cooled to 0 °C. Subsequently, to the formed Grignard solution was added dropwise cyclopropanecarbaldehyde (673 mmL, 9.00 mmol, 0.900 equiv). The resulting reaction mixture was stirred at RT for 18 h and quenched by the addition of sat. aq. NH_4Cl-solution (20 mL). The layers were separated, and the aqueous layer was extracted with Et_2O (2 × 20 mL). The combined organic extracts were dried over Na_2SO_4, filtered and solvents removed under reduced pressure. Purification by column chromatography of the residue (SiO_2, eluent load, pentane:Et_2O, 60:40) afforded alcohol **2e** (1.44 g, 7.57 mmol, 75%) as a colourless oil.

IR (film) ν_{max}/cm^{-1} 3374, 3081, 3026, 3003, 2935, 2856, 2361, 1603, 1496, 1453, 1085, 1018, 967, 915, 824, 747, 699.

^{1}H NMR ($CDCl_3$, 400 MHz) δ = 7.31–7.25 (2H, m), 7.22–7.15 (3H, m), 2.88 (1H, dt, J = 8.5, 6.1 Hz), 2.65 (2H, td, J = 8.1, 7.5, 2.3 Hz), 1.88–1.70 (2H, m), 1.69–1.61 (2H, m), 1.49 (1H, s), 0.89 (1H, qt, J = 8.3, 5.0 Hz), 0.60–0.43 (2H, m), 0.33–0.15 (2H, m) ppm.

^{13}C NMR ($CDCl_3$, 101 MHz) δ = 142.6, 128.6 (2C), 128.4 (2C), 125.8, 76.9, 37.0, 36.2, 27.7, 18.2, 3.0, 2.6 ppm.

HRMS (ESI$^+$) Found $[M+H]^+$ = 191.1430; $C_{13}H_{19}O$ requires 191.1436.

4-(Benzyloxy)-1-cyclopropylbutan-1-ol (2f)

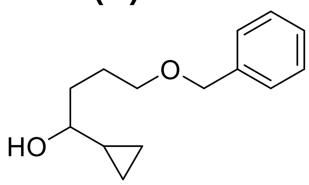

2f

$C_{14}H_{20}O_2$

220.31 g mol^{-1}

To a solution of oxalyl chloride (1.93 mL, 22.5 mmol, 1.50 equiv) in DCM (34.0 mL) at –78 °C was added DMSO (3.20 mL, 45.0 mmol, 3.00 equiv). The mixture was stirred for 10 min at –78 °C, after which a solution of 4-(benzyloxy)butan-1-ol (2.64 mL, 15.0 mmol, 1.00 equiv) in DCM (13.6 mL + 2 × 4 mL rinse) was added dropwise over 15 min. Following this, the reaction was stirred for 20 min at –78 °C and then Et_3N (10.5 mL, 75.0 mmol, 5.00 equiv) added dropwise. The suspension was allowed to warm to RT over 50 min and sat. aq. NH_4Cl (50 mL) added. The layers were separated, and the organic layer washed with sat. aq. NH_4Cl (3 × 50 mL), brine (50 mL), dried ($MgSO_4$) and concentrated *in vacuo*. The crude aldehyde was carried through to the next step in the synthesis without further purification.

To a solution of cyclopropylMgBr (0.5 M in THF, 39.0 mL, 19.5 mmol, 1.30 equiv) at RT was added a solution of the crude aldehyde (assumed quant., 15.0 mmol, 1.00 equiv) in THF (6.50 mL + 2 × 2 mL rinse) dropwise over 15 min (a gentle reflux was observed). The reaction was stirred for 1 h, and then carefully poured onto cold 1 *N* HCl (30 mL). The layers were separated and the aqueous layer extracted with Et_2O (2 × 40 mL). The combined organics were washed with H_2O (60 mL), brine (60 mL), dried ($MgSO_4$) and concentrated *in vacuo*. Purification by column chromatography (SiO_2, eluent load, Pentane:Et_2O, 70:30→65:35→60:40) afforded alcohol **2f** (2.26 g, 10.3 mmol, 68% over 2 steps) as a pale yellow oil.

IR (film) ν_{max}/cm^{-1} 3410, 3079, 3066, 3029, 2998, 2981, 2972, 2950, 2930, 2915, 2858, 1496, 1454, 1431, 1410, 1393, 1362, 1308, 1265, 1243, 1205, 1152, 1091, 1077, 1047, 1020, 978.

^{1}H NMR ($CDCl_3$, 400 MHz) δ = 7.37–7.31 (4H, m), 7.30–7.26 (1H, m), 4.52 (2H, s), 3.51 (2H, t, *J* = 6.0 Hz), 2.86 (1H, td, *J* = 8.2, 3.8 Hz), 2.17–2.06 (1H, br. s), 1.82–1.71 (3H, m), 1.71–1.60 (1H, m), 0.94–0.84 (1H, m), 0.55–0.45 (2H, m), 0.33–0.24 (1H, m), 0.23–0.14 (1H, m) ppm.

^{13}C NMR ($CDCl_3$, 101 MHz) δ = 138.4, 128.5 (2C), 127.8 (2C), 127.7, 76.7, 73.1, 70.7, 34.5, 26.4, 18.0, 2.8, 2.8 ppm.

HRMS (ESI$^+$) Found [M+Na]$^+$ = 243.1356; $C_{14}H_{20}O_2Na$ requires 243.1356.

1-Cyclopropyl-4-(dibenzylamino)butan-1-ol (2g)

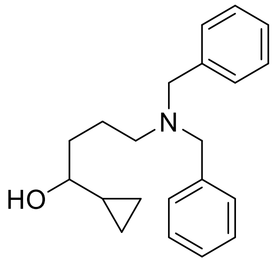

2g
$C_{21}H_{27}NO$
309.45 g mol^{-1}

To a solution of oxalyl chloride (1.84 mL, 21.5 mmol, 1.50 equiv) in DCM (32.5 mL) at –78 °C was added DMSO (3.05 mL, 42.9 mmol, 3.00 equiv). The mixture was stirred for 10 min at –78 °C, after which a solution of 4-(Dibenzylamino)butan-1-ol (3.85 g, 14.3 mmol, 1.00 equiv) in DCM (13 mL + 2 × 4 mL rinse) was added dropwise over 15 min. Following this, the reaction was stirred for 20 min at –78 °C and then Et_3N (10 mL, 71.5 mmol, 5.00 equiv) added dropwise. The suspension was allowed to warm to RT over 50 min and sat. aq. NH_4Cl (50 mL) added. The layers were separated, and the organic layer washed with sat. aq. NH_4Cl (3 × 50 mL), brine (50 mL), dried ($MgSO_4$) and concentrated *in vacuo*. The crude aldehyde was carried through to the next step in the synthesis without further purification.

To a solution of cyclopropylMgBr (0.5 M in THF, 37.2 mL, 18.6 mmol, 1.30 equiv) at RT was added a solution of the crude aldehyde (assumed quant., 14.3 mmol, 1.30 equiv) in THF (5 mL + 2 × 2 mL rinse) dropwise over 15 min (a gentle reflux was observed). The reaction was stirred for 1 h, and then quenched by the addition of sat. aq. NH_4Cl (15 mL). The layers were separated and the aqueous layer extracted with Et_2O (2 × 50 mL). The combined organics were dried ($MgSO_4$) and concentrated *in vacuo*. Purification by column chromatography (SiO_2, eluent load, Pentane:Et_2O, 80:20→70:30) afforded alcohol **2g** (3.12 g, 10.1 mmol, 70% over 2 steps) as a pale yellow oil.

IR (film) v_{max}/cm^{-1} 3383, 3062, 3027, 3002, 2937, 2794, 1601, 1494, 1453, 1366, 1244, 1124, 1060, 1027, 971.

^{1}H NMR ($CDCl_3$, 400 MHz) δ = 7.38–7.28 (8H, m), 7.27–7.21 (2H, m), 3.62 (3H, m), 3.51 (2H, d, J = 13.5 Hz), 2.71 (1H, td, J = 8.2, 3.4 Hz), 2.52–2.39 (2H, m), 1.75–1.63 (3H, m), 1.57–1.43 (1H, m), 0.90–0.79 (1H, m), 0.53–0.40 (2H, m), 0.31–0.23 (1H, m), 0.14–0.06 (1H, m) ppm.

^{13}C NMR ($CDCl_3$, 101 MHz) δ = 138.9 (2C), 129.3 (4C), 128.4 (4C), 127.1 (2C), 76.6, 58.4 (2C), 53.9, 35.7, 23.7, 18.0, 3.0, 2.6 ppm.

HRMS (ESI$^+$) Found [M+H]$^+$ = 310.2169; $C_{21}H_{28}ON$ requires 310.2165.

1-Cyclopropyl-4-(methylthio)butan-1-ol (2h)

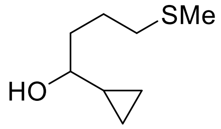

2h
$C_8H_{16}OS$
160.28 g mol^{-1}

A suspension of magnesium turnings (280 mg, 11.5 mmol, 1.30 equiv) in Et_2O (15 mL) was stirred at RT and 1,2-dibromoethane (2–5 drops) was added followed by addition of (3-bromopropyl)(methyl)sulfane (1.50 g, 8.87 mmol, 1.00 equiv) at such a rate as to maintain gentle reflux. After the addition was complete the resulting suspension was heated to reflux for a further 2 h and then cooled to 0 °C. Subsequently, to the formed Grignard solution was added dropwise cyclopropanecarbaldehyde (596 mmL, 7.98 mmol, 0.900 equiv). The resulting reaction mixture was stirred at RT for 72 h and quenched by the addition of sat. aq. NH_4Cl-solution (20 mL). The layers were separated and the aqueous layer was extracted with Et_2O (2 × 20 mL). The combined organic extracts were dried over Na_2SO_4, filtered and solvents removed under reduced pressure. Purification by column chromatography of the residue (SiO_2, eluent load, pentane:Et_2O, 85:15) afforded alcohol **2h** (833 mg, 5.20 mmol, 59%) as a colourless oil. b

IR (film) ν_{max}/cm^{-1} 3385, 3078, 3002, 2915, 2859, 1428, 1290, 1068, 1043, 1018, 952, 916, 855, 824, 774, 696.
^{1}H NMR ($CDCl_3$, 400 MHz) δ = 2.92–2.84 (1H, m), 2.56–2.49 (2H, m), 2.10 (3H, s), 1.83–1.63 (4H, m), 1.54 (1H, br. s), 0.91 (1H, qt, J = 8.3, 4.9 Hz), 0.59–0.45 (2H, m), 0.31–0.18 (2H, m) ppm.
^{13}C NMR ($CDCl_3$, 101 MHz) δ = 76.7, 35.8, 32.4, 24.8, 18.6, 14.7, 2.9, 1.9 ppm.
HRMS (ESI$^-$) Found [M–H]$^-$ = 159.0839; $C_8H_{15}OS$ requires 159.0844.

1-Cyclopropyl-3-(pyridin-2-yl)propan-1-ol (2i)

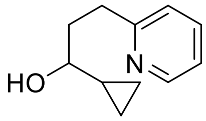

2i
$C_{11}H_{15}NO$
177.25 g mol^{-1}

To a solution of 1-cyclopropylethan-1-one (3.47 mL, 35.0 mmol, 1.00 equiv) and Picolinaldehyde (3.33 mL, 35.0 mmol, 1.00 equiv) in EtOH (60 mL) was added an aqueous solution of NaOH (28 mL, 2.5 M, 70 mL) at 0 °C. The resultion reaction mixture was stirred at

RT overnight and subsequently extracted with DCM (3 × 60 mL). The combined organic extracts were washed with H_2O (60 mL) and concentrated under reduced pressure. The crude condensation product was used without further purification in the next step.

To a solution of crude 1-cyclopropyl-3-(pyridin-2-yl)prop-2-en-1-one (35.0 mmol, 1.00 equiv) in dry THF (80 mL) cooled to 0°C was added $LiAlH_4$ (3.99 g, 105 mmol, 3.00 equiv). The resulting reaction mixture was stirred at RT overnight and subsequently quenched by the addition of H_2O (4 mL). An aq. NaOH-solution (15%, 4 mL), H_2O (12 mL) and Na_2SO_4 were added in this order. The mixture was filtered and the solvents removed under reduced pressure. Purification by column chromatography of the residue (SiO_2, eluent load, DCM:MeOH, 98:2) afforded alcohol **2i** (746 mg, 4.21 mmol, 12% over two steps) as a pale yellow oil.

IR (film) ν_{max}/cm^{-1} 3352, 3079, 3003, 2921, 1594, 1569, 1477, 1434, 1297, 1150, 1077, 1044, 1017, 1002, 954, 915, 865, 823, 751, 632.
^{1}H NMR ($CDCl_3$, 400 MHz) δ = 8.48 (1H, m), 7.60 (1H, td *J* = 7.6, 1.8 Hz), 7.18 (1H, m) 7.11 (1H, ddd, *J* = 7.5, 4.9, 1.2 Hz), 3.07–2.94 (3H, m), 2.14–2.06 (1H, m), 2.06–2.03 (1H, m), 1.94 (1H, br. s), 0.94 (1H, qt *J* = 8.1, 4.9 Hz,), 0.54–0.44 (2H, m), 0.38–0.29 (1H, m), 0.24–0.15 (1H, m) ppm.
^{13}C NMR ($CDCl_3$, 101 MHz) δ = 161.9, 148.9, 136.8, 123.2, 121.2, 76.1, 36.2, 34.9, 17.9, 2.9, 2.4 ppm.
HRMS (ESI$^+$) Found [M+H]$^+$ = 178.1228; $C_{11}H_{16}NO$ requires 178.1232.

***trans*-2-Phenylcyclopropylmethanol**

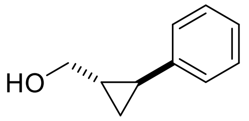

$C_{10}H_{12}O$
148.21 g mol^{-1}

To a solution of *trans*-2-phenylcyclopropane-1-carboxylic acid (1.50 g, 9.25 mmol, 1.00 equiv) in THF (46 mL) at 0 °C was added $BH_3{\cdot}SMe_2$ (1.30 mL, 13.9 mmol, 1.50 equiv) dropwise over 10 min. The mixture was allowed to warm to RT slowly over 22 h. After this time the reaction was cooled to 0 °C, and quenched with MeOH (6.7 mL) and H_2O (21.8 mL). The mixture was extracted with EtOAc (3 × 50 mL) and the combined organics dried ($MgSO_4$) and concentrated *in vacuo*. Purification by column chromatography (SiO_2, solid load, Pentane:Et_2O, 60:40→50:50) afforded alcohol *trans*-2-phenylcyclopropylmethanol (1.36 g, 9.18 mmol, 99%) as a colourless oil.

IR (film) v_{max}/cm^{-1} 3331, 3083, 3064, 3026, 3004, 2919, 2871, 1604, 1497, 1461, 1444, 1413, 1377, 1329, 1309, 1290, 1242, 1218, 1183, 1155, 1126, 1108, 1090, 1065, 1031, 1018, 926.
^{1}H NMR ($CDCl_3$, 400 MHz) δ = 7.26 (2H, app. t, *J* = 7.7, 7.2 Hz), 7.16 (1H, app. t, *J* = 7.6, 7.2 Hz), 7.07 (2H, d, *J* = 7.7 Hz), 3.68–3.57 (2H, m), 1.83 (1H, dt, *J* = 8.7, 4.9 Hz), 1.58–1.50 (1H, br. s), 1.50–1.42 (1H, m), 1.01–0.90 (2H, m) ppm.
^{13}C NMR ($CDCl_3$, 101 MHz) δ = 142.5, 128.5 (2C), 125.9 (2C), 125.8, 66.7, 25.5, 21.4, 14.0 ppm.
HRMS (CI, CH_4) Found $[M+H]^+$ = 149.0955; $C_{10}H_{13}O$ requires 149.0961.

***trans*-2-Phenylcyclopropylethan-1-ol (2j and 2j')**

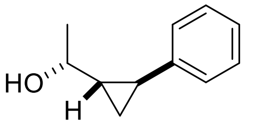

2j (*major*)
$C_{11}H_{14}O$
162.23 g mol^{-1}

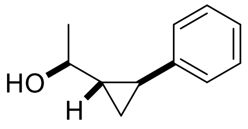

2j' (*minor*)
$C_{11}H_{14}O$
162.23 mol^{-1}

To a solution of oxalyl chloride (0.87 mL, 10.1 mmol, 1.50 equiv) in DCM (15.3 mL) at –78 °C was added DMSO (1.44 mL, 20.3 mmol, 3.00 equiv). The mixture was stirred for 10 min at –78 °C, after which a solution of *trans*-2-phenylcyclopropylmethanol (1.00 g, 6.75 mmol, 1.00 equiv) in DCM (6.1 mL + 2 × 2 mL rinse) was added dropwise over 15 min. Following this, the reaction was stirred for 20 min at –78 °C and then Et_3N (4.70 mL, 33.8 mmol, 5.00 equiv) added dropwise. The suspension was allowed to warm to RT over 50 min and sat. aq. NH_4Cl (30 mL) added. The layers were separated, and the organic layer washed with sat. aq. NH_4Cl (3 × 30 mL), brine (50 mL), dried ($MgSO_4$) and concentrated *in vacuo*. The crude aldehyde was carried through to the next step in the synthesis without further purification.

To a solution of MeMgBr (3 M in THF, 2.93 mL, 8.78 mmol, 1.30 equiv) at RT was added a solution of the crude aldehyde (assumed quant., 6.75 mmol, 1.00 equiv) in THF (3 mL + 2 × 2 mL rinse) dropwise over 15 min (a gentle reflux was observed). The reaction was stirred for 1 h, and then quenched by the addition of sat. aq. NH_4Cl (8 mL). The layers were separated and the aqueous layer extracted with Et_2O (2 × 20 mL). The combined organics were dried ($MgSO_4$) and concentrated *in vacuo*. Purification by column chromatography (SiO_2, eluent load, Pentane:Et_2O, 85:15→80:20→70:30→60:40) afforded diasteromeric alcohols **2j'** (423 mg, 38%) as a colourless solid and **2j** (476 mg, 2.93 mmol, 43%) as a colourless oil.

<u>Spectroscopic data of the major diastereoisomer</u>

IR (film) v_{max}/cm^{-1} 3363, 3085, 3064, 3027, 3002, 2970, 2927, 2873, 1605, 1498, 1455, 1446, 1414, 1370, 1328, 1294, 1260,1219, 1181, 1153, 1105, 1079, 1029, 1001, 975.

^{1}H NMR ($CDCl_3$, 400 MHz) δ = 7.27 (2H, app. t, J = 7.6 Hz), 7.17 (1H, app. t, J = 7.3 Hz), 7.07 (2H, app. d, J = 7.5 Hz), 3.42–3.34 (1H, m), 1.81 (1H, dt, J = 8.8, 5.0 Hz), 1.69 (1H, br. s), 1.35 (3H, d, J = 6.2 Hz), 1.31–1.23 (1H, m), 1.04–0.92 (2H, m) ppm.
^{13}C NMR ($CDCl_3$, 101 MHz) δ = 142.5, 128.5 (2C), 126.0 (2C), 125.8, 71.9, 30.9, 22.9, 20.9, 13.9 ppm.
HRMS (CI, CH_4) Found $[M+H]^+$ = 163.1115; $C_{11}H_{15}O$ requires 163.1117.

<u>Spectroscopic data of the minor diastereoisomer</u>

m.p. = 62–64 °C.
IR (film) v_{max}/cm^{-1} 3275, 3078, 3061, 3029, 3011, 2996, 2978, 2933, 2875, 1603, 1496, 1454, 1448, 1408, 1371, 1307, 1286, 1266, 1221, 1191, 1181, 1115, 1107, 1085, 1065, 1029, 975.
^{1}H NMR ($CDCl_3$, 400 MHz) δ = 7.22 (2H, app. t, J = 7.8, 7.2 Hz), 7.11 (1H, app. t, J = 7.3 Hz), 7.03 (2H, d, J = 7.6 Hz), 3.34 (1H, quint., J = 6.5 Hz), 1.86 (1H, dt, J = 8.6, 5.0 Hz), 1.54 (1H, br. s), 1.29 (3H, d, J = 6.2 Hz), 1.29–1.20 (1H, m), 0.92–0.83 (2H, m) ppm.
^{13}C NMR ($CDCl_3$, 101 MHz) δ = 142.8, 128.5 (2C), 125.9 (2C), 125.7, 72.0, 31.0, 22.6, 21.5, 13.5 ppm.
HRMS (CI, CH_4) Found $[M+H]^+$ = 163.1122; $C_{11}H_{15}O$ requires 163.1117.
The spectroscopic data is in good agreement with previous reports.[13]

1-(Bicyclo[4.1.0]heptan-1-yl)ethan-1-one[14]

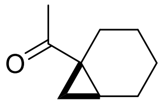

$C_9H_{14}O$
138.21 g mol^{-1}

1-Acetyl-1-cyclohexene (1.86 g, 15.0 mmol, 1.00 equiv), NaH (720 mg, 60% dispersion in mineral oil, 18.0 mmol, 1.20 equiv), trimethylsulfoxonium iodide (3.96 g, 18.0 mmol, 1.20 equiv) and anhydrous DMSO (30 mL, 0.5 M) were subjected to General Procedure **L**. Purification by flash column chromatography (Pentane:Et_2O, 100:0→99:1→98:2) afforded 1-(Bicyclo[4.1.0]heptan-1-yl)ethan-1-one (1.80 g, 13.0 mmol, 43%) as a colourless oil.

IR (film) v_{max}/cm^{-1} 3073, 3000, 2930, 2860, 1681, 1451, 1427, 1384, 1352, 1303, 1292, 1274, 1251, 1239, 1165, 1149, 1130, 1113, 1071, 1030, 977, 938, 925, 902, 833, 799, 748, 662.
^{1}H NMR ($CDCl_3$, 500 MHz) δ = 2.49 (1H, dt, J = 13.4, 6.4 Hz), 2.04 (3H, s), 1.90 (1H, td, J = 13.3, 7.2 Hz), 1.76–1.69 (1H, m), 1.69–1.63 (1H, m), 1.60 (1H, dtd, J = 9.6, 6.9, 1.8 Hz), 1.33 (1H, ddd, J = 9.6, 4.4, 0.8 Hz), 1.33–1.24 (3H, m), 1.24–1.15 (1H, m), 0.72 (1H, dd, J = 6.9, 4.3 Hz) ppm.
^{13}C NMR ($CDCl_3$, 126 MHz) δ = 209.9, 31.5, 25.5, 24.5, 23.4, 22.8, 21.8, 21.4, 20.6 ppm.

HRMS (ESI^+) Found $[M+H]^+$ = 139.1118; $C_9H_{15}O$ requires 139.1117.

The product is fairly volatile, and care should be taken when concentrating *in vacuo*.

***rel*-(*R*/*S*)-1-((1*R*,6*R*)-Bicyclo[4.1.0]heptan-1-yl)ethan-1-ol (*rel*-2l)**

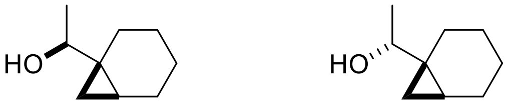

2l

$C_9H_{16}O$

140.23 g mol^{-1}

To a solution of 1-(Bicyclo[4.1.0]heptan-1-yl)ethan-1-one (1.80 g, 13.0 mmol, 1.00 equiv) in anhydrous DCM (52 mL) cooled to –78 ºC was added DIBAL-H (16.3 mL, 1.0 M in DCM, 16.3 mmol, 1.25 equiv) over 5 min. The reaction mixture was warmed to 0 ºC and stirred for 2h. The reaction was quenched by the addition of sat. aq. Rochelle's salt, stirred for 1 h and diluted with brine. After separation of the layers, the aqueos layer was extracted with DCM. The combined organics are dried over $NaSO_4$ and the solvent removed under reduced pressure. Purification by flash column chromatography (Pentane:Et_2O, 50:50) afforded an inseparable mixture of alcohols **2l** (1.52 g, 10.7 mmol, 82%) as a colourless oil.

IR (film) ν_{max}/cm^{-1} 3353 (br.), 3056, 2972, 2926, 2855, 1451, 1403, 1368, 1348, 1327, 1292, 1260, 1219, 1179, 1156, 1108, 1098, 1066, 1024, 1012, 983, 949, 927, 892, 865, 841, 804, 765.

^{1}H NMR ($CDCl_3$, 500 MHz, isomer A) δ = 3.08 (1H, q, *J* = 6.4 Hz), 1.96–1.87 (1H, m), 1.83–1.73 (1H, m), 1.71–1.62 (1H, m), 1.49 (1H, ddd, *J* = 13.8, 8.7, 4.9 Hz), 1.41 (1H, s), 1.33–1.19 (2H, m), 1.18 (3H, d, *J* = 6.4 Hz), 1.22–1.12 (1H, m), 1.13–1.07 (1H, m), 0.75 (1H, dddd, *J* = 9.4, 6.8, 5.6, 1.6 Hz), 0.51 (1H, ddd, *J* = 9.4, 4.3, 0.8 Hz), 0.25 (1H, ddd, *J* = 5.6, 4.3, 0.5 Hz) ppm.

^{13}C NMR ($CDCl_3$, 126 MHz, isomer A) δ = 76.2, 24.8, 23.8, 23.5, 22.19, 21.3, 19.4, 16.5, 15.2 ppm.

^{1}H NMR ($CDCl_3$, 500 MHz, isomer B) δ = 2.98 (1H, q, *J* = 6.4 Hz), 1.96–1.87 (1H, m), 1.83–1.73 (1H, m), 1.65–1.57 (1H, m), 1.54 (1H, ddd, *J* = 13.4, 6.9, 5.3 Hz), 1.41 (1H, s), 1.33–1.19 (2H, m), 1.17 (3H, d, *J* = 6.4 Hz), 1.22–1.12 (2H, m), 0.82 (1H, dddd, *J* = 9.0, 7.0, 5.5, 1.7 Hz), 0.40 (1H, ddd, *J* = 9.3, 4.6, 0.8 Hz), 0.22 (1H, ddd, *J* = 5.5, 4.5, 0.5 Hz) ppm.

^{13}C NMR ($CDCl_3$, 126 MHz, isomer B) δ = 76.7, 25.1, 24.1, 22.7, 22.2, 21.5, 18.7, 17.7, 15.0 ppm.

HRMS (CH_4 CI^+) Found $[M+H]^+$ = 141.1273; $C_9H_{17}O$ requires 141.1274.

The d.r. is 1.2:1, but it is not possible to distinguish between either isomers.

2-Methylenecyclohexan-1-ol[15,16]

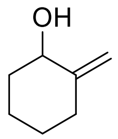

$C_7H_{12}O$
112.17 g mol^{-1}

A well-stirred suspension of trimethylsulfonium iodide (20.4 g, 100 mmol, 4.00 equiv) in anhydrous THF (50 mL) was cooled to –10 °C, then *n*-BuLi (61 mL, 1.52 M in hexanes, 92.7 mmol, 3.70 equiv) was carefully added dropwise over 30 min (caution: effervescence and exotherm), then stirred for a further 30 min at the same temperature. A solution of cyclohexene oxide (2.53 mL, 2.45 g, 25.0 mmol, 1.00 equiv) in anhydrous THF (75 mL, final concentration 0.2 M) was added over 5 min, then the reaction was warmed to 0 °C and stirred for 1 h, before warming to RT and stirring for a further 2 h. The reaction mixture was cooled to around –10 to 0 °C, then quenched with sat. aq. NH_4Cl (50 mL) and diluted with H_2O (25 mL) and Et_2O (25 mL). The layers were separated, the aqueous layer extracted with Et_2O (3 × 30 mL), and the combined organics dried (Na_2SO_4) and concentrated *in vacuo*. Purification by flash column chromatography (Pentane:Et_2O, 95:5→90:10→85:15) afforded 2-methylenecyclohexan-1-ol (900 mg, 8.02 mmol, 32%) as a volatile colourless oil.

IR (film) v_{max}/cm^{-1} 3348 (br.), 3089, 2988, 2931, 2857, 1654, 1446, 1397, 1343, 1287, 1245, 1231, 1150, 1120, 1078, 1034, 993, 964, 944, 925, 896, 857, 826.
^{1}H NMR ($CDCl_3$, 500 MHz) δ = 4.88 (1H, q, *J* = 1.5 Hz), 4.75 (1H, quint., *J* = 1.4 Hz), 4.09 (1H, dd, *J* = 9.0, 4.4 Hz), 2.40 (1H, dddt, *J* = 13.5, 5.2, 4.0, 1.2 Hz), 2.05–1.99 (1H, m), 1.96 (1H, dddd, *J* = 10.5, 4.6, 3.1, 1.4 Hz), 1.81 (1H, dtdd, *J* = 12.4, 5.4, 3.9, 1.9 Hz), 1.68–1.60 (2H, m), 1.47 (1H, qt, *J* = 10.4, 3.4 Hz), 1.44–1.33 (2H, m) ppm.
^{13}C NMR ($CDCl_3$, 126 MHz) δ = 151.8, 105.2, 72.8, 36.8, 33.6, 27.8, 23.9 ppm.
A stable molecular ion could not be detected by ESI, APCI or EI.
The spectroscopic data is in good agreement with previous reports.[15,16]

Spiro[2.5]octan-4-ol (2m)[17]

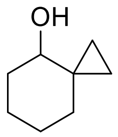

2m
$C_8H_{14}O$
126.20 g mol^{-1}

A solution of Et_2Zn (32 mL, 1.0 M in hexanes, 32 mmol, 4.0 equiv) in anhydrous DCM (40 mL) was cooled to –20 °C, then diiodomethane (5.16 mL, 17.1 g, 64.0 mmol, 8.00 equiv) was *very*

carefully added over 15 min, during which time voluminous white precipitates are gradually formed, then stirred for a further 10 min at the same temperature. A solution of allylic alcohol 2-methylenecyclohexan-1-ol (900 mg, 8.02 mmol, 1.00 equiv) in anhydrous DCM (10 mL, final concentration 0.1 M) was *very* carefully added over 30 min while ensuring that the reaction temperature does not rise above –10 °C, then stirred for a further 30 min at –20 to –10 °C. The reaction was then warmed to 0 °C and stirred for another 5 h. The reaction was quenched at 0 °C by the *very* careful dropwise addition of sat. aq. NH_4Cl (50 mL), then diluted with H_2O (50 mL). The layers were separated, the aqueous layer extracted with DCM (4 × 30 mL), and the combined organics dried (Na_2SO_4) and concentrated *in vacuo*. Purification by flash column chromatography (Pentane:Et_2O, 95:5→90:10) afforded alcohol **2m** (901 mg, 7.14 mmol, 89%) as a colourless oil.

IR (film) v_{max}/cm^{-1} 3356 (br.), 3071, 2998, 2926, 2853, 1445, 1427, 1349, 1291, 1268, 1216, 1165, 1149, 1138, 1105, 1074, 1044, 1014, 1002, 981, 932, 923, 899, 882, 860, 840, 802, 722, 686.

^{1}H NMR ($CDCl_3$, 500 MHz) δ = 3.19 (1H, br. t, *J* = 3.8 Hz), 1.74 (1H, br. t, *J* = 10.5 Hz), 1.71–1.68 (1H, m), 1.68–1.60 (2H, m), 1.61–1.52 (1H, m), 1.52–1.45 (1H, m), 1.45–1.37 (1H, m), 1.38 (1H, s), 0.96 (1H, br. dt, *J* = 13.7, 4.6 Hz), 0.46–0.41 (1H, m), 0.41–0.36 (1H, m), 0.28–0.24 (1H, m), 0.24–0.19 (1H, m) ppm.

^{13}C NMR ($CDCl_3$, 126 MHz) δ = 73.9, 32.7, 31.9, 25.1, 23.8, 21.5, 10.4, 9.5 ppm.

HRMS (CH_4 CI^+) Found $[M+H]^+$ = 127.1120; $C_8H_{15}O$ requires 127.1117.

The spectroscopic data is in good agreement with previous reports.[13,14,15]

3-Cyclopropyl-3-hydroxy-1-(2,3,4,5,6-pentamethylphenyl)pentan-1-one (7)

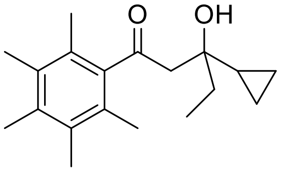

7

$C_{19}H_{28}O_2$

288.43 g mol^{-1}

To a solution of 1-(2,3,4,5,6-pentamethylphenyl)ethan-1-one (**1**, 1.00 g, 5.26 mmol, 1.00 equiv) in Et_2O (12 mL) cooled to 0 °C was added MeMgBr (3 M in Et_2O, 1.90 mL, 5.26 mmol, 1.00 equiv) dropwise. The solution was heated to 40 °C and stirred for 1 h, during which time a white precipitate formed. To this mixture was added 1-cyclopropylpropan-1-one (567 mg, 5.78 mmol, 1.10 equiv) in Et_2O (4 mL) dropwise. The reaction was maintained at RT for 3 h and quenched with sat. aq. NH_4Cl (10 mL). The mixture was extracted with Et_2O (3 × 20 mL)

and the combined organics washed with H_2O (20 mL), brine (20 mL), dried (Na_2SO_4) and concentrated under reduced pressure. Purification by column chromatography (SiO_2, eluent load, Pentane:Et_2O, 95:3.5) afforded β-hydroxy ketone **7** (610 mg, 2.11 mmol, 40%) as a colourless oil.

IR (film) ν_{max}/cm^{-1} 3751, 3340, 2922, 2851, 2361, 2341, 1736, 1664, 1497, 1447, 1421, 1292, 1235, 1080, 1023, 953, 923, 772, 723, 626, 612.
^{1}H NMR ($CDCl_3$, 400 MHz) δ = 3.87 (1H, s), 2.96 (1H, d, J = 19.2 Hz), 2.89 (1H, d, J = 19.2 Hz), 2.24 (3H, s), 2.19 (6H, s), 2.17 (6H, s), 1.81–1.69 (2H, m), 0.99 (3H, t, J = 7.6 Hz), 0.89 (1H, tt, J = 8.4, 5.5 Hz), 0.61–0.54 (2H, m), 0.41–0.34 (2H, m) ppm.
^{13}C NMR ($CDCl_3$, 101 MHz) δ = ^{13}C NMR (101 MHz, $CDCl_3$) δ 214.3, 140.1, 135.9, 133.4 (2C), 127.4 (2C), 71.7, 53.3, 34.3, 19.3, 17.1 (2C), 16.8, 16.1 (2C), 8.4, 0.9, 0.4 ppm.
HRMS (ESI^+) Found $[M+Na]^+$ = 311.1982; $C_{19}H_{28}O_2Na$ requires 311.1982.

(*E/Z*)-3-Cyclopropyl-1-(2,3,4,5,6-pentamethylphenyl)pent-2-en-1-one ((*E/Z*)-6)

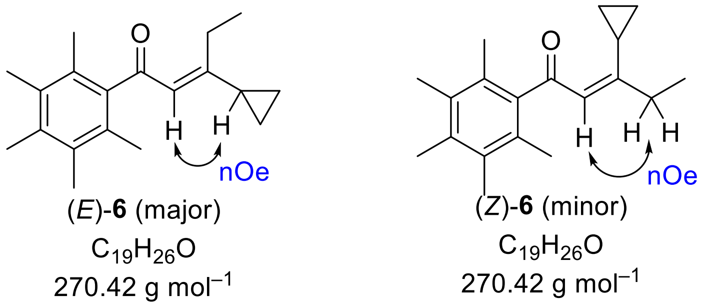

(*E*)-**6** (major)
$C_{19}H_{26}O$
270.42 g mol^{-1}

(*Z*)-**6** (minor)
$C_{19}H_{26}O$
270.42 g mol^{-1}

To a solution of **1** (519 mg, 1.80 mmol, 1.00 equiv) in DCM (2 mL) was added Et_3N (1.07 mL, 7.74 mmol, 4.30 equiv) and DMAP (24 mg, 0.24 mmol, 0.11 equiv) sequentially. The mixture was cooled to 0 °C and a solution of trifluoroacetic anhydride (503 µL, 3.60 mmol, 2.00 equiv) in DCM (4 mL) added dropwise. The mixture was warmed to RT slowly over 16 h, after which sat. aq. NH_4Cl (20 mL), H_2O (5 mL) and Et_2O (20 mL) were added sequentially. The layers were separated and the aqueous layer extracted with Et_2O (2 × 20 mL). The combined organics were dried ($MgSO_4$) and concentrated *in vacuo*. Purification by column chromatography (SiO_2, eluent load, Pentane:Et_2O, 97:3) afforded Enones (*E/Z*)-**6** (238 mg, 880 µmol, 49%) in a 55:45 ratio (*E*:*Z*) as a colourless solid along with an unclean fraction of Enones (*E/Z*)-**8** (127 mg, 470 µmol, ≈ 26%) in a (*E*:*Z*) ratio of 59:41.

m.p. = 101 °C.
IR (film) ν_{max}/cm^{-1} 2977, 2936, 1663, 1581, 1454, 1341, 1309, 1265, 1176, 1122, 1067, 1018, 963, 941, 921, 884, 884, 829, 699, 616.

^{1}H NMR ($CDCl_3$, 400 MHz, *E*-isomer, major) δ = 6.03 (1H, s), 2.59 (2H, q, *J* = 7.5 Hz), 2.30–2.06 (15H, m), 1.47 (1H, tt, *J* = 7.9, 4.9 Hz), 1.18 (3H, t, *J* = 7.5 Hz), 0.94–0.83 (2H, m), 0.73–0.64 (2H, m) ppm.

^{13}C NMR ($CDCl_3$, 101 MHz, *E*-isomer, major) δ = 200.1, 168.4, 142.3, 135.0, 133.0 (2C), 128.1 (2C), 121.5, 24.7, 18.7, 17.2 (2C), 16.8, 16.1 (2C), 13.7, 8.6 (2C) ppm

^{1}H NMR ($CDCl_3$, 400 MHz, *Z*-isomer, minor) δ = 6.27 (1H, s), 3.23 (1H, tt, *J* = 8.5, 5.3 Hz), 2.30–2.06 (15H, m), 1.77 (2H, q, *J* = 7.3 Hz), 1.02 (3H, t, *J* = 7.4 Hz), 0.94–0.83 (2H, m), 0.78 (1H, dd, *J* = 5.4, 2.2 Hz) ppm.

^{13}C NMR ($CDCl_3$, 126 MHz, *Z*-isomer, minor) δ = 201.53, 166.19, 142.3, 135.0, 133.0 (2C), 128.1 (2C), 125.40, 24.04, 17.3 (2C), 16.8, 16.1 (2C), 13.8, 13.5, 7.7 (2C) ppm.

HRMS (ESI$^+$) Found $[M+H]^+$ = 271.2056; $C_{19}H_{27}O$ requires 271.2062.

(*E/Z*)-3-cyclopropyl-1-(2,3,4,5,6-pentamethylphenyl)pent-3-en-1-one ((*E/Z*)-8)

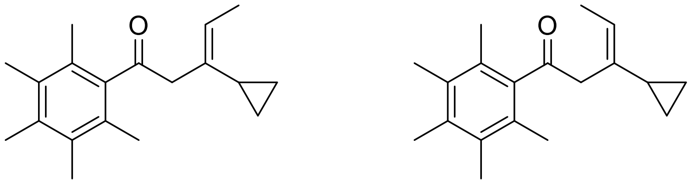

(*Z*)-**8**
$C_{19}H_{26}O$
270.42 g mol^{-1}

(*E*)-**8**
$C_{19}H_{26}O$
270.42 g mol^{-1}

IR (film) v_{max}/cm^{-1} 2984, 2916, 2161, 1702, 1665, 1589, 1447, 1404, 1315, 1257, 1175, 1107, 1067, 1020, 994, 926, 881, 819, 784, 727, 689, 638.

^{1}H NMR ($CDCl_3$, 400 MHz, major, selected signals) δ = 5.43 (1H, q, *J* = 6.8), 3.23 (2H, s), 2.26–2.23 (3H, m), 2.22–2.16 (6H, m), 1.80 (3H, dd, *J* = 6.8, 1.1 Hz), 0.74–0.67 (2H, m), 0.62–0.55 (1H, m), 0.49–0.41 (2H, m).ppm.

^{13}C NMR ($CDCl_3$, 101 MHz, major, selected signals) δ = 210.3, 127.0, 52.1, 14.0, 5.4, 5.3, 5.3 ppm

^{1}H NMR ($CDCl_3$, 400 MHz, minor selected signals) δ 5.53 (1H, qd, *J* = 6.8, 1.4 Hz), 3.52 (1H, s), 2.26–2.23 (3H, m), 2.22–2.16 (6H, m), 1.56 (3H, dd, *J* = 6.8, 1.3 Hz), 0.74–0.67 (2H, m), 0.62–0.55 (1H, m), 0.49–0.41 (2H, m) ppm.

^{13}C NMR ($CDCl_3$, 126 MHz, minor, selected signals) δ = 208.9, 122.0, 48.2, 13.7, 5.4, 5.3, 5.3 ppm.

HRMS (ESI$^+$) Found $[M+H]^+$ = 271.2057; $C_{19}H_{27}O$ requires 271.2062.

***trans*-(2-Ethylcyclopentyl)(2,3,4,5,6-pentamethylphenyl)methanone (4a)**

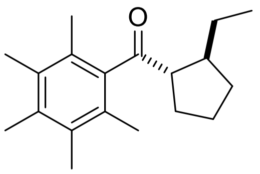

4a
$C_{19}H_{28}O$
272.43 g mol^{-1}

Ketone **1** (114 mg, 0.600 mmol, 1.00 equiv), [Ir(COD)Cl]$_2$ (4.03 mg, 1 mol%), cataCXium® A (17.2 mg, 8 mol%), KO*t*-Bu (67.3 mg, 0.600 µmol, 1.00 equiv), 1-cyclopropylpropan-1-ol (**2a**, 120 mg, 1.20 mmol, 2.00 equiv) and toluene (150 µL, 4 M) were subjected to **General Procedure A** at 125 °C. The crude reaction mixture showed a d.r. of 99:1. Purification by flash column chromatography (Pentane:Et_2O, 98.5:1.5) afforded ketone **4a** (112 mg, 0.411 mmol, 69%) as a colourless solid.

m.p. = 52–54 °C.
IR (film) v_{max}/cm^{-1} 2953, 2869, 1684, 1448, 1377, 1342, 1302, 1265, 1168, 1125, 1066, 1003, 959, 836, 713, 692.
^{1}H NMR ($CDCl_3$, 400 MHz) δ = 2.87 (1H, dt, *J* = 8.6, 7.1 Hz), 2.28–2.20 (4H, m), 2.18 (6H, s), 2.11 (6H, s), 1.99–1.86 (2H, m), 1.85–1.75 (1H, m), 1.72–1.58 (2H, m), 1.54–1.46 (1H, m), 1.32–1.22 (1H, m), 1.21–1.10 (1H, m), 0.86 (3H, t, *J* = 7.3 Hz) ppm.
An indicative signal for what we presume to be the minor diastereoisomer can be found at δ = 3.24 (1H, td, *J* = 7.7, 6.0 Hz) in the crude reaction mixture.
^{13}C NMR ($CDCl_3$, 101 MHz) δ = 214.6, 141.1, 135.4, 133.2 (2C), 127.9 (2C), 60.1, 43.9, 32.2, 29.9, 28.8, 25.2, 18.1 (2C), 16.9, 16.2 (2C), 12.8 ppm.
HRMS (ESI$^+$) Found [M+H]$^+$ = 273.2213; $C_{19}H_{29}O$ requires 273.2213.
The relative stereochemistry of the product was confirmed by single crystal X-ray diffraction.

^{1}H NMR ($CDCl_3$, 400 MHz) crude reaction mixture

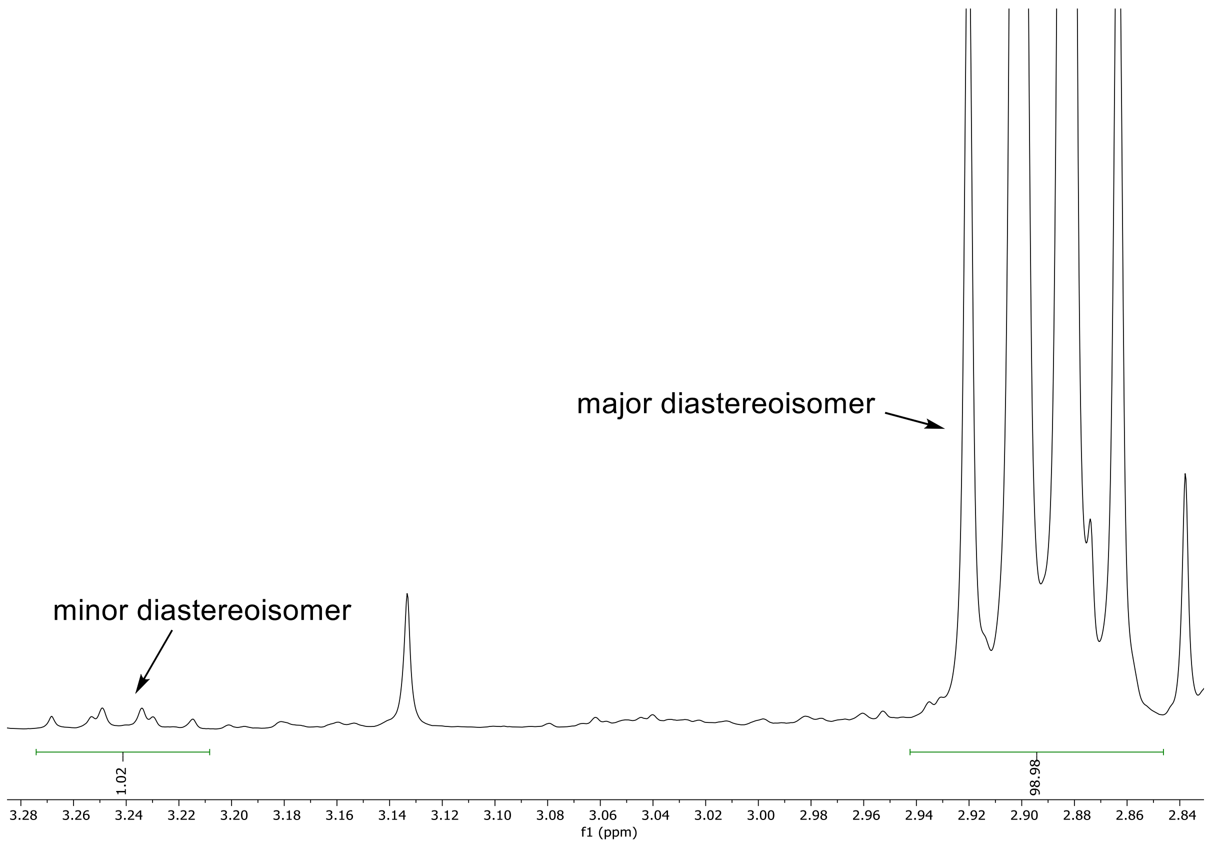

***trans*-(2-Butylcyclopentyl)(2,3,4,5,6-pentamethylphenyl)methanone (4b)**

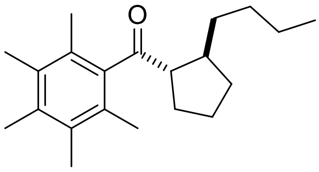

4b

$C_{21}H_{32}O$

300.49 g mol^{-1}

Ketone **1** (114 mg, 0.600 mmol, 1.00 equiv), $[Ir(COD)Cl]_2$ (4.03 mg, 1 mol%), cataCXium® A (17.2 mg, 8 mol%), KO*t*-Bu (67.3 mg, 0.600 µmol, 1.00 equiv), 1-cyclopropylpentan-1-ol (**4b**, 153 mg, 1.20 mmol, 2.00 equiv) and toluene (150 µL, 4 M) were subjected to **General Procedure A** at 125 °C. Purification by flash column chromatography (Pentane:Et_2O, 98:4) afforded ketone **4b** (107 mg, 0.353 mmol, 59%) as pale yellow oil.

IR (film) ν_{max}/cm^{-1} 2953, 2927, 2870, 2361, 2341, 1690, 1454, 1381, 1304, 1120, 999, 669.

^{1}H NMR ($CDCl_3$, 400 MHz) δ = 2.85 (1H, m_c), 2.32–2.21 (4H, m), 2.18 (6H, s), 2.11 (6H, s), 1.98–1.87 (2H, m), 1.85–1.74 (1H, m), 1.69–1.59 (2H, m), 1.49–1.40 (1H, m), 1.32–1.07 (6H, m), 0.86 (3H, m_c) ppm.

^{13}C NMR ($CDCl_3$, 101 MHz) δ = 214.6, 141.1, 135.4, 133.2 (2C), 128.0 (2C), 60.4, 42.2, 35.7, 32.6, 30.7, 29.8, 25.2, 22.9, 18.1 (2C), 16.9, 16.2 (2C), 14.2 ppm.

HRMS (ESI$^+$) Found $[M+H]^+$ = 301.2527; $C_{21}H_{33}O$ requires 301.2531.
The relative stereochemistry was assigned by analogy to **4a**. Signals for the minor diastereoisomer were not found by ^{1}H NMR analysis of the crude reaction mixture.

***trans*-2-Cyclobutylcyclopentyl)(2,3,4,5,6-pentamethylphenyl)methanone (4c)**

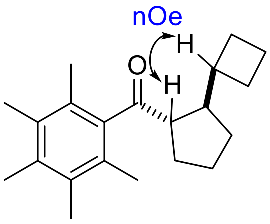

4c
$C_{21}H_{30}O$
298.47 g mol^{-1}

Ketone **1** (114 mg, 0.600 mmol, 1.00 equiv), $[Ir(COD)Cl]_2$ (4.03 mg, 1 mol%), cataCXium® A (17.2 mg, 8 mol%), KO*t*-Bu (67.3 mg, 0.600 mmol, 1.00 equiv), cyclobutyl(cyclopropyl) methanol (**2c**, 151 mg, 1.20 mmol, 2.00 equiv) and toluene (150 µL, 4 M) were subjected to **General Procedure A** at 125 °C. Purification by flash column chromatography (Pentane:Et_2O, 99:1→96:4) afforded ketone **4c** (140 mg, 0.469 mmol, 78%) as a colourless solid.

m.p. = 71–73 °C.
IR (film) ν_{max}/cm^{-1} 2948, 2866, 1688, 1447, 1382, 1338, 1304, 1256, 1126, 997, 942, 854.
^{1}H NMR ($CDCl_3$, 400 MHz) δ = 2.84 (1H, dt, J = 8.5, 5.3 Hz), 2.45 (1H, m), 2.26–2.16 (10H, m), 2.12 (6H, m), 1.99–1.49 (11H, m), 1.37 (1H, m) ppm.
^{13}C NMR ($CDCl_3$, 101 MHz) δ = 213.9, 140.6, 135.3, 133.1 (2C), 128.0 (2C), 58.0, 46.4, 40.7, 29.5, 29.4, 27.0, 26.4, 25.3, 18.2 (2C), 17.8, 16.8, 16.2 (2C) ppm.
HRMS (ESI$^+$) Found $[M+H]^+$ = 299.2370; $C_{21}H_{31}O$ requires 299.2369.
Signals for the minor diastereoisomer were not found by ^{1}H NMR analysis of the crude reaction mixture.

***trans*-(2,3,4,5,6-Pentamethylphenyl)(*trans*-2-phenethylcyclopentyl)methanone (4d)**

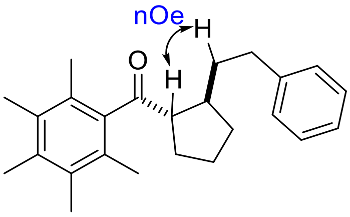

4d
$C_{25}H_{32}O$
348.53 g mol^{-1}

Ketone **1** (114 mg, 0.600 mmol, 1.00 equiv), $[Ir(COD)Cl]_2$ (4 mg, 1 mol%), cataCXium® A (17.2 mg, 8 mol%), KO*t*-Bu (67.3 mg, 0.600 mmol, 1.00 equiv), 1-cyclopropyl-3-

phenylpropan-1-ol (**2d**, 212 mg, 1.20 mmol, 2.00 equiv) and toluene (150 μL, 4 M) were subjected to **General Procedure A** at 125 °C. Purification by flash column chromatography (Pentane:Et_2O, 98:2) afforded ketone **4d** (131 mg, 0.378 mmol, 63%) as a colourless solid.

m.p. = 81–83 °C.
IR (film) ν_{max}/cm^{-1} 2934, 2863, 1685, 1454, 1382, 1303, 1261, 1133, 1091, 1004, 838, 748, 699.
^{1}H NMR ($CDCl_3$, 400 MHz) δ = 7.30–7.24 (2H, m), 7.22–7.16 (1H, m), 7.13–7.06 (2H, m), 2.93 (1H, m), 2.64 (1H, ddd, J = 13.6, 10.2, 5.4 Hz), 2.55 (1H, ddd, J = 13.7, 9.9, 6.5 Hz), 2.39–2.29 (1H, m), 2.27 (3H, s), 2.20 (6H, s), 2.16–1.96 (8H, m), 1.92–1.65 (4H, m), 1.50 (1H, dtd, J = 13.2, 9.8, 5.4 Hz), 1.39 (1H, dq, J = 12.4, 7.6 Hz) ppm.
^{13}C NMR ($CDCl_3$, 101 MHz) δ = 214.0, 142.5, 140.9, 135.4, 133.1 (2C), 128.4 (2C), 128.4 (2C), 128.0 (2C), 125.7, 60.2, 41.8, 38.0, 34.8, 32.6, 29.4, 25.2, 18.1 (2C), 16.8, 16.1 (2C) ppm.
HRMS (ESI$^+$) Found [M+H]$^+$ = 349.2525; $C_{25}H_{33}O$ requires 349.2531.
Signals for the minor diastereoisomer were not found by ^{1}H NMR analysis of the crude reaction mixture.

***trans*-(2,3,4,5,6-Pentamethylphenyl)(2-(3-phenylpropyl)cyclopentyl)methanone) (4e)**

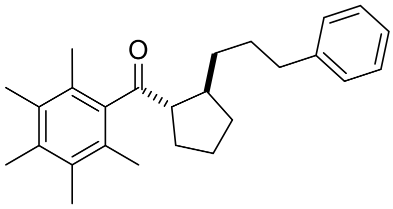

4e
$C_{26}H_{34}O$
362.56 g mol^{-1}

Ketone **1** (114 mg, 0.600 mmol, 1.00 equiv), $[Ir(COD)Cl]_2$ (4.03 mg, 1 mol%), cataCXium® A (17.2 mg, 8 mol%), KO*t*-Bu (67.3 mg, 0.600 mmol, 1.00 equiv), 1-cyclopropyl-4-phenylbutan-1-ol (**2e**, 228 mg, 1.20 mmol, 2.00 equiv) and toluene (150 μL, 4 M) were subjected to **General Procedure A** at 125 °C. Purification by flash column chromatography (Pentane:Et_2O, 96:4) afforded ketone **4e** (171 mg, 0.468 mmol, 79%) as a colourless oil.

IR (film) ν_{max}/cm^{-1} 2932, 1687, 1496, 1496, 1452, 1382, 1304, 1259, 1119, 1069, 998, 842, 747, 698.
^{1}H NMR ($CDCl_3$, 400 MHz) δ = 7.37–7.30 (2H, m), 7.28–7.21 (1H, m), 7.19–7.17 (2H, m), 3.03–2.84 (1H, m), 2.60 (2H, m), 2.47–2.37 (1H, m), 2.30 (3H, s), 2.24 (6H, s), 2.17 (6H, s), 2.07–1.94 (2H, m), 1.94–1.80 (1H, m), 1.80–1.53 (5H, m), 1.39–1.25 (2H, m) ppm.

^{13}C NMR ($CDCl_3$, 101 MHz) δ = 214.3, 142.7, 140.9, 135.4, 133.1 (2C), 128.4 (2C), 128.3 (2C), 127.9 (2C), 125.7, 60.2, 41.9, 36.0, 35.6, 32.6, 30.2, 29.7, 25.2, 18.0 (2C), 16.8, 16.1 (2C) ppm.
HRMS (ESI^+) Found $[M+H]^+$ = 363.2683; $C_{26}H_{35}O$ requires 363.2688.
The relative stereochemistry was assigned by analogy to **4a**. Signals for the minor diastereoisomer were not found by ^{1}H NMR analysis of the crude reaction mixture.

***trans*-(2-(3-(Benzyloxy)propyl)cyclopentyl)(2,3,4,5,6-pentamethylphenyl)methanone (4f)**

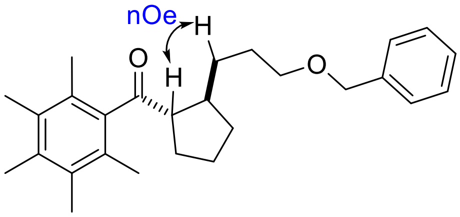

4f
$C_{27}H_{36}O_2$
392.58 g mol^{-1}

Ketone **1** (114 mg, 0.600 mmol, 1.00 equiv), $[Ir(COD)Cl]_2$ (4.03 mg, 1 mol%), cataCXium® A (17.2 mg, 8 mol%), KO*t*-Bu (67.3 mg, 0.600 mmol, 1.00 equiv), 1-cyclopropyl-4-benzyloxybutan-1-ol (**2f**, 264 mg, 1.20 mmol, 2.00 equiv) and toluene (150 µL, 4 M) were subjected to **General Procedure A** at 125 °C. Purification by flash column chromatography (Pentane:Et_2O, 98:2→95:5) afforded ketone **4f** (175 mg, 0.446 mmol, 74%) as a colourless oil.

IR (film) ν_{max}/cm^{-1} 3087, 3062, 3028, 2936, 2856, 2793, 1687, 1573, 1495, 1453, 1410, 1382, 1361, 1305, 1258, 1204, 1154, 1100, 1027, 999, 945, 916, 848, 734, 697.
^{1}H NMR ($CDCl_3$, 500 MHz) δ = 7.36–7.25 (5H, m), 4.47 (2H, s), 3.46–3.35 (2H, m), 2.87 (1H, dt, *J* = 8.7, 7.0 Hz), 2.36–2.26 (1H, m), 2.22 (3H, s), 2.17 (6H, s), 2.10 (6H, s), 1.99–1.88 (2H, m), 1.85–1.77 (1H, m), 1.71–1.51 (6H, m), 1.33–1.18 (2H, m) ppm.
^{13}C NMR ($CDCl_3$, 126 MHz) δ = 214.3, 141.0, 138.8, 135.4, 133.2 (2C), 128.5 (2C), 128.0 (2C), 127.7 (2C), 127.6, 72.9, 70.6, 60.3, 42.0, 32.5, 32.5, 29.8, 28.7, 25.1, 18.1 (2C), 16.9, 16.2 (2C) ppm.
HRMS (ESI^+) Found $[M+H]^+$ = 393.2791; $C_{27}H_{37}O_2$ requires 393.2788.
Signals for the minor diastereoisomer were not found by ^{1}H NMR analysis of the crude reaction mixture.

***trans*-(2-(3-(Dibenzylamino)propyl)cyclopentyl)(2,3,4,5,6-pentamethylphenyl)methanone (4g)**

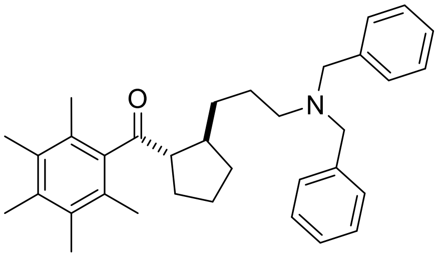

4g
$C_{34}H_{43}NO$
481.72 g mol^{-1}

Ketone **1** (114 mg, 0.600 mmol, 1.00 equiv), [Ir(COD)Cl]$_2$ (4.03 mg, 1 mol%), cataCXium® A (17.2 mg, 8 mol%), KO*t*-Bu (67.3 mg, 0.600 mmol, 1.00 equiv), 1-cyclopropyl-4-(dibenzylamino)butan-1-ol (**2g**, 371 mg, 1.20 mmol, 2.00 equiv) and toluene (150 µL, 4 M) were subjected to **General Procedure A** at 125 °C. Purification by flash column chromatography (Pentane:Et_2O, 98:2→96:4→94:6) afforded ketone **4g** (207 mg, 0.430 mmol, 72%) as a colourless oil.

IR (film) ν_{max}/cm^{-1} 3084, 3061, 3026, 2939, 2866, 2794, 1688, 1601, 1574, 1494, 1452, 1381, 1366, 1345, 1304, 1257, 1207, 1155, 1123, 1070, 1027, 998, 977, 910, 844, 824, 742, 698.
^{1}H NMR ($CDCl_3$, 500 MHz) δ = 7.35–7.27 (8H, m), 7.25–7.20 (2H, m), 3.50 (4H, s), 2.84 (1H, dt, *J* = 8.6, 7.0 Hz), 2.39–2.19 (6H, m), 2.16 (6H, s), 2.09 (6H, s), 1.97–1.86 (2H, m), 1.86–1.83 (1H, m), 1.70–1.55 (2H, m), 1.52–1.37 (3H, m), 1.30–1.18 (1H, m), 1.17–1.07 (1H, m) ppm.
^{13}C NMR ($CDCl_3$, 126 MHz) δ = 214.3, 141.0, 140.1 (2C), 135.4, 133.2 (2C), 128.8 (4C), 128.3 (4C), 128.0 (2C), 126.8 (2C), 60.3, 58.3 (2C), 53.6, 42.1, 33.8, 32.7, 29.8, 25.8, 25.2, 18.1 (2C), 16.8, 16.2 (2C) ppm.
HRMS (ESI$^+$) Found [M+H]$^+$ = 482.3416; $C_{34}H_{44}NO$ requires 482.3417.
The relative stereochemistry was assigned by analogy to **4a**. Signals for the minor diastereoisomer were not found by ^{1}H NMR analysis of the crude reaction mixture.

***trans*-(2-(3-(Methylthio)propyl)cyclopentyl)(2,3,4,5,6-pentamethylphenyl)methanone (4h)**

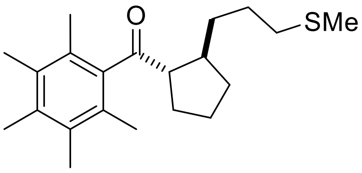

4h
$C_{21}H_{32}OS$
332.55 g mol^{-1}

Ketone **1** (114 mg, 0.600 mmol, 1.00 equiv), $[Ir(COD)Cl]_2$ (4.03 mg, 1 mol%), cataCXium® A (17.2 mg, 8 mol%), KO*t*-Bu (67.3 mg, 0.600 mmol, 1.00 equiv), 1-cyclopropyl-4-(methylthio)butan-1-ol (**2h**, 192 mg, 1.20 mmol, 2.00 equiv) and toluene (150 µL, 4 M) were subjected to **General Procedure A** at 125 °C. The crude reaction mixture showed a d.r of ≥99:1. Purification by flash column chromatography (Pentane:Et_2O, 96:4) afforded ketone **4h** (130 mg, 0.390 mmol, 65%) as a yellow solid.

m.p. = 50–51 °C.
IR (film) ν_{max}/cm^{-1} 2933, 2859, 1686, 1446, 1384, 1302, 1265, 1175, 1129, 1066, 1021, 1003, 957, 838, 696.
^{1}H NMR ($CDCl_3$, 400 MHz) δ = 2.85 (1H, dt, *J* = 8.7, 7.0 Hz), 2.48–2.36 (2H, m), 2.35–2.25 (1H, m), 2.23 (3H, s), 2.18 (6H, s), 2.11 (6H, s), 2.05 (3H, s), 2.00–1.86 (2H, m) 1.86–1.75 (1H, m), 1.71–1.49 (5H, m), 1.33–1.20 (2H, m) ppm.
An indicative signal for what we presume to be the minor diastereoisomer can be found at δ = 3.26–3.19 (1H, m) ppm in the crude reaction mixture.
^{13}C NMR ($CDCl_3$, 101 MHz) δ = 214.2, 140.9, 135.5, 133.2 (2C), 128.2 (2C), 60.3, 41.8, 35.2, 34.5, 32.7, 29.7, 28.1, 25.2, 18.1 (2C), 16.9, 16.2 (2C), 15.6 ppm.
HRMS (ESI$^+$) Found $[M+Na]^+$ = 355.2067; $C_{21}H_{32}NaOS$ requires 355.2072.
The relative stereochemistry was assigned by analogy to **4a**.

^{1}H NMR ($CDCl_3$, 400 MHz) crude reaction mixture

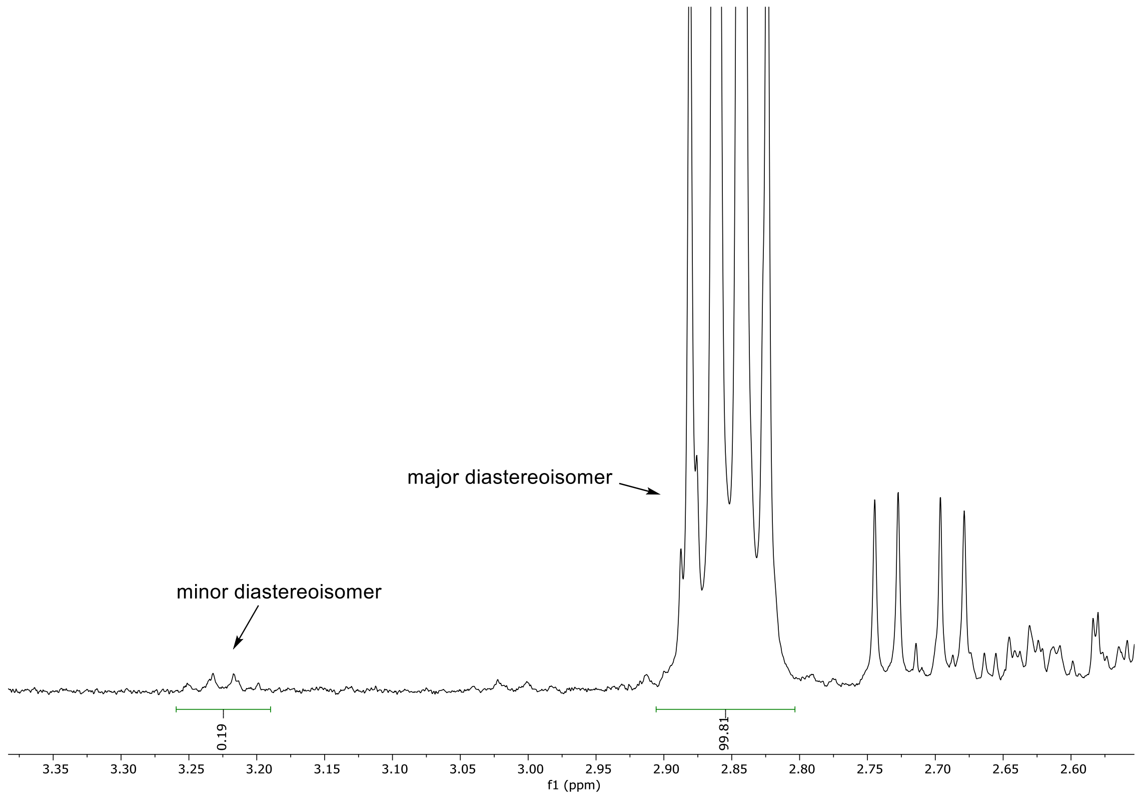

***trans*-(2,3,4,5,6-Pentamethylphenyl)(2-(2-(pyridin-2-yl)ethyl)cyclopentyl)methanone (4i)**

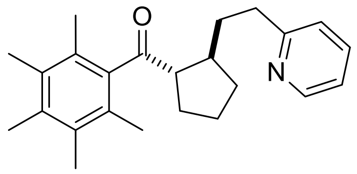

4i
$C_{24}H_{31}NO$
349.52 g mol^{-1}

Ketone **1** (114 mg, 0.600 mmol, 1.00 equiv), [Ir(COD)Cl]$_2$ (4.03 mg, 1 mol%), cataCXium® A (17.2 mg, 8 mol%), KO*t*-Bu (67.3 mg, 0.600 mmol, 1.00 equiv), 1-cyclopropyl-3-(pyridin-2-yl)propan-1-ol (**2i**, 213 mg, 1.20 mmol, 2.00 equiv) and toluene (150 μL, 4 M) were subjected to **General Procedure A** at 125 °C. Purification by flash column chromatography (Pentane:Et_2O, 96:4) afforded ketone **4i** (111 mg, 0.318 mmol, 53%) as a brown solid.

m.p. = 79–80 °C.
IR (film) v_{max}/cm^{-1} 2923, 2866, 1683, 1590, 1567, 1473, 1433, 1383, 1301, 1263, 1183, 1133, 1070, 1051, 1006, 993, 838, 770, 751, 715, 690.
^{1}H NMR ($CDCl_3$, 400 MHz) δ = 8.49 (1H, ddd, *J* = 4.9, 1.8, 0.9 Hz), 7.54 (1H, td, *J* = 7.6, 1.9 Hz), 7.15–6.99 (2H, m), 2.92 (1H, dt, *J* = 8.7, 6.8 Hz), 2.85–2.65 (2H, m), 2.35 (1H, tdd, *J* =

11.9, 5.9, 3.6 Hz), 2.22 (3H, s), 2.16 (6H, s), 2.07 (6H, s), 2.05–1.76 (4H, m), 1.72–1.62 (3H, m), 1.39 (1H, dq, J = 12.5, 7.5 Hz) ppm.

^{13}C NMR ($CDCl_3$, 101 MHz) δ = 214.1, 162.2, 149.3, 140.9, 136.4, 135.4, 133.2 (2C), 128.0 (2C), 122.8, 121.0, 60.3, 41.8, 37.3, 36.1, 32.5, 29.5, 25.2, 18.1 (2C), 16.8, 16.2 (2C) ppm.

HRMS (ESI$^+$) Found $[M+H]^+$ = 350.2479; $C_{24}H_{32}NO$ requires 350.2484.

The relative stereochemistry was assigned by analogy to **4a**. Signals for the minor diastereoisomer were not found by ^{1}H NMR analysis of the crude reaction mixture.

(2-Methyl-5-phenylcyclopentyl)(2,3,4,5,6-pentamethylphenyl)methanone (4j and 4j')

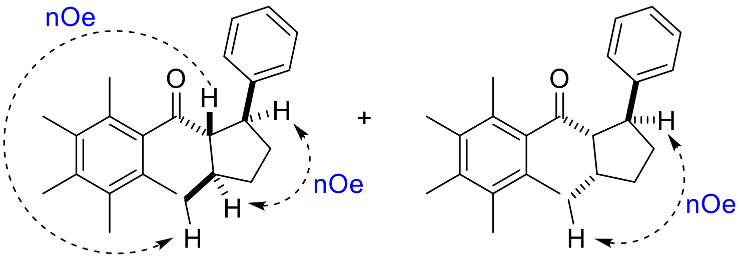

4j major
$C_{24}H_{30}O$
334.50 g mol^{-1}

4j' minor
$C_{24}H_{30}O$
334.50 g mol^{-1}

Ketone **1** (114 mg, 0.600 mmol, 1.00 equiv), $[Ir(COD)Cl]_2$ (4.03 mg, 1 mol%), cataCXium® A (17.2 mg, 8 mol%), KO*t*-Bu (67.3 mg, 0.600 mmol, 1.00 equiv), 1-(*trans*-2-phenylcyclopropyl)-ethan-1-ol (**2j**, 103 mg, 1.20 mmol, 2.00 equiv) and toluene (150 μL, 4 M) were subjected to **General Procedure A** at 125 °C. Purification by flash column chromatography (Pentane:Et_2O, 99.5:0.5→99:1) afforded a yellow oil (108 mg, 54%, 77:23 d.r.) that contained an inseparable mixture of ketones **4j** and **4j'**.

IR (film) ν_{max}/cm^{-1} 3082, 3061, 3027, 2950, 2869, 1686, 1602, 1573, 1493, 1453, 1381, 1338, 1303, 1269, 1167, 1126, 1092, 1070, 1029, 999, 950, 910, 823, 756, 700.

^{1}H NMR ($CDCl_3$, 400 MHz, major isomer, selected signals) δ = 7.21–7.14 (2H, m), 7.14–7.09 (3H, m), 3.60–3.54 (1H, m), 2.97–2.89 (1H, m), 2.69–2.60 (1H, m), 1.03 (3H, d, J = 6.7 Hz) ppm.

^{13}C NMR ($CDCl_3$, 101 MHz, major isomer) δ = 212.9, 145.2, 140.7, 135.4, 133.0 (2C, br. s), 128.2 (2C), 128.0 (2C, v. br. s), 127.6 (2C), 126.0, 69.7, 48.6, 38.3, 34.9, 34.5, 21.7, 17.8 (br. s), 17.8 (br. s), 16.8, 16.1 (2C, br. s) ppm.

^{1}H NMR ($CDCl_3$, 400 MHz, minor isomer, selected signals) δ = 7.21–7.14 (2H, m), 7.14–7.09 (1H, m), 7.08–7.04 (2H, m), 3.64–3.57 (1H, m), 3.33 (1H, dd, J = 9.9, 6.0 Hz), 2.69–2.59 (1H, m), 1.15 (3H, d, J = 7.0 Hz) ppm.

^{13}C NMR ($CDCl_3$, 101 MHz, minor isomer) δ = 212.6, 146.1, 141.0, 135.4, 133.0 (2C), 128.4, 128.4, 128.2 (2C), 127.8 (2C), 126.0, 67.0, 45.4, 38.0, 34.8, 32.8, 17.4 (2C, br. s), 16.8, 16.2, 16.1 (2C) ppm.
HRMS (ESI$^+$) Found $[M+H]^+$ = 335.2370; $C_{24}H_{31}O$ requires 335.2370.

***trans*-(1-Methyloctahydro-1H-inden-2-yl)(2,3,4,5,6-pentamethylphenyl)methanone (4l)**

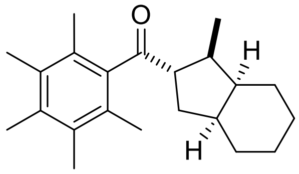

4l
$C_{22}H_{32}O$
312.50 g mol^{-1}

Ketone **1** (114 mg, 0.600 mmol, 1.00 equiv), $[Ir(COD)Cl]_2$ (4.03 mg, 1 mol%), cataCXium® A (17.2 mg, 8 mol%), KO*t*-Bu (67.3 mg, 0.600 mmol, 1.00 equiv), alcohol **2l** (168 mg, 1.20 mmol, 2.00 equiv) and toluene (150 μL, 4 M) were subjected to **General Procedure A** at 125 °C. Purification by flash column chromatography (Pentane:Et_2O, 99:1) afforded ketone **4l** (120 mg, 0.385 mmol, 64%) as a colourless solid.

m.p. = 118–120 °C.
IR (film) ν_{max}/cm^{-1} 2921, 2872, 2853, 1687, 1573, 1448, 1379, 1361, 1340, 1309, 1258, 1221, 1174, 1140, 1130, 1122, 1104, 1070, 1020, 998, 958, 926, 912, 870, 853, 814, 706.
^{1}H NMR ($CDCl_3$, 500 MHz) δ = 2.99 (1H, ddd, *J* = 10.8, 9.7, 5.0 Hz), 2.41 (1H, dquint., *J* = 9.7, 6.8 Hz), 2.30–2.19 (4H, m), 2.18 (6H, s), 2.12 (6H, s), 1.98–1.89 (2H, m), 1.81 (1H, dq, *J* = 11.1, 5.6 Hz), 1.69 (1H, dquint.d, *J* = 12.9, 3.4, 1.3 Hz), 1.59–1.51 (3H, m), 1.46 (2H, m), 1.30 (1H, m), 1.11 (1H, qt, *J* = 13.0, 3.2 Hz), 0.96–0.84 (3H, m) ppm.
^{13}C NMR ($CDCl_3$, 126 MHz) δ = 215.4, 141.4, 135.4, 133.2 (2C), 127.8 (2C, v. br. s), 58.5, 45.0, 42.3, 38.8, 30.4, 26.7, 25.5, 23.1, 20.9, 18.1 (2C, br. s), 16.9, 16.2 (2C), 15.3 ppm.
HRMS (ESI$^+$) Found $[M+H]^+$ = 313.2525; $C_{22}H_{33}O$ requires 313.2526.
The relative stereochemistry of the product was confirmed by single crystal X-ray diffraction. Signals for the minor diastereoisomer were not found by ^{1}H NMR analysis of the crude reaction mixture.

(Octahydro-1H-inden-1-yl)(2,3,4,5,6-pentamethylphenyl)methanone (4m)

nOe:

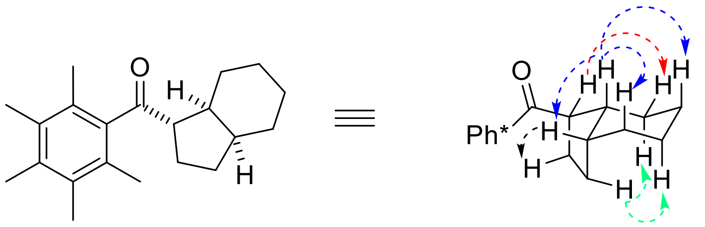

4m

$C_{21}H_{30}O$

298.47 g mol^{-1}

Ketone **1** (114 mg, 0.600 mmol, 1.00 equiv), $[Ir(COD)Cl]_2$ (4.03 mg, 1 mol%), cataCXium® A (17.2 mg, 8 mol%), KO*t*-Bu (67.3 mg, 0.600 mmol, 1.00 equiv), spirocyclic alcohol (**2m**, 151 mg, 1.20 mmol, 2.00 equiv) and toluene (150 µL, 4 M) were subjected to **General Procedure A** at 125 °C. Purification by flash column chromatography (Pentane:Et_2O, 99:1) afforded ketone **4m** (83.0 mg, 0.277 mmol, 46%) as a colourless solid.

m.p. = 86–88 °C.

IR (film) ν_{max}/cm^{-1} 2922, 2853, 1688, 1574, 1448, 1382, 1341, 1308, 1258, 1182, 1153, 1118, 1102, 1068, 1041, 1002, 944, 909, 894, 879, 853, 827, 804, 702.

^{1}H NMR ($CDCl_3$, 500 MHz) δ = 3.11 (1H, dt, *J* = 9.7, 6.1 Hz), 2.29 (1H, quint., *J* = 6.1 Hz), 2.23 (3H, s), 2.18 (6H, s), 2.11 (7H, m), 2.06–1.91 (2H, m), 1.73 (1H, ddt, *J* = 12.6, 9.5, 6.4 Hz), 1.61–1.44 (4H, m), 1.44–1.27 (5H, m) ppm.

^{13}C NMR ($CDCl_3$, 126 MHz) δ = 214.9, 141.2, 135.4, 133.2 (2C), 128.0 (2C, br. s), 57.0, 42.2, 39.6, 29.6, 27.9, 27.6, 26.7, 23.6, 23.3, 18.0 (2C, br. s), 16.8, 16.2 (2C) ppm.

HRMS (ESI$^+$) Found $[M+H]^+$ = 299.2369; $C_{21}H_{31}O$ requires 299.2369.

Signals for the minor diastereoisomer were not found by ^{1}H NMR analysis of the crude reaction mixture.

(2-Methyloctahydro-1H-inden-1-yl)(2,3,4,5,6-pentamethylphenyl)methanone (4n)

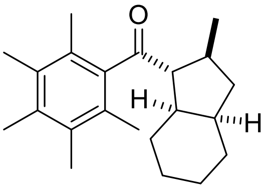

4n

$C_{22}H_{32}O$

312.50 g mol^{-1}

Ketone **1** (114 mg, 0.600 mmol, 1.00 equiv), $[Ir(COD)Cl]_2$ (4.03 mg, 1 mol%), cataCXium® A (17.2 mg, 8 mol%), KO*t*-Bu (67.3 mg, 0.600 mmol, 1.00 equiv), 1-(*exo*-bicyclo[4.1.0]heptan-7-

yl)ethan-1-ol (**2n**, 168 mg, 1.20 mmol, 2.00 equiv) and toluene (150 μL, 4 M) were subjected to **General Procedure A** at 125 °C. Purification by flash column chromatography (Pentane:Et_2O, 98:2) afforded ketone **4n** (101 mg, 0.323 mmol, 54%) as a colourless solid.

m.p. = 81–84 °C.
IR (film) ν_{max}/cm^{-1} 2922, 2853, 1447, 1372, 1339, 1301, 1275, 1187, 1132, 1105, 1056, 1016, 994, 960, 915, 886, 824, 798, 710, 677.
^{1}H NMR ($CDCl_3$, 400 MHz) δ = 2.59 (1H, dd, *J* = 7.1, 4.0 Hz), 2.43 (1H, m), 2.27 (1H, m), 2.23 (3H, s), 2.18 (6H, s), 2.16–2.12 (1H, m), 2.11 (6H, s), 1.81 (1H, m), 1.60–1.47 (4H, m), 1.44–1.34 (3H, m), 1.32–1.17 (2H, m), 1.01 (3H, d, *J* = 6.8 Hz) ppm.
^{13}C NMR ($CDCl_3$, 101 MHz) δ = 214.3, 141.3, 135.3, 133.1 (2C), 127.7 (2C, v. br. s), 67.3, 42.4, 39.1, 38.2, 36.3, 29.7, 27.6, 25.3, 22.7, 22.3, 18.1 (2C, br. s), 16.9, 16.2 (2C) ppm.
HRMS (ESI^+) Found $[M+H]^+$ = 313.2526; $C_{22}H_{33}O$ requires 313.2531.
The relative stereochemistry of the product was confirmed by single crystal X-ray diffraction. Signals for the minor diastereoisomer were not found by ^{1}H NMR analysis of the crude reaction mixture.

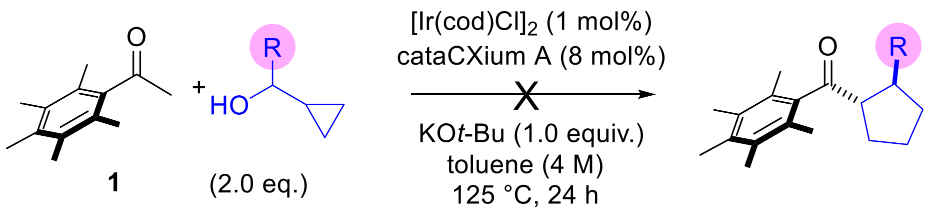

The following cyclopropyl alcohols were not compatible with the rearrangement reaction

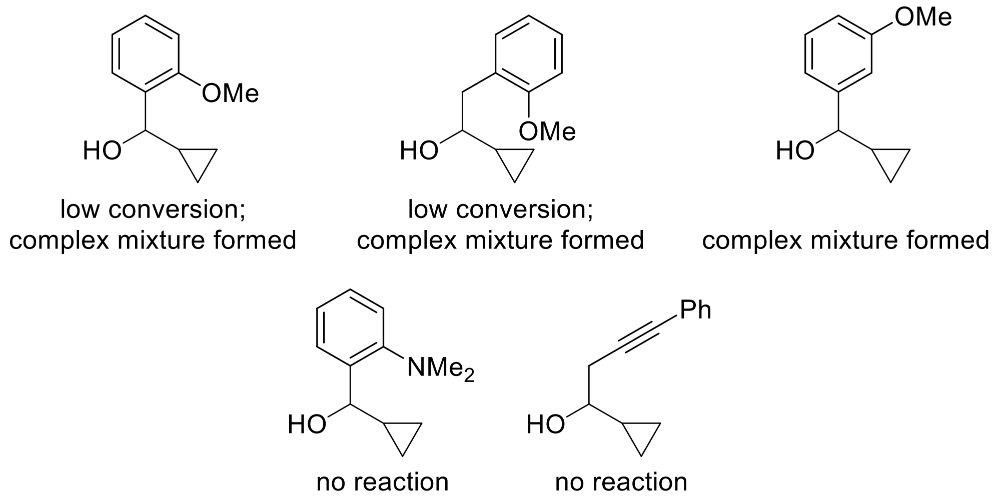

Scheme S2 Cyclopropyl alcohols not compatible with the rearrangement reaction

Note also that attempts to subject PhCOMe to the standard reaction conditions, using 1-cyclopropylethan-1-ol, gave rise to a complex reaction mixture.

***trans*-*S*-Benzyl 2-ethylcyclopentane-1-carbothioate (5a)**

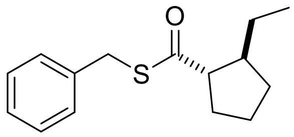

5a

$C_{15}H_{20}OS$

248.38 g mol^{-1}

Ketone **4a** (54.5 mg, 0.200 mmol, 1.00 equiv), Br_2 (20 μL, 64 mg, 0.40 mmol, 2.0 equiv) and DCM (1.0 mL, 0.2 M) were subjected to a modification of **General Procedure B** with BnSH (70 μL, 75 mg, 0.600 mmol, 3.00 equiv) as the nucleophile (caution: stench). The reaction was directly blown down with a gentle stream of Ar, and the residue purified by flash column chromatography (Pentane:Et_2O, 100:0→99.75:0.25→99.5:0.5) to afford thioester **5a** (36.7 mg, 0.148 mmol, 74%) as a colourless oil.

IR (film) ν_{max}/cm^{-1} 3087, 3063, 3030, 2958, 2927, 2872, 1686, 1603, 1585, 1495, 1454, 1412, 1379, 1361, 1290, 1241, 1180, 1098, 1071, 1029, 1002, 936, 914, 890, 816, 769, 699.

^{1}H NMR ($CDCl_3$, 500 MHz) δ = 7.29 (4H, s), 7.28–7.20 (1H, m), 4.13 (2H, q_{AB}, J_{AB} = 13.7 Hz, Δ_{AB} = 4.7 Hz), 2.61 (1H, q, J = 8.1 Hz), 2.12 (1H, quint.d, J = 8.2, 5.6 Hz), 2.00–1.90 (1H, m), 1.93 (1H, dtd, J = 12.6, 7.7, 5.2 Hz), 1.86 (1H, dq, J = 13.3, 7.5 Hz), 1.76–1.59 (2H, m), 1.55 (1H, dqd, J = 13.2, 7.5, 5.7 Hz), 1.29 (1H, dquint., J = 13.5, 7.7 Hz), 1.23 (1H, dq, J = 12.5, 8.3 Hz), 0.90 (3H, t, J = 7.4 Hz) ppm.
^{13}C NMR ($CDCl_3$, 126 MHz) δ = 202.6, 137.9, 128.9 (2C), 128.7 (2C), 127.3, 59.5, 46.6, 33.3, 32.4, 31.5, 28.1, 25.0, 12.8 ppm.
HRMS (ESI^+) Found $[M+H]^+$ = 249.1308; $C_{15}H_{21}OS$ requires 249.1308.

***trans*-2-Cyclobutylcyclopentane-1-carboxylic acid (5c)**

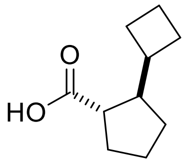

5c
$C_{10}H_{16}O_2$
168.24 g mol^{-1}

Ketone **4c** (59.7 mg, 0.200 mmol, 1.00 equiv), Br_2 (20 µL, 64 mg, 0.40 mmol, 2.0 equiv) and DCM (1.0 mL, 0.2 M) were subjected to **General Procedure B** with 2-mercaptobenzothiazole (100 mg, 0.600 mmol, 3.00 equiv) as the nucleophile. Purification by flash column chromatography (Pentane:Et_2O, 80:20→60:40) afforded carboxylic acid **5c** (30.5 mg, 0.181 mmol, 91%) as a colourless oil.

IR (film) ν_{max}/cm^{-1} 2954, 2867, 2673, 1699, 1420, 1342, 1305, 1291, 1230, 1162, 940, 696.
^{1}H NMR ($CDCl_3$, 500 MHz) δ = 11.51 (1H, v. br. s), 2.33 (1H, dt, J = 8.4, 7.0 Hz), 2.31–2.18 (2H, m), 2.04–1.92 (2H, m), 1.95–1.83 (2H, m), 1.86–1.76 (2H, m), 1.79–1.70 (2H, m), 1.72–1.63 (2H, m), 1.66–1.55 (1H, m), 1.25 (1H, dq, J = 12.3, 7.3 Hz) ppm.
^{13}C NMR ($CDCl_3$, 126 MHz) δ = 183.9, 50.1, 48.2, 40.6, 30.9, 30.0, 26.9, 26.8, 25.2, 18.1 ppm.
HRMS (ESI^-) Found $[M–H]^-$ = 167.1077; $C_{10}H_{15}O_2$ requires 167.1078.
Note: The desired thioester was not observed.

trans-2-Phenethylcyclopentane-1-carboxylic acid (5d)

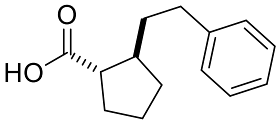

5d

$C_{14}H_{18}O_2$

218.30 g mol^{-1}

Ketone **4d** (69.7 mg, 0.200 mmol, 1.00 equiv), Br_2 (20 µL, 64 mg, 0.40 mmol, 2.0 equiv) and DCM (1.0 mL, 0.2 M) were subjected to a modification of **General Procedure B** without a nucleophile added. After 2 h, the reaction was directly quenched with sat. aq. Na_2SO_3 (2 mL) and diluted with DCM (2 mL). The layers were separated, the organic layer extracted with 6 M NaOH (2 × 2 mL). The combined aqueous layer were extracted with Et_2O (2 × 2 mL) and the combined organics discarded. The aqueous extracts were acidified with 6 M HCl (5–6 mL), saturated with NaCl, and extracted with EtOAc (5 × 5 mL). The combined organics were then dried (Na_2SO_4) and concentrated *in vacuo* to directly afford carboxylic acid **5d** (37.9 mg, 0.174 mmol, 87%) as a colourless oil.

IR (film) v_{max}/cm^{-1} 3085, 3062, 3026, 2937, 2858, 2656, 1698, 1603, 1496, 1453, 1421, 1292, 1231, 1031, 942, 748, 698.

^{1}H NMR ($CDCl_3$, 500 MHz) δ = 9.71 (1H, v. br. s), 7.33 –7.22 (2H, m), 7.23–7.10 (3H, m), 2.66 (1H, ddd, *J* = 13.2, 10.7, 5.5 Hz), 2.63 (1H, ddd, *J* = 13.7, 10.3, 5.9 Hz), 2.44 (1H, q, *J* = 8.1 Hz), 2.21 (1H, sext., *J* = 8.2, 5.8 Hz), 2.05–1.93 (2H, m), 1.96–1.86 (2H, m), 1.78–1.66 (2H, m), 1.62 (1H, dtd, *J* = 14.1, 9.3, 5.6 Hz), 1.31 (1H, dq, *J* = 13.0, 8.7 Hz) ppm.

^{13}C NMR ($CDCl_3$, 126 MHz) δ = 183.3, 142.5, 128.45 (2C), 128.42 (2C), 125.9, 50.4, 44.4, 37.4, 34.7, 32.8, 30.4, 25.0 ppm.

HRMS (ESI$^-$) Found [M–H]$^-$ = 217.1231; $C_{14}H_{17}O_2$ requires 217.1234.

Octahydro-1*H*-cyclopenta[*c*]oxepin-1-one (5f)

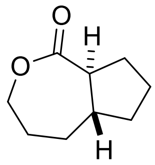

5f

$C_9H_{14}O_2$

154.21 g mol^{-1}

Ketone **4f** (98.2 mg, 0.250 mmol, 1.00 equiv), Br_2 (26 µL, 80 mg, 0.50 mmol, 2.0 equiv) and DCM (1.3 mL, 0.2 M) were subjected to a modification of **General Procedure B** with no nucleophile being added and the reaction being stirred for 2 h. Purification by flash column

chromatography (Pentane:Et_2O, 100:0→80:20) afforded lactone **5f** (34.2 mg, 0.222 mmol, 89%) as a colourless oil.

IR (film) ν_{max}/cm^{-1} 2928, 2870, 1730, 1473, 1451, 1431, 1393, 1364, 1334, 1297, 1268, 1173, 1147, 1108, 1079, 1033, 1009, 966, 919, 894, 837, 680.
^{1}H NMR ($CDCl_3$, 500 MHz) δ = 4.32–4.20 (2H, m), 2.71 (1H, dt, *J* = 10.9, 8.5 Hz), 2.22 (1H, dq, *J* = 13.5, 8.0 Hz), 2.12 (1H, dqd, *J* = 13.6, 3.4, 1.1 Hz), 1.97 (1H, dq, *J* = 12.5, 7.6, 5.8 Hz), 1.91 (1H, dqd, *J* = 15.7, 4.3, 1.3 Hz), 1.86 (1H, dt, *J* = 13.7, 8.5, 7.2 Hz), 1.81 (1H, ddd, *J* = 11.2, 10.2, 7.5, 3.5 Hz), 1.75 (1H, dddt, *J* = 15.8, 13.3, 9.9, 3.5 Hz), 1.65 (2H, ddq_{AB}, *J* = 6.9, 5.8 Hz, J_{AB} = 8.2 Hz, Δ_{AB} = 3.8 Hz), 1.37 (1H, tdd, *J* = 13.3, 11.8, 4.0 Hz), 1.28 (1H, ddt, *J* = 12.6, 9.9, 8.8 Hz) ppm.
^{13}C NMR ($CDCl_3$, 126 MHz) δ = 176.8, 68.9, 48.1, 43.5, 35.0, 34.8, 28.9, 28.4, 23.9 ppm.
HRMS (ESI$^+$) Found $[M+Na]^+$ = 177.0888; $C_9H_{14}O_2Na$ requires 177.0886.
The spectroscopic data is in good agreement with previous reports.[18]

***trans*-1-Benzylpiperidin-4-yl *trans*-1-methyloctahydro-1H-indene-2-carboxylate (5l)**

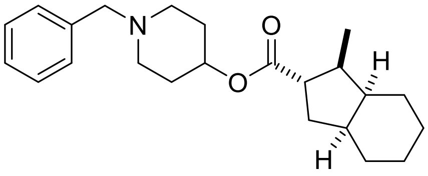

5l
$C_{23}H_{33}NO_2$
355.52 g mol^{-1}

Ketone **4l** (62.5 mg, 0.200 mmol, 1.00 equiv), Br_2 (20 μL, 64 mg, 0.40 mmol, 2.0 equiv) and DCM (1.0 mL, 0.2 M) were subjected to **General Procedure B** with *N*-benzyl-4-hydroxypiperidine (115 mg, 0.600 mmol, 3.00 equiv) as the nucleophile. Purification by flash column chromatography (Pentane:Et_2O, 85:15→70:30) afforded ester **5l** (55.1 mg, 0.155 mmol, 94%) as a colourless oil.

IR (film) ν_{max}/cm^{-1} 3085, 3062, 3028, 2922, 2873, 2853, 2804, 2764, 1726, 1494, 1453, 1376, 1358, 1340, 1312, 1266, 1248, 1223, 1200, 1181, 1166, 1143, 1109, 1073, 1040, 1009, 978, 950, 927, 904, 859, 807, 794, 737, 698.

^{1}H NMR ($CDCl_3$, 500 MHz) δ = 7.35–7.28 (4H, m), 7.29–7.22 (1H, m), 4.81 (1H, tt, J = 8.0, 4.0 Hz), 3.51 (2H, s), 2.67 (2H, br. dt, J = 10.6, 5.0 Hz), 2.48 (1H, td, J = 10.6, 4.9 Hz), 2.30 (2H, br. t, J = 10.4 Hz), 2.30–2.17 (1H, m), 2.21 (1H, dquint.d, J = 9.8, 6.8, 5.8 Hz), 1.91 (1H, dt, J = 13.6, 11.3 Hz), 1.94–1.86 (2H, m), 1.83 (1H, ddd, J = 13.7, 9.4, 5.0 Hz), 1.76 (1H, dq, J = 13.0, 5.5 Hz), 1.75–1.65 (3H, m), 1.62–1.52 (2H, m), 1.53–1.43 (2H, m), 1.30 (1H, qt, J = 12.9, 5.0, 3.3 Hz), 1.10 (1H, qt, J = 12.9, 3.3 Hz), 0.99 (3H, d, J = 6.9 Hz), 0.87 (1H, qd, J = 13.0, 3.5 Hz) ppm.

^{13}C NMR ($CDCl_3$, 126 MHz) δ = 177.0, 138.5, 129.2 (2C), 128.3 (2C), 127.1 (2C), 69.8 (v. br. s), 63.1, 50.8 (2C), 48.9, 44.5, 43.9, 38.7, 30.96 (br. s), 30.91 (br. s), 30.78, 26.7, 25.4, 22.8, 20.9, 14.7 ppm.

^{15}N NMR ($CDCl_3$, 50.7 MHz) δ = 51.0 ppm.

HRMS (ESI^{+}) Found $[M+H]^{+}$ = 356.2582; $C_{23}H_{34}NO_2$ requires 356.2584.

(4-Benzylpiperazin-1-yl)(octahydro-1H-inden-1-yl)methanone (5m)

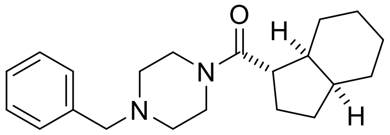

5m

$C_{21}H_{30}N_2O$

326.48 g mol^{-1}

Ketone **4m** (40.0 mg, 0.134 mmol, 1.00 equiv), Br_2 (14 µL, 44 mg, 0.28 mmol, 2.0 equiv) and DCM (0.70 mL, 0.2 M) were subjected to **General Procedure B** with *N*-benzylpiperazine (70 µL, 71 mg, 0.40 mmol, 3.00 equiv) as the nucleophile. Purification by flash column chromatography (Pentane:EtOAc, 60:40) afforded amide **5m** (22.6 mg, 69.2 µmol, 52%) as a colourless oil.

IR (film) ν_{max}/cm^{-1} 3085, 3061, 3027, 3003, 2921, 2852, 2807, 2766, 1638, 1494, 1432, 1366, 1348, 1298, 1275, 1238, 1218, 1146, 1103, 1074, 1038, 1001, 964, 909, 891, 849, 800, 740, 699.

^{1}H NMR ($CDCl_3$, 500 MHz) δ = 7.36–7.29 (4H, m), 7.31–7.23 (1H, m), 3.68 (2H, app. ddq$_{AB}$, J = 3.6, 5.9 Hz, J_{AB} = 13.0 Hz, Δ_{AB} = 34.7 Hz), 3.52 (2H, s), 3.50 (2H, t, J = 5.0 Hz), 2.83 (1H, dt, J = 10.0, 6.2 Hz), 2.47–2.36 (4H, m), 2.30 (1H, tt, J = 6.2, 5.8 Hz), 2.10–2.01 (1H, app. sext., J = 6.0 Hz), 2.02–1.90 (1H, m), 1.86–1.74 (1H, m), 1.84–1.70 (1H, m), 1.59–1.48 (3H, m), 1.52–1.43 (1H, m, C10H), 1.45–1.37 (1H, m), 1.40–1.33 (2H, m), 1.32–1.27 (1H, m), 1.27–1.21 (1H, m) ppm.

^{13}C NMR ($CDCl_3$, 126 MHz) δ = 174.9, 137.8, 129.3 (2C), 128.4 (2C), 127.4, 63.0, 53.5, 53.1, 45.7, 43.1, 43.0, 42.0, 39.1, 29.8, 28.0, 27.9, 27.0, 23.9, 23.3 ppm.

^{15}N NMR ($CDCl_3$, 50.7 MHz) δ = 49.9, 116.4 ppm.

HRMS (ESI+) Found $[M+H]^+$ = 327.2429; $C_{21}H_{31}NO_2$ requires 327.2431.

Control Experiments

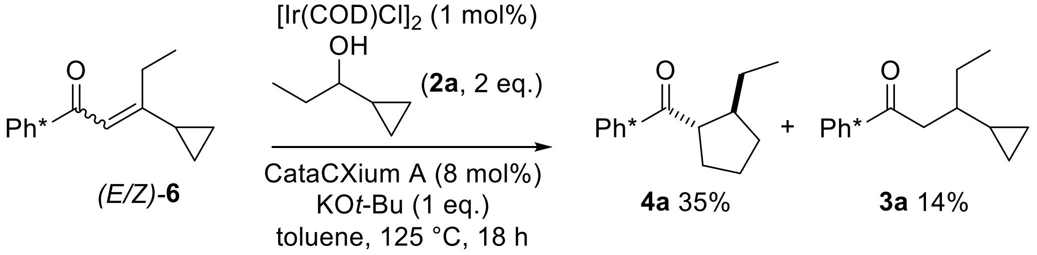

To a 2–5 mL microwave vial equipped with a stirrer bar was added enone (*E/Z*)-**6** (66 mg, 0.24 mmol, 1.0 equiv), 1-cyclopropylpropan-1-ol (**2a**, 49 mg, 0.49 mmol, 2.0 equiv), $[Ir(COD)Cl]_2$ (1.6 mg, 2.4 µmol, 1.0 mol%), cataCXium® A (7.0 mg, 20 µmol, 8.0 mol%), KO*t*-Bu (27 mg, 0.24 mmol, 1.0 equiv) and toluene (0.1 mL) sequentially in the open atmosphere. The vial was sealed with a microwave vial cap, then evacuated and backfilled with Argon. The vial was then heated to 125 °C in a preheated oil bath for 18 h. The reaction mixture was cooled to RT, diluted with Et_2O and washed with sat. aq. NH_4Cl. The aqueous layer was extracted with Et_2O (3x) and the combined organic layers were concentrated *in vacuo*. Purification by flash column chromatography (Pentane:Et_2O, 98:2) afforded ketones **4a** (23 mg, 85 µmol, 35%) and **3a** (9.0 mg, 34 µmol, 14%) as white solids.

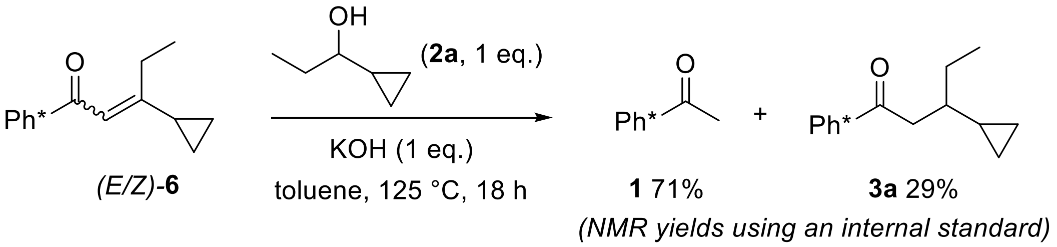

To a 2–5 mL microwave vial equipped with a stirrer bar was added enone (*E/Z*)-**6** (50.0 mg, 185 µmol, 1.0 equiv), 1-cyclopropylpropan-1-ol (**2a**, 18.5 mg, 185 µmol, 1.0 equiv), KOH (10.4 mg, 185 µmol, 1.0 equiv) and toluene (0.1 mL) sequentially in the open atmosphere. The vial was sealed with a microwave vial cap, then evacuated and backfilled with Argon. The vial was then heated to 125 °C in a preheated oil bath for 18 h. The reaction mixture was cooled to RT and concentrated *in vacuo*. ^{1}H-NMR analysis of the residue with 1,1,2,2-tetrachloroethane (0.5 equiv) as the internal standard showed ketone **1** (71%) along with ketone **2a** (29%).

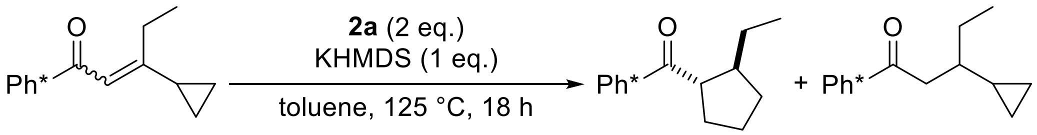

(E/Z)-**6** **4a** 34% **3a** 28%

To a 2–5 mL microwave vial equipped with a stirrer bar was added enone (*E/Z*)-**6** (20 mg, 74 µmol, 1.0 equiv) and 1-cyclopropylpropan-1-ol (**2a**, 15 mg, 0.15 mmol, 2.0 equiv) sequentially in the open atmosphere. The vial was sealed with a microwave vial cap, then evacuated and backfilled with Argon and a solution of KHMDS (0.5 M in toluene, 148 µL, 74.0 µmol, 1.00 equiv) was added by syringe. The vial was then heated to 125 °C in a preheated oil bath for 18 h. The reaction mixture was cooled to RT, diluted with Et_2O and washed with sat. aq. NH_4Cl. The aqueous layer was extracted with Et_2O (3x) and the combined organic layers were concentrated *in vacuo*. Purification by flash column chromatography (Pentane:Et_2O, 98:2) afforded ketones **4a** (6.9 mg, 25 µmol, 34%) and **3a** (5.6 mg, 21 µmol, 28%) as a white solids.

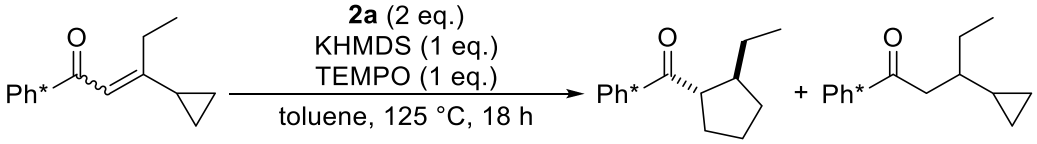

(E/Z)-**6** **4a** - **3a** 45%

To a 2–5 mL microwave vial equipped with a stirrer bar was added enone (*E/Z*)-**6** (20 mg, 74 µmol, 1.0 equiv), 1-cyclopropylpropan-1-ol (**2a**, 15 mg, 0.15 mmol, 2.0 equiv) and TEMPO (11.5 mg, 74.0 µmol, 1.00 equiv) sequentially in the open atmosphere. The vial was sealed with a microwave vial cap, then evacuated and backfilled with Argon and a solution of KHMDS (0.5 M in toluene, 148 µL, 74.0 µmol, 1.00 equiv) was added by syringe. The vial was then heated to 125 °C in a preheated oil bath for 18 h. The reaction mixture was cooled to RT, diluted with Et_2O and washed with sat. aq. NH_4Cl. The aqueous layer was extracted with Et_2O (3x) and the combined organic layers were concentrated *in vacuo*. Purification by flash column chromatography (Pentane:Et_2O, 98:2) afforded ketone **3a** (9.0 mg, 33 µmol, 45%) as a white solid.

m.p. = 34–37 °C.

IR (film) ν_{max}/cm^{-1} 2959, 2929, 2361, 1460, 1400, 1305, 1267, 1116, 1016, 962, 820, 690.

^{1}H NMR ($CDCl_3$, 400 MHz) δ = 2.83 (1H, dd, *J* = 19.3, 4.9 Hz), 2.72 (1H, dd, *J* = 19.4, 6.8 Hz), 2.23 (3H, s), 2.18 (6H, s), 2.12 (6H, s), 1.74–1.59 (1H, m), 1.56–1.45 (1H, m), 1.41–1.28 (1H, m), 1.00 (3H, t, *J* = 7.4 Hz), 0.63 (1H, dtt, *J* = 9.8, 8.0, 5.0 Hz), 0.52 (1H, dddd, *J* = 9.1, 8.1, 5.4, 3.9 Hz), 0.38 (1H, dddd, *J* = 9.2, 8.0, 5.2, 3.8 Hz), 0.28–0.20 (1H, m), 0.20–0.13 (1H, m) ppm.
^{13}C NMR ($CDCl_3$, 101 MHz) δ = 211.3, 141.1, 135.3, 133.2 (2C), 127.4 (2C), 51.0, 39.5, 28.0, 17.1 (2C), 16.8, 16.3, 16.1 (2C), 11.6, 5.4, 3.4 ppm.
HRMS (ESI^+) Found $[M+H]^+$ = 273.2213; $C_{19}H_{29}O$ requires 273.2213.

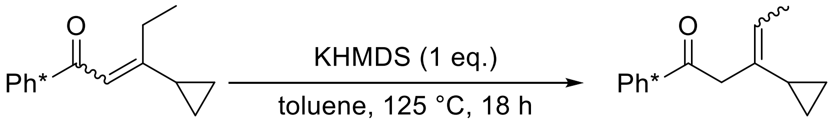

(E/Z)-**6** (*E*/*Z*)-**8**

To a 2–5 mL microwave vial equipped with a stirrer bar was added enone (*E/Z*)-**6** (20 mg, 74 µmol, 1.0 equiv), the vial was sealed with a microwave vial cap, then evacuated and backfilled with Argon and a solution of KHMDS (0.5 M in toluene, 148 µL, 74.0 µmol, 1.00 equiv) was added by syringe. The vial was then heated to 125 °C in a preheated oil bath for 18 h. The reaction mixture was cooled to RT, diluted with Et_2O and filtered over a plug consisting of Na_2SO_4/SiO_2/Celite. The solvent was removed under reduced pressure. ^{1}H NMR of residue revealed that it consists mostly of (*E/Z*)-**8** (16 mg, 80%).

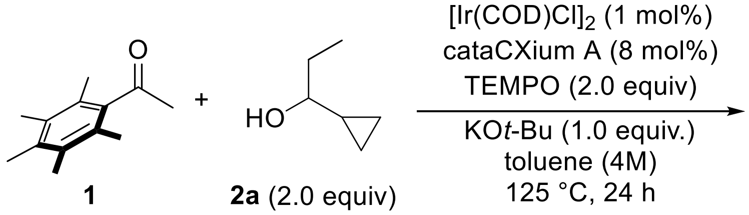

1 **2a** (2.0 equiv)

Ketone **1** (57 mg, 0.30 mmol, 1.0 equiv), $[Ir(COD)Cl]_2$ (2 mg, 1 mol%), cataCXium® A (8.6 mg, 8 mol%), KO*t*-Bu (33.7 mg, 0.30 µmol, 1.0 equiv), 1-cyclopropylpropan-1-ol (**2a**, 60 mg, 0.60 mmol, 2.0 equiv), TEMPO (94 mg, 0.60 mmol, 2.0 equiv) and toluene (4 M) were subjected to **General Procedure A** at 125 °C. Purification by flash column chromatography (Pentane:Et_2O, 98:2) afforded a mixture containing alkylated product **3a** (6%) and rearranged product **4a** (6%).

1-Cyclopropylpropan-1-ol (2a)
^{1}H NMR ($CDCl_3$, 400 MHz)

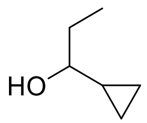

2a

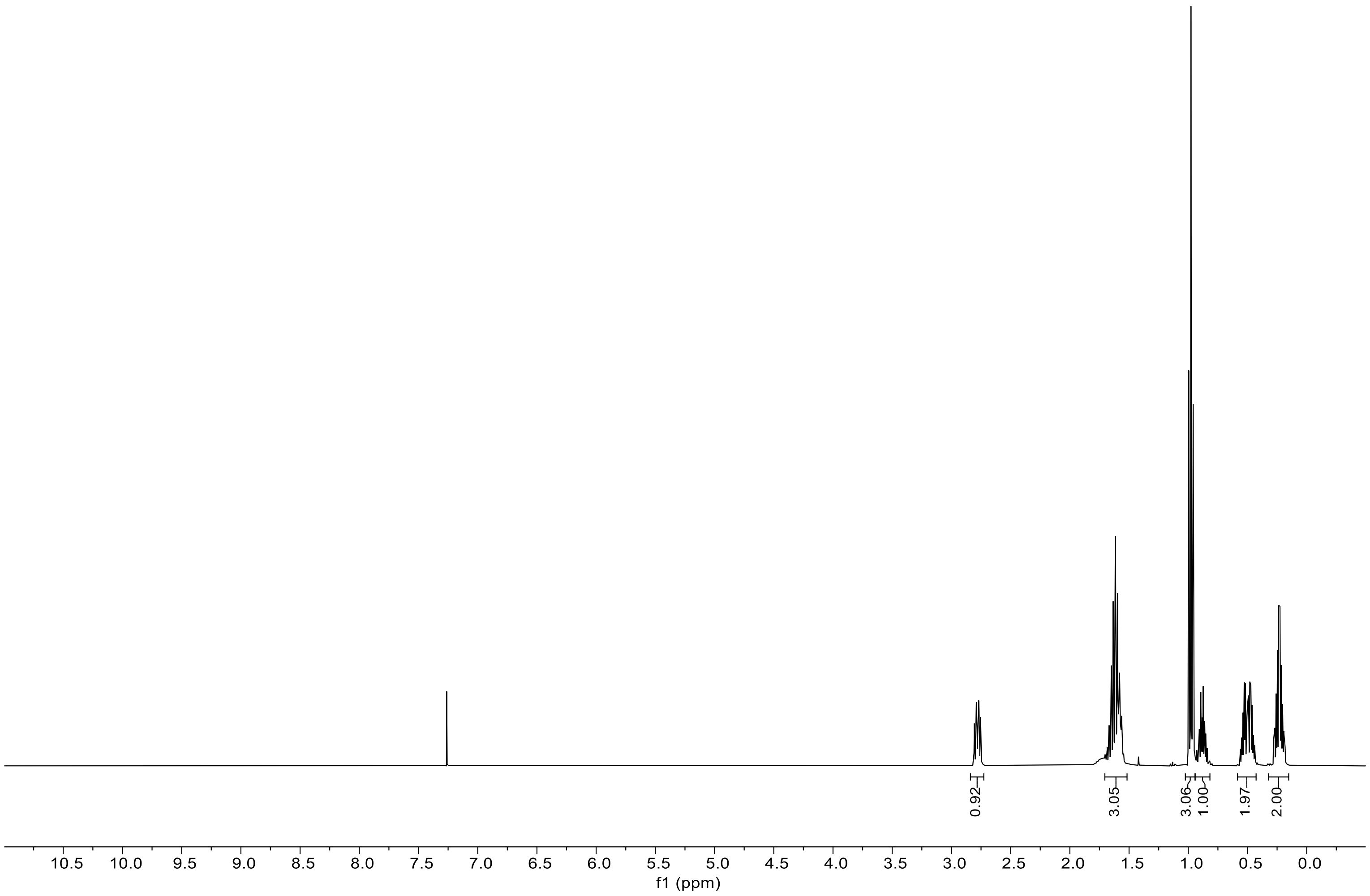

^{13}C NMR ($CDCl_3$, 101 MHz)

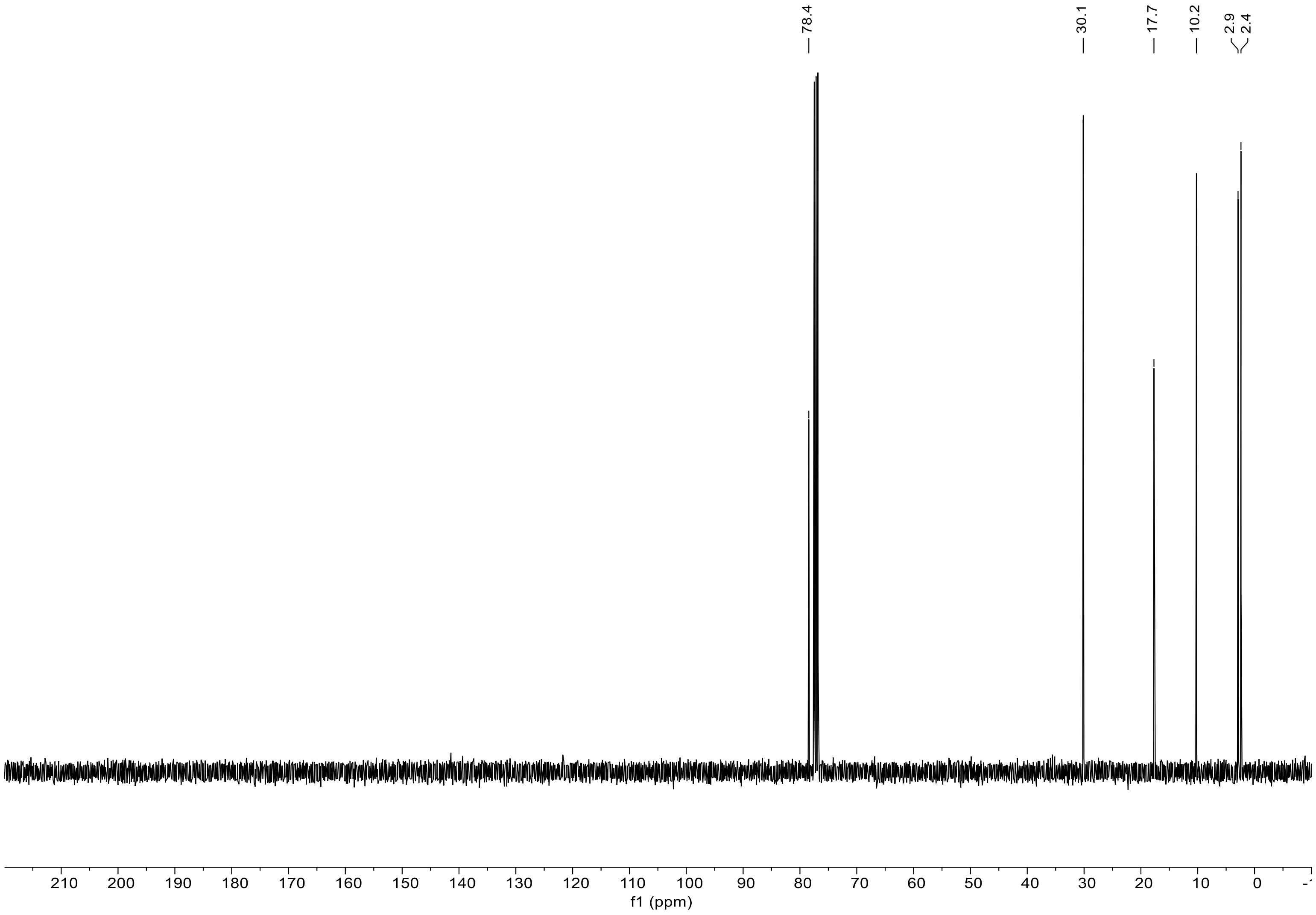

Cyclobutyl(cyclopropyl)methanol (2c)
¹H NMR (CDCl$_3$, 400 MHz)

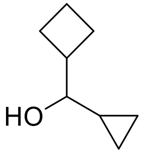

2c

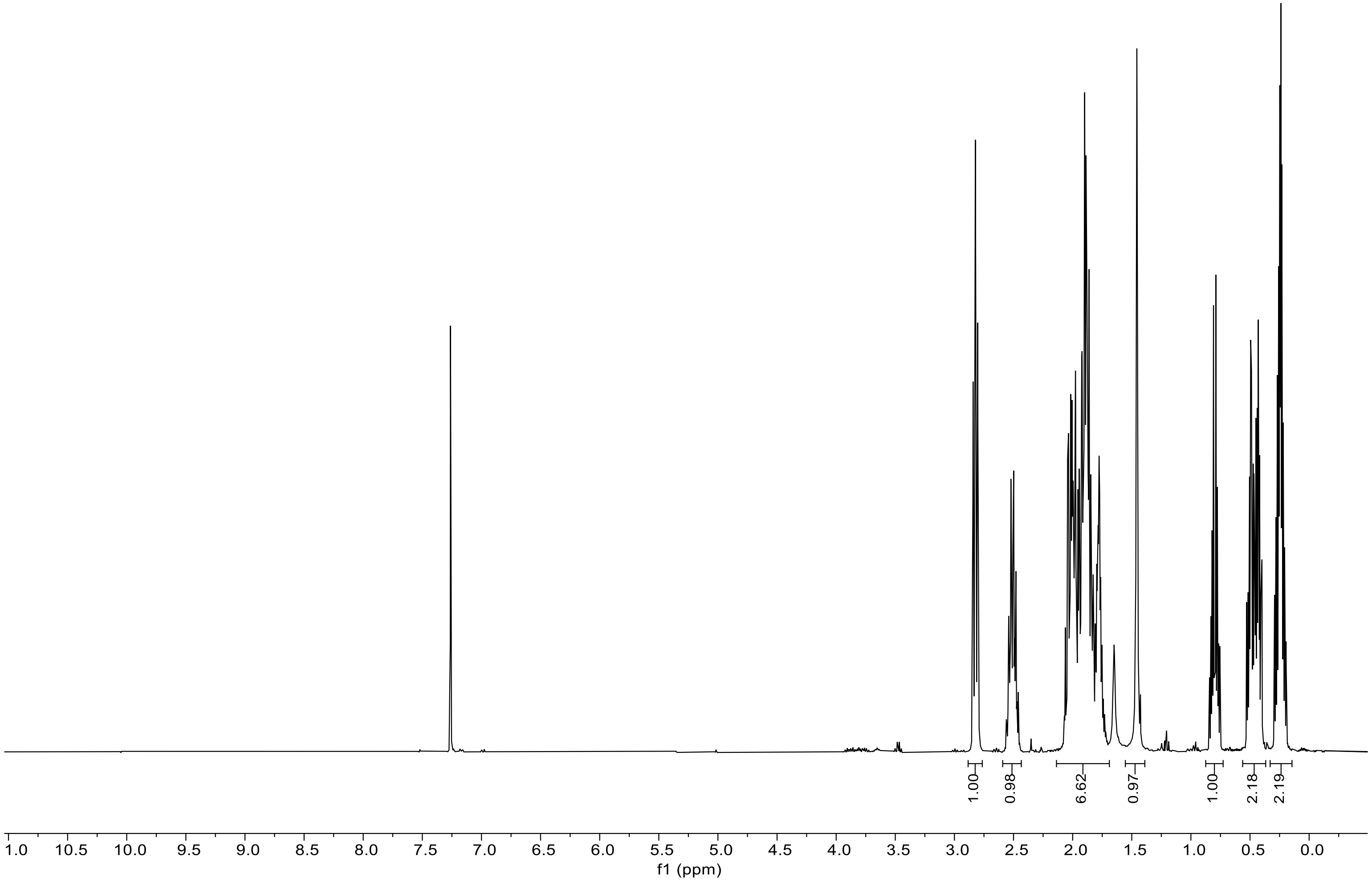

^{13}C NMR ($CDCl_3$, 101 MHz)

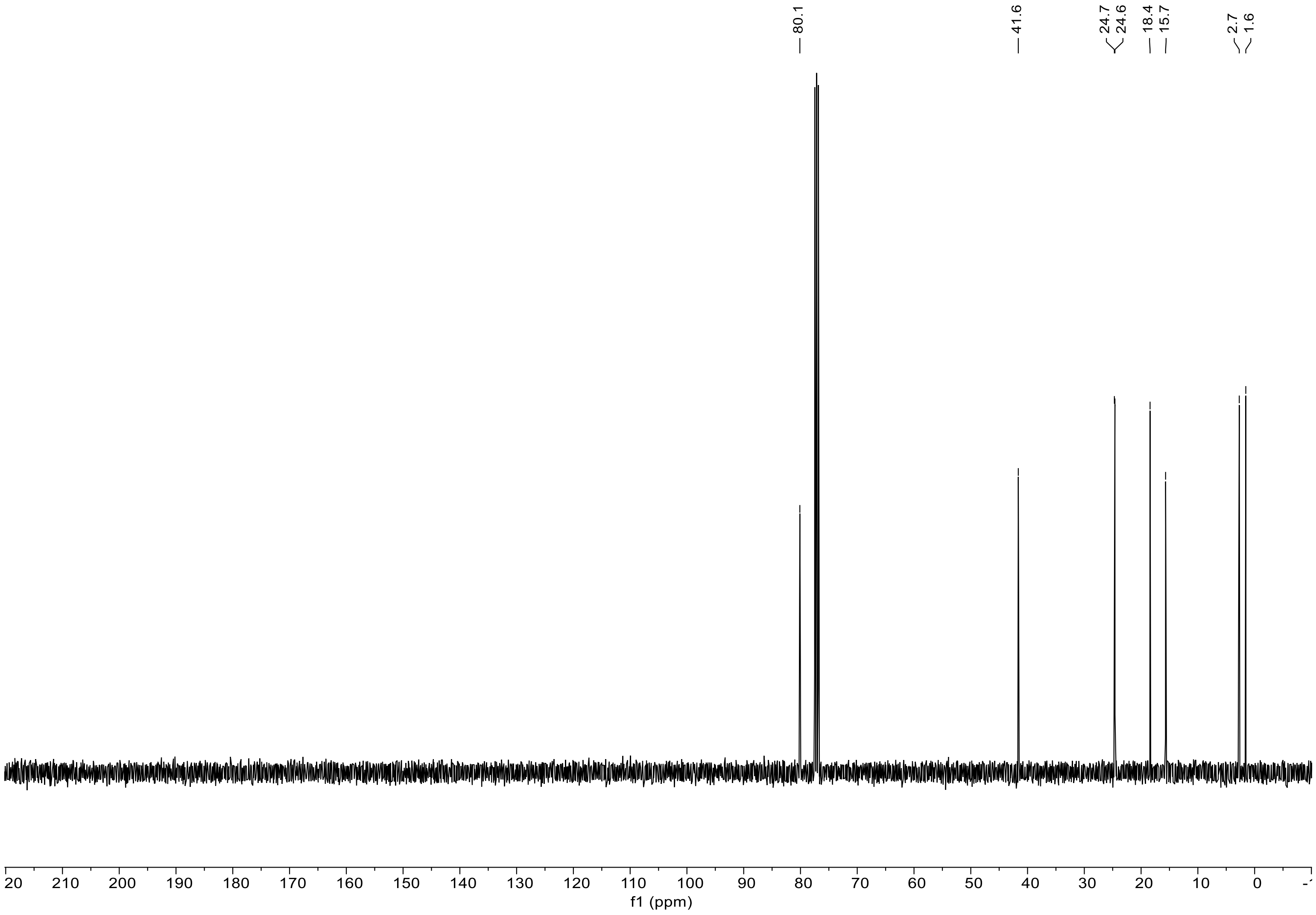

4-(Benzyloxy)-1-cyclopropylbutan-1-ol (2f)

^{1}H NMR ($CDCl_3$, 400 MHz)

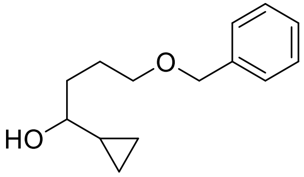

2f

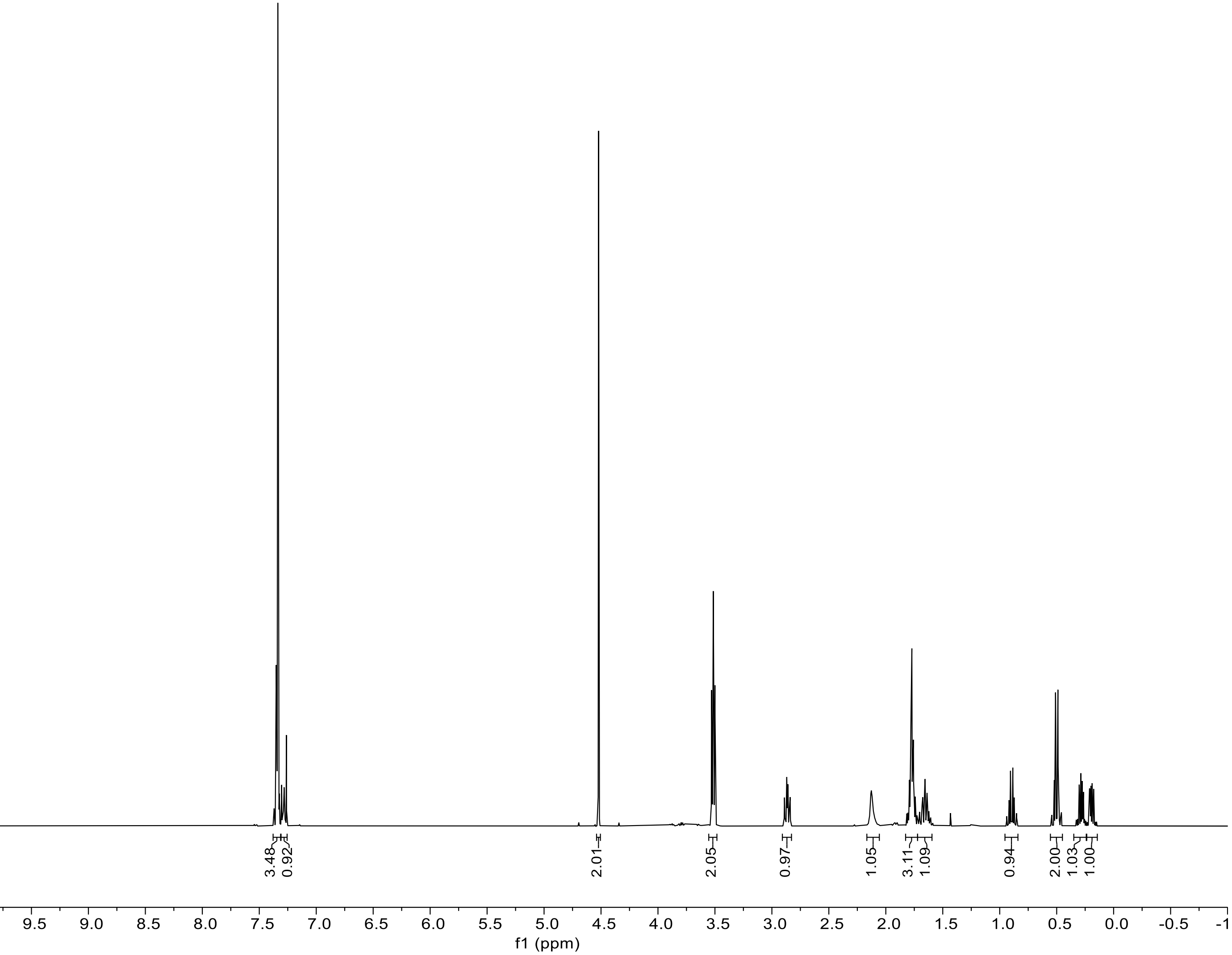

^{13}C NMR ($CDCl_3$, 101 MHz)

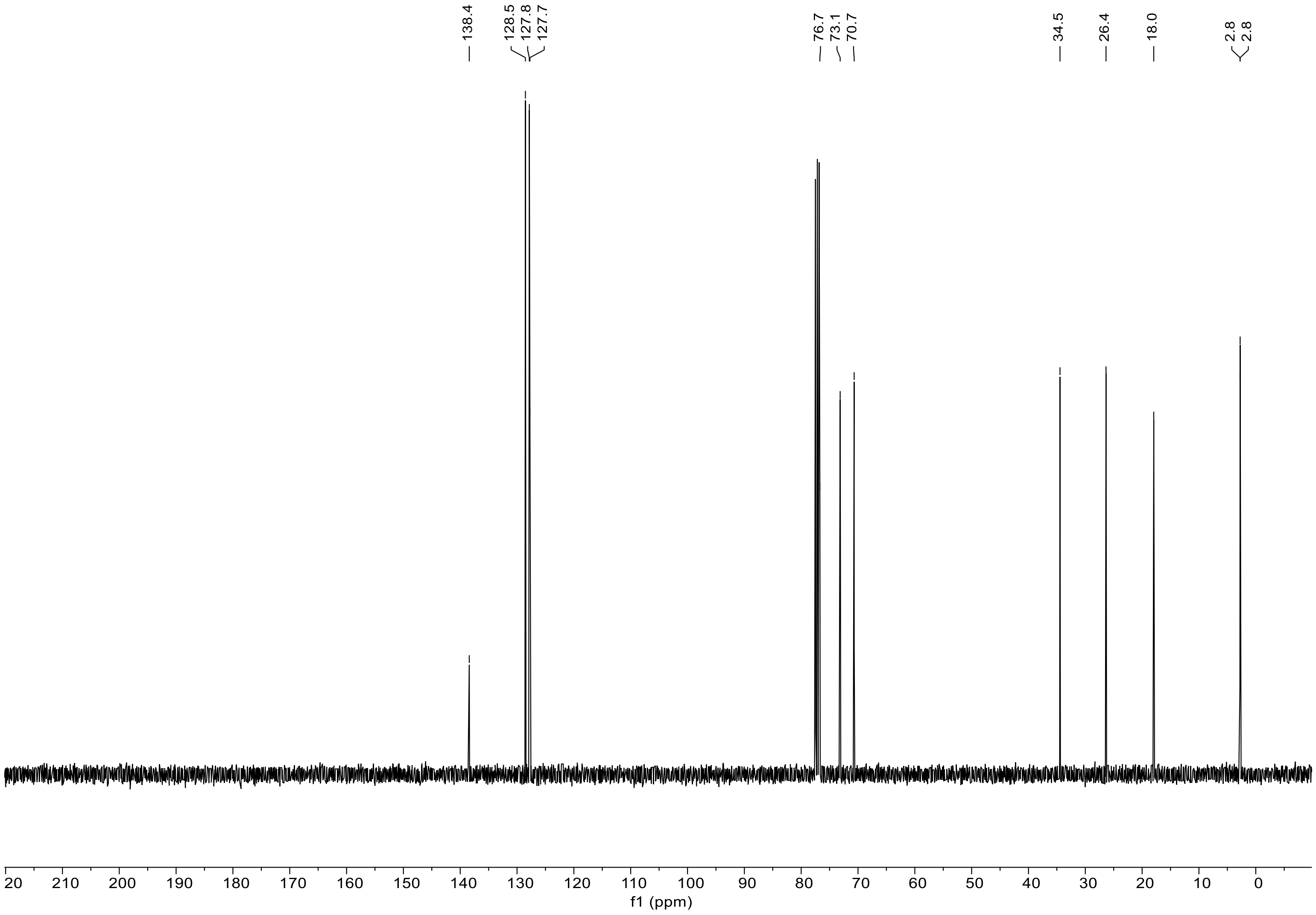

1-Cyclopropyl-4-(dibenzylamino)butan-1-ol (2g)
^{1}H NMR ($CDCl_3$, 400 MHz)

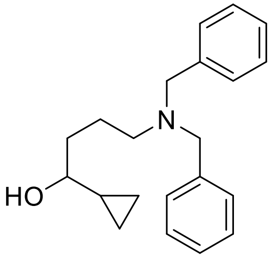

2g

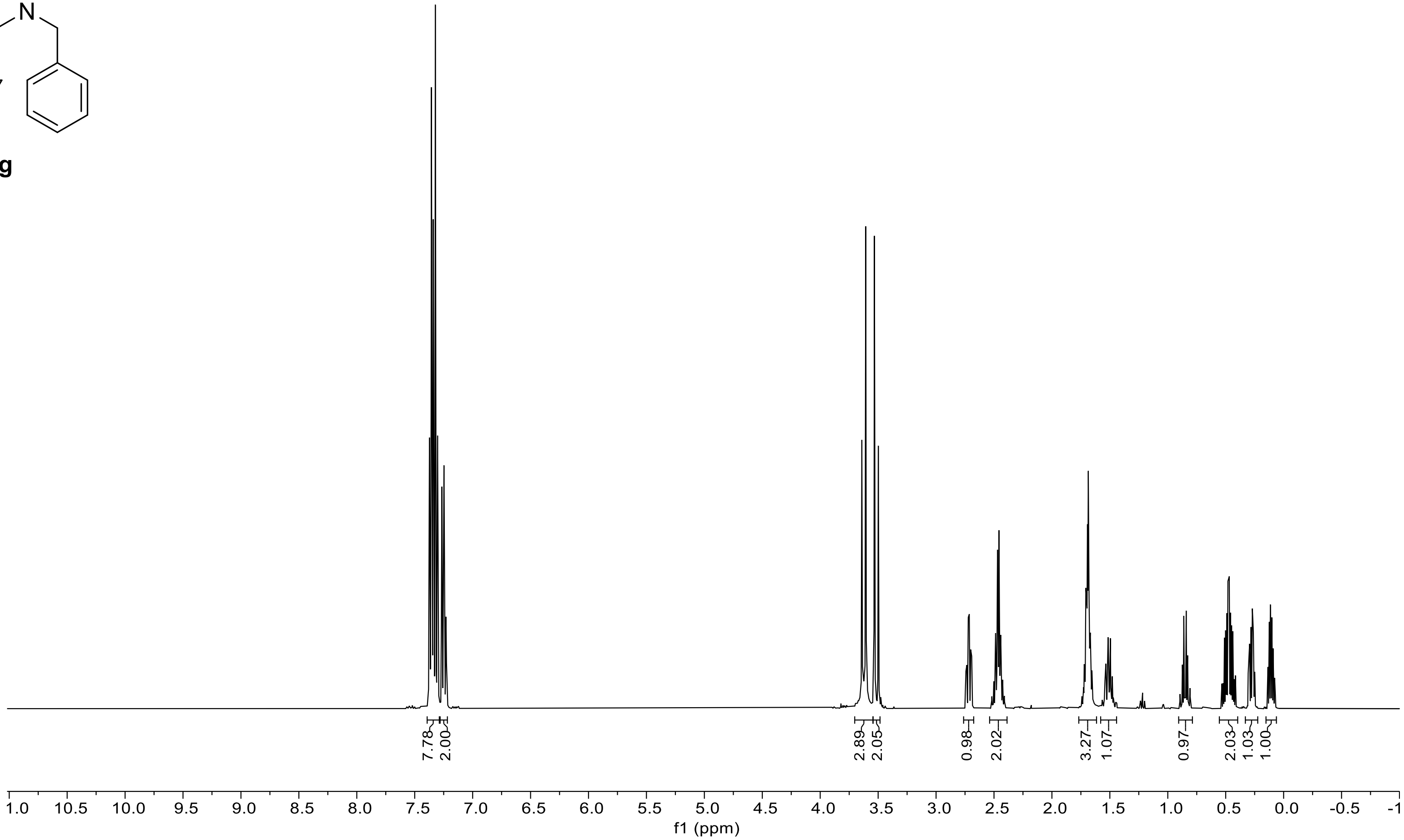

^{13}C NMR ($CDCl_3$, 101 MHz)

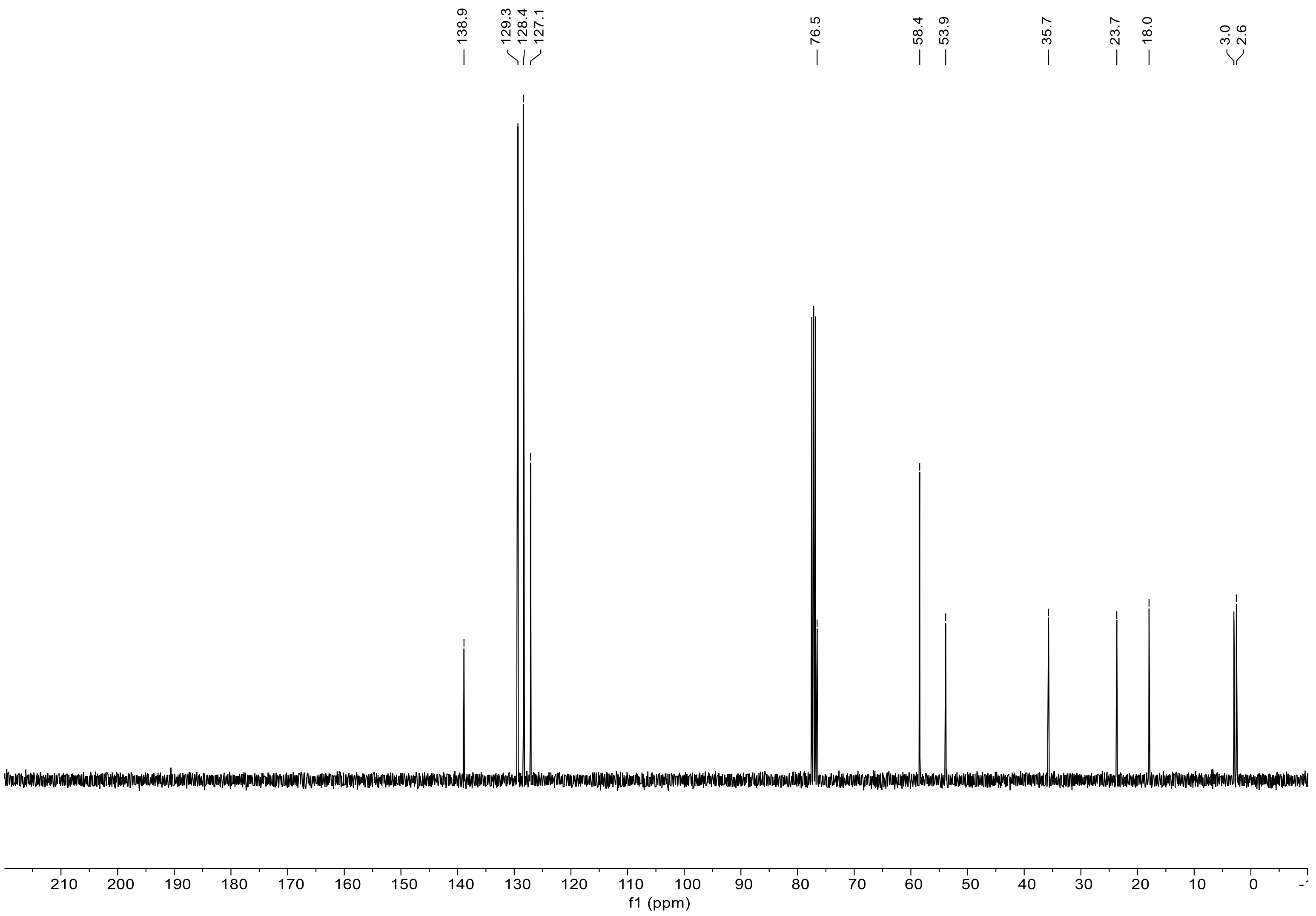

1-Cyclopropyl-4-(methylthio)butan-1-ol (2h)
^{1}H NMR ($CDCl_3$, 400 MHz)

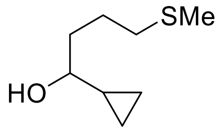

2h

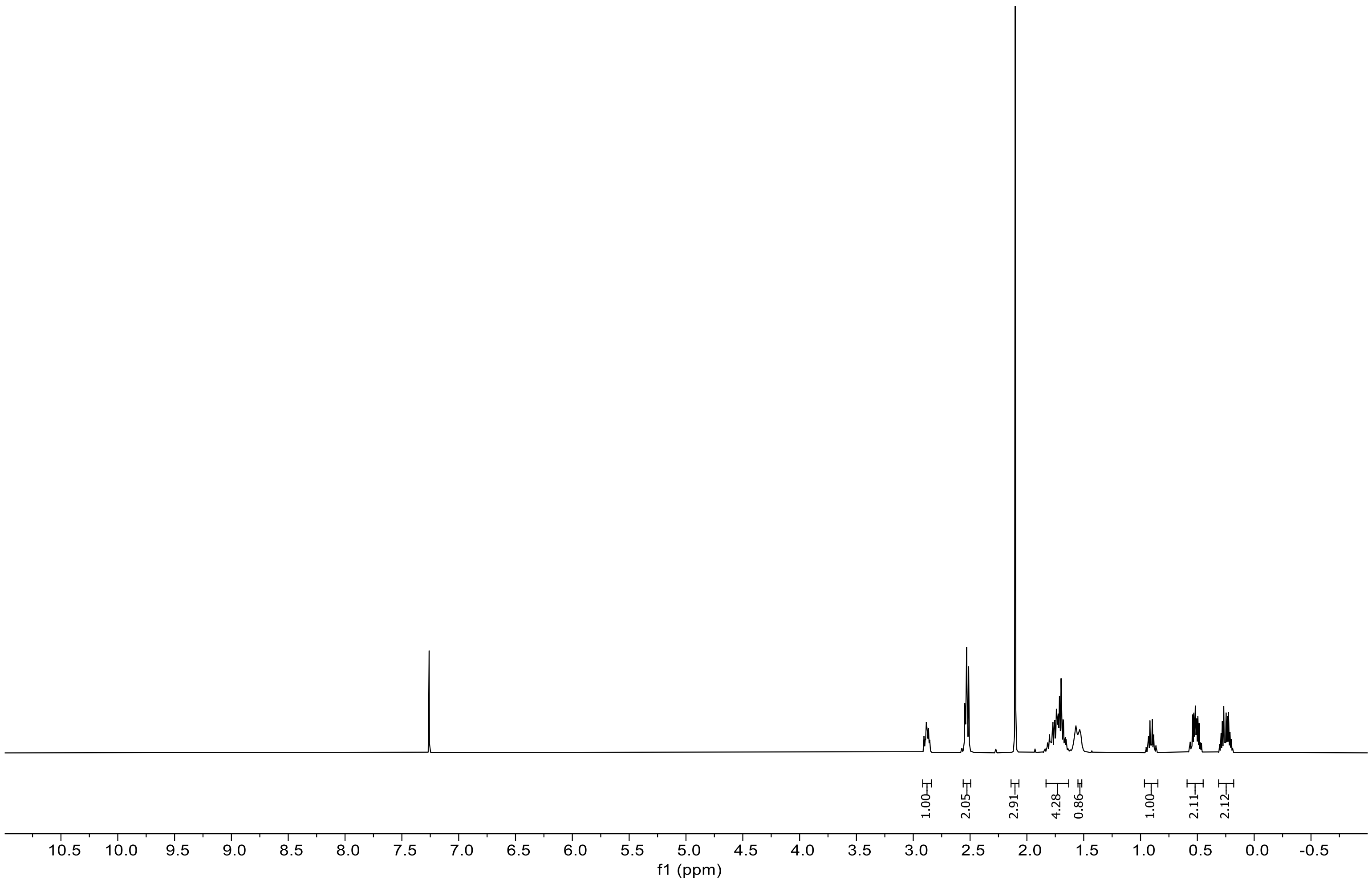

^{13}C NMR ($CDCl_3$, 101 MHz)

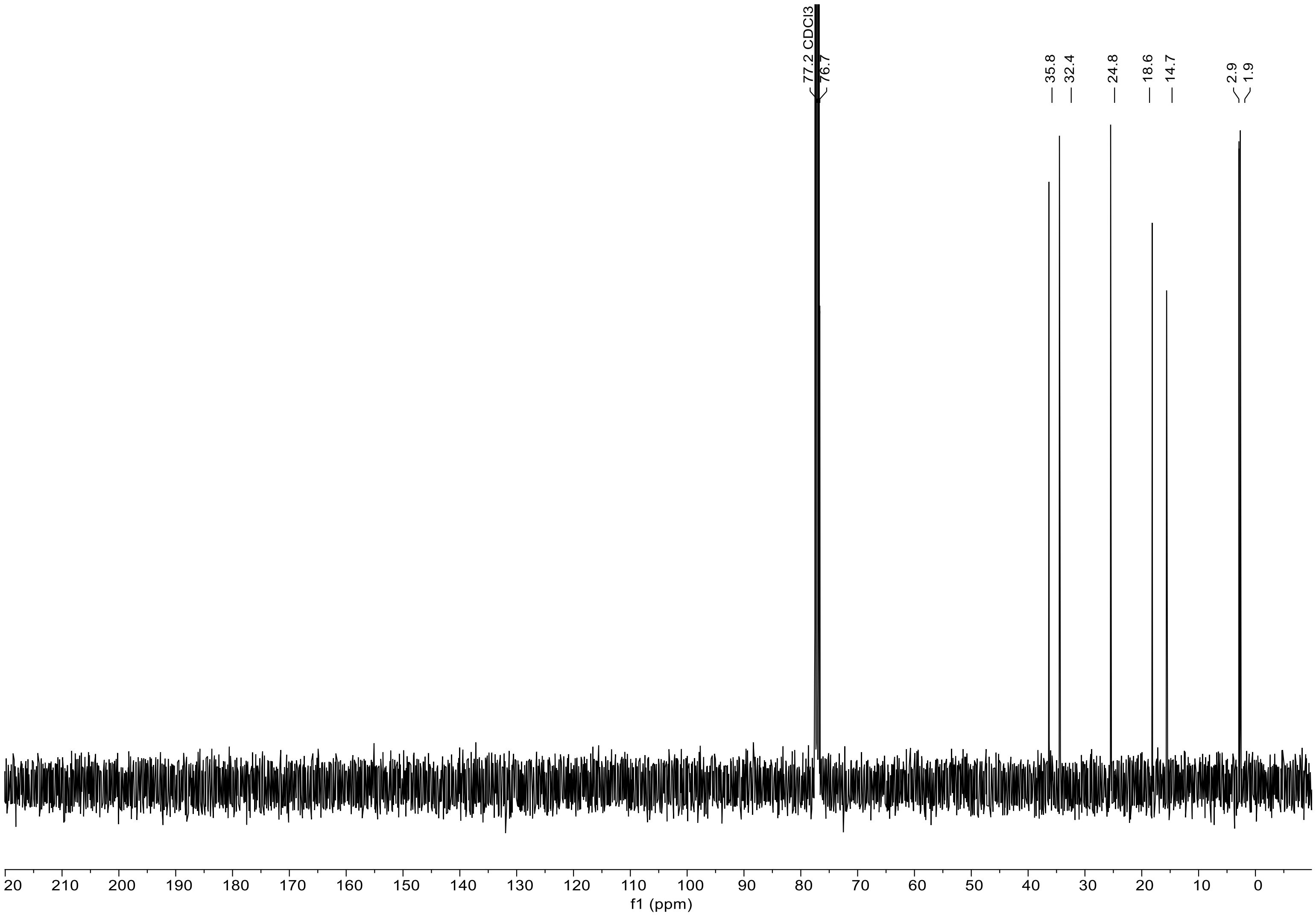

1-Cyclopropyl-3-(pyridin-2-yl)propan-1-ol (2i)
^{1}H NMR ($CDCl_3$, 400 MHz)

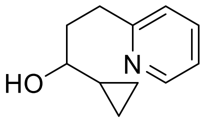

2i

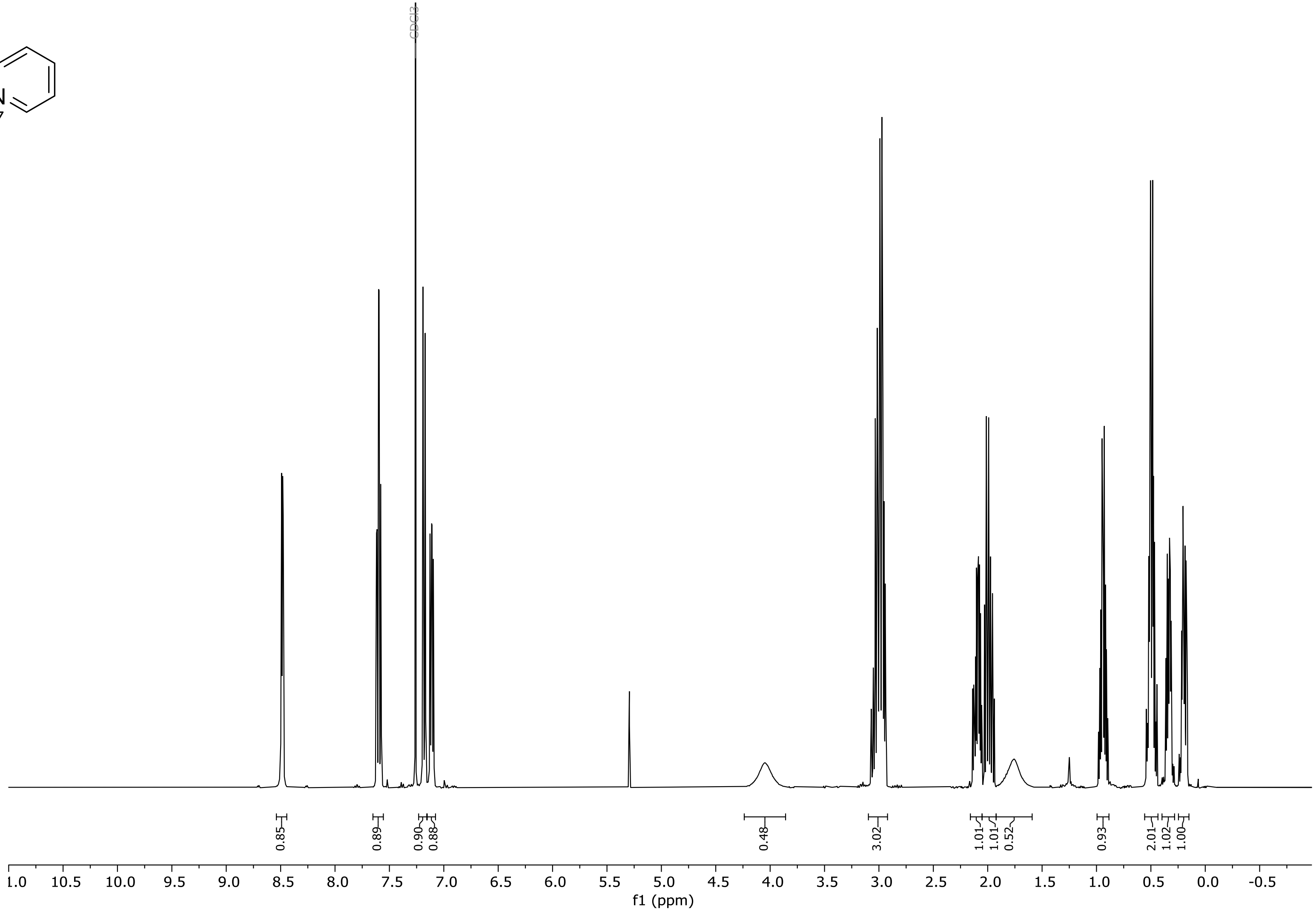

^{13}C NMR ($CDCl_3$, 101 MHz)

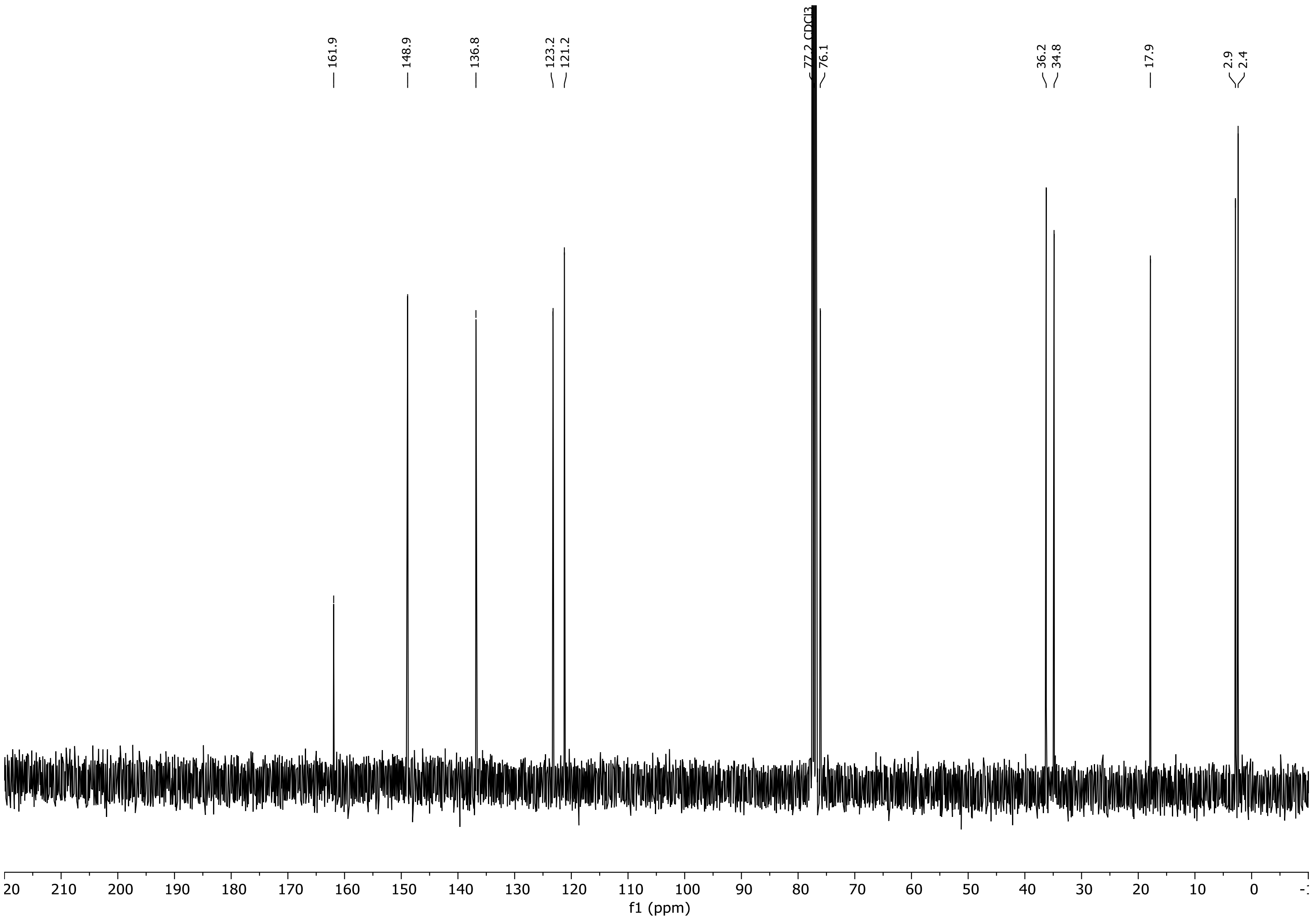

1-Cyclopropyl-4-phenylbutan-1-ol (2e)
^{1}H NMR ($CDCl_3$, 400 MHz)

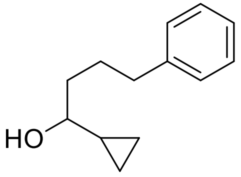

2e

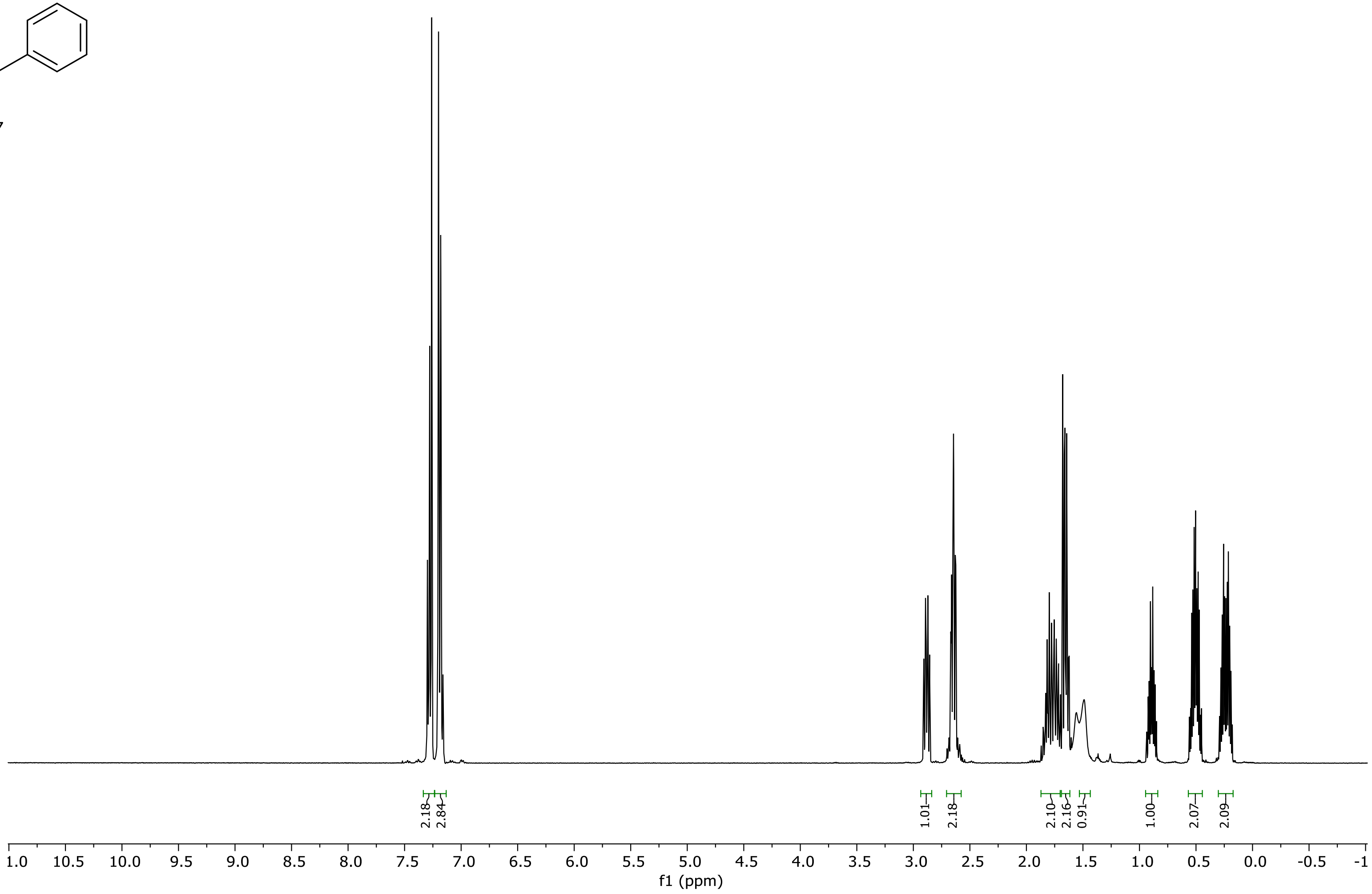

^{13}**C NMR** ($CDCl_3$, 101 MHz)

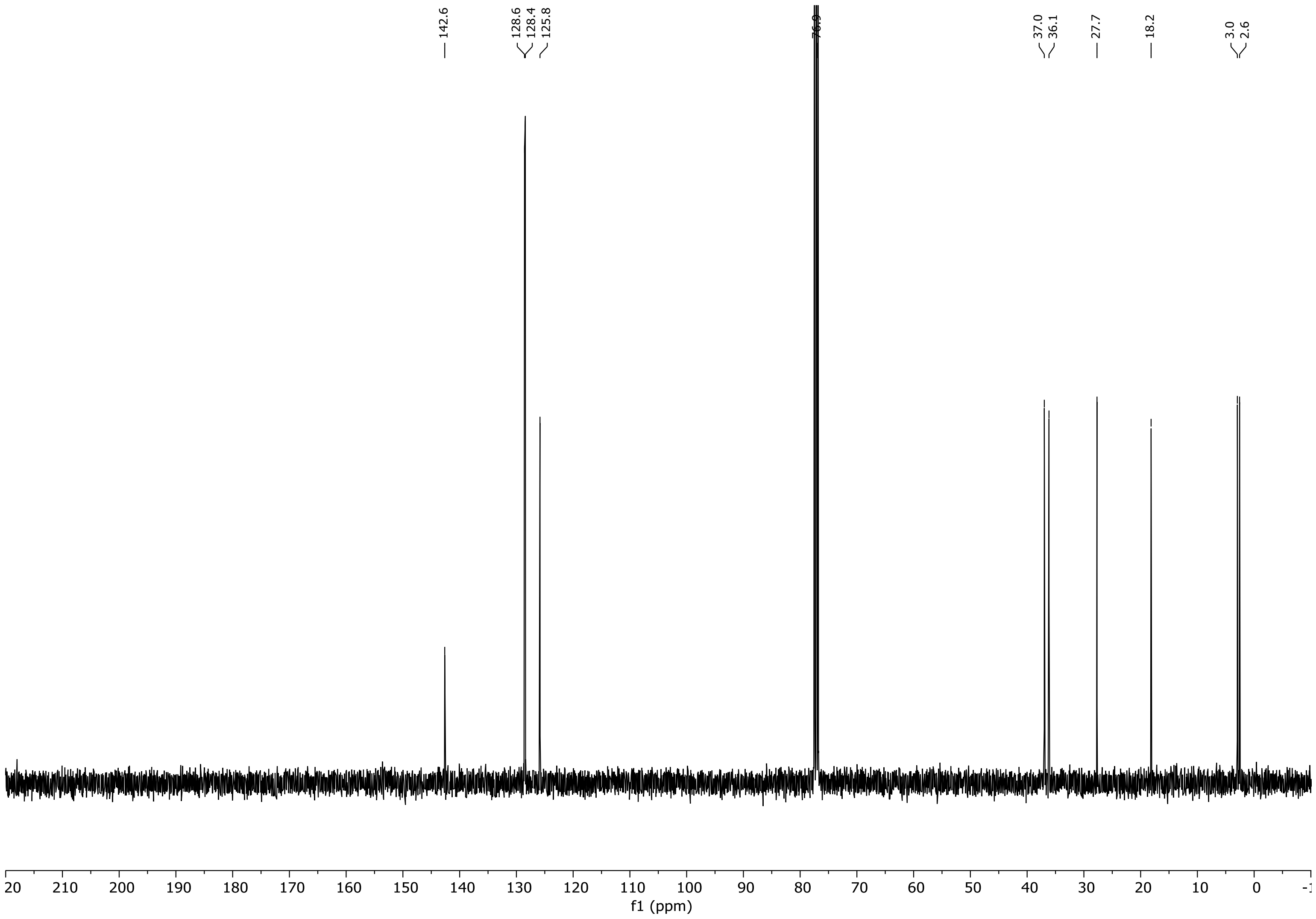

***trans*-2-Phenylcyclopropyl)methanol**
^{1}H NMR ($CDCl_3$, 400 MHz)

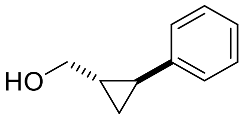

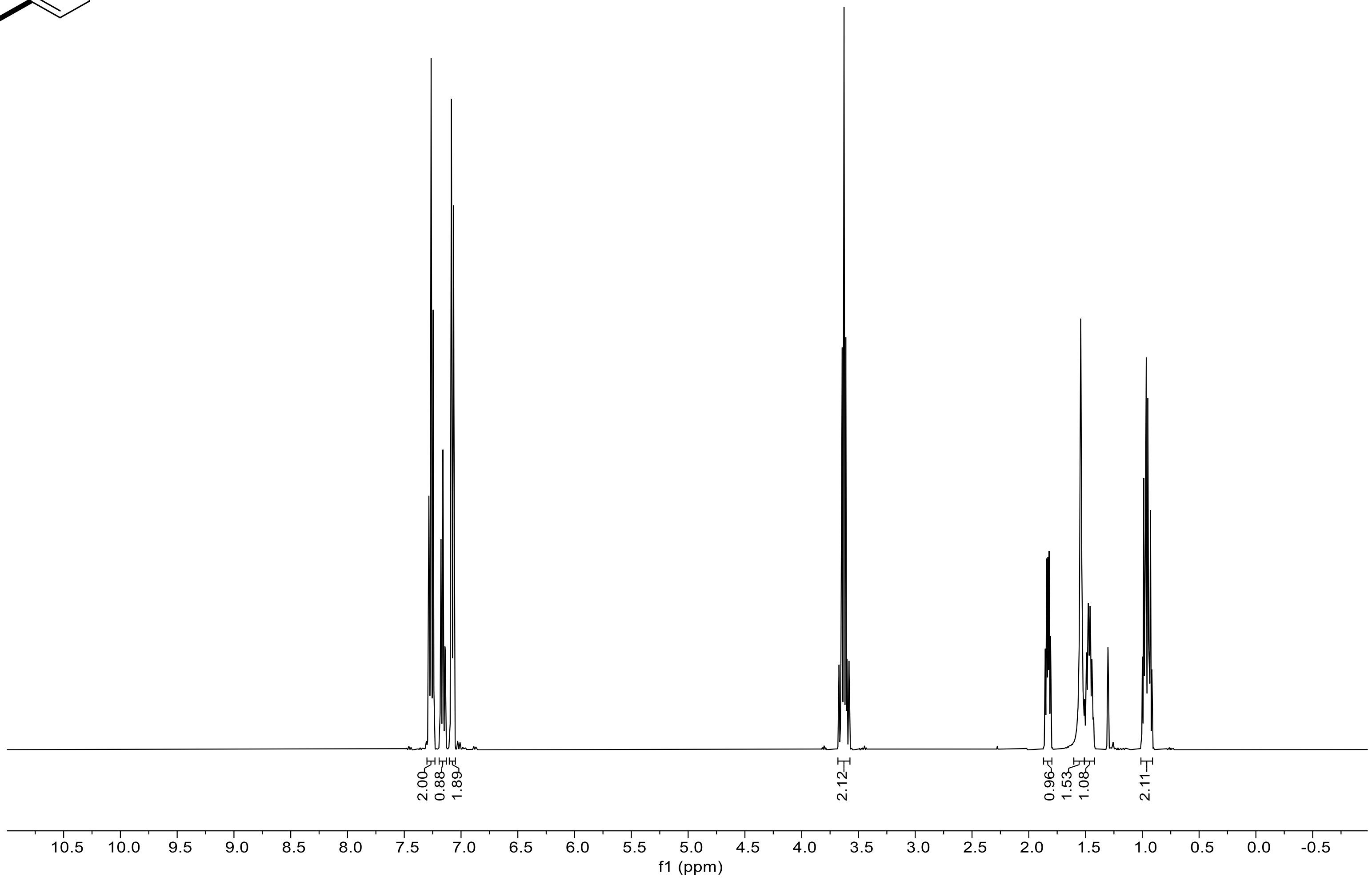

[13]**C NMR** (CDCl$_3$, 101 MHz)

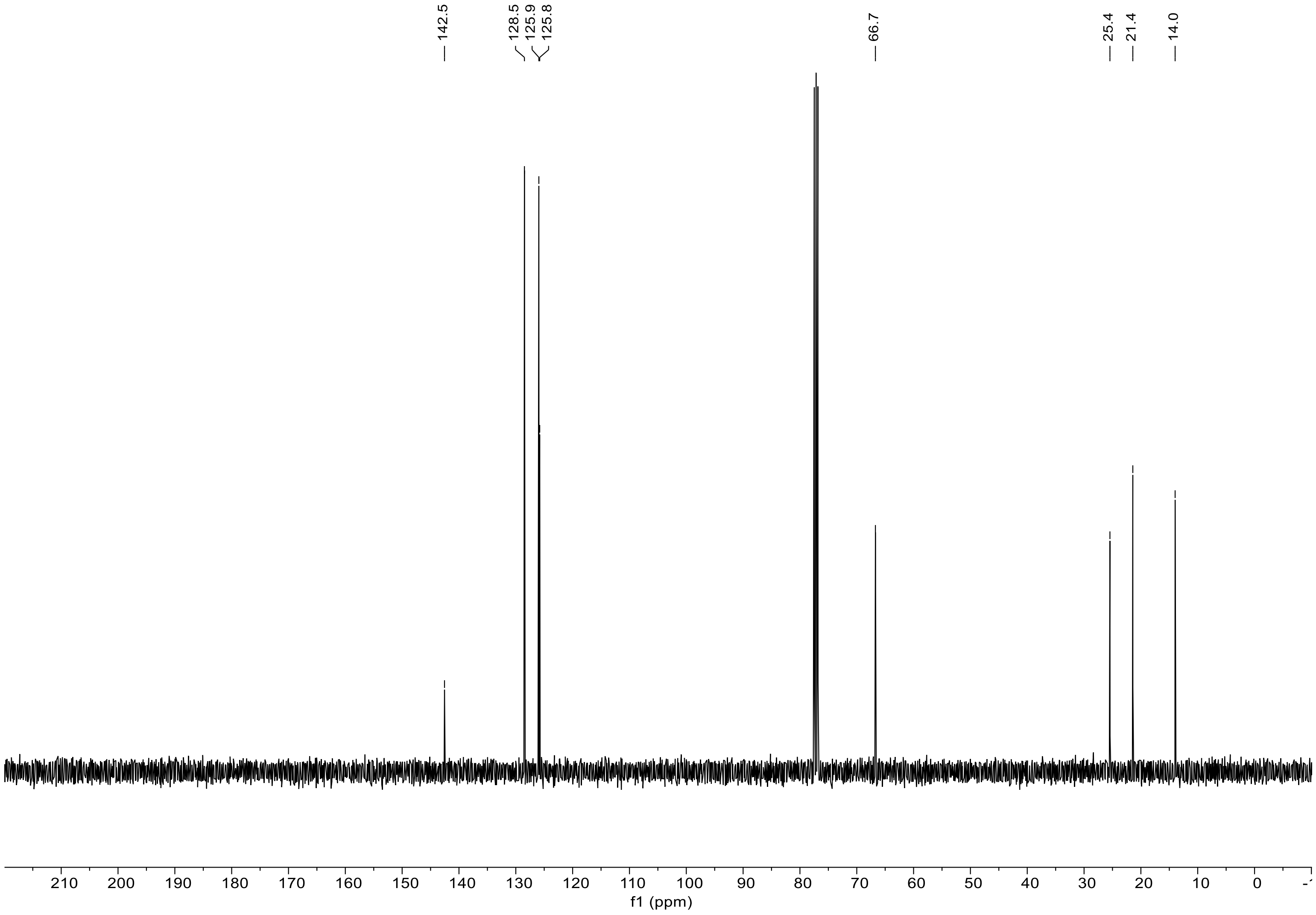

***trans*-2-Phenylcyclopropylethan-1-ol (major, 2j)**
^{1}H NMR ($CDCl_3$, 400 MHz)

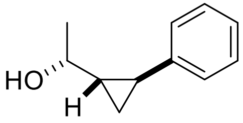

2j (major)

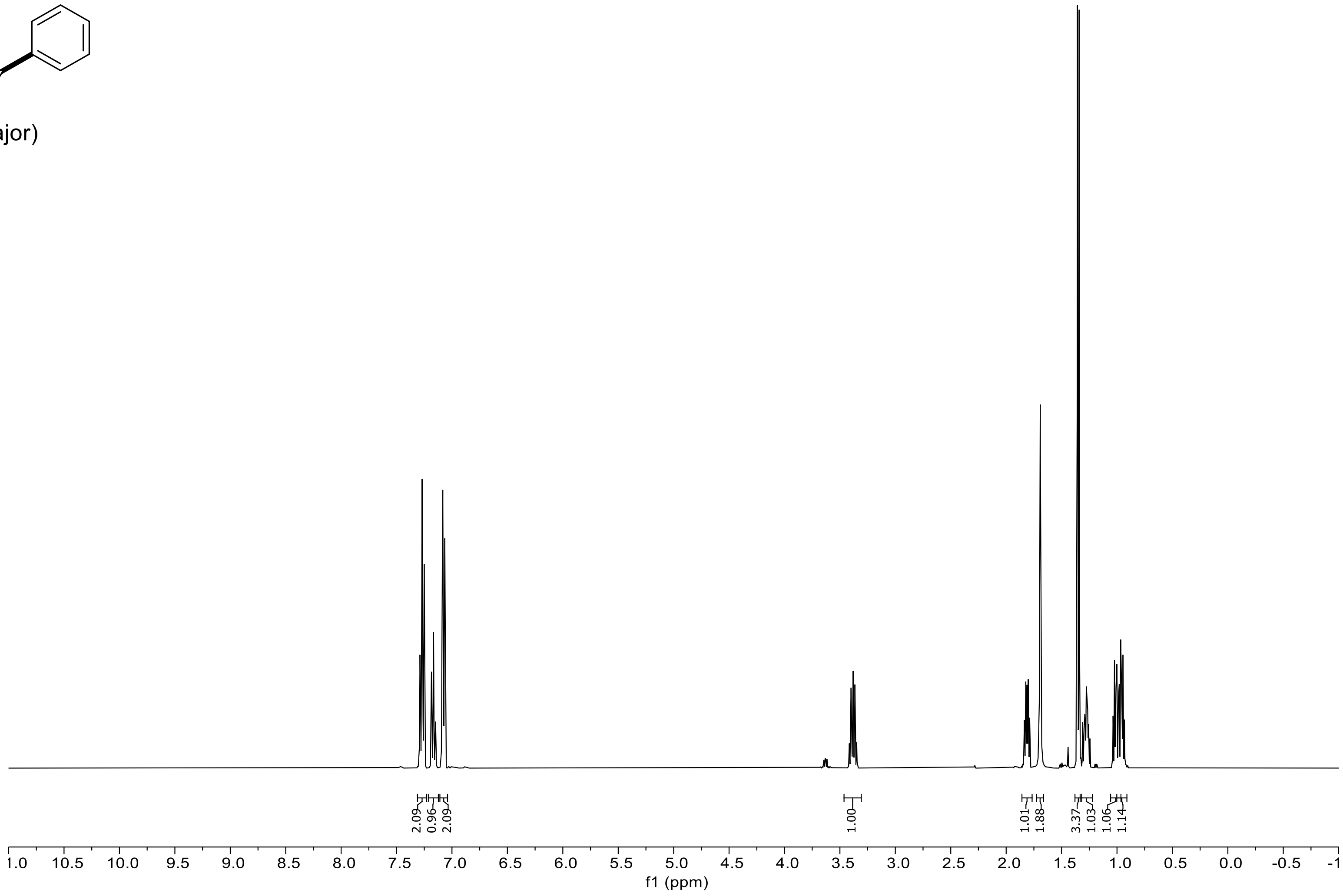

^{13}C NMR ($CDCl_3$, 101 MHz)

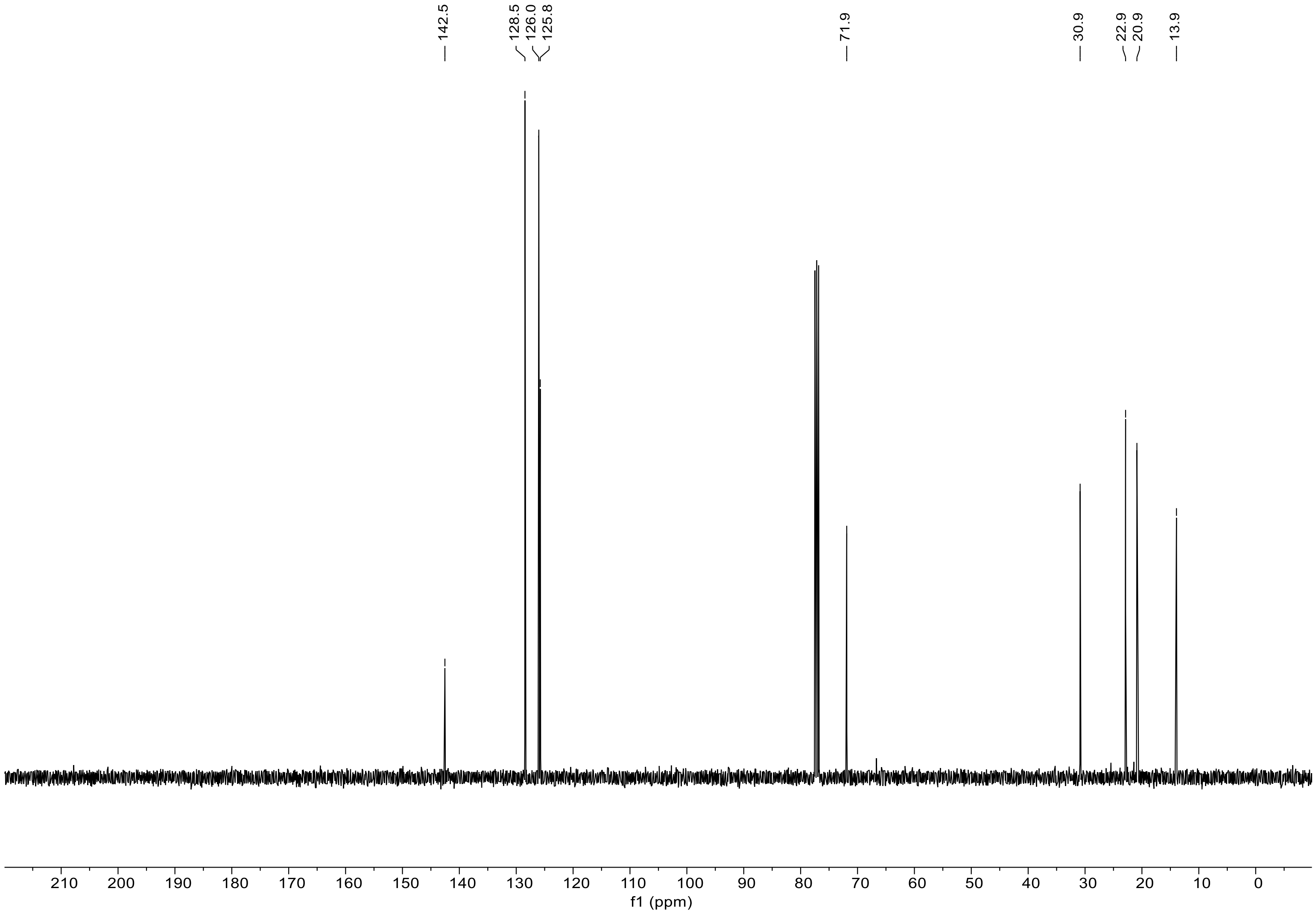

***trans*-2-Phenylcyclopropylethan-1-ol (minor, 2j')**
^{1}H NMR ($CDCl_3$, 500 MHz)

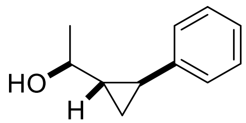

2j' (minor)

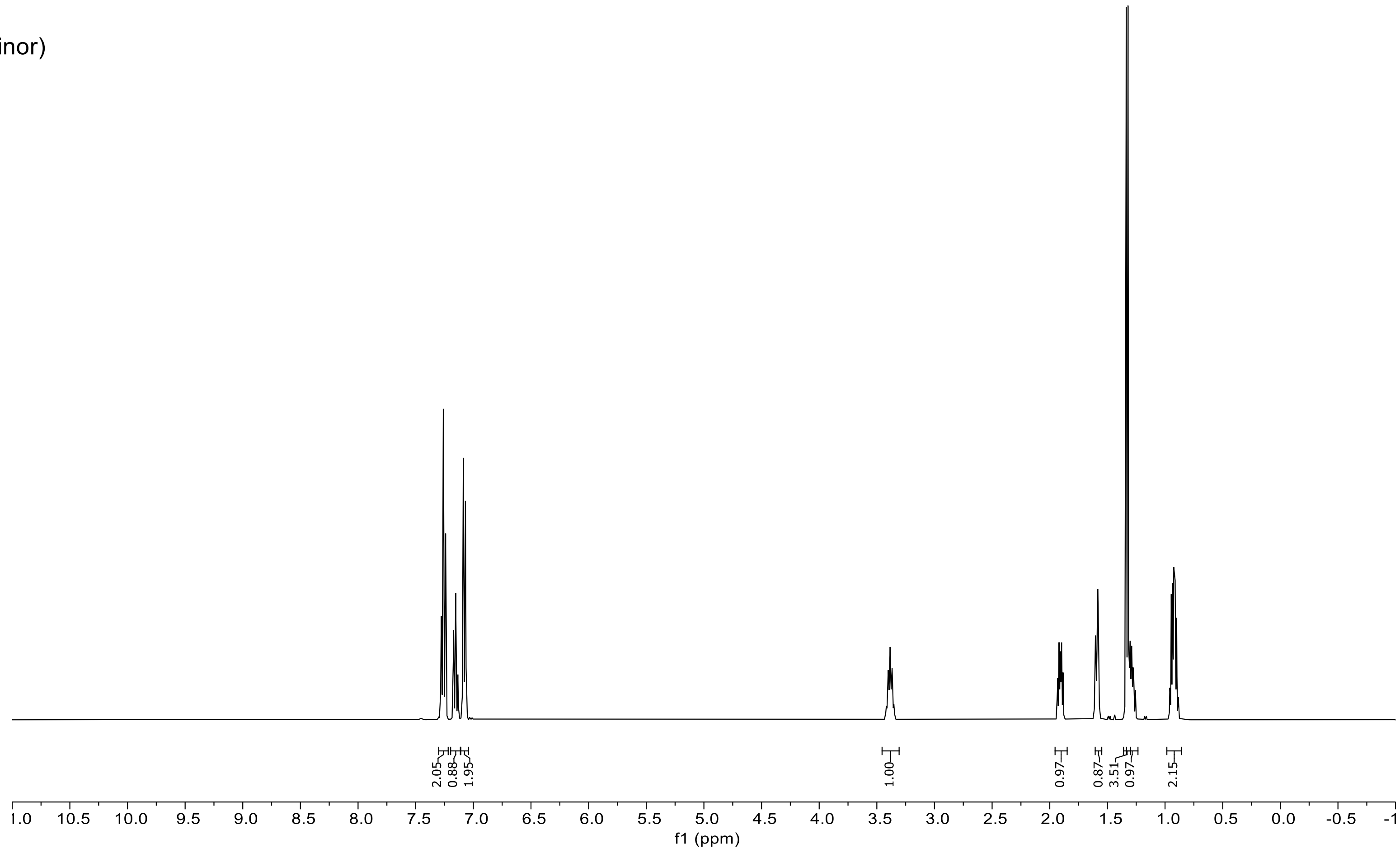

^{13}C NMR ($CDCl_3$, 126 MHz)

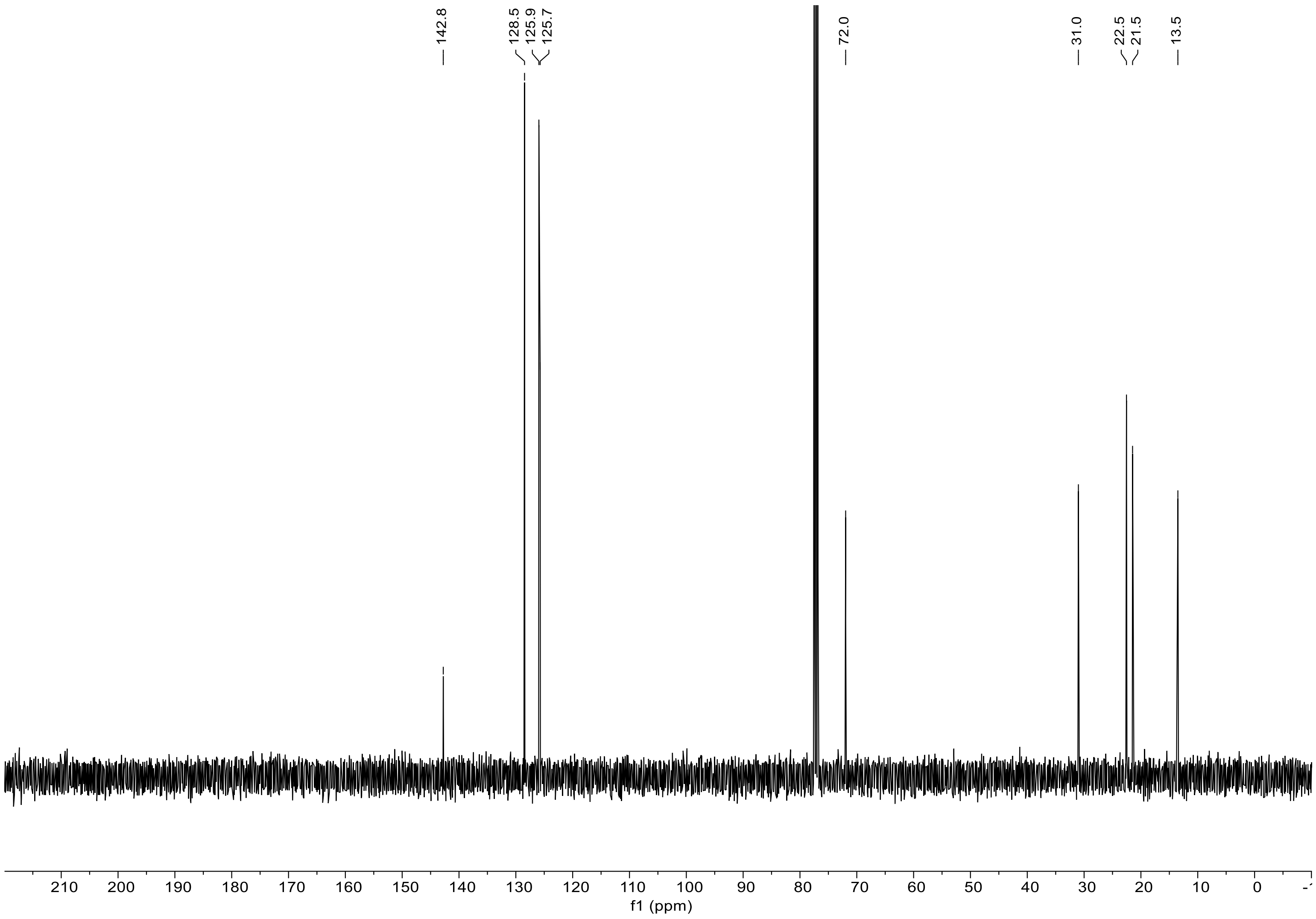

1-(Bicyclo[4.1.0]heptan-1-yl)ethan-1-one
^{1}H NMR ($CDCl_3$, 500 MHz)

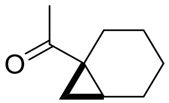

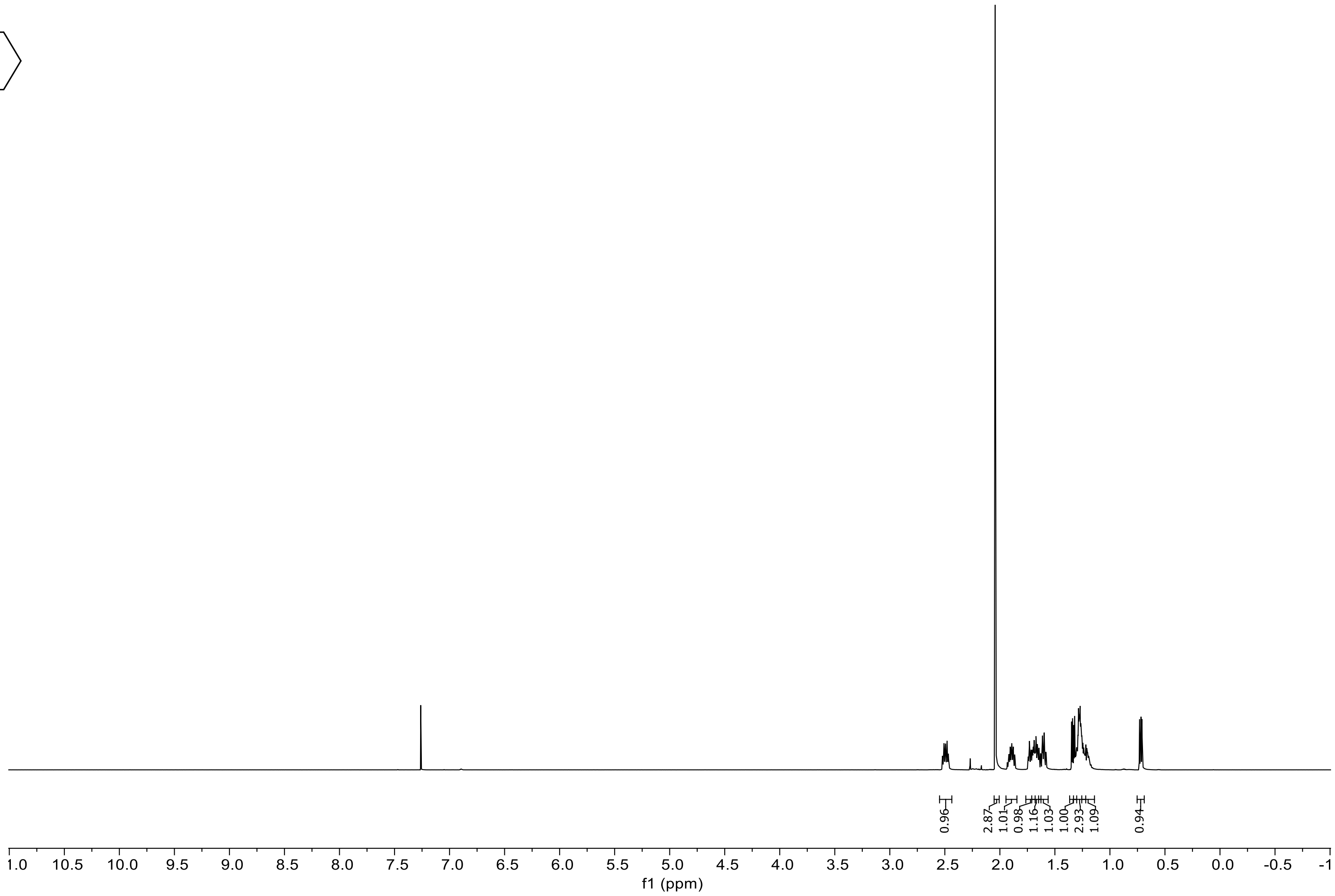

^{13}C NMR ($CDCl_3$, 126 MHz)

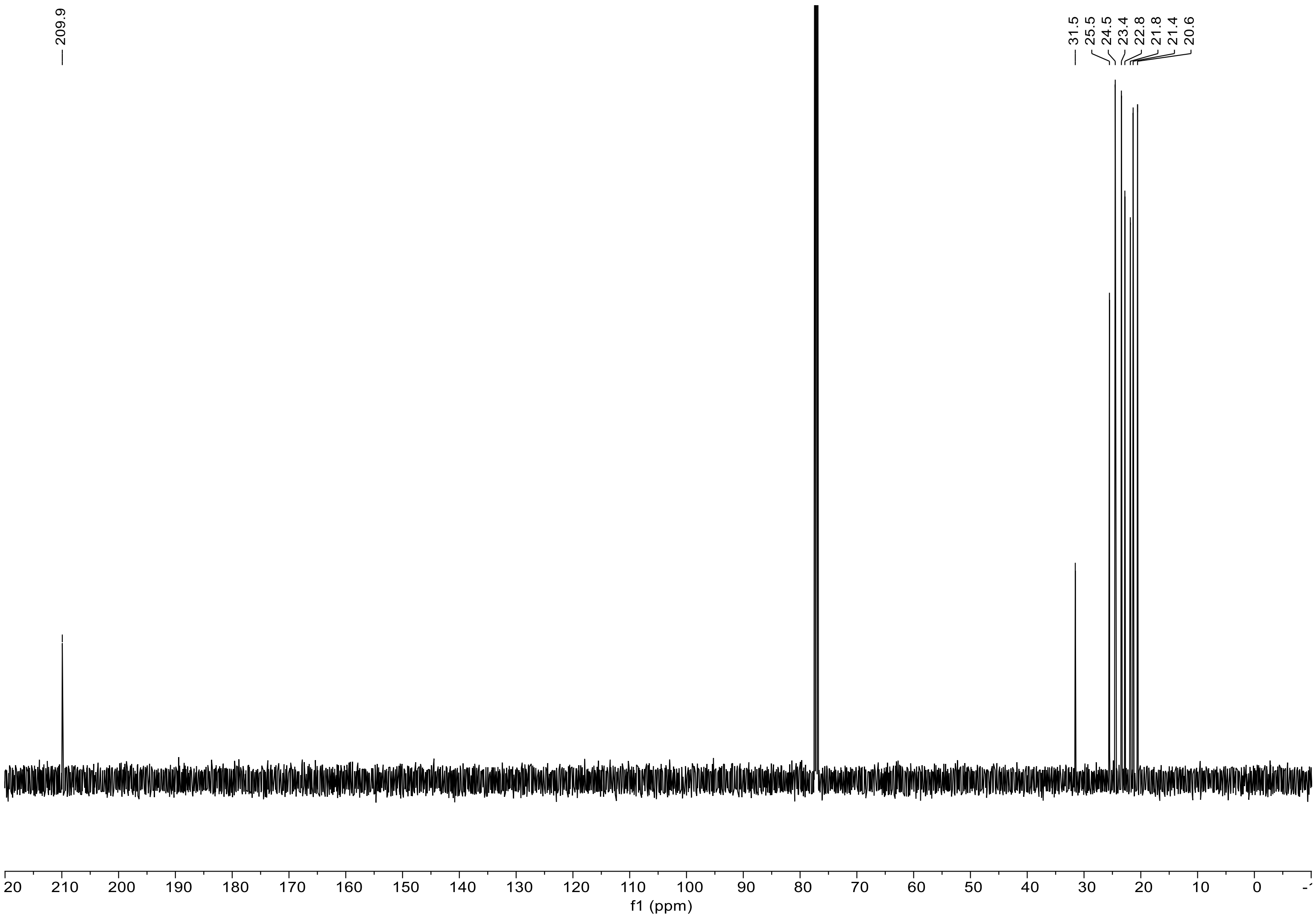

1-(Bicyclo[4.1.0]heptan-1-yl)ethan-1-ol (2l)
^{1}H NMR ($CDCl_3$, 500 MHz)

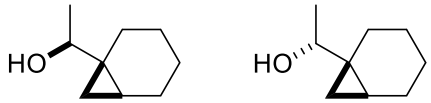

2l **2l'**

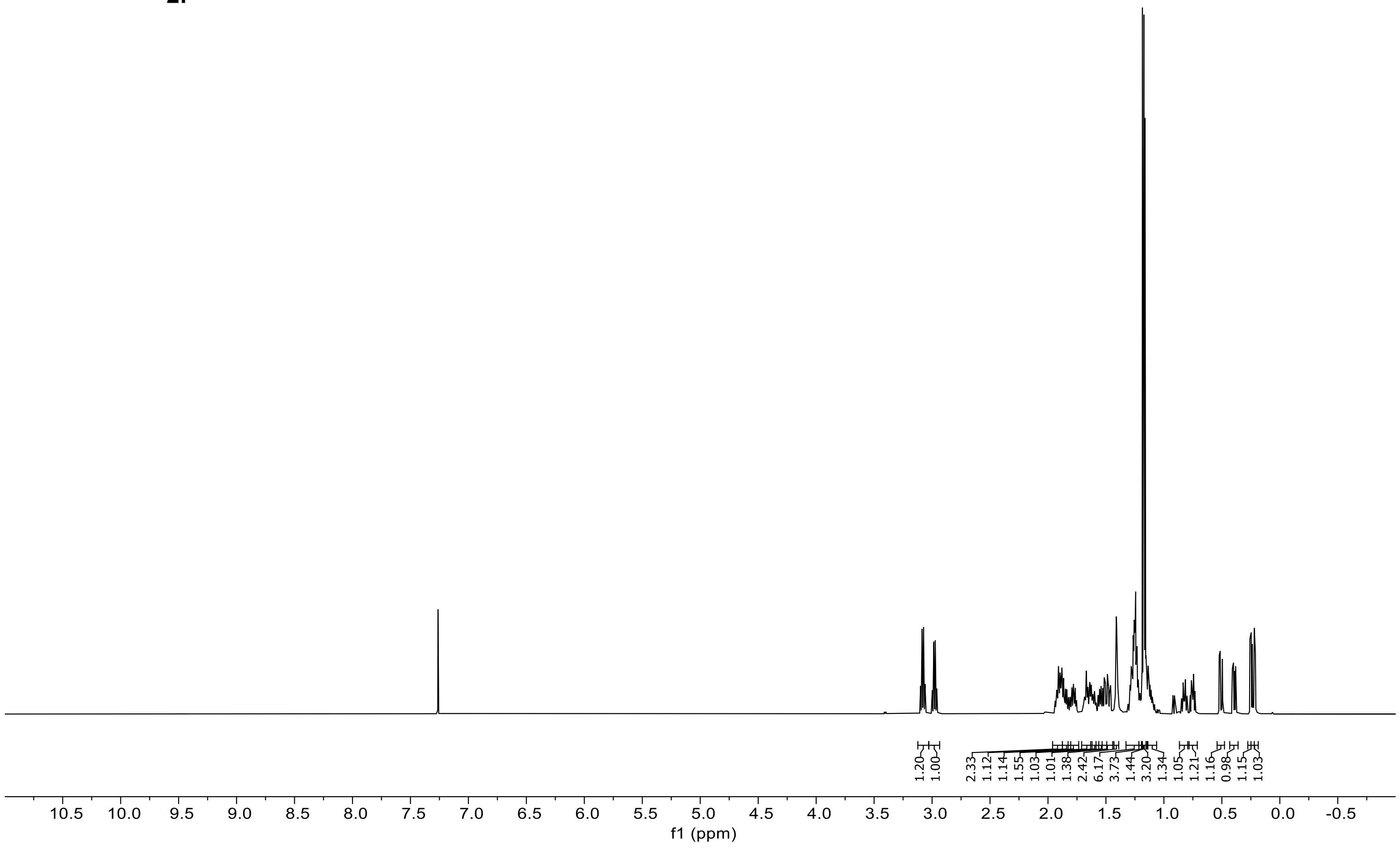

^{13}C NMR ($CDCl_3$, 126 MHz)

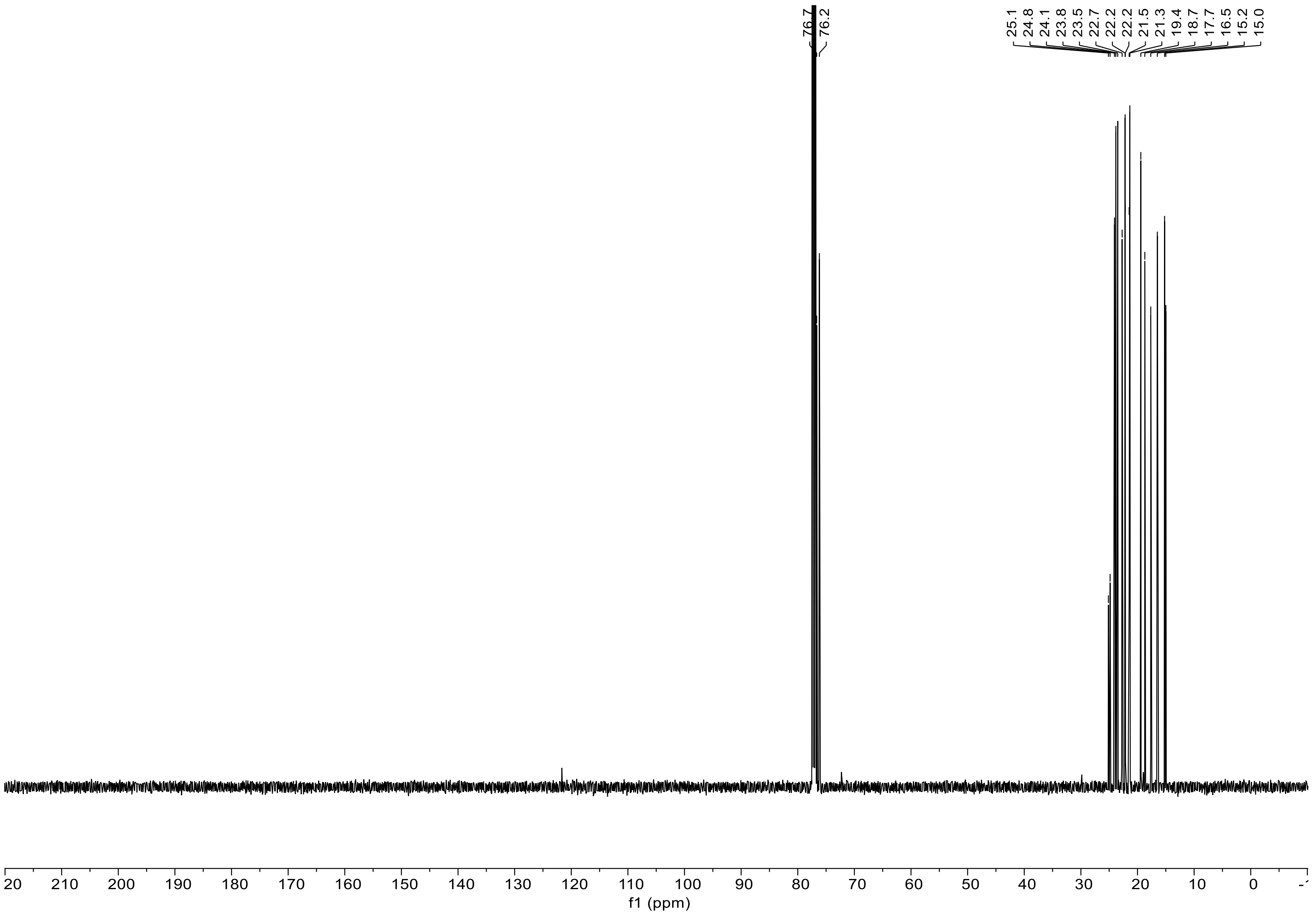

2-Methylenecyclohexan-1-ol
¹H NMR ($CDCl_3$, 500 MHz)

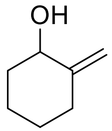

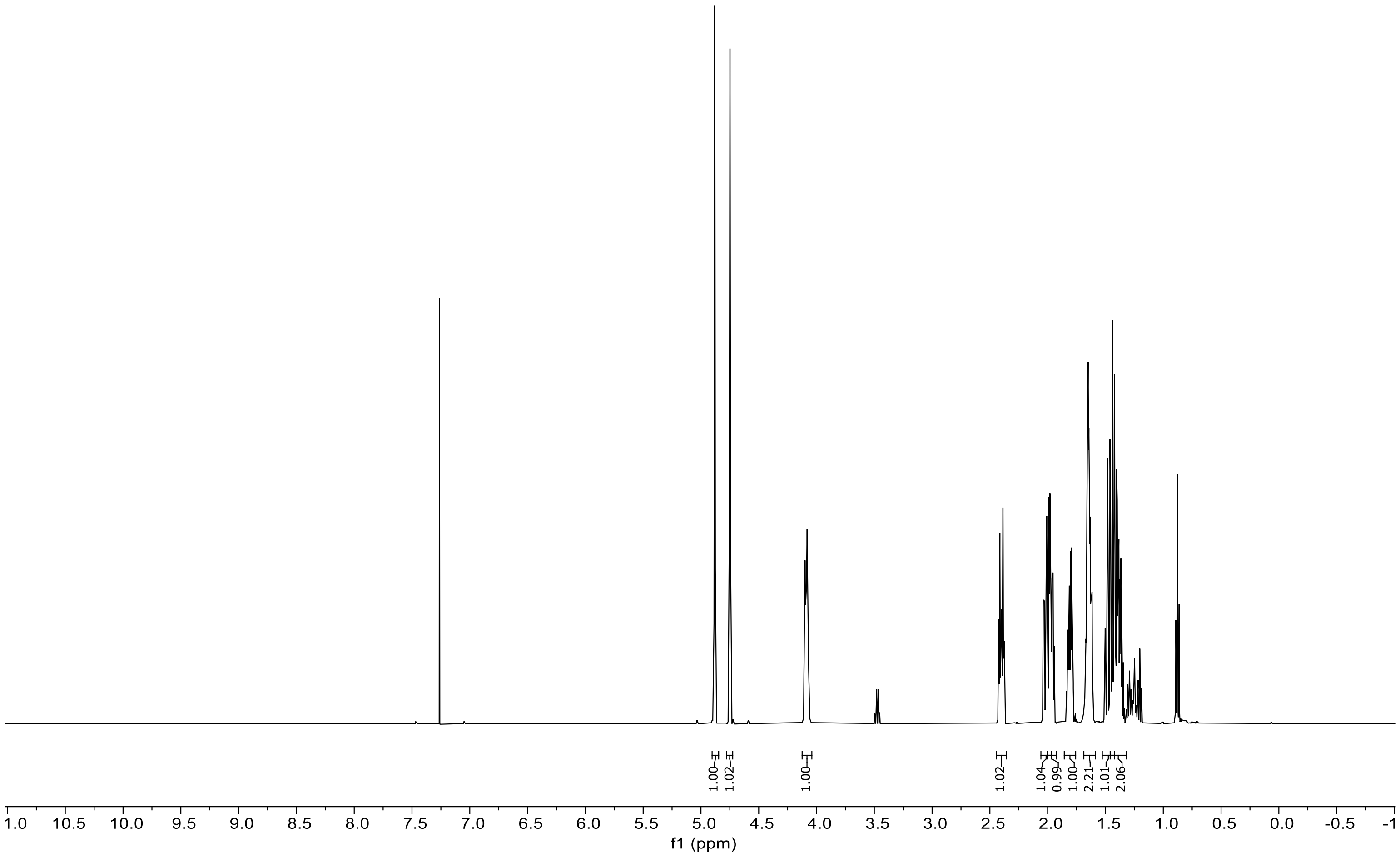

13C NMR ($CDCl_3$, 126 MHz)

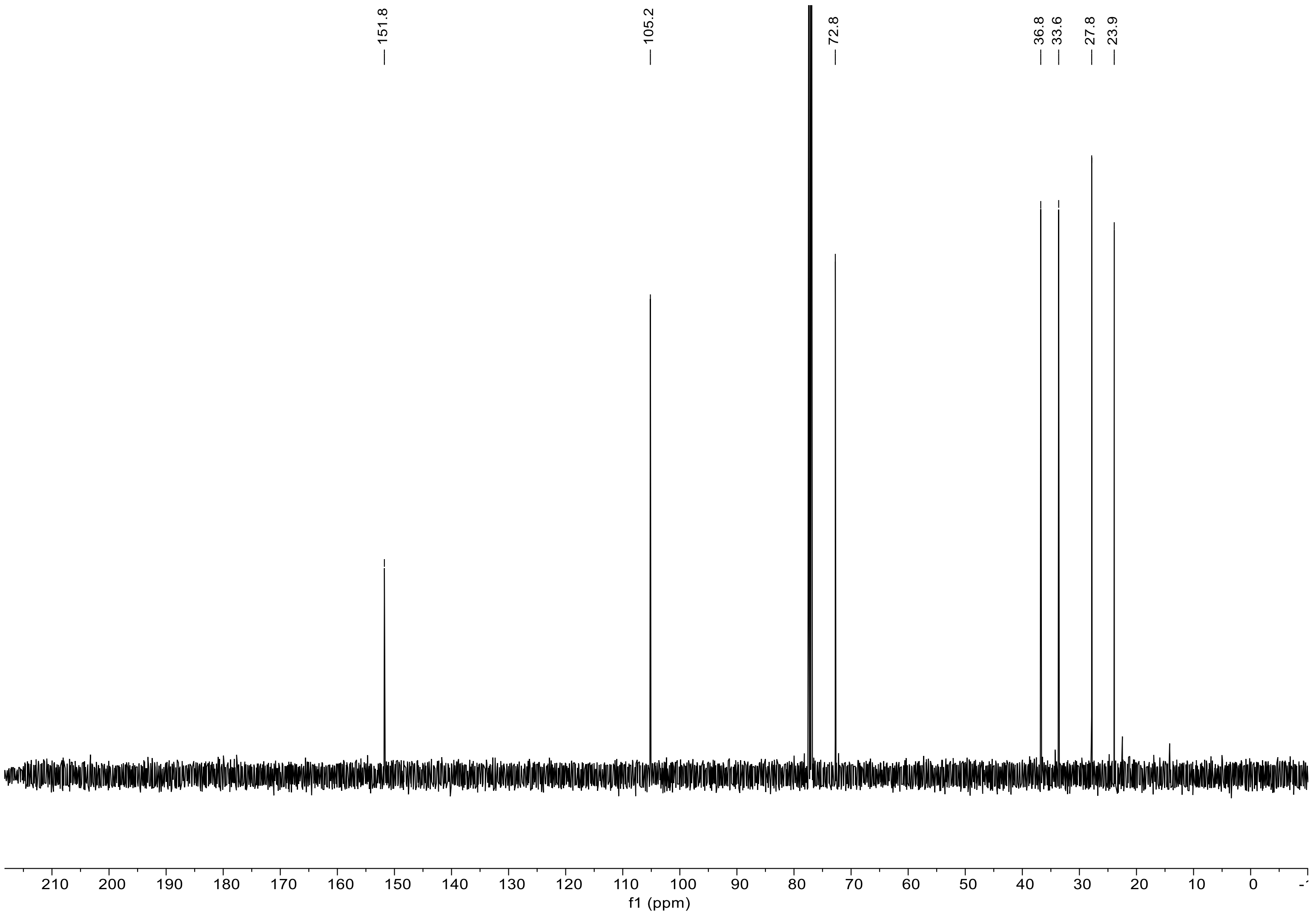

Spiro[2.5]octan-4-ol (2m)
¹H NMR ($CDCl_3$, 500 MHz)

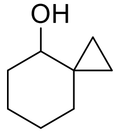

2m

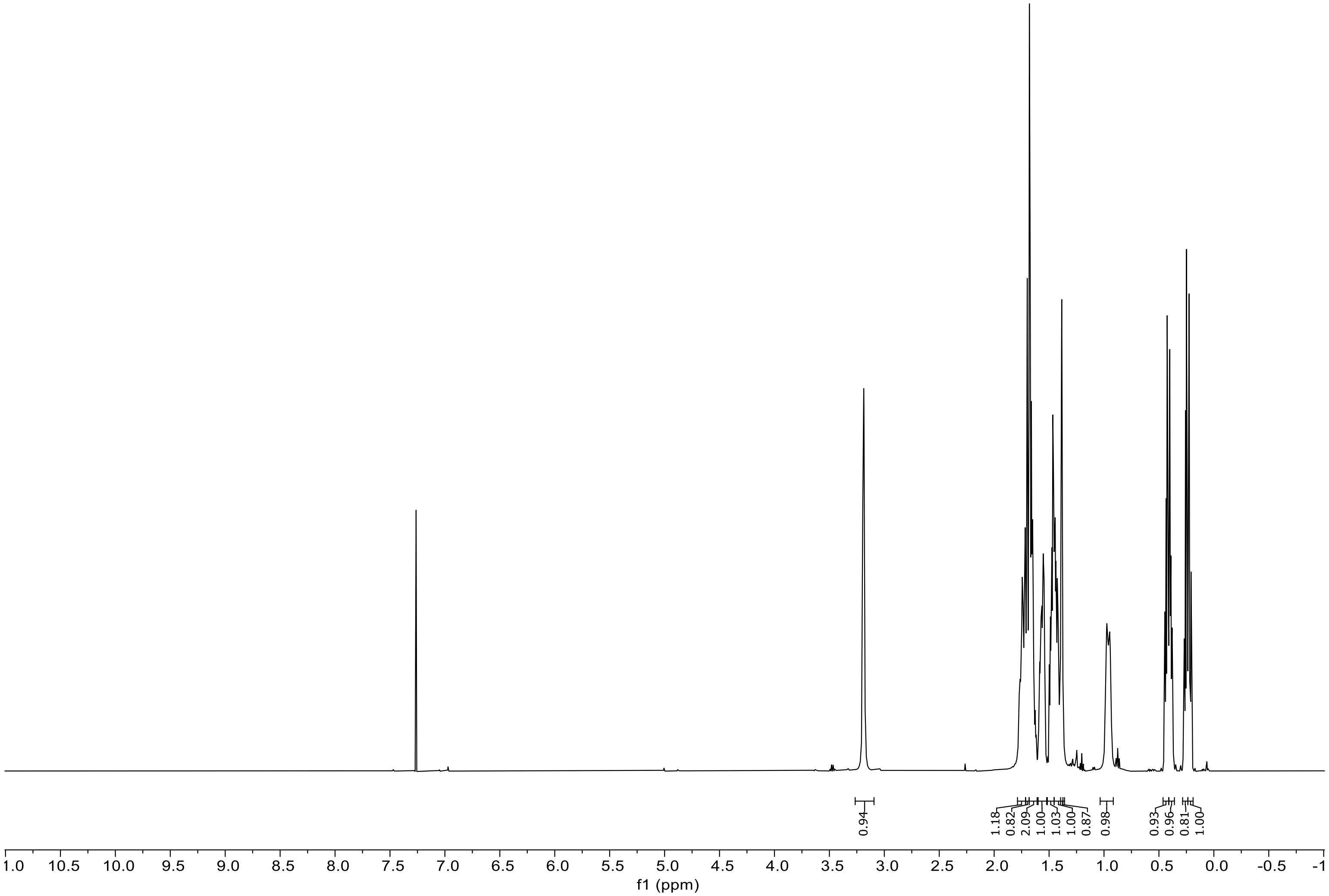

^{13}C NMR ($CDCl_3$, 126 MHz)

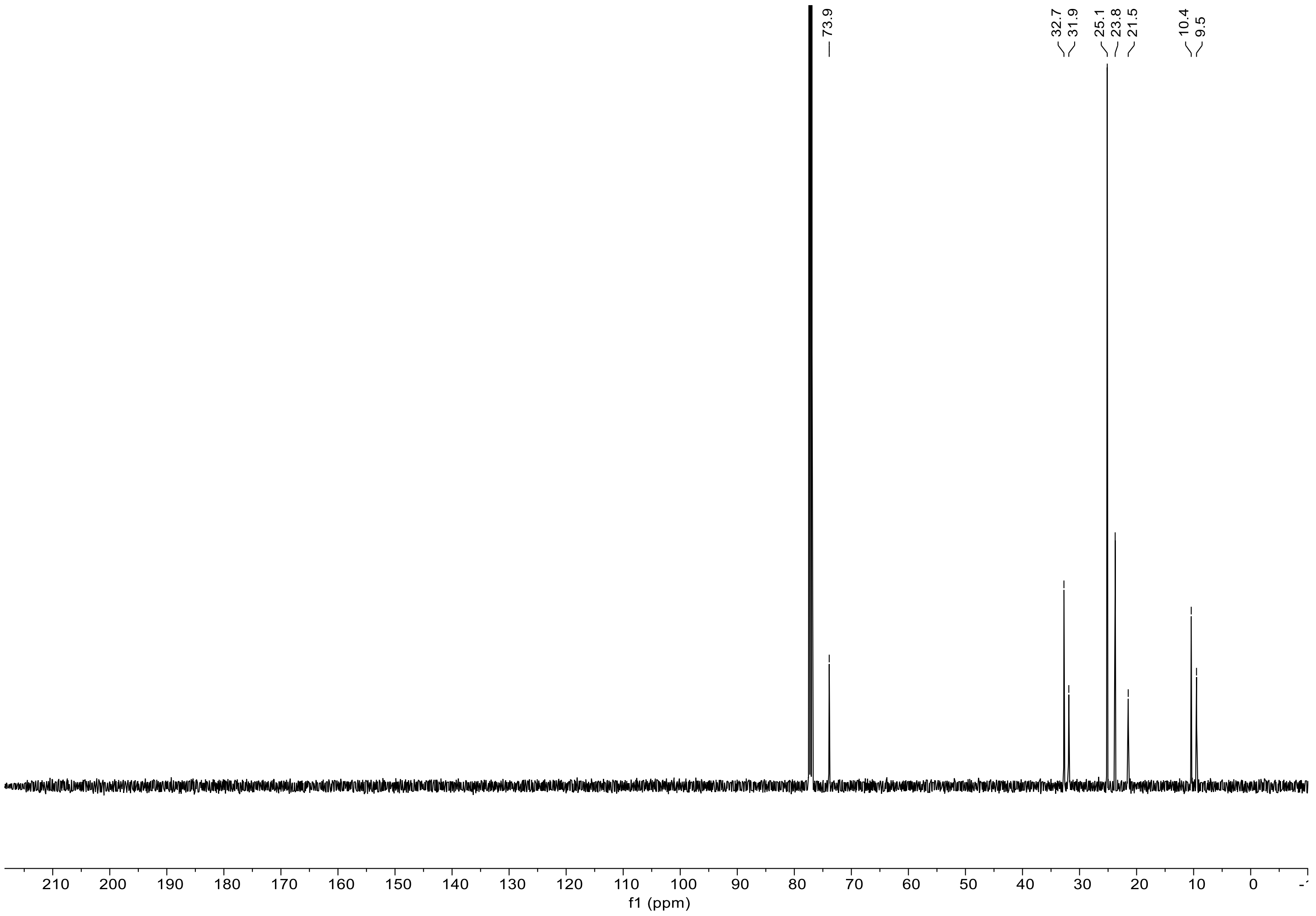

3-Cyclopropyl-3-hydroxy-1-(2,3,4,5,6-pentamethylphenyl)pentan-1-one (7)
^{1}H NMR ($CDCl_3$, 400 MHz)

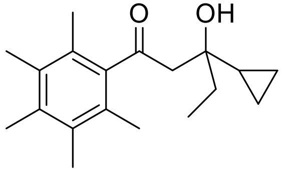

7

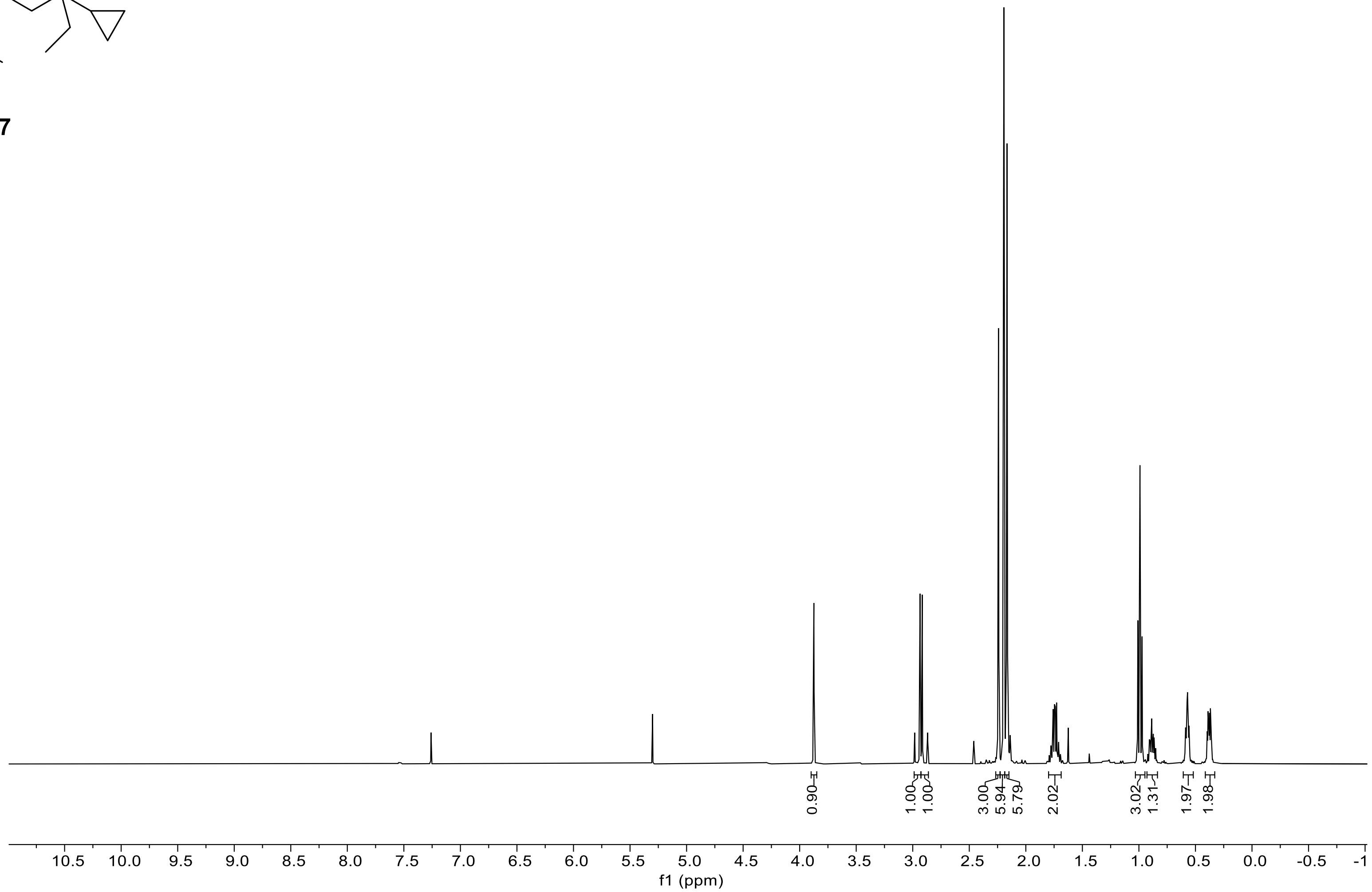

^{13}C NMR ($CDCl_3$, 101 MHz)

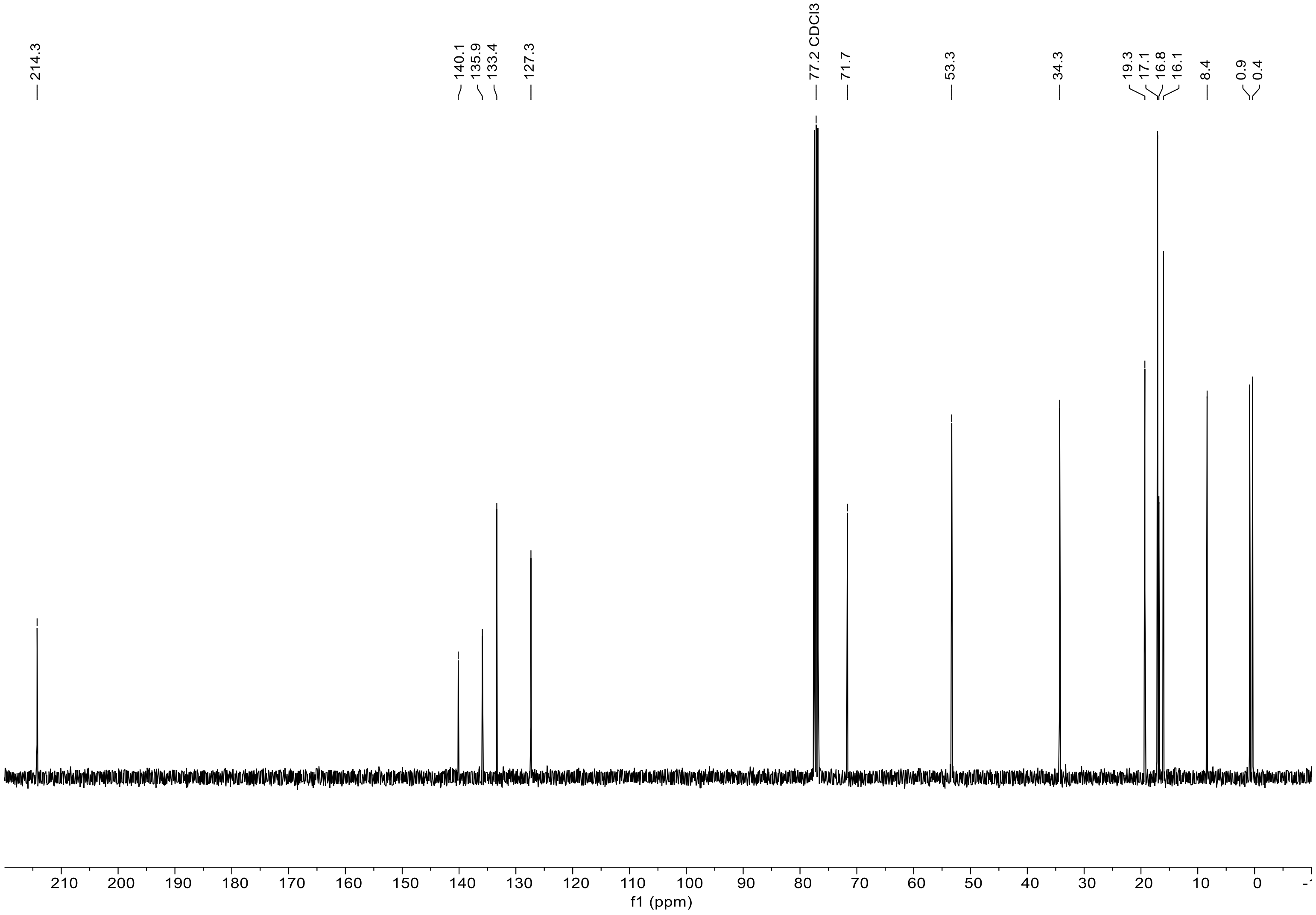

(*E/Z*)-3-Cyclopropyl-1-(2,3,4,5,6-pentamethylphenyl)pent-2-en-1-one ((*E/Z*)-6)
^{1}H NMR ($CDCl_3$, 400 MHz)

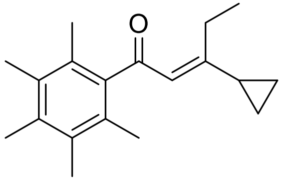

(*E*)-**6**

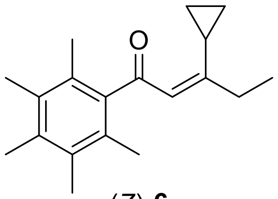

(*Z*)-**6**

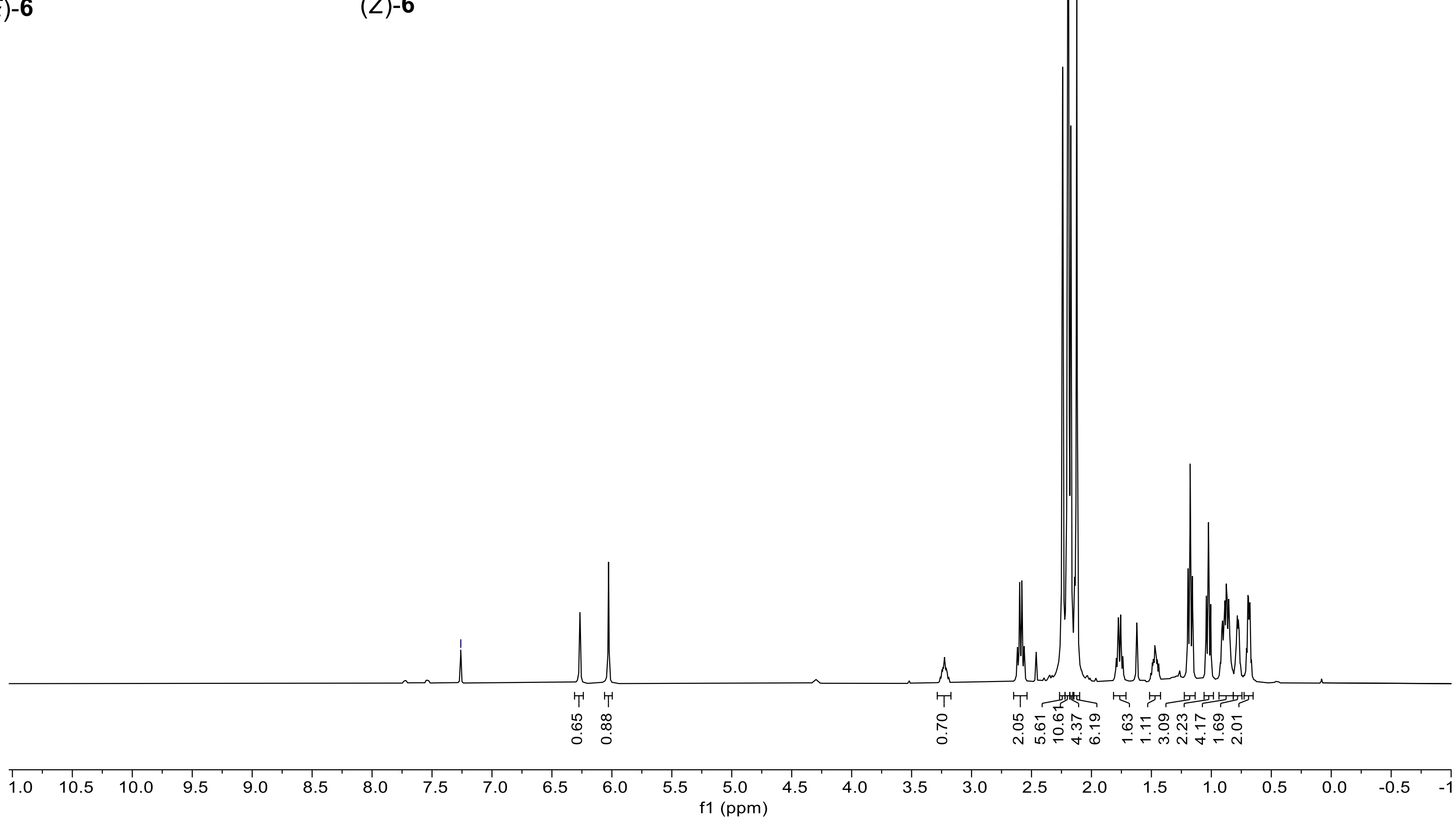

^{13}C NMR ($CDCl_3$, 101 MHz)

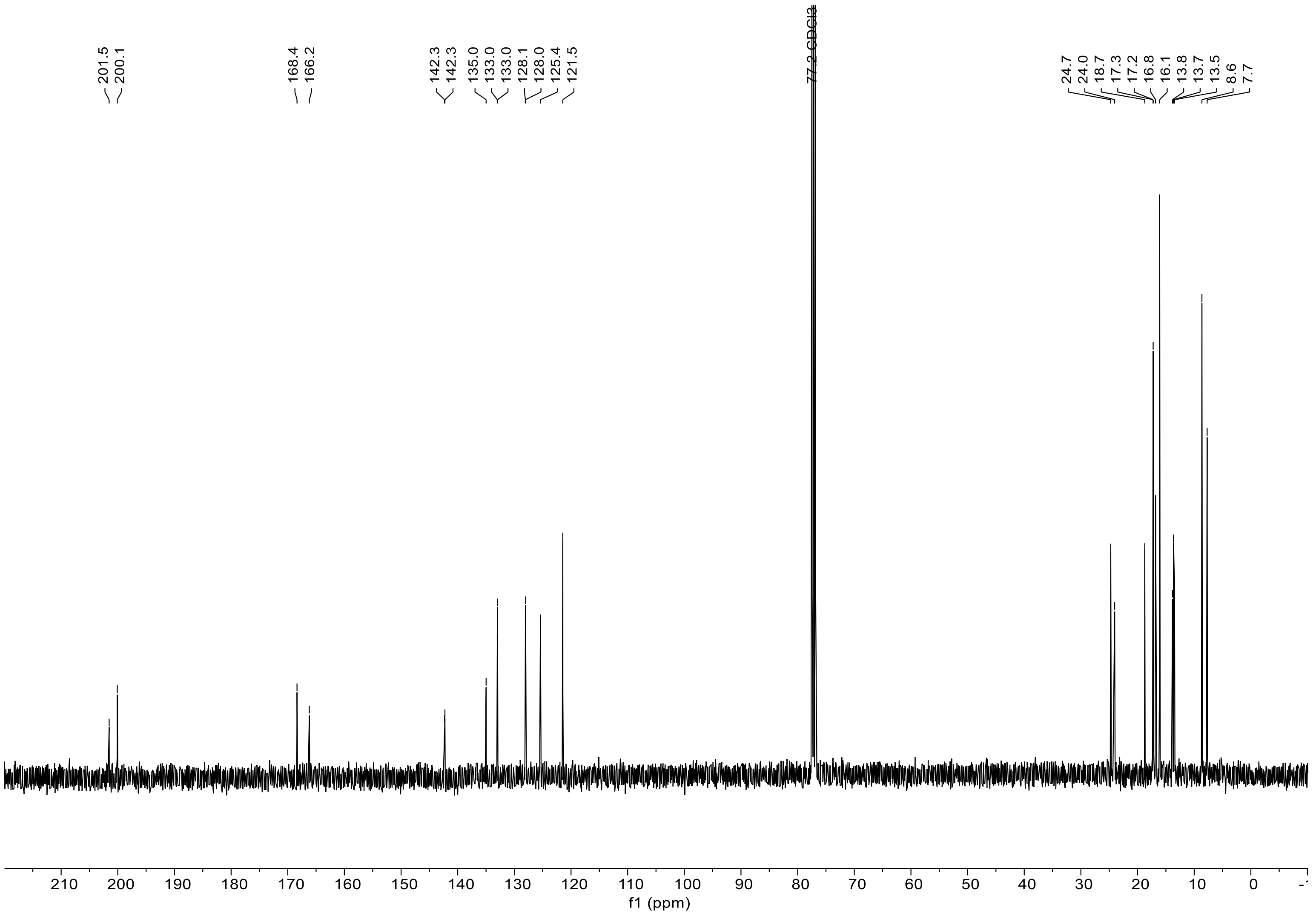

(*E*/*Z*)-3-cyclopropyl-1-(2,3,4,5,6-pentamethylphenyl)pent-3-en-1-one ((*E*/*Z*)-8)
^{1}H NMR ($CDCl_3$, 400 MHz)

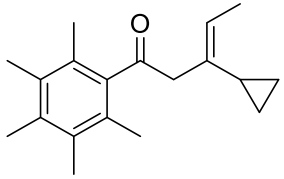

(*Z*)-**8**

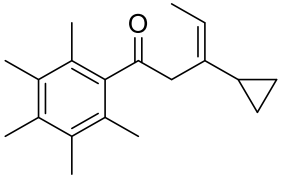

(*E*)-**8**

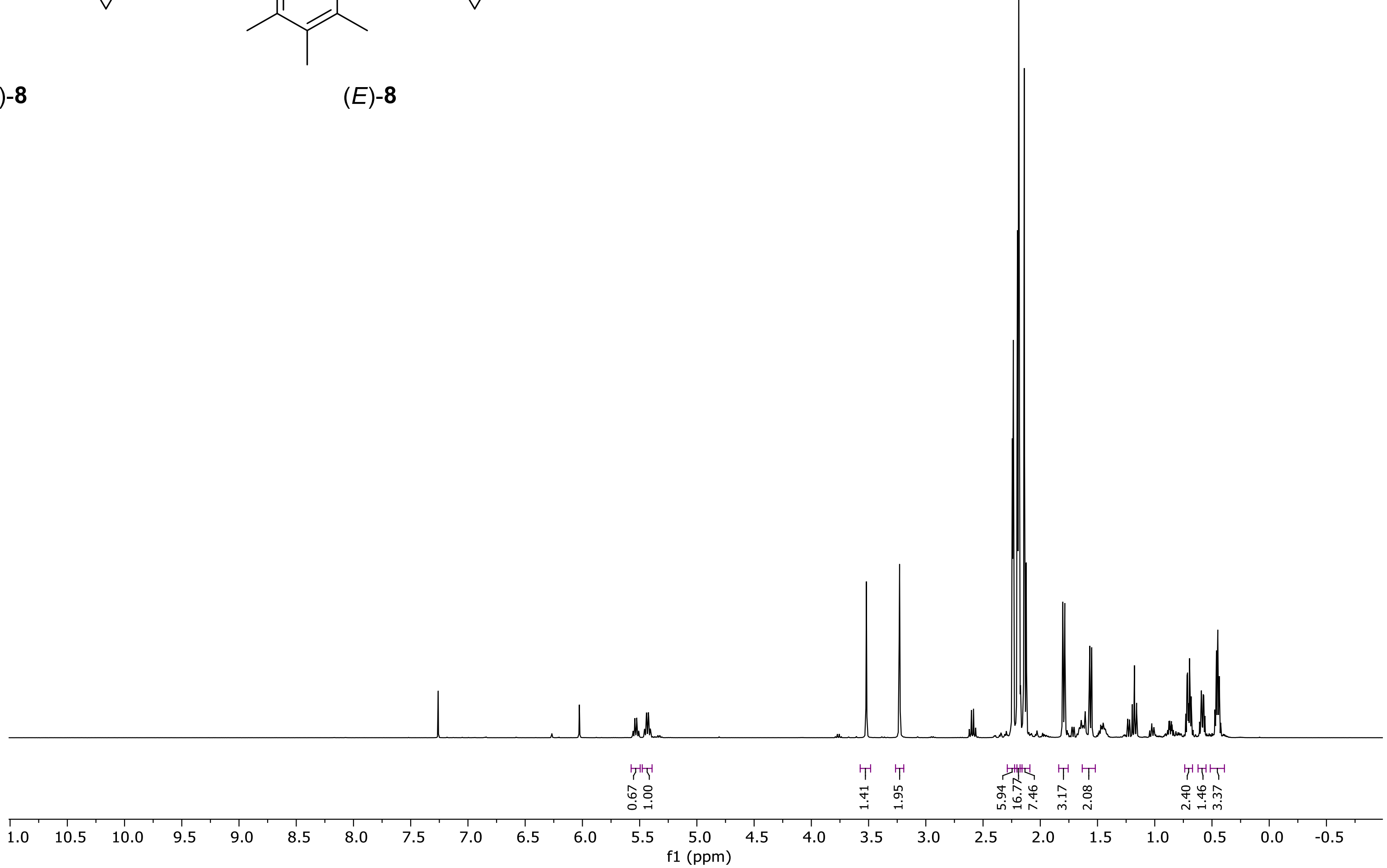

^{13}C NMR ($CDCl_3$, 101 MHz)

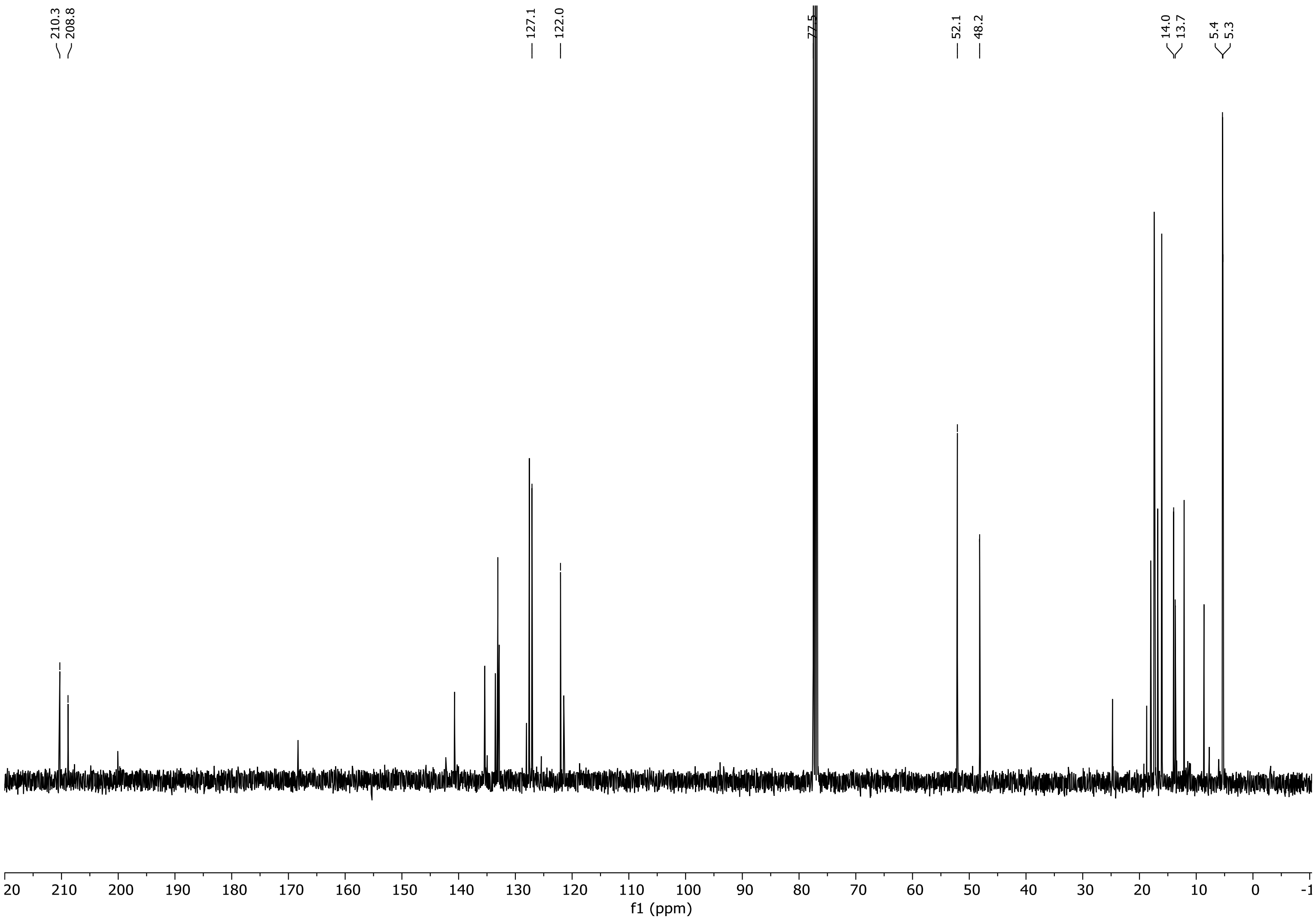

***trans*-(2-Ethylcyclopentyl)(2,3,4,5,6-pentamethylphenyl)methanone (4a)**
^{1}H NMR ($CDCl_3$, 400 MHz)

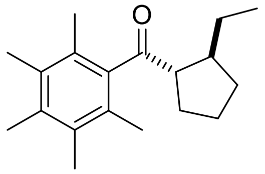

4a

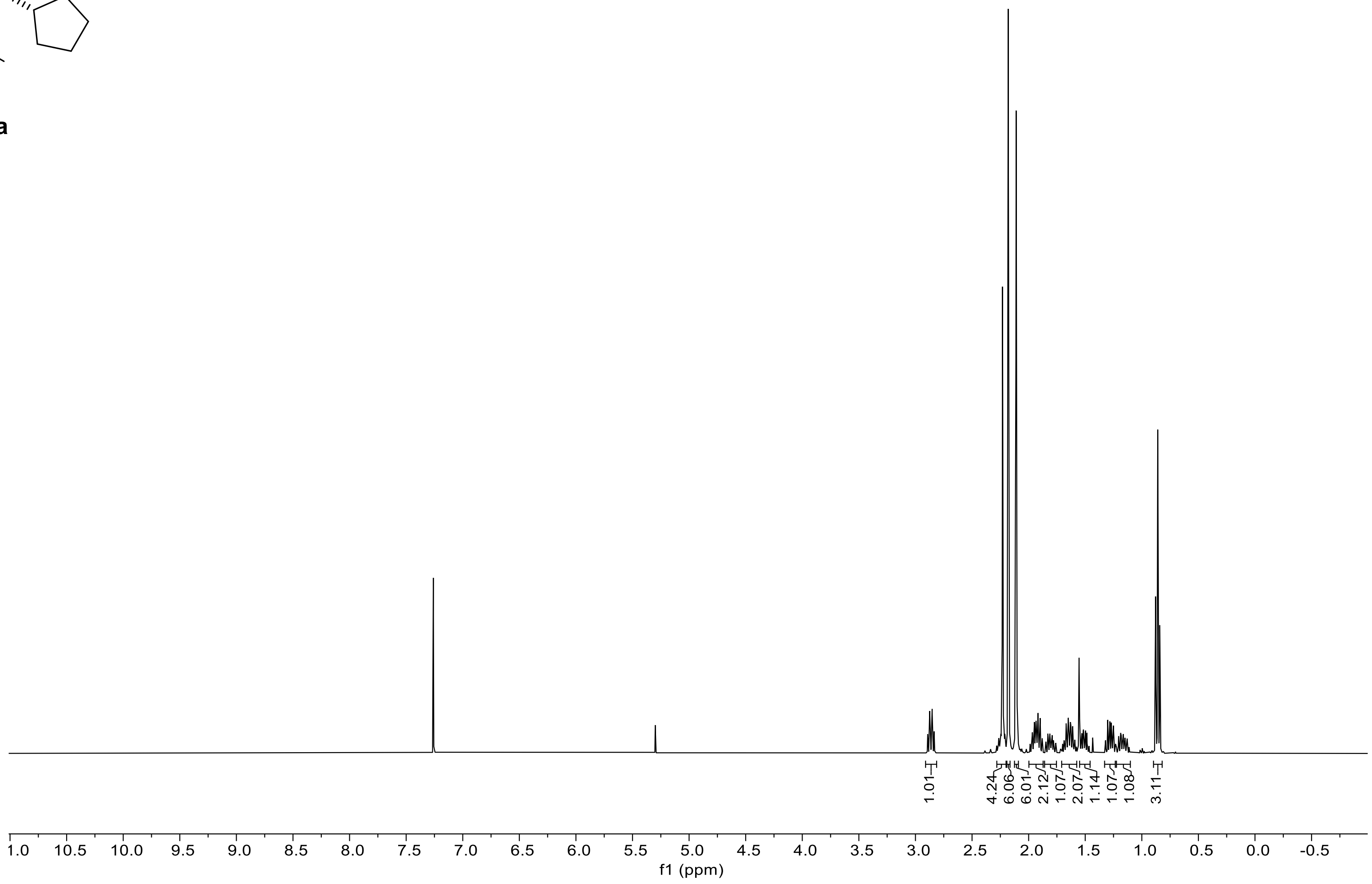

^{13}C NMR ($CDCl_3$, 101 MHz)

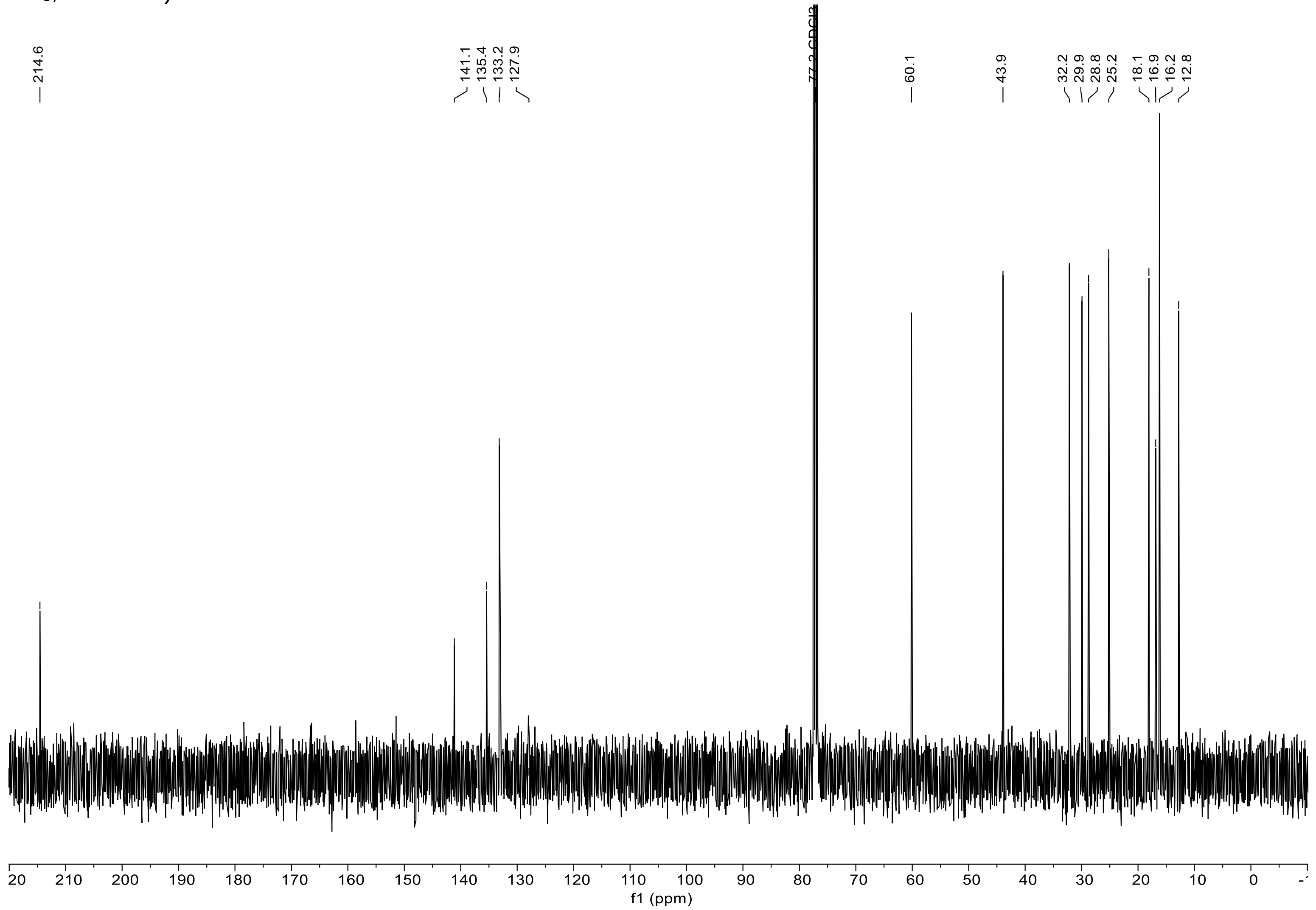

***trans*-(2-Butylcyclopentyl)(2,3,4,5,6-pentamethylphenyl)methanone (4b)**

^{1}H NMR ($CDCl_3$, 400 MHz)

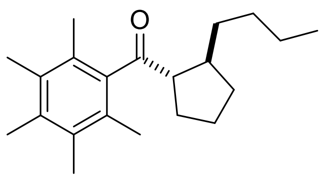

4b

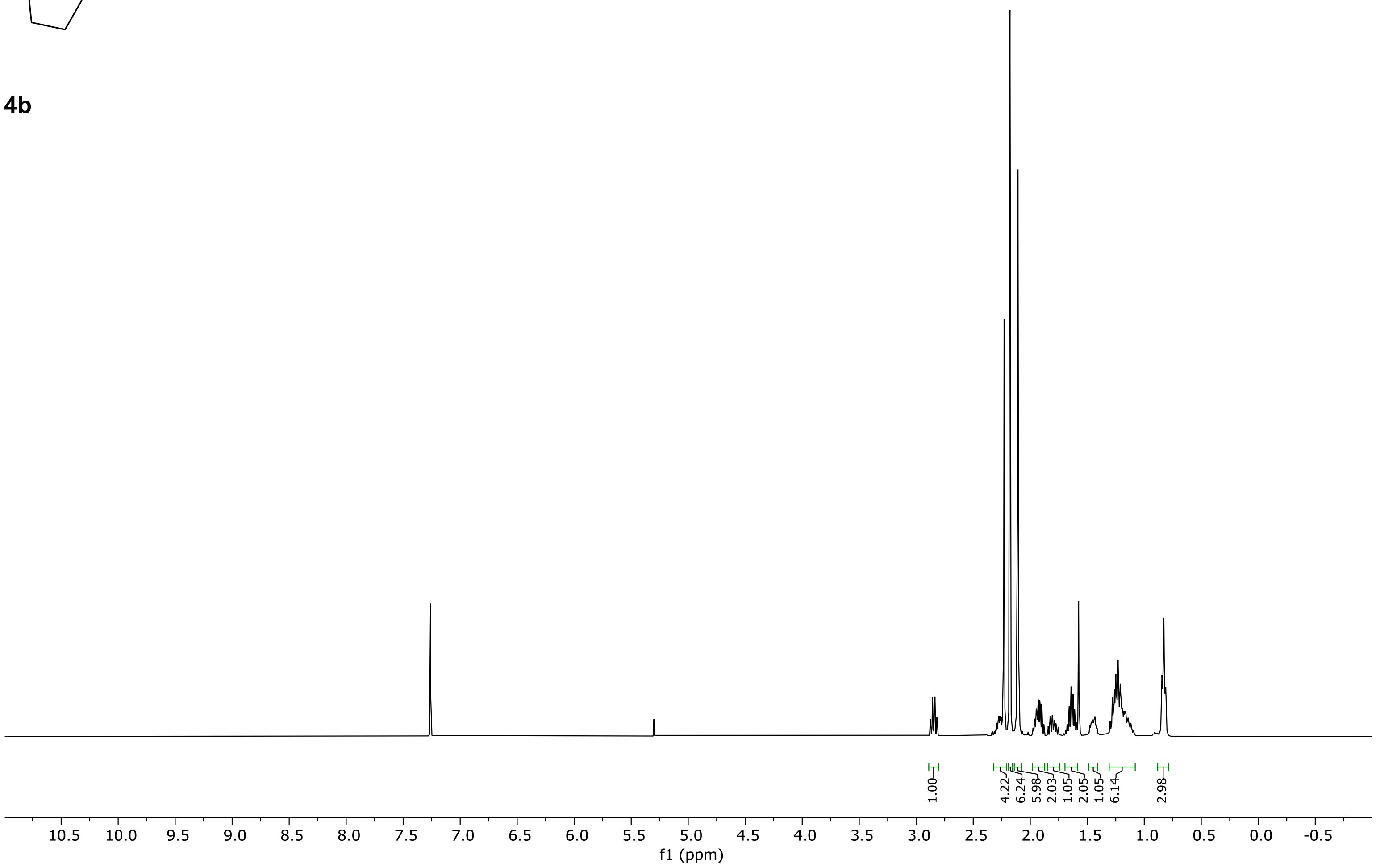

^{13}C NMR ($CDCl_3$, 101 MHz)

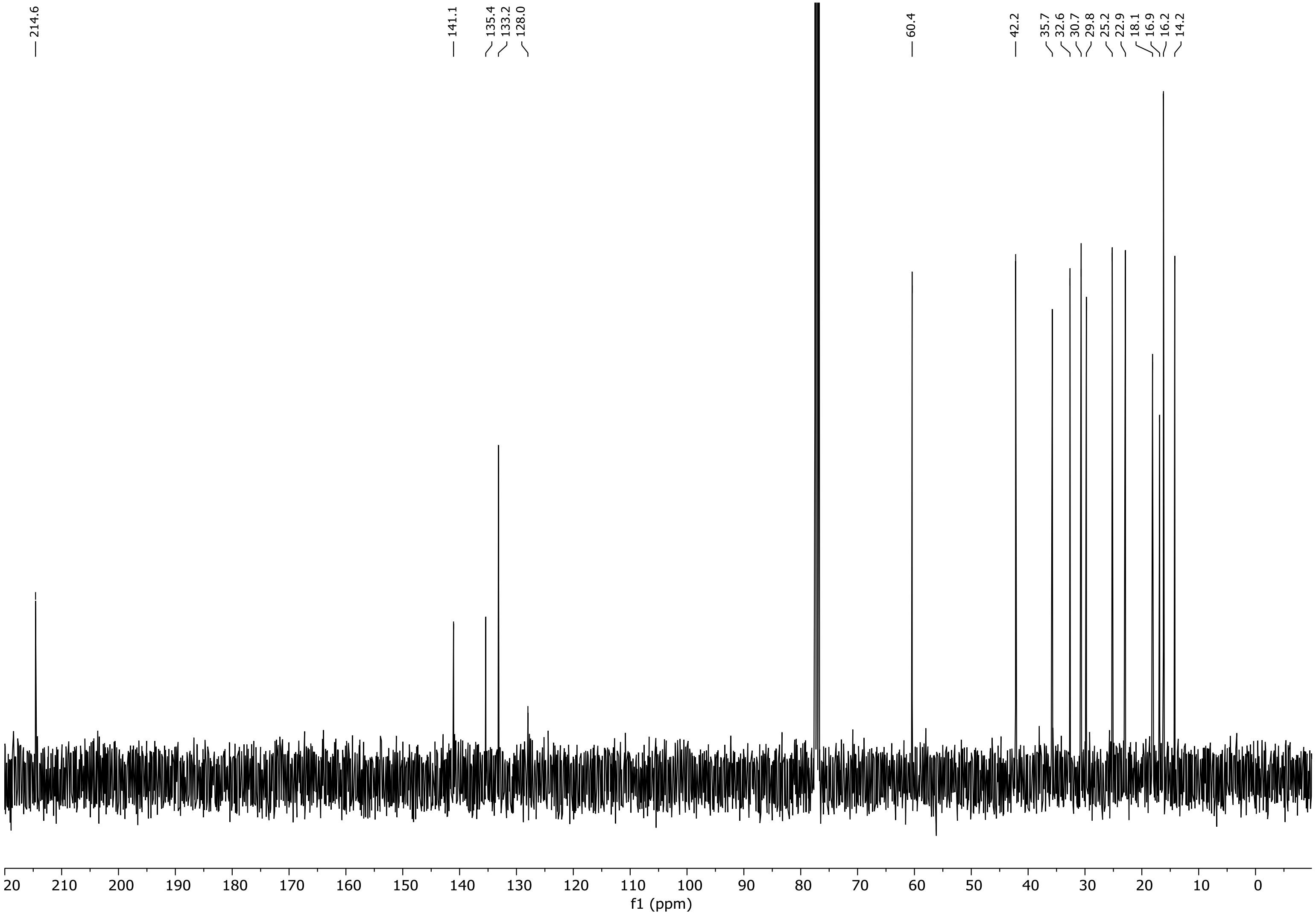

***trans*-2-Cyclobutylcyclopentyl)(2,3,4,5,6-pentamethylphenyl)methanone (4c)**

^{1}H NMR ($CDCl_3$, 400 MHz)

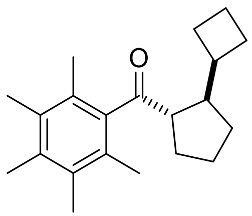

4c

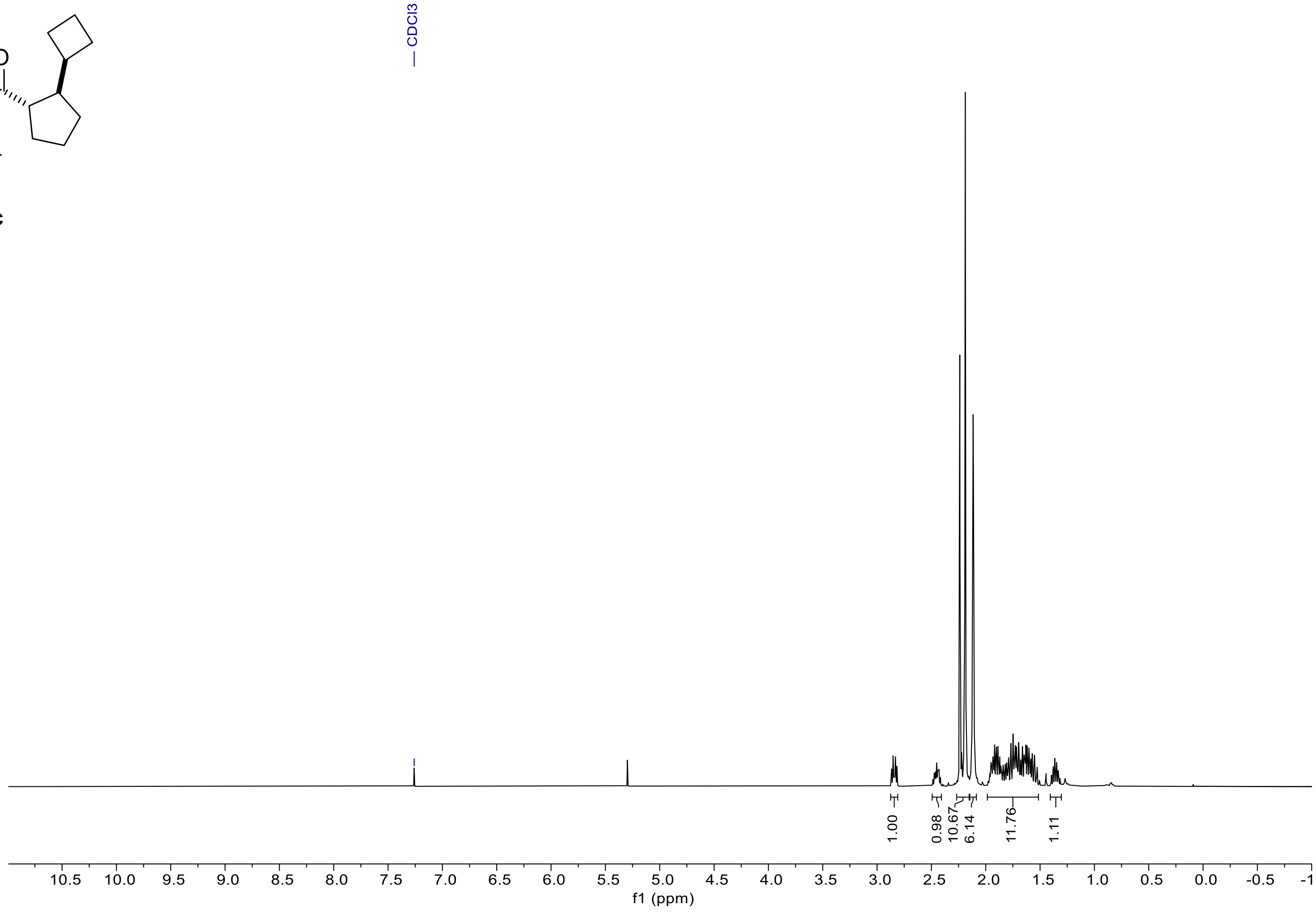

^{13}C NMR ($CDCl_3$, 101 MHz)

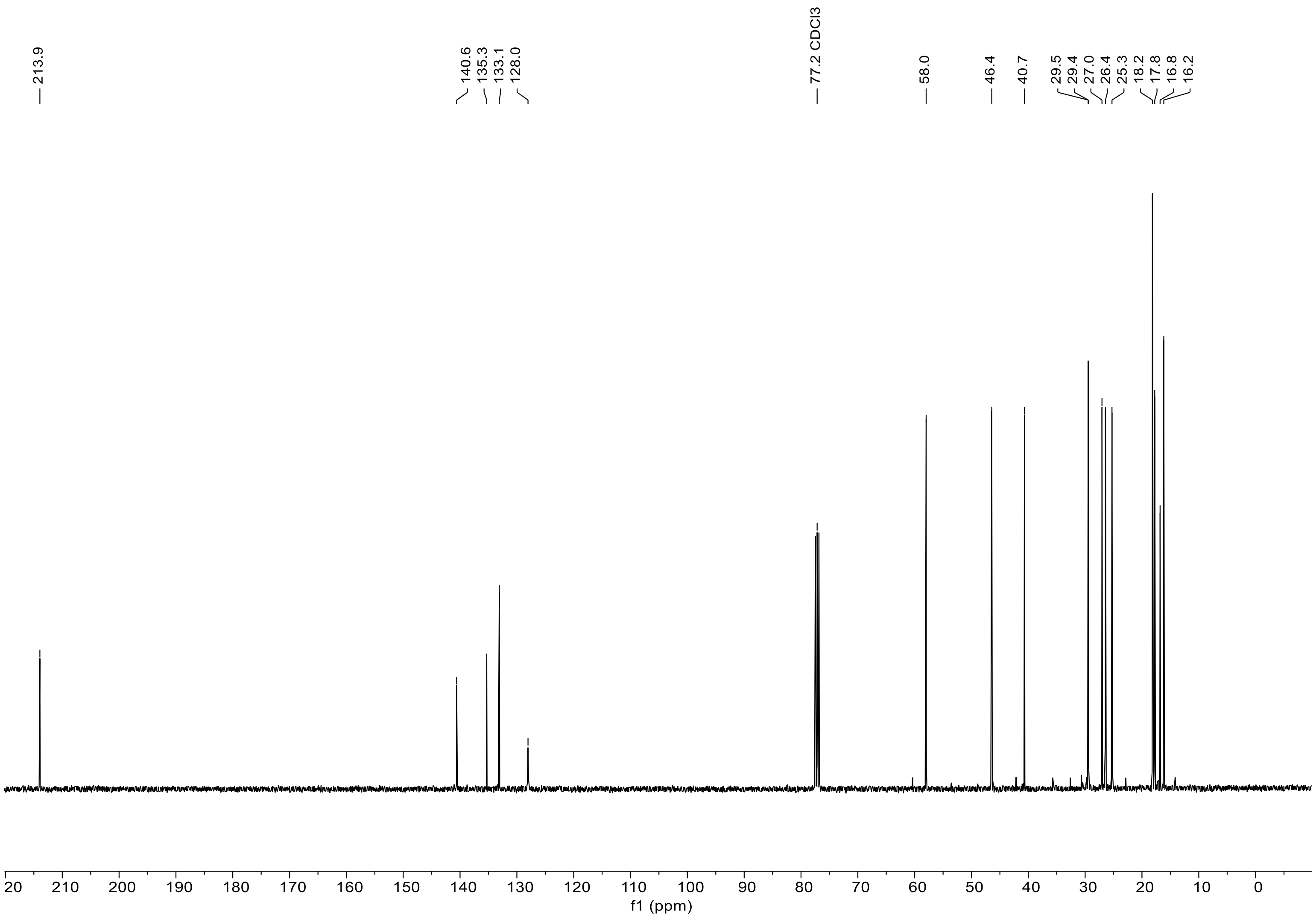

***trans*-(2,3,4,5,6-Pentamethylphenyl)(2-phenethylcyclopentyl)methanone (4d)**
¹H NMR ($CDCl_3$, 400 MHz)

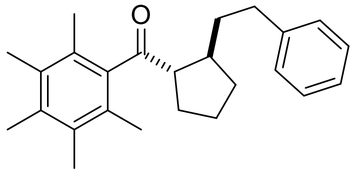

4d

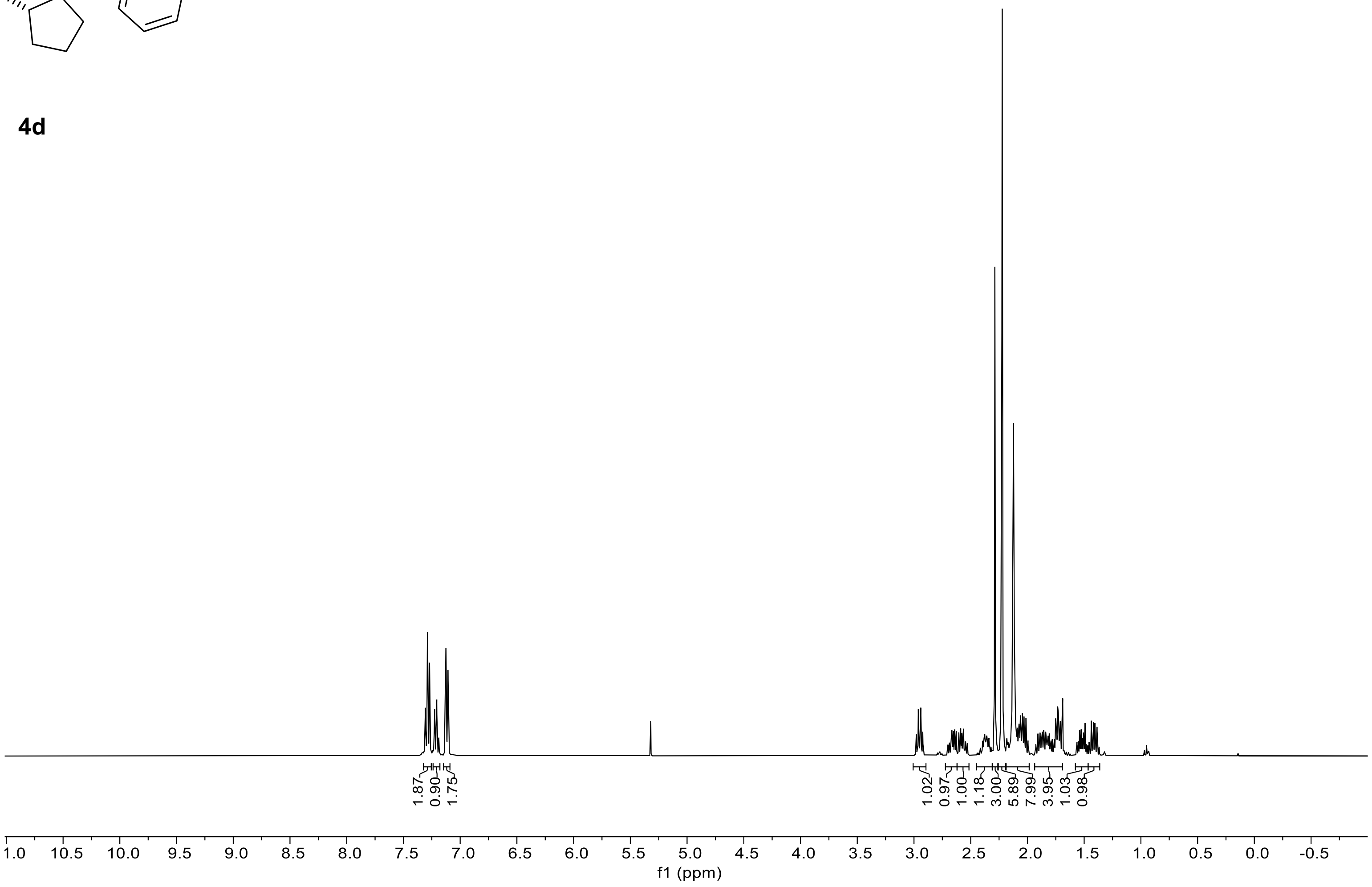

^{13}C NMR ($CDCl_3$, 101 MHz)

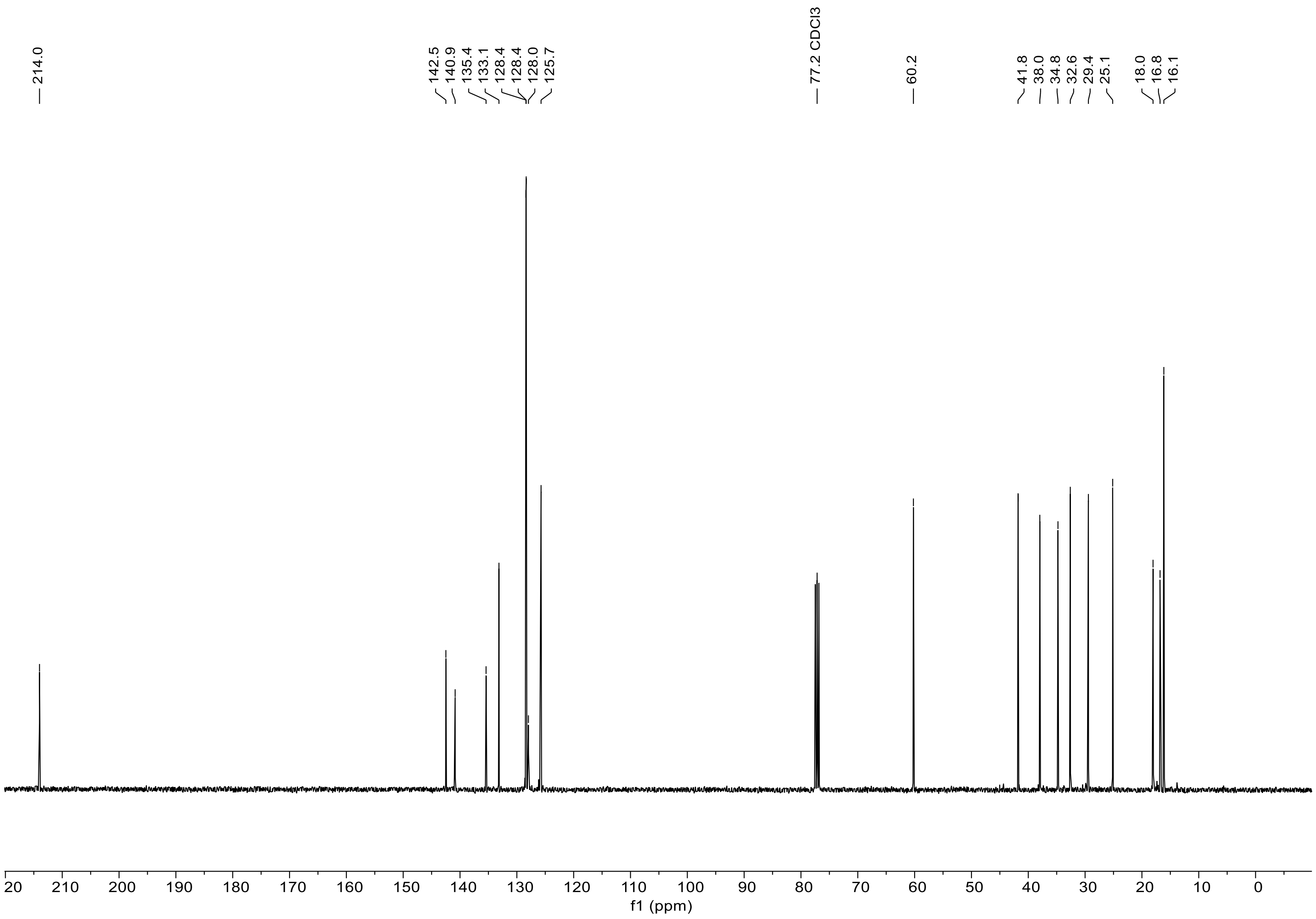

***trans*-(2,3,4,5,6-Pentamethylphenyl)((2-(3-phenylpropyl)cyclopentyl)methanone) (4e)**
^{1}H NMR ($CDCl_3$, 400 MHz)

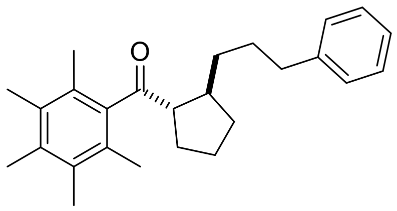

4e

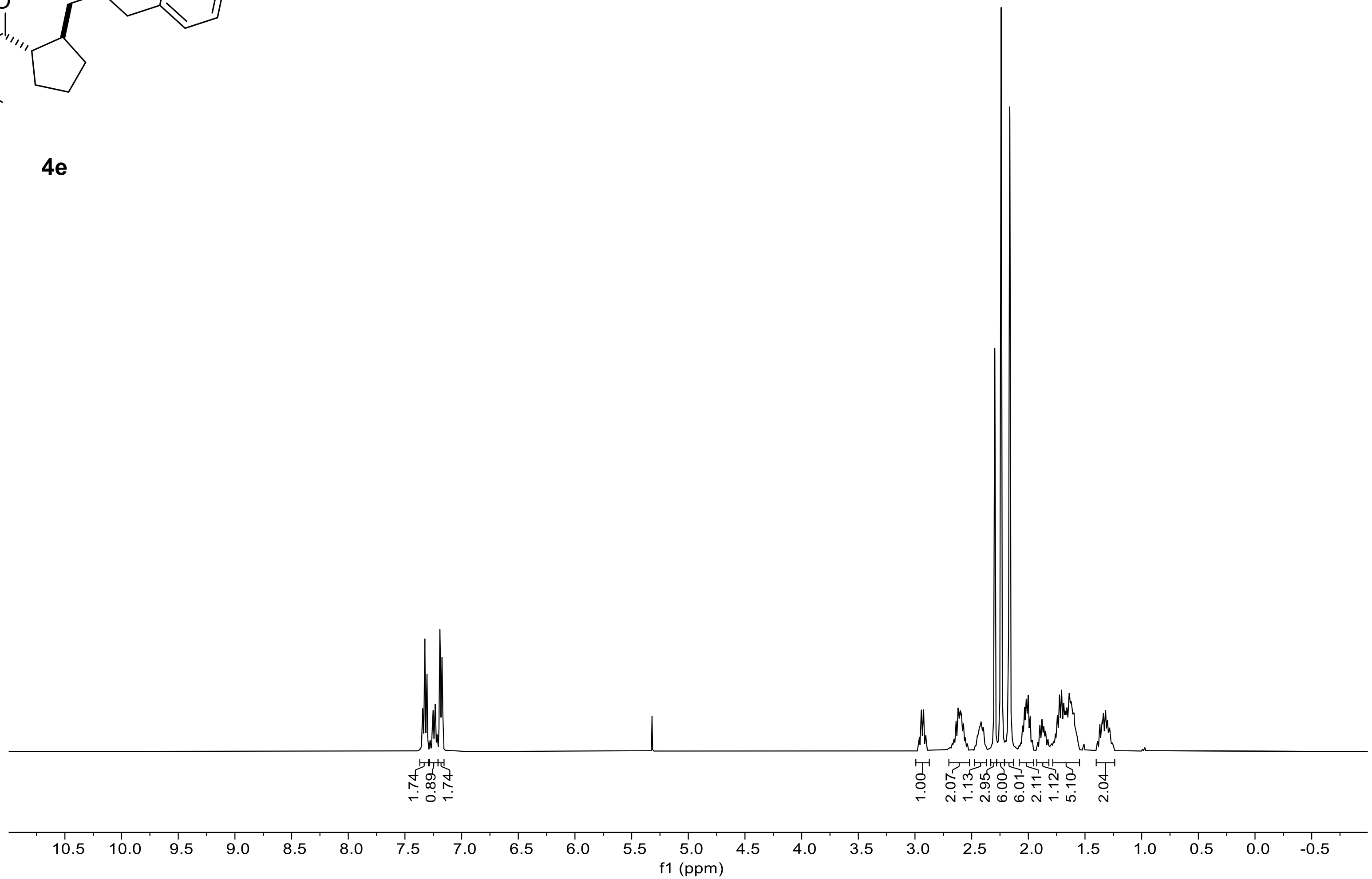

^{13}C NMR ($CDCl_3$, 101 MHz)

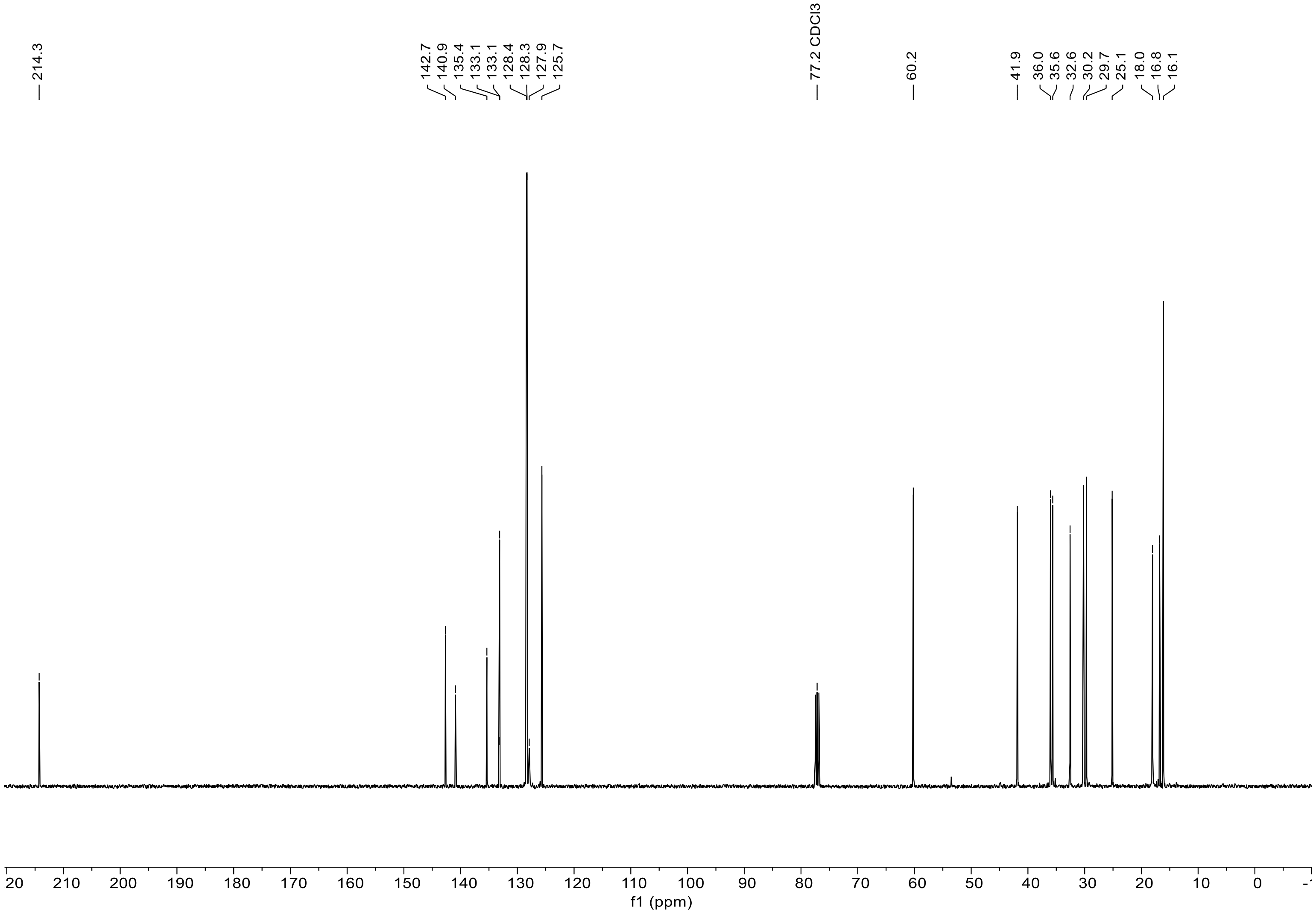

***trans*-(2-(3-(Benzyloxy)propyl)cyclopentyl)(2,3,4,5,6-pentamethylphenyl)methanone (4f)**
^{1}H NMR ($CDCl_3$, 500 MHz)

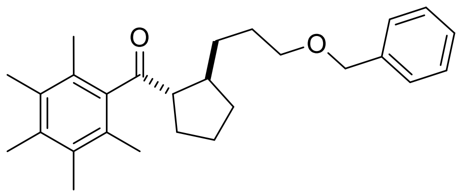

4f

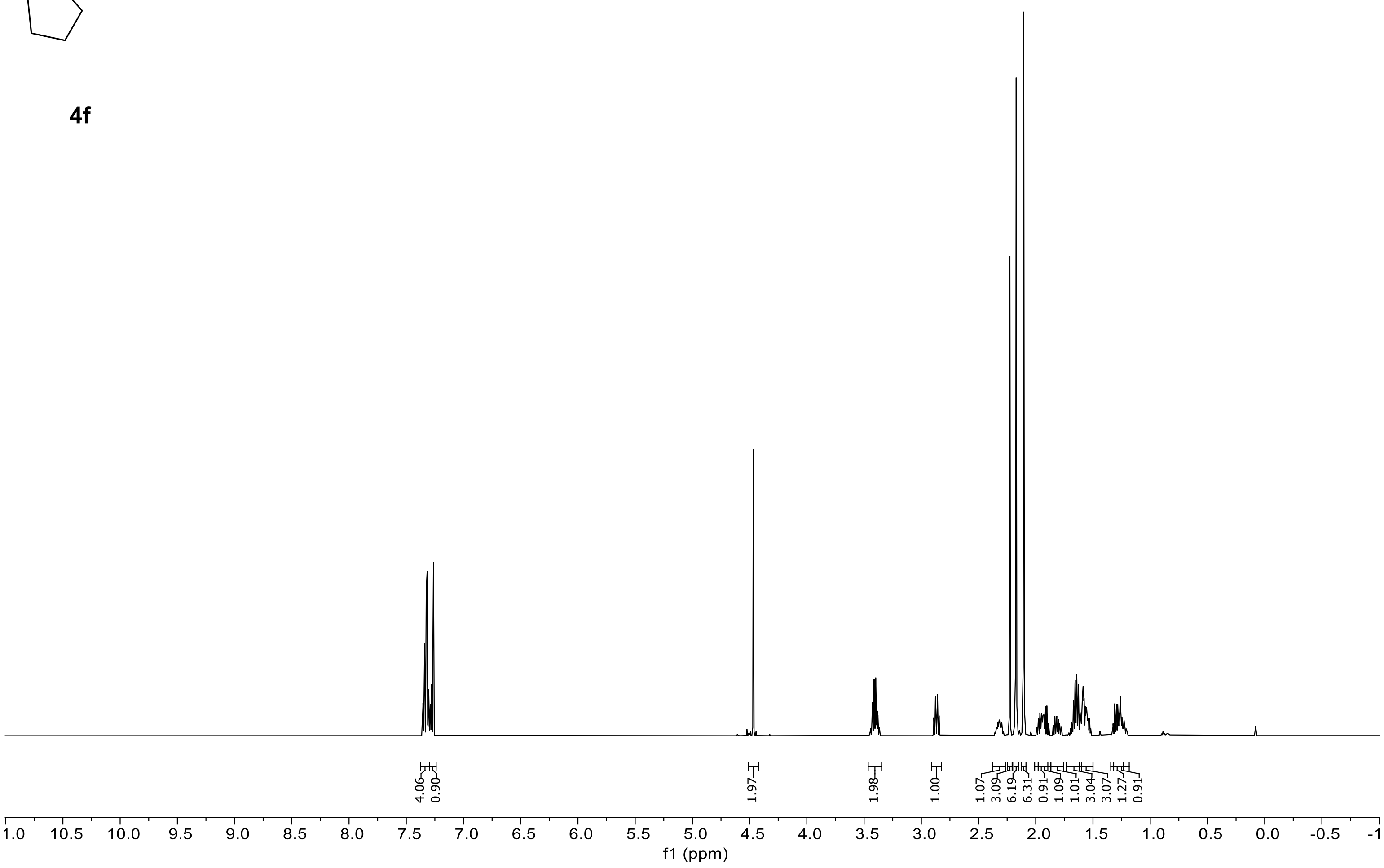

^{13}C NMR ($CDCl_3$, 126 MHz)

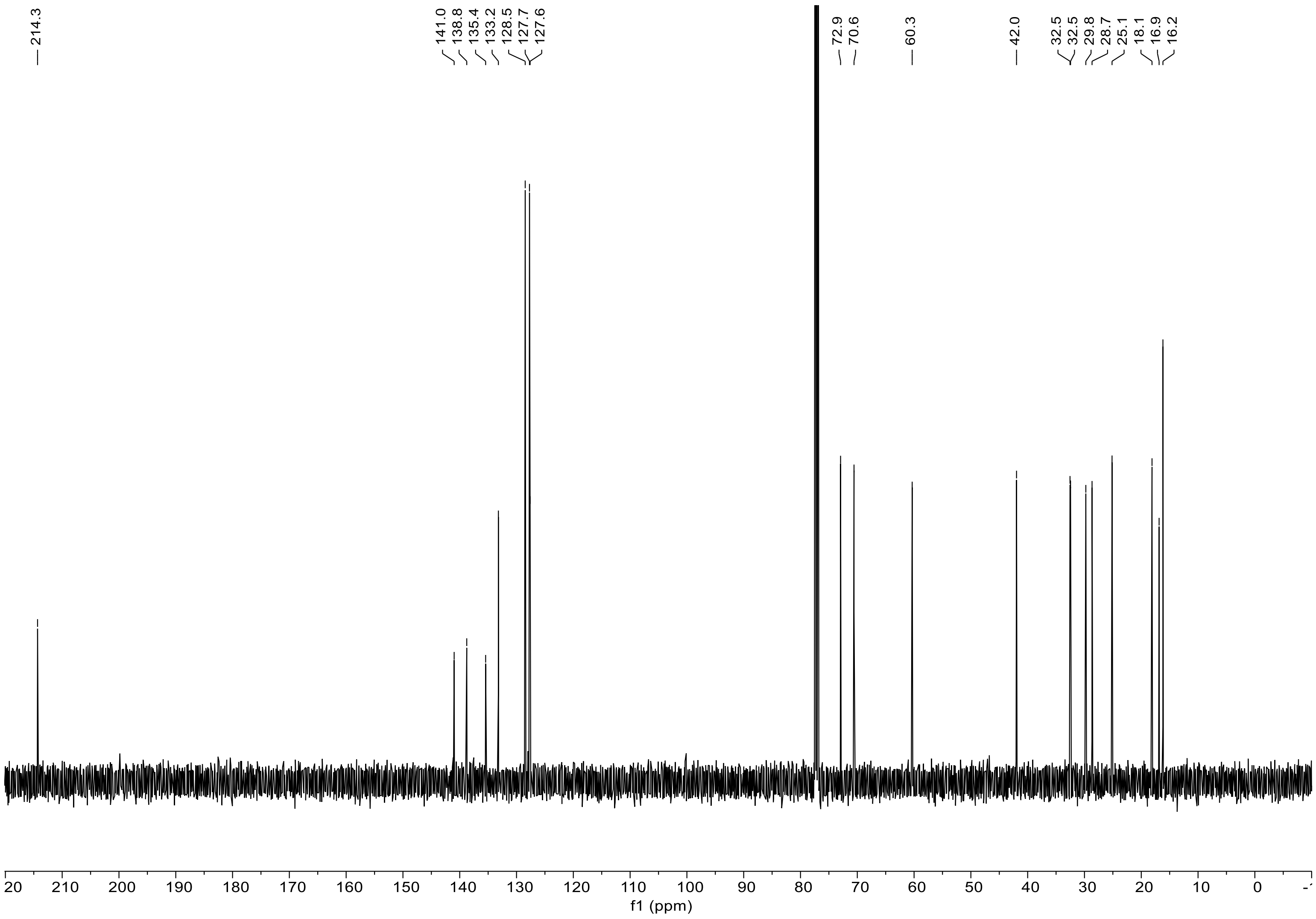

***trans*-(2-(3-(Dibenzylamino)propyl)cyclopentyl)(2,3,4,5,6-pentamethylphenyl)methanone (4g)**
^{1}H NMR ($CDCl_3$, 500 MHz)

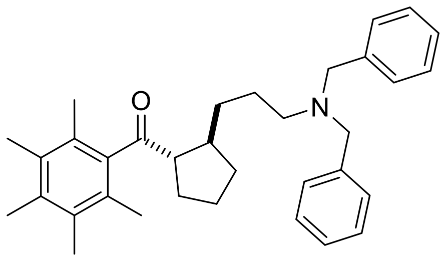

4g

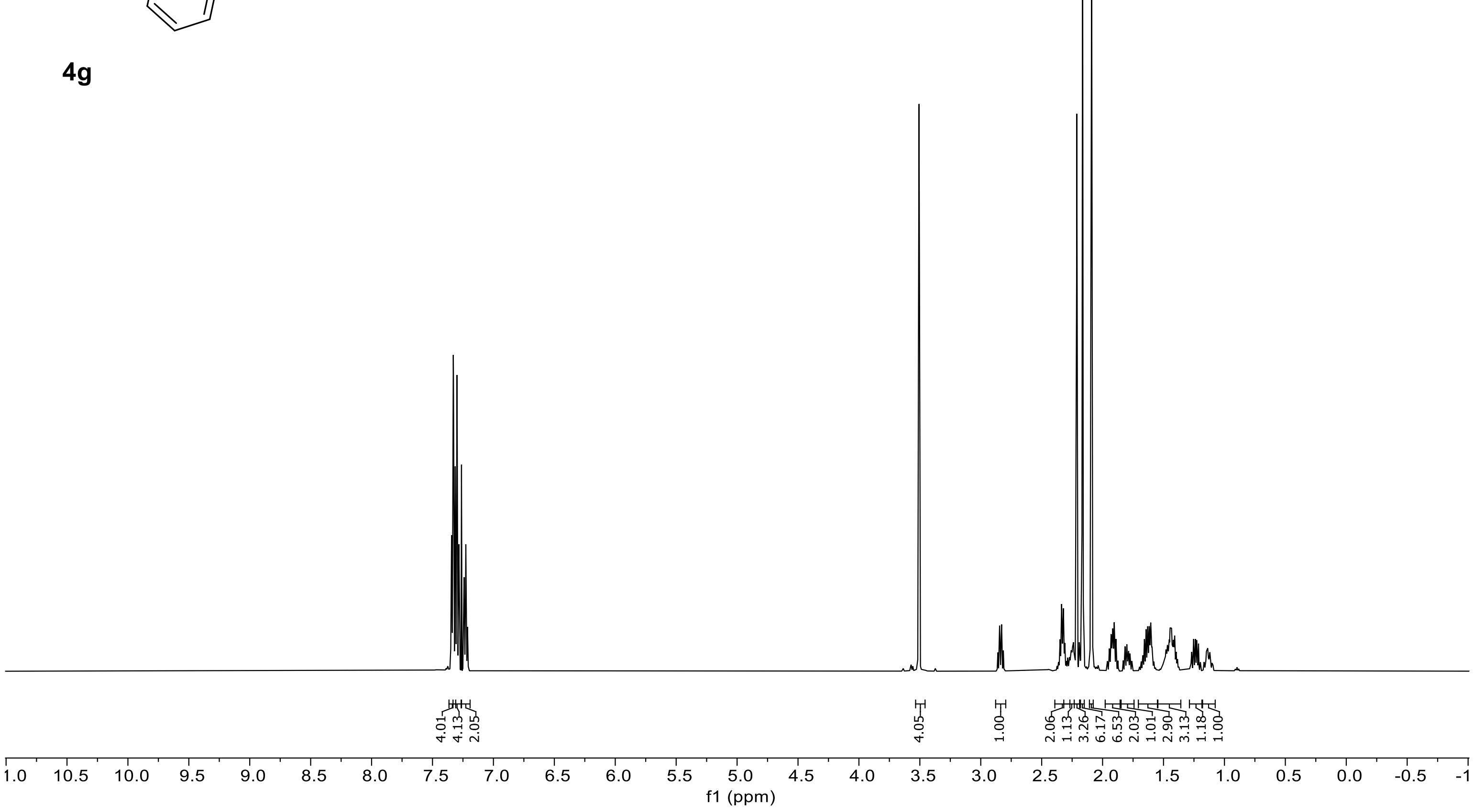

^{13}C NMR ($CDCl_3$, 126 MHz)

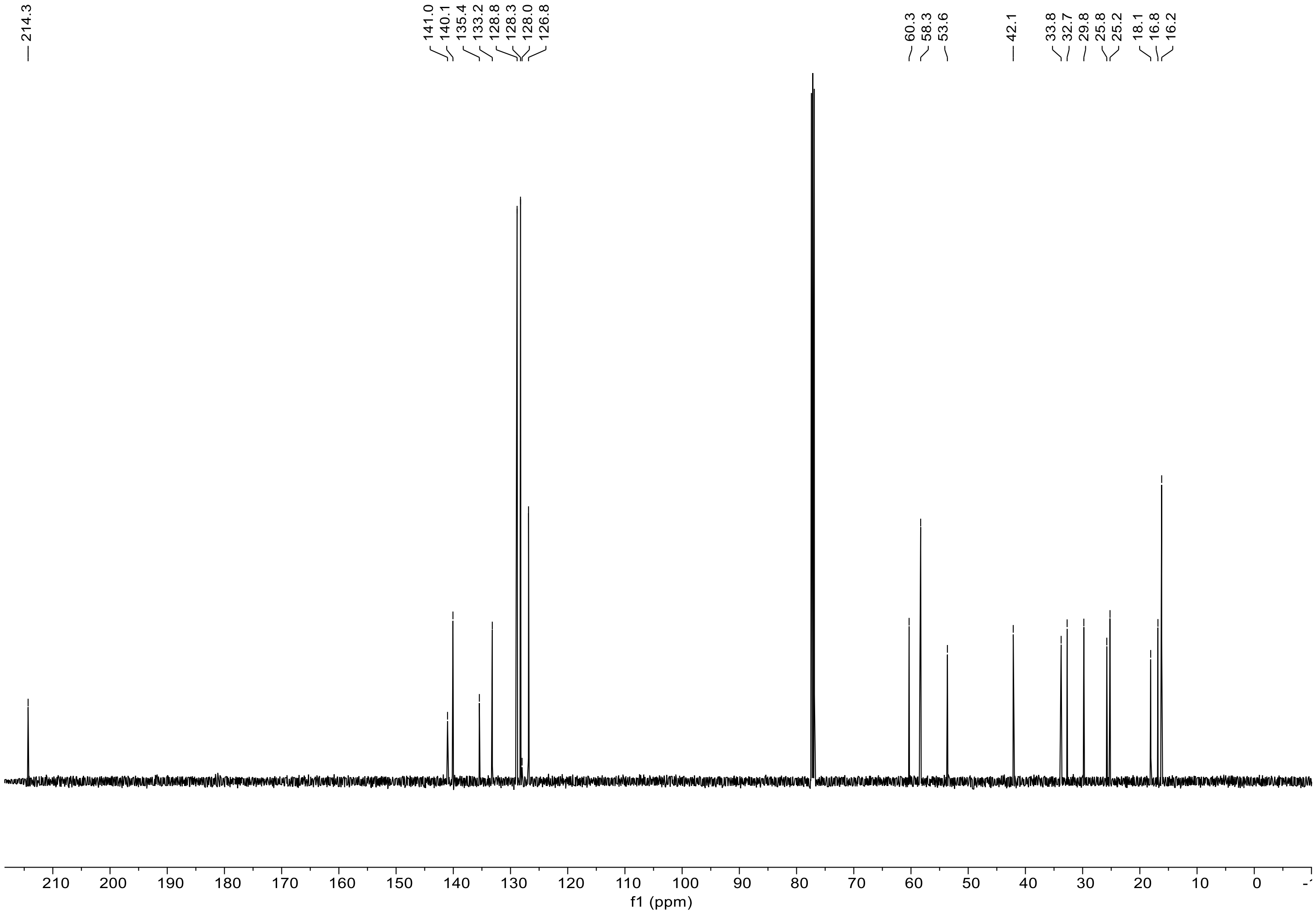

***trans*-(2-(3-(Methylthio)propyl)cyclopentyl)(2,3,4,5,6-pentamethylphenyl)methanone (4h)**

^{1}H NMR ($CDCl_3$, 400 MHz)

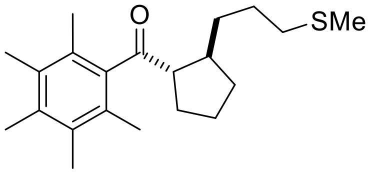

4h

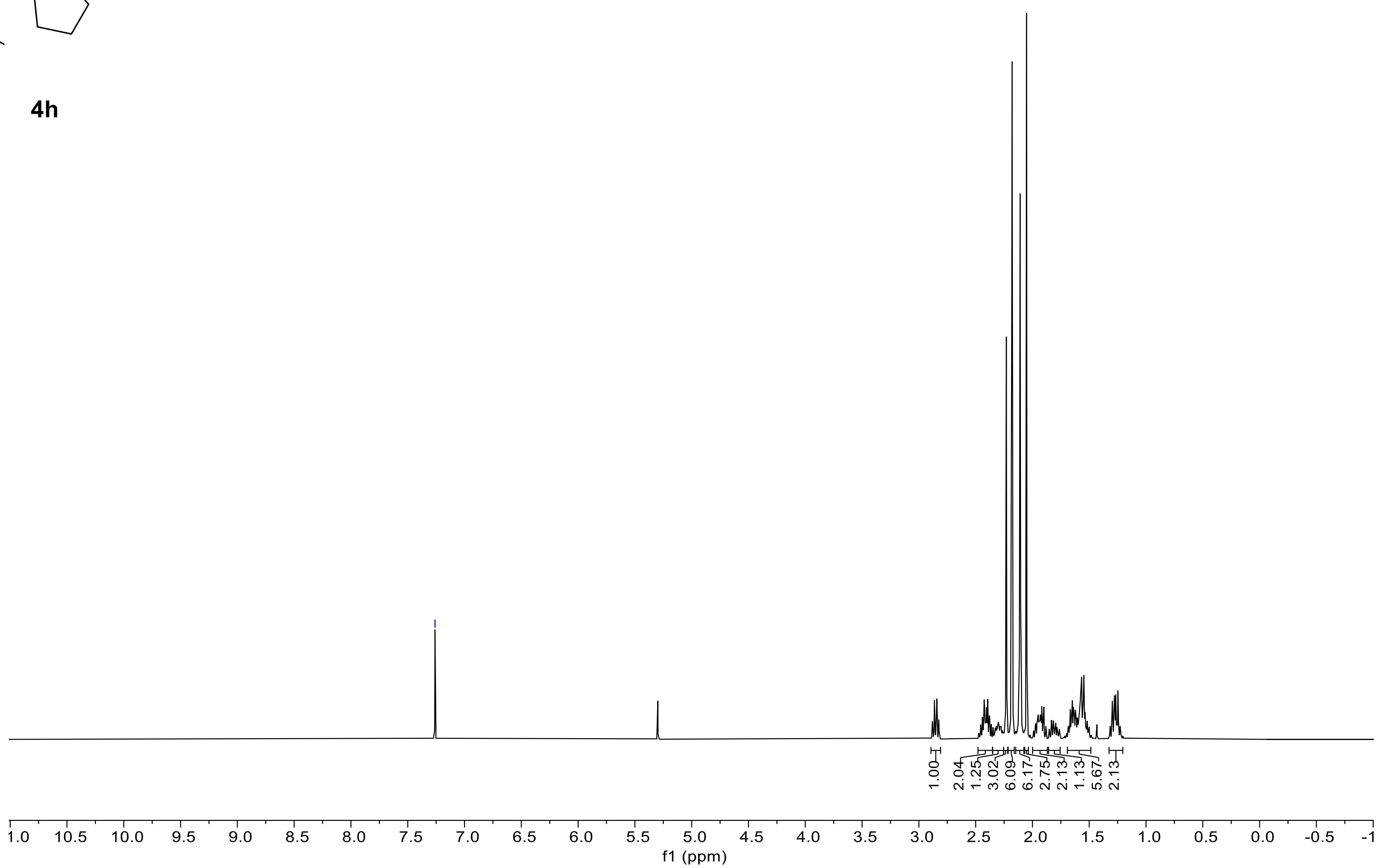

^{13}C NMR ($CDCl_3$, 101 MHz)

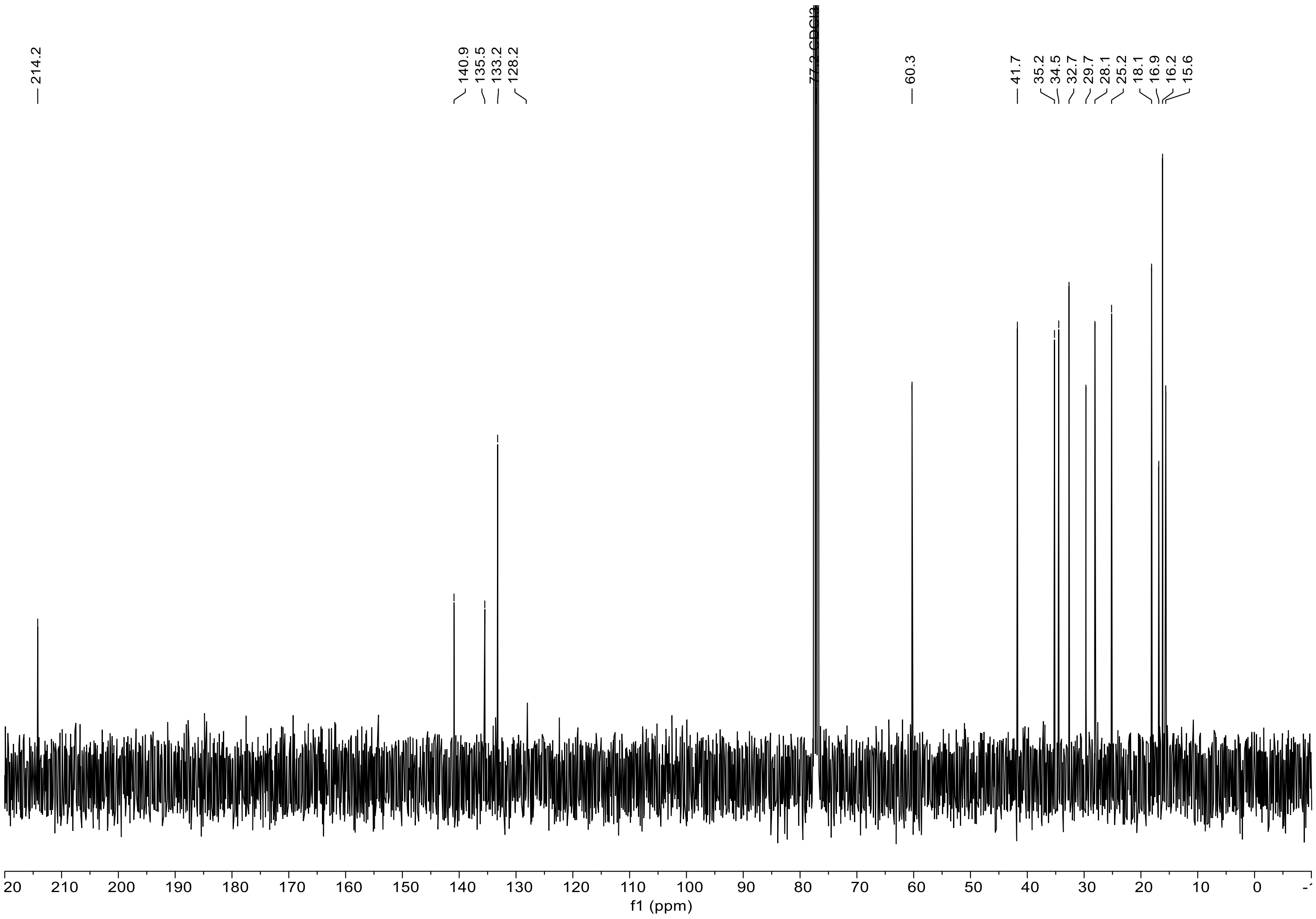

***trans*-(2,3,4,5,6-Pentamethylphenyl)(2-(2-(pyridin-2-yl)ethyl)cyclopentyl)methanone (4i)**
^{1}H NMR ($CDCl_3$, 400 MHz)

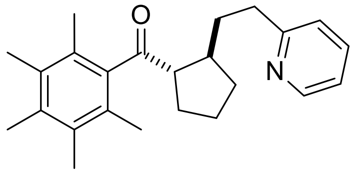

4i

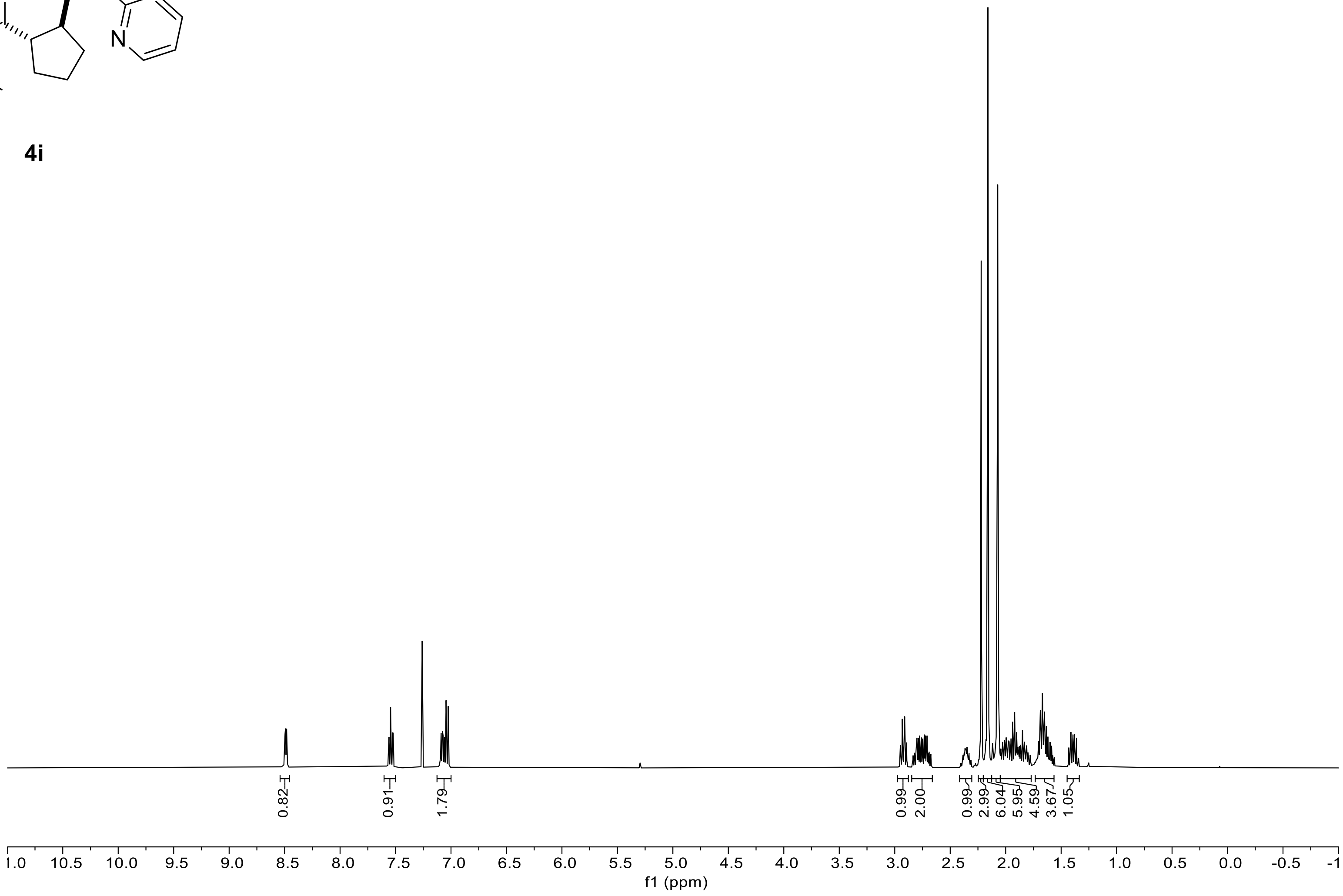

^{13}C NMR ($CDCl_3$, 101 MHz)

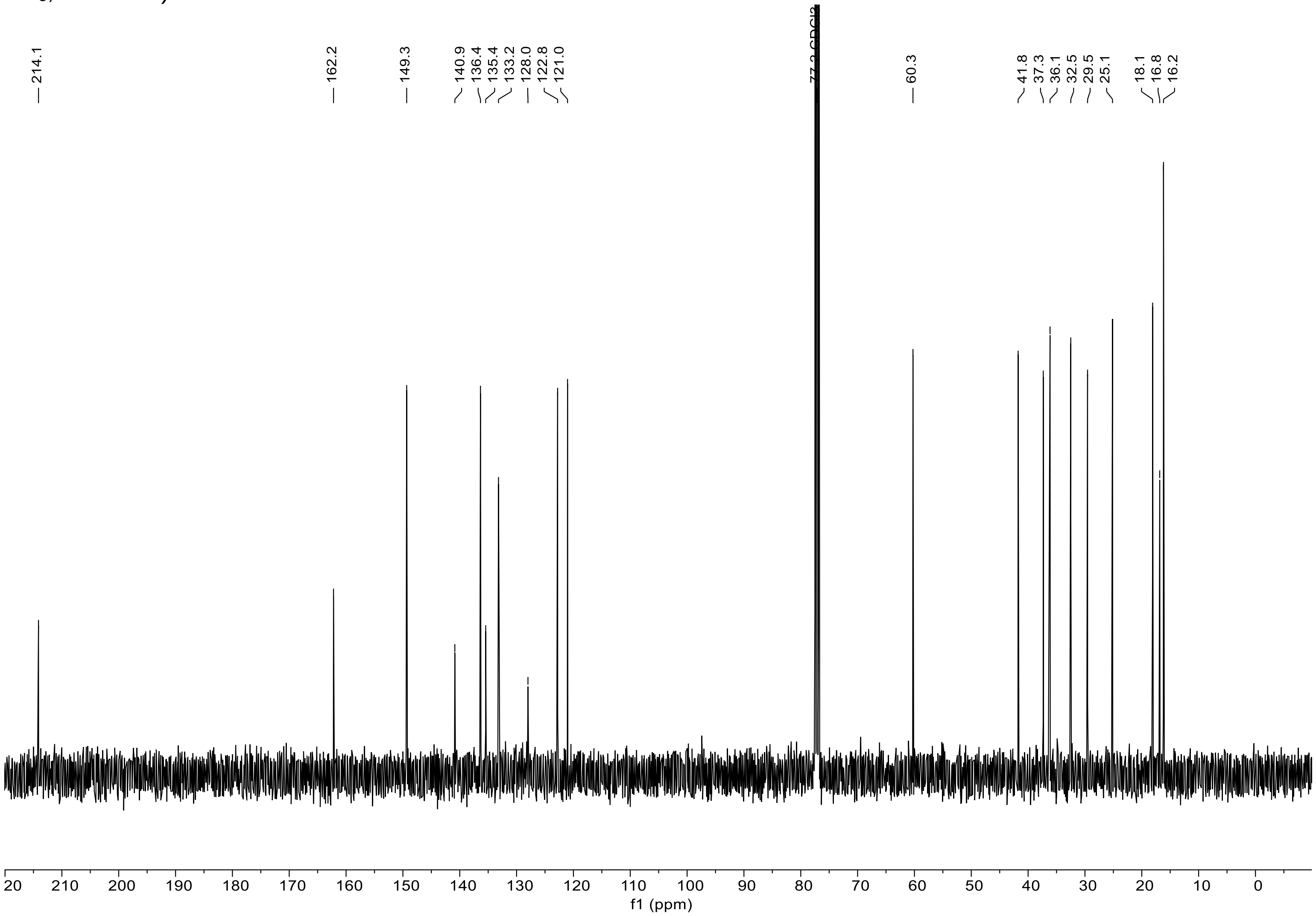

(2-Methyl-5-phenylcyclopentyl)(2,3,4,5,6-pentamethylphenyl)methanone (4j and 4j')
^{1}H NMR ($CDCl_3$, 400 MHz)

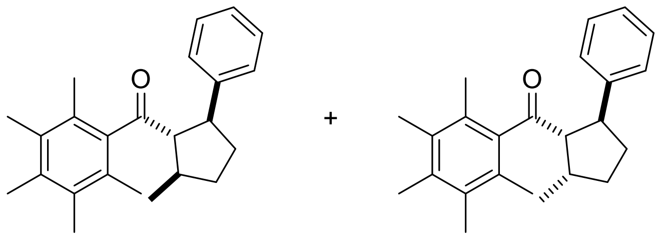

4j 77:23 **4j'**

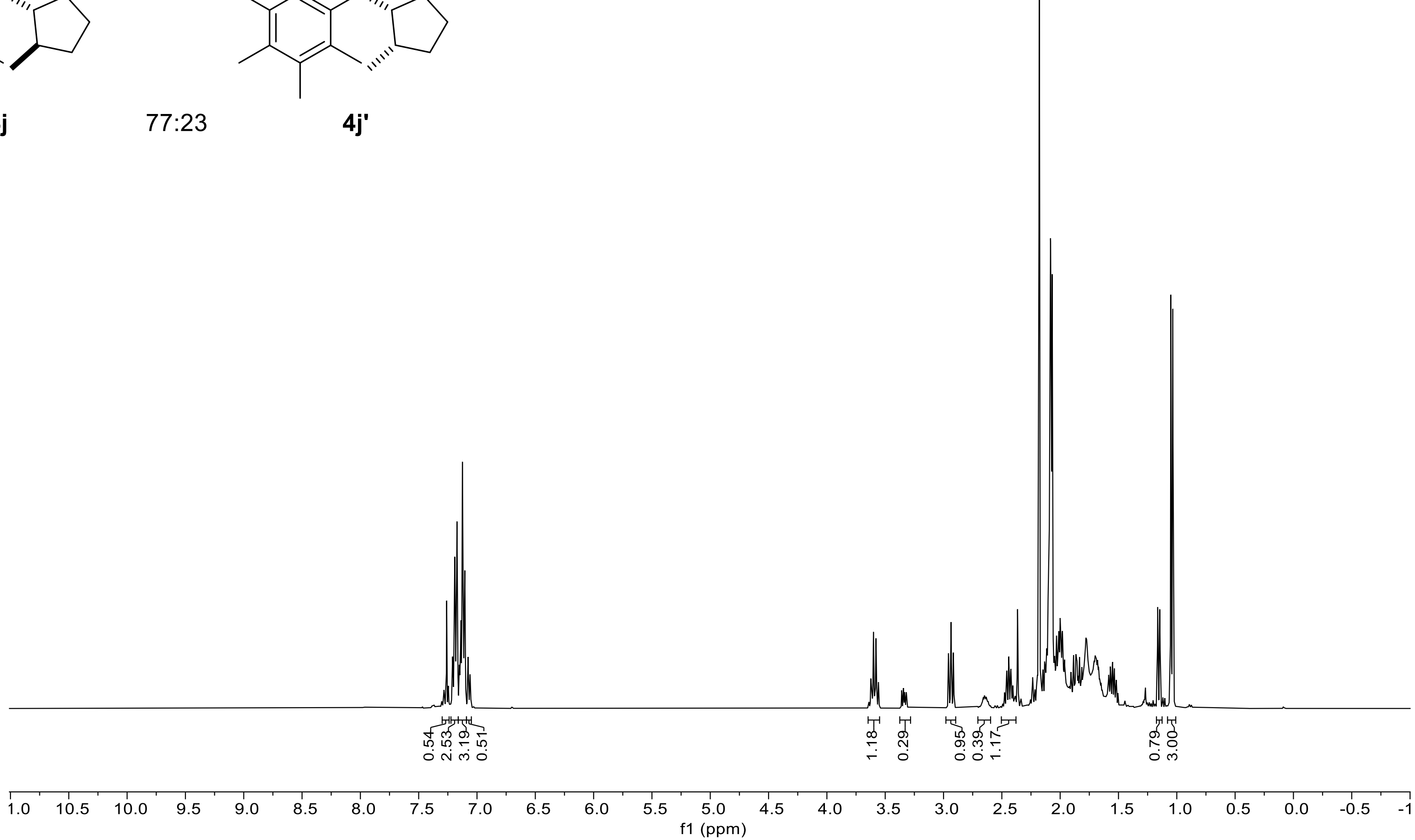

^{13}C NMR ($CDCl_3$, 101 MHz)

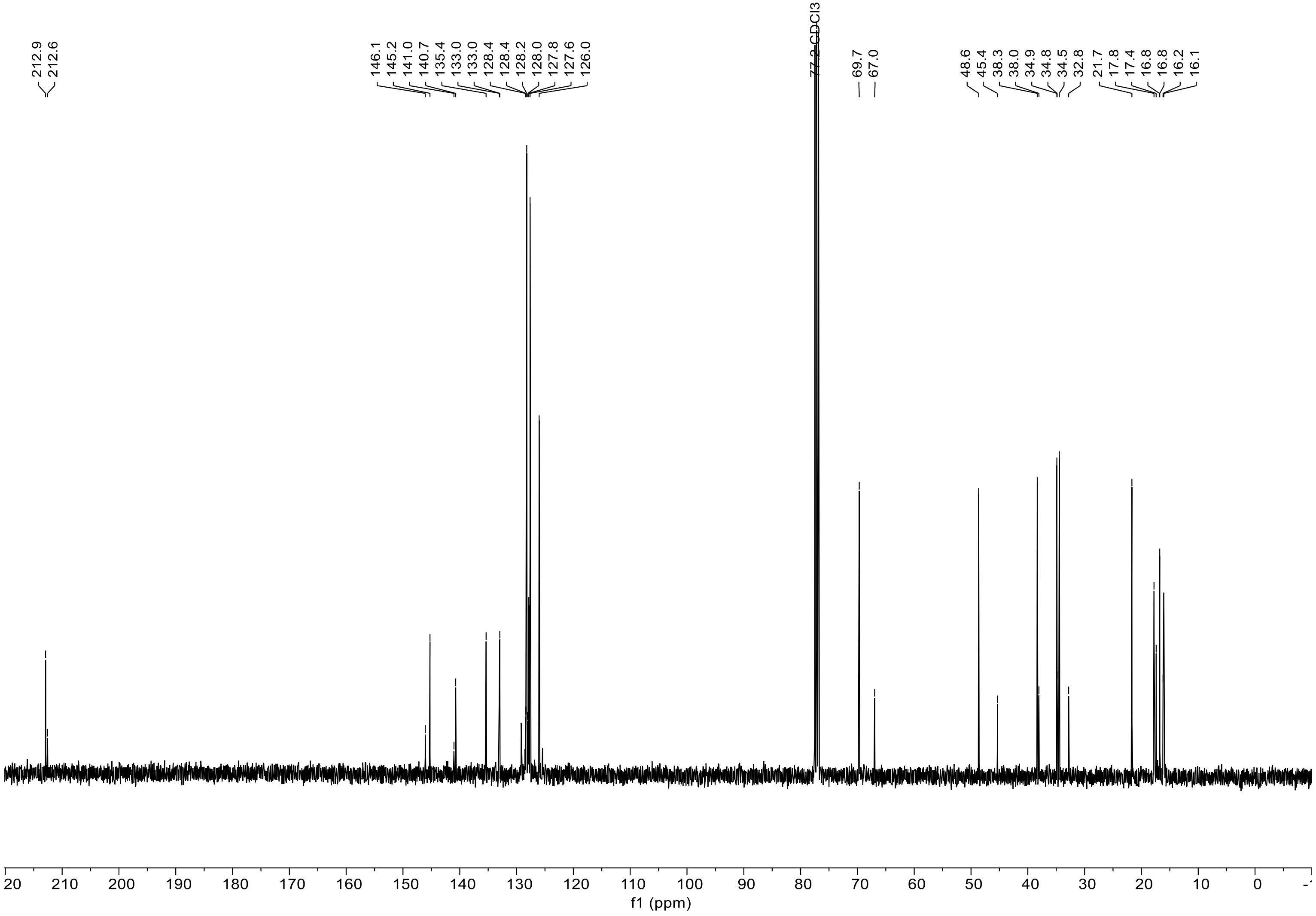

***trans*-(1-Methyloctahydro-1H-inden-2-yl)(2,3,4,5,6-pentamethylphenyl)methanone (4l)**
^{1}H NMR ($CDCl_3$, 500 MHz)

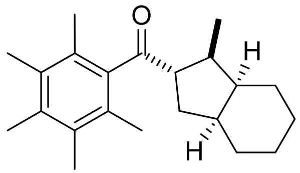

4l

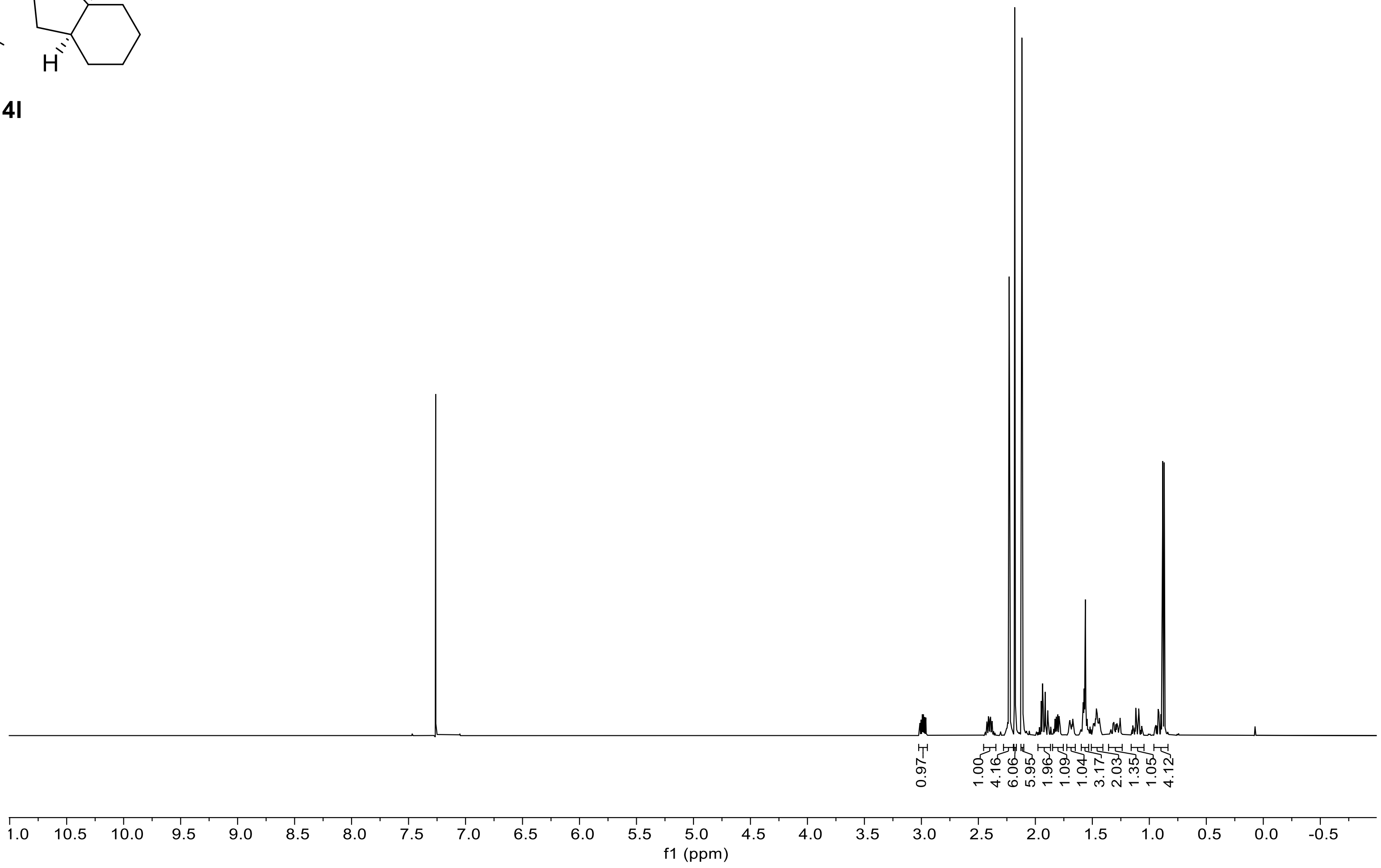

^{13}C NMR ($CDCl_3$, 126 MHz)

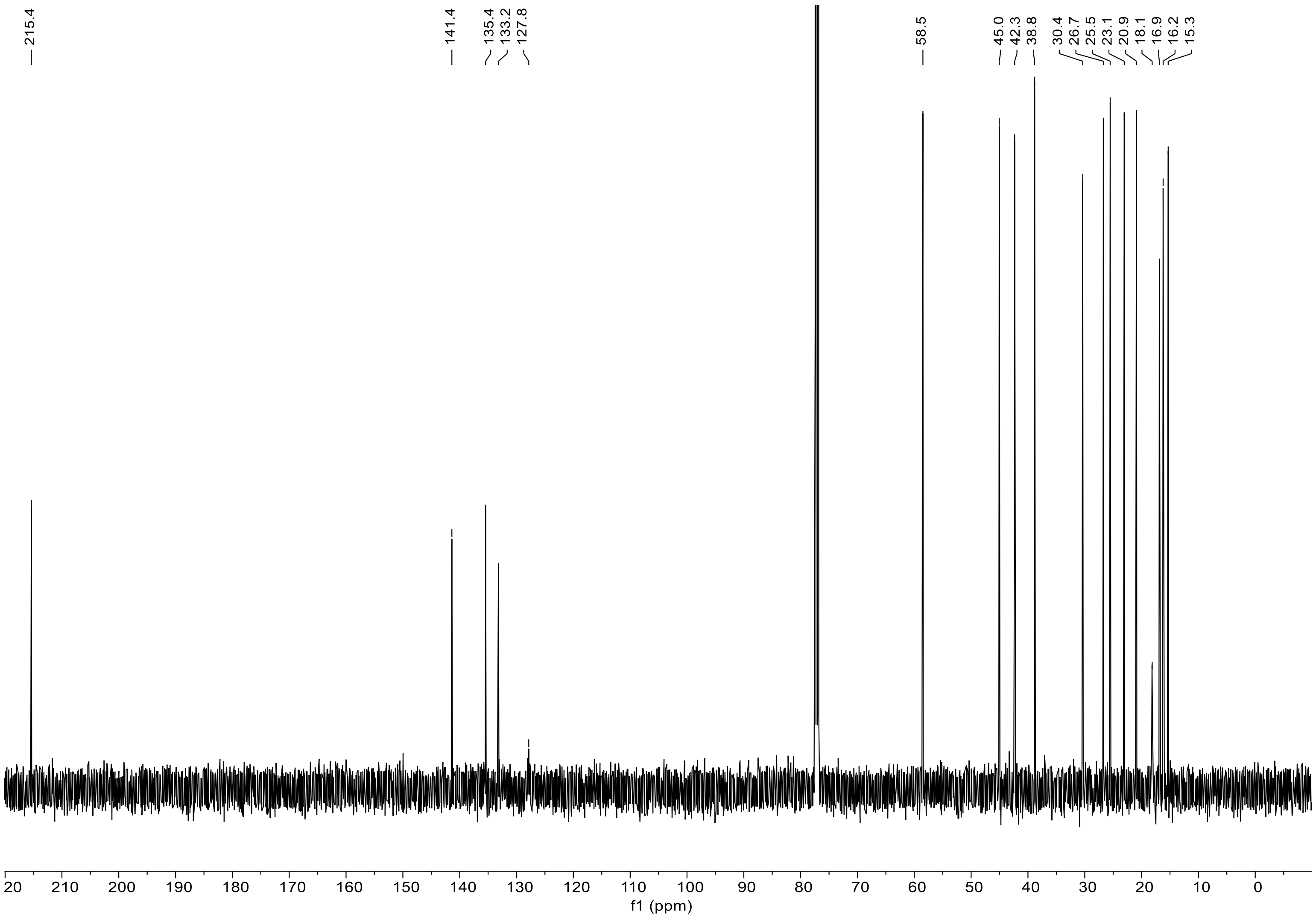

(Octahydro-1H-inden-1-yl)(2,3,4,5,6-pentamethylphenyl)methanone (4m)
^{1}H NMR ($CDCl_3$, 500 MHz)

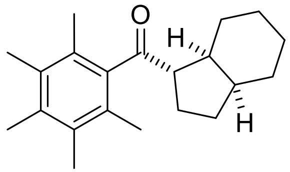

4m

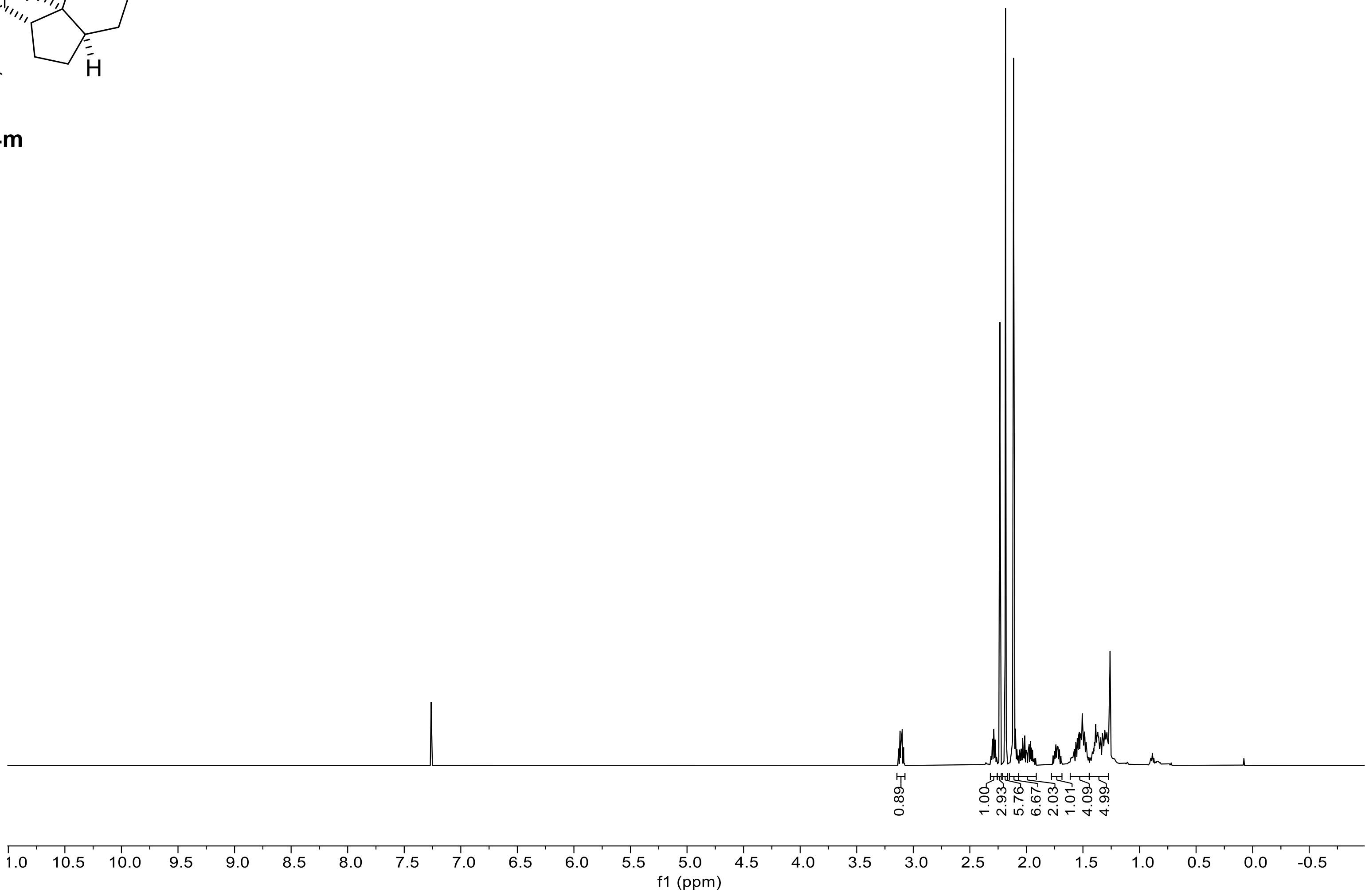

^{13}C NMR ($CDCl_3$, 126 MHz)

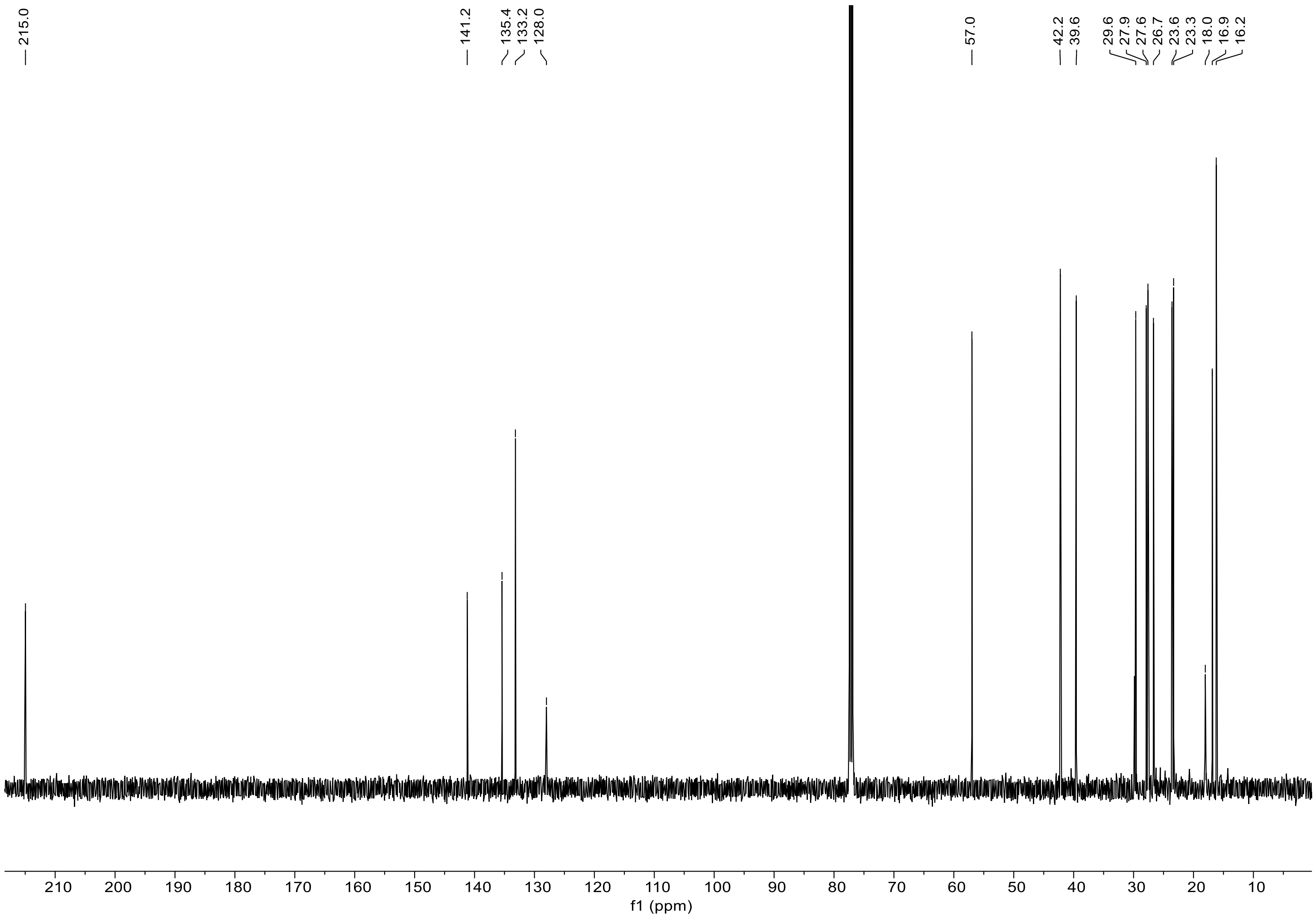

(2-Methyloctahydro-1H-inden-1-yl)(2,3,4,5,6-pentamethylphenyl)methanone (4n)
^{1}H NMR ($CDCl_3$, 400 MHz)

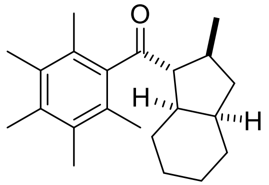

4n

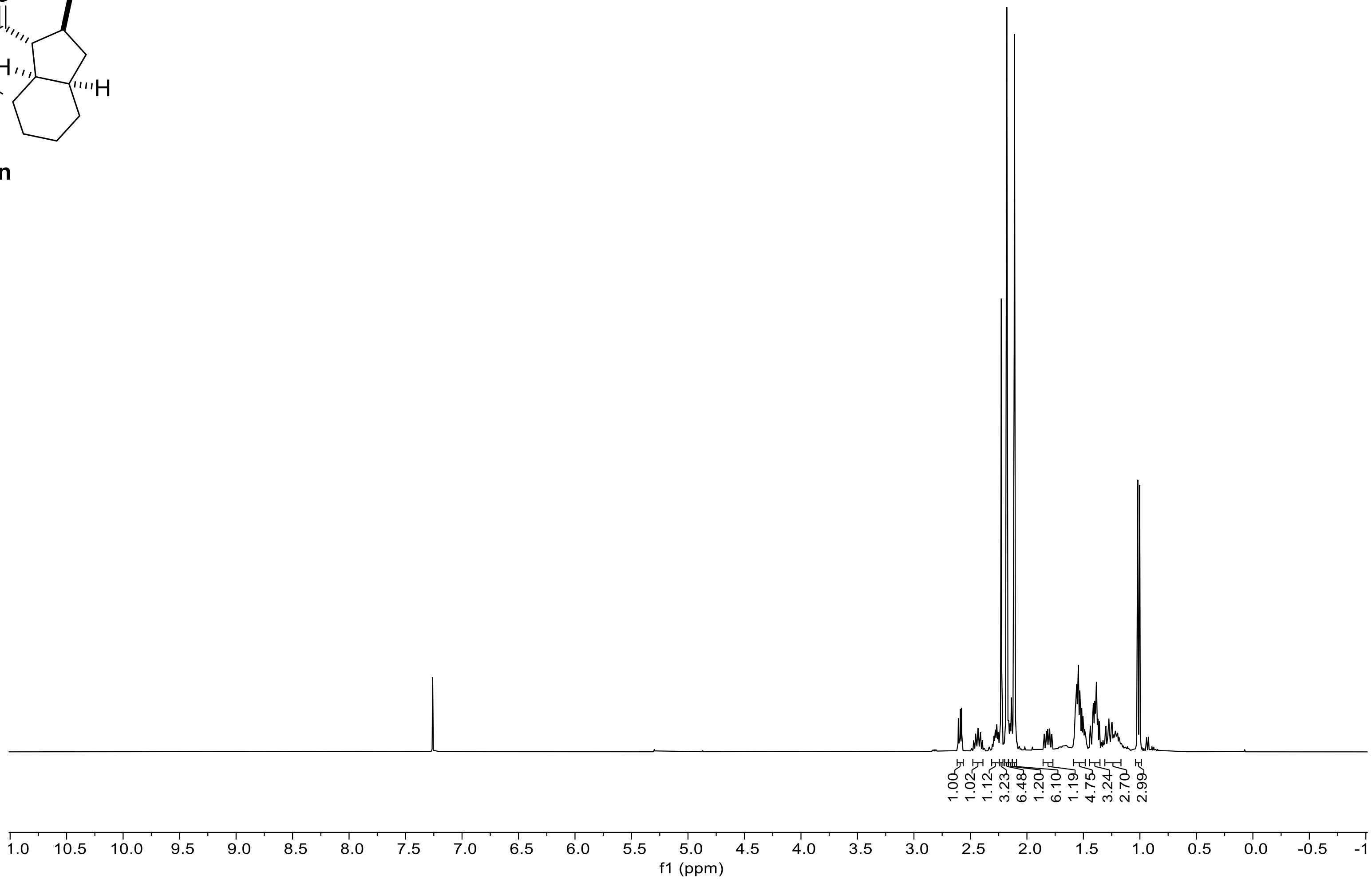

^{13}C NMR ($CDCl_3$, 101 MHz)

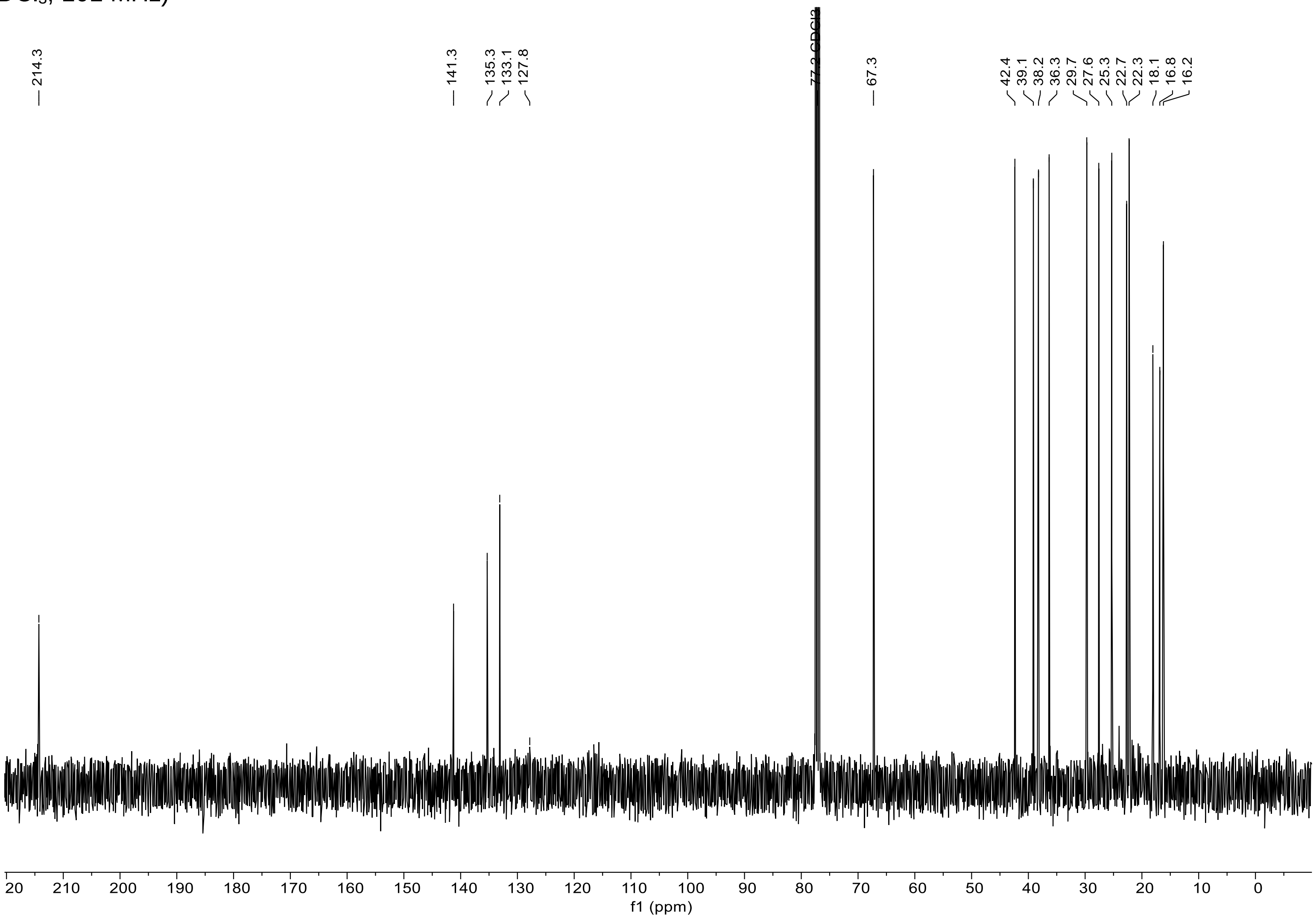

***trans*-*S*-Benzyl 2-ethylcyclopentane-1-carbothioate (5a)**
^{1}H NMR ($CDCl_3$, 500 MHz)

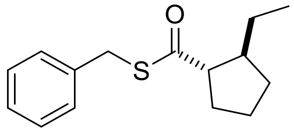

5a

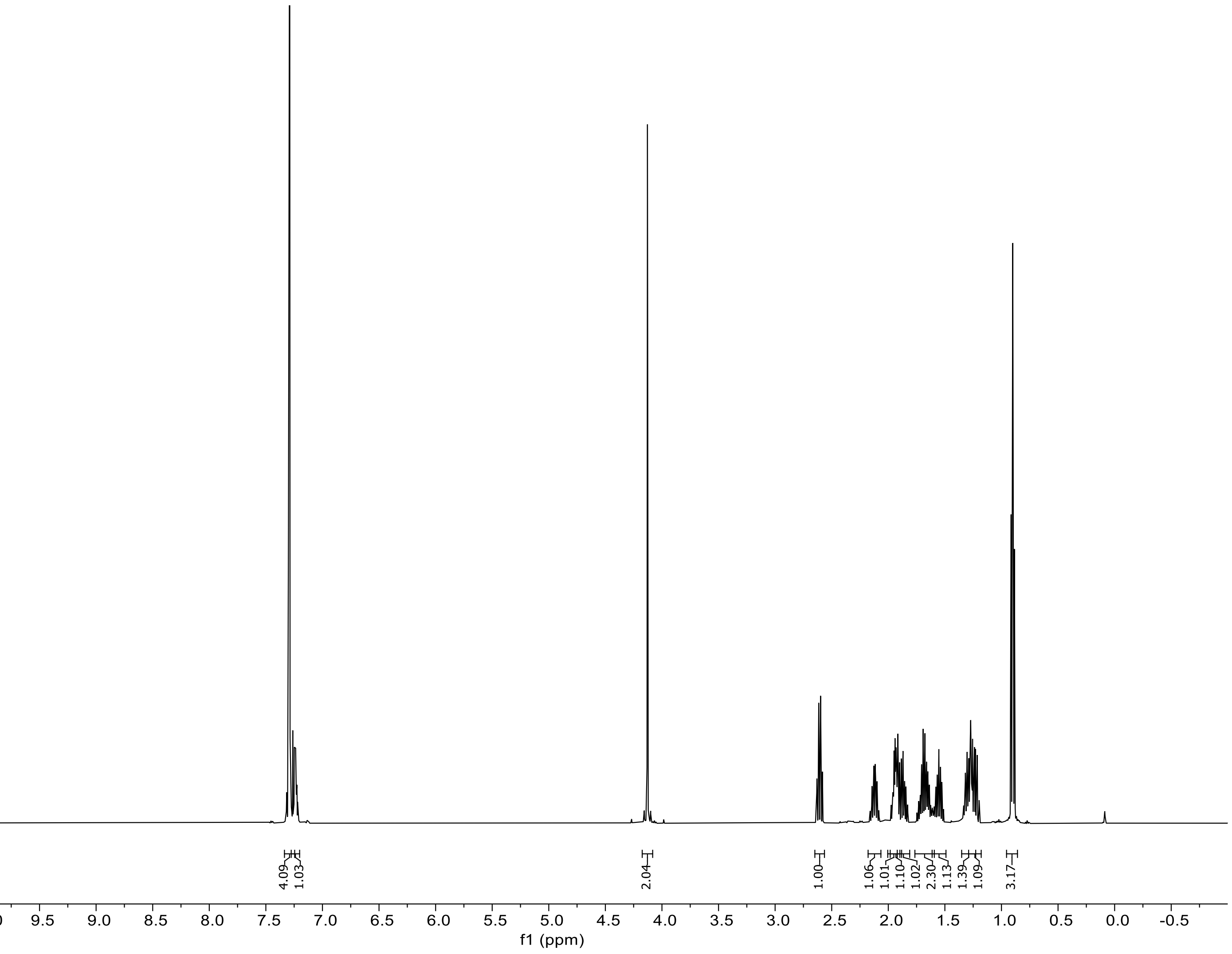

^{13}C NMR ($CDCl_3$, 126 MHz)

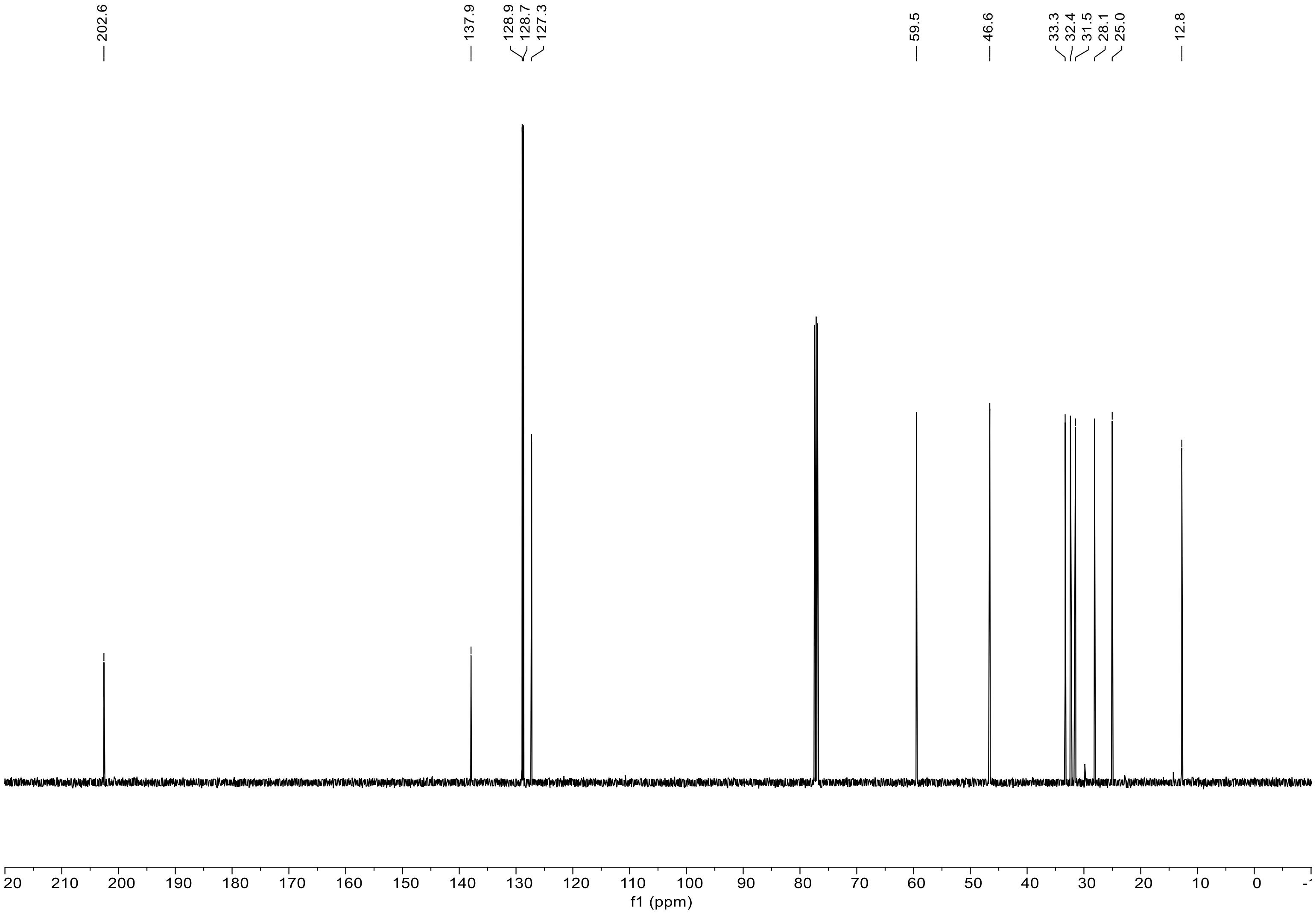

***trans*-2-Cyclobutylcyclopentane-1-carboxylic acid (5c)**
^{1}H NMR ($CDCl_3$, 500 MHz)

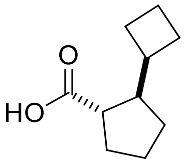

5c

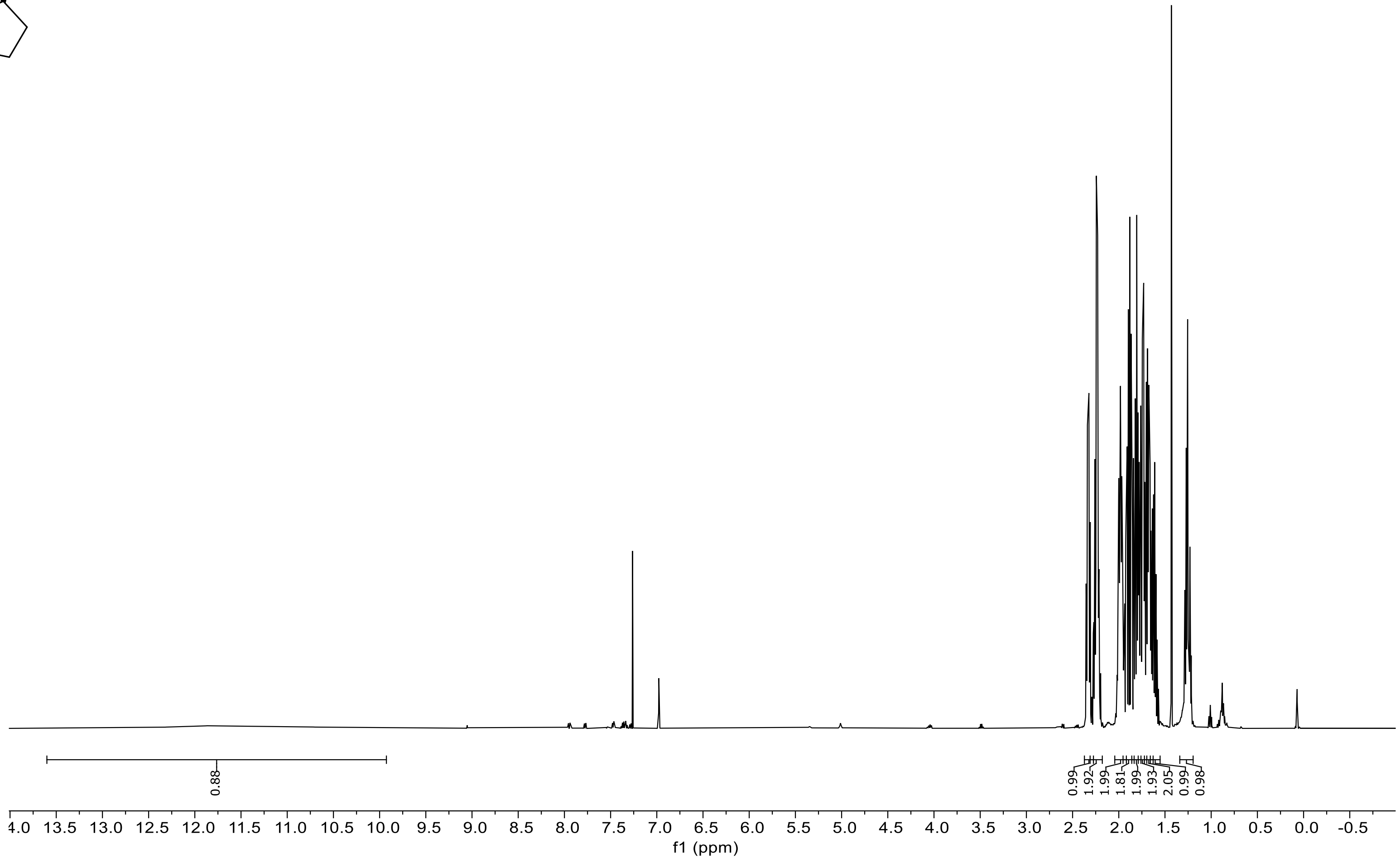

^{13}C NMR ($CDCl_3$, 126 MHz)

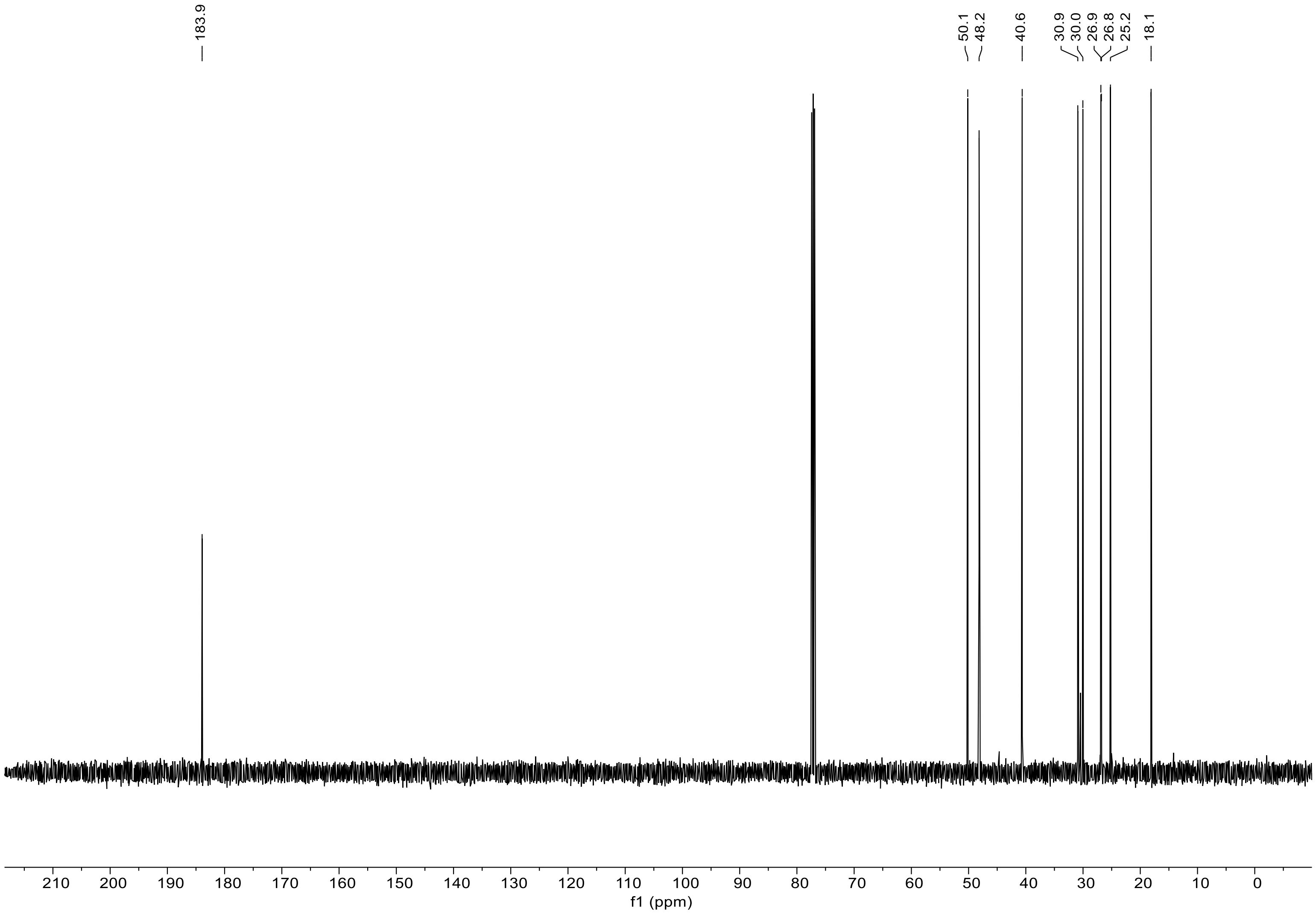

***trans*-2-Phenethylcyclopentane-1-carboxylic acid (5d)**
^{1}H NMR ($CDCl_3$, 500 MHz)

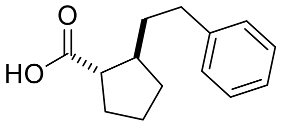

5d

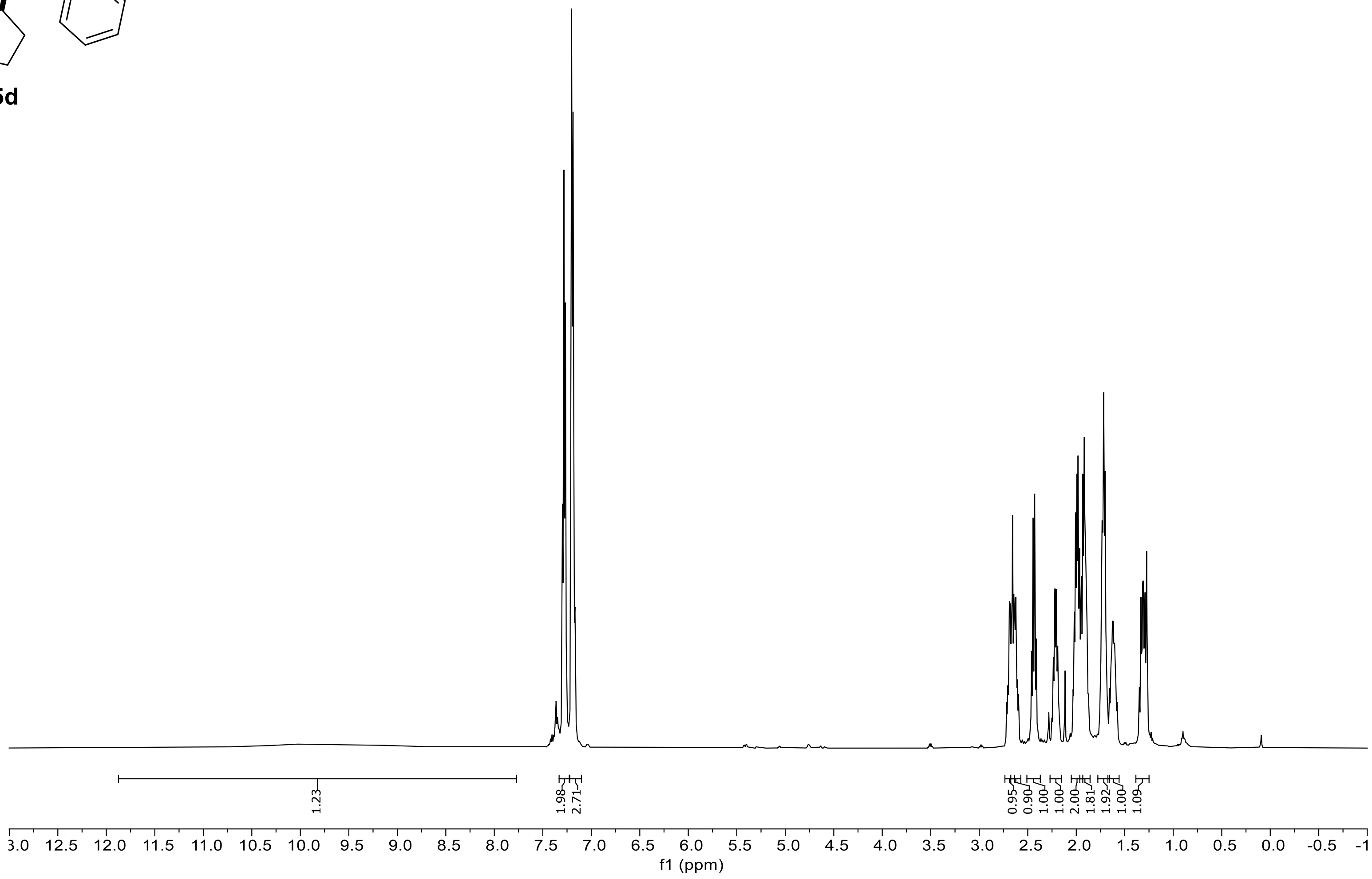

^{13}C NMR ($CDCl_3$, 126 MHz)

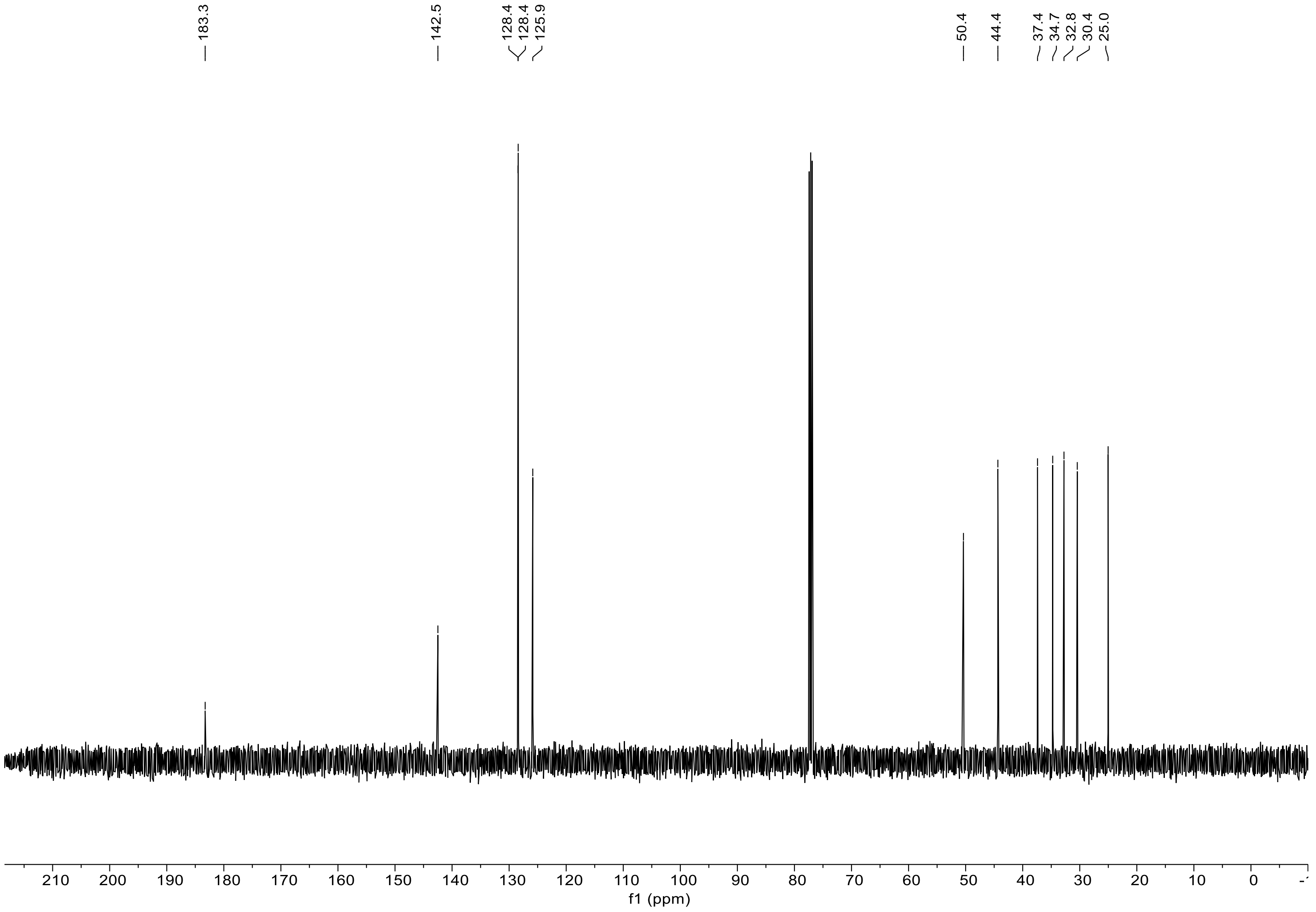

Octahydro-1*H*-cyclopenta[*c*]oxepin-1-one (5f)
^{1}H NMR ($CDCl_3$, 500 MHz)

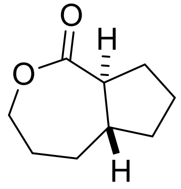

5f

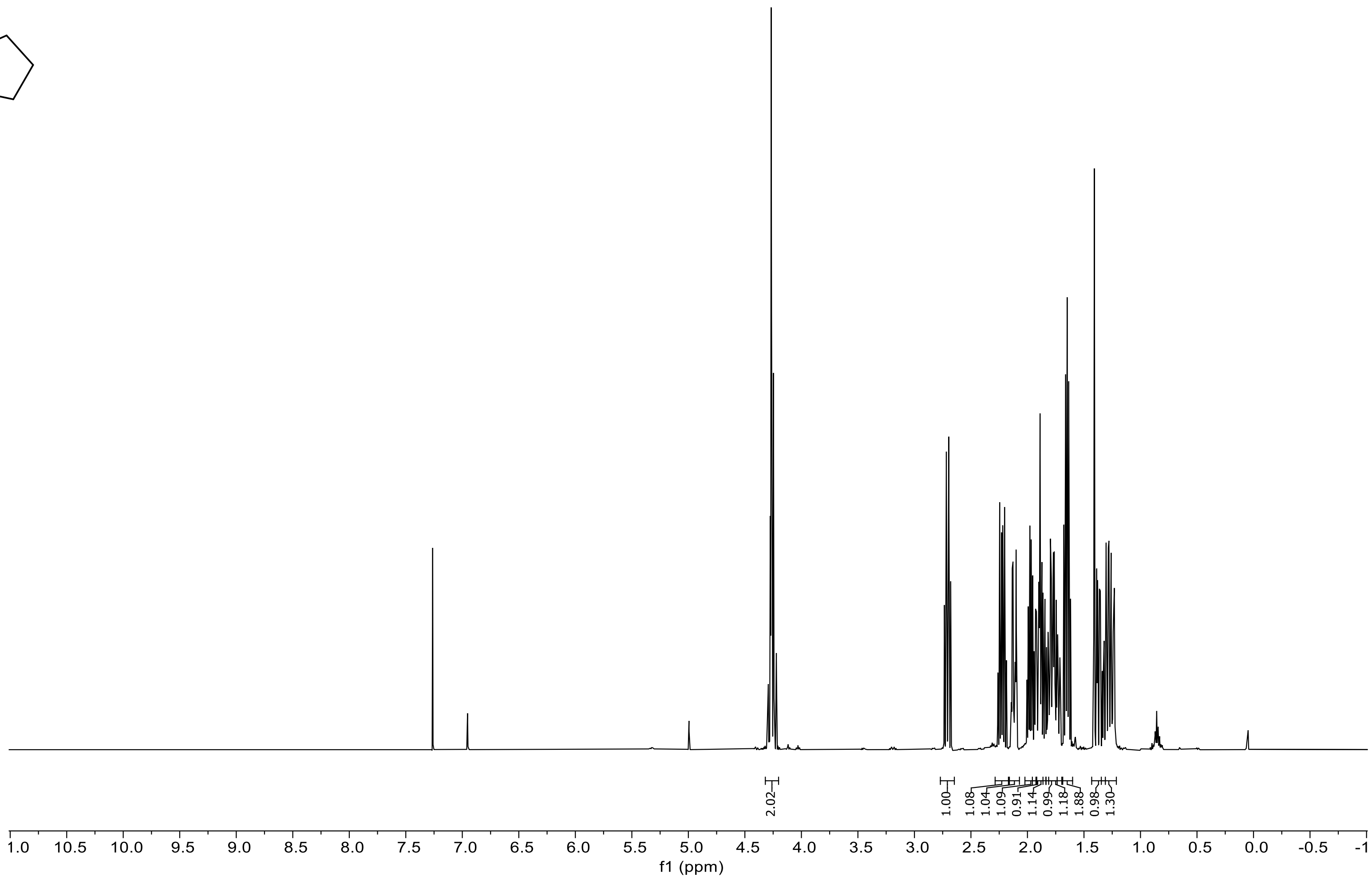

^{13}C NMR ($CDCl_3$, 126 MHz)

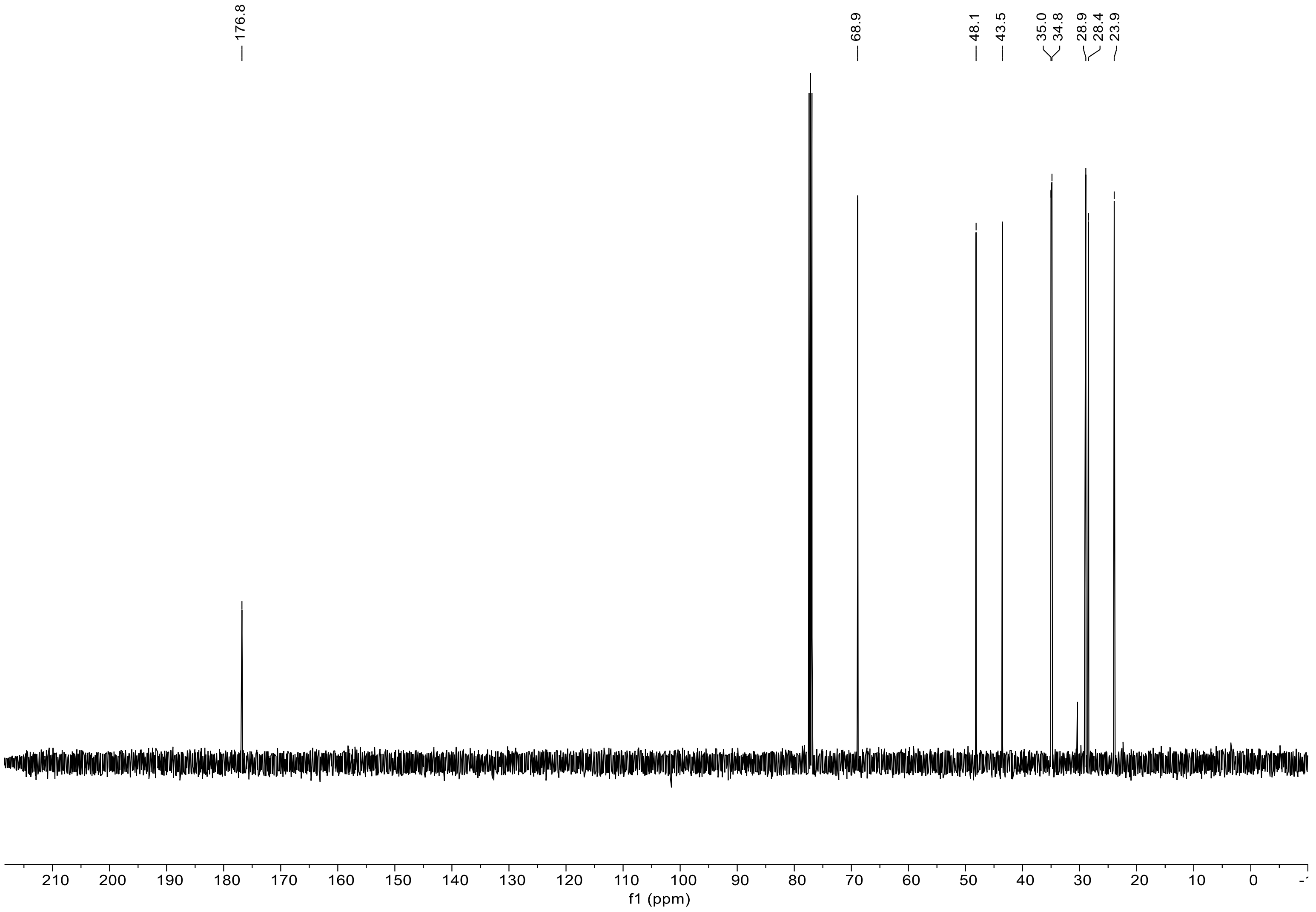

1-Benzylpiperidin-4-yl *rel*-(1*S*,6*S*,7*S*,8*S*)-7-methylbicyclo[4.3.0]nonane-8-carboxylate (5l)
^{1}H NMR ($CDCl_3$, 500 MHz)

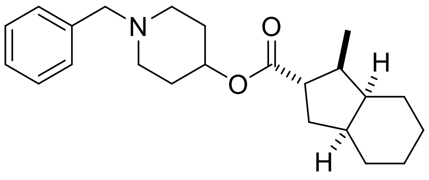

5l

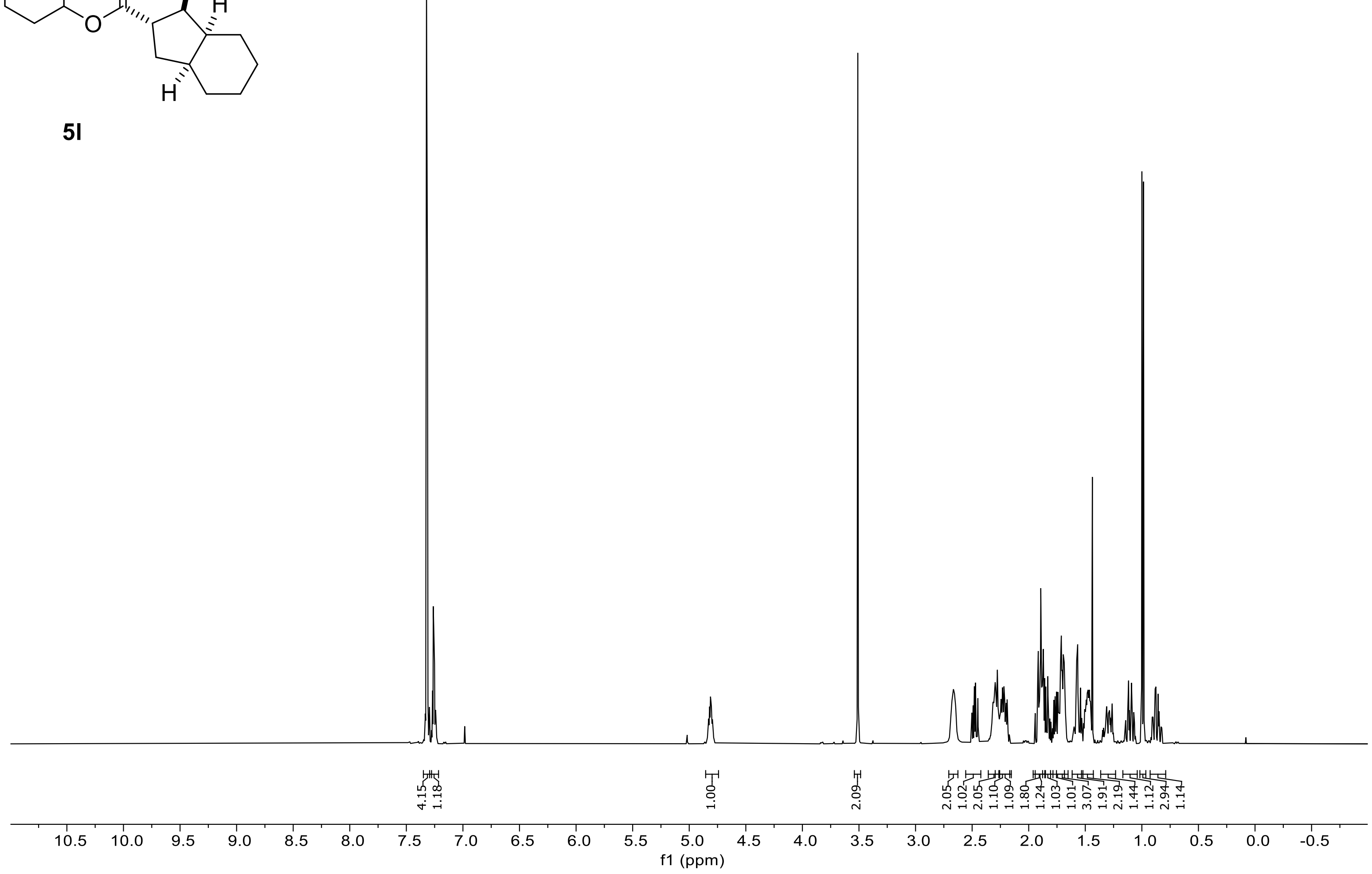

^{13}C NMR ($CDCl_3$, 126 MHz)

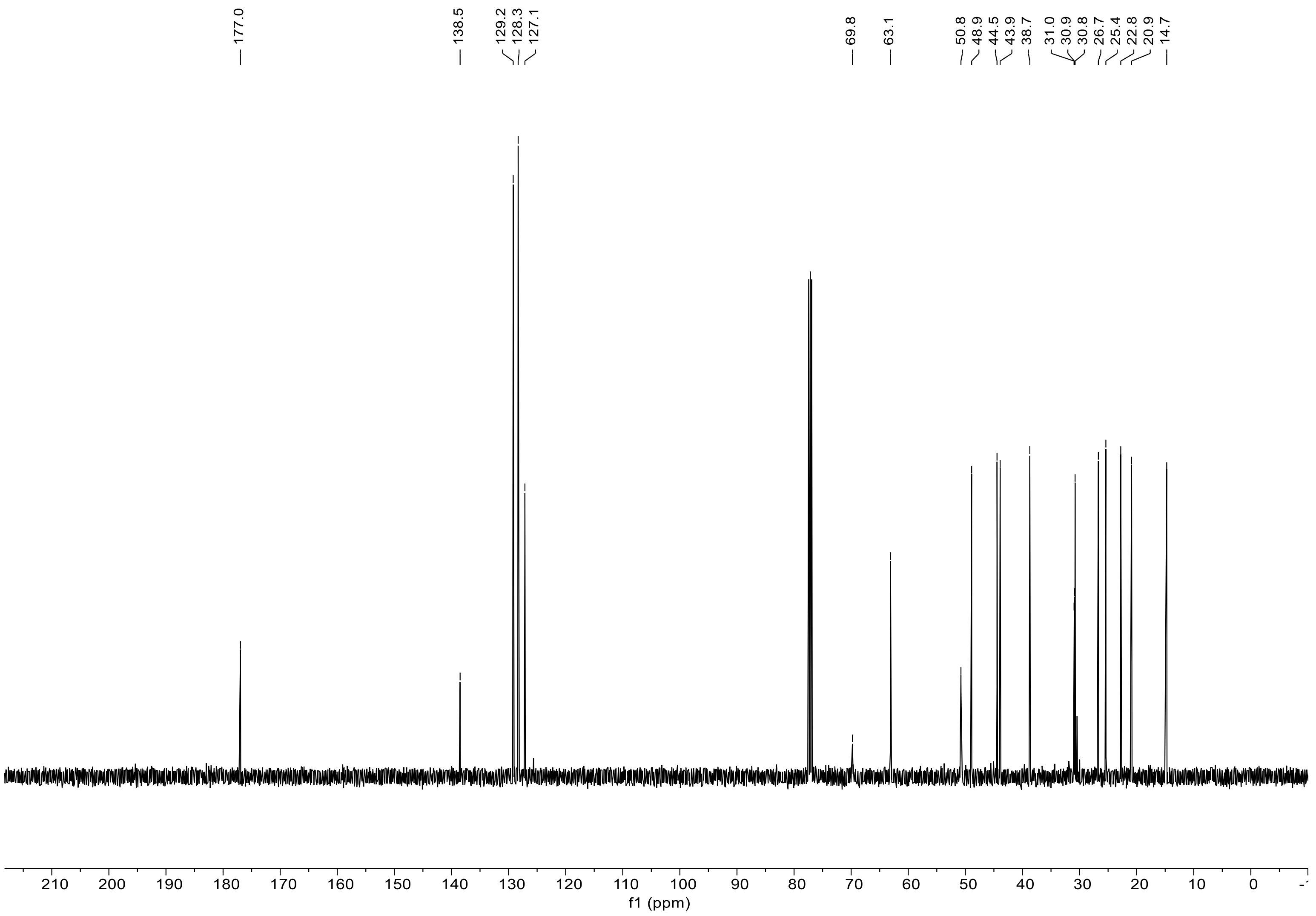

(4-Benzylpiperazin-1-yl)(octahydro-1H-inden-1-yl)methanone (5m)
^{1}H NMR ($CDCl_3$, 500 MHz)

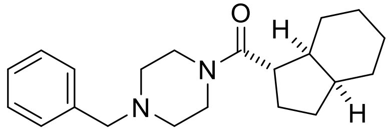

5m

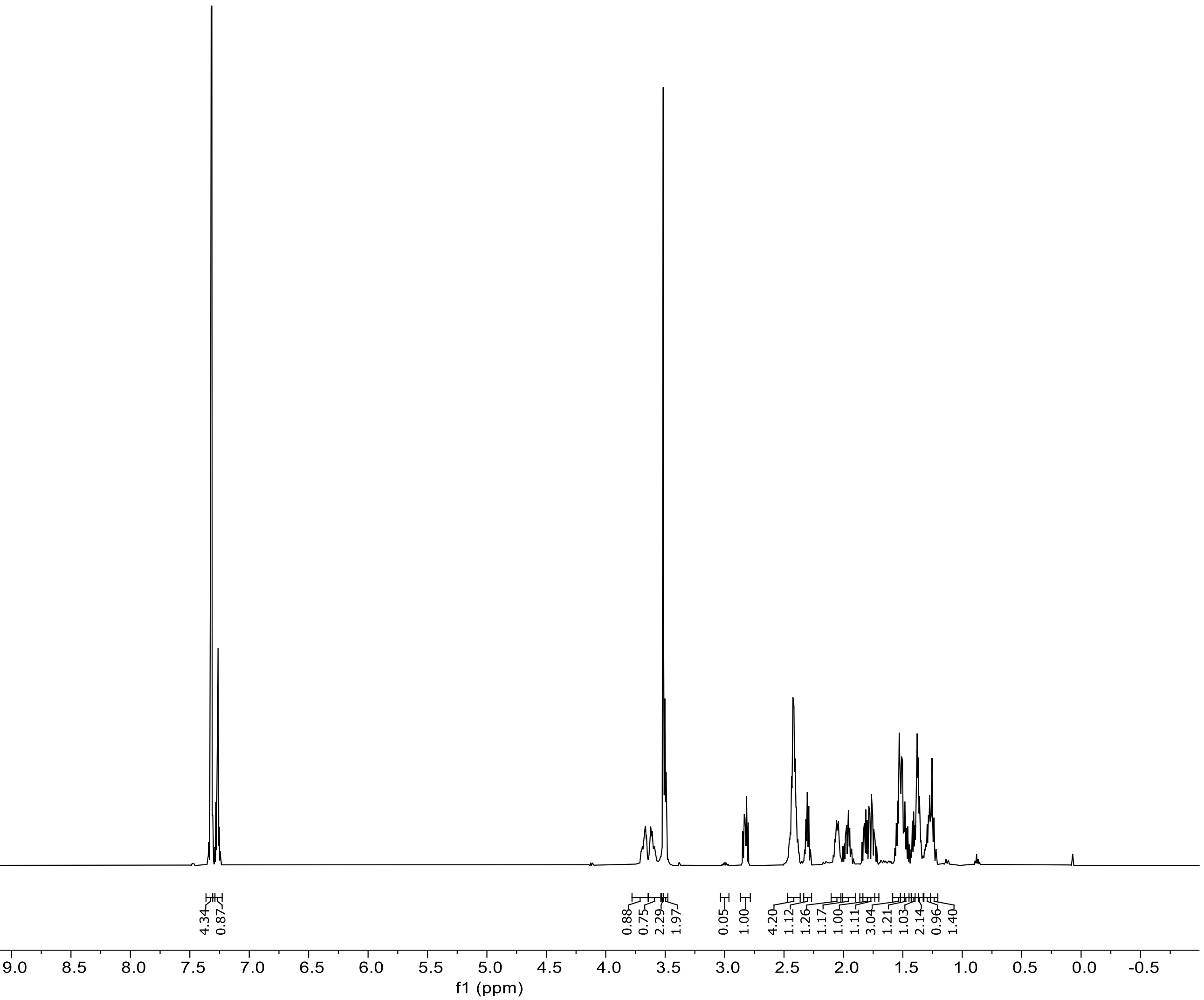

^{13}C NMR ($CDCl_3$, 126 MHz)

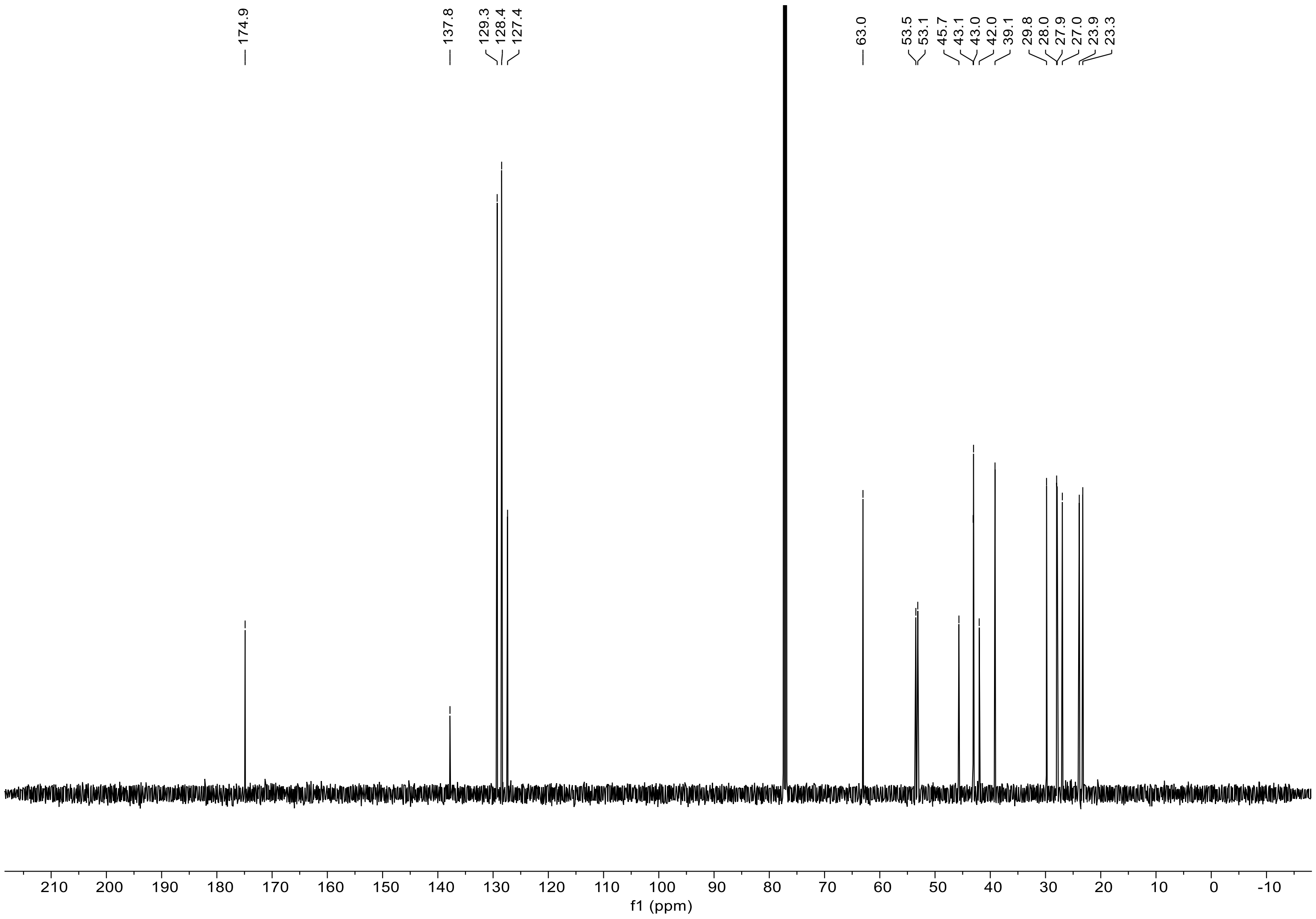

3-Cyclopropyl-1-(2,3,4,5,6-pentamethylphenyl)pentan-1-one (3a)
^{1}H NMR ($CDCl_3$, 400 MHz)

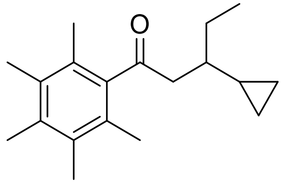

3a

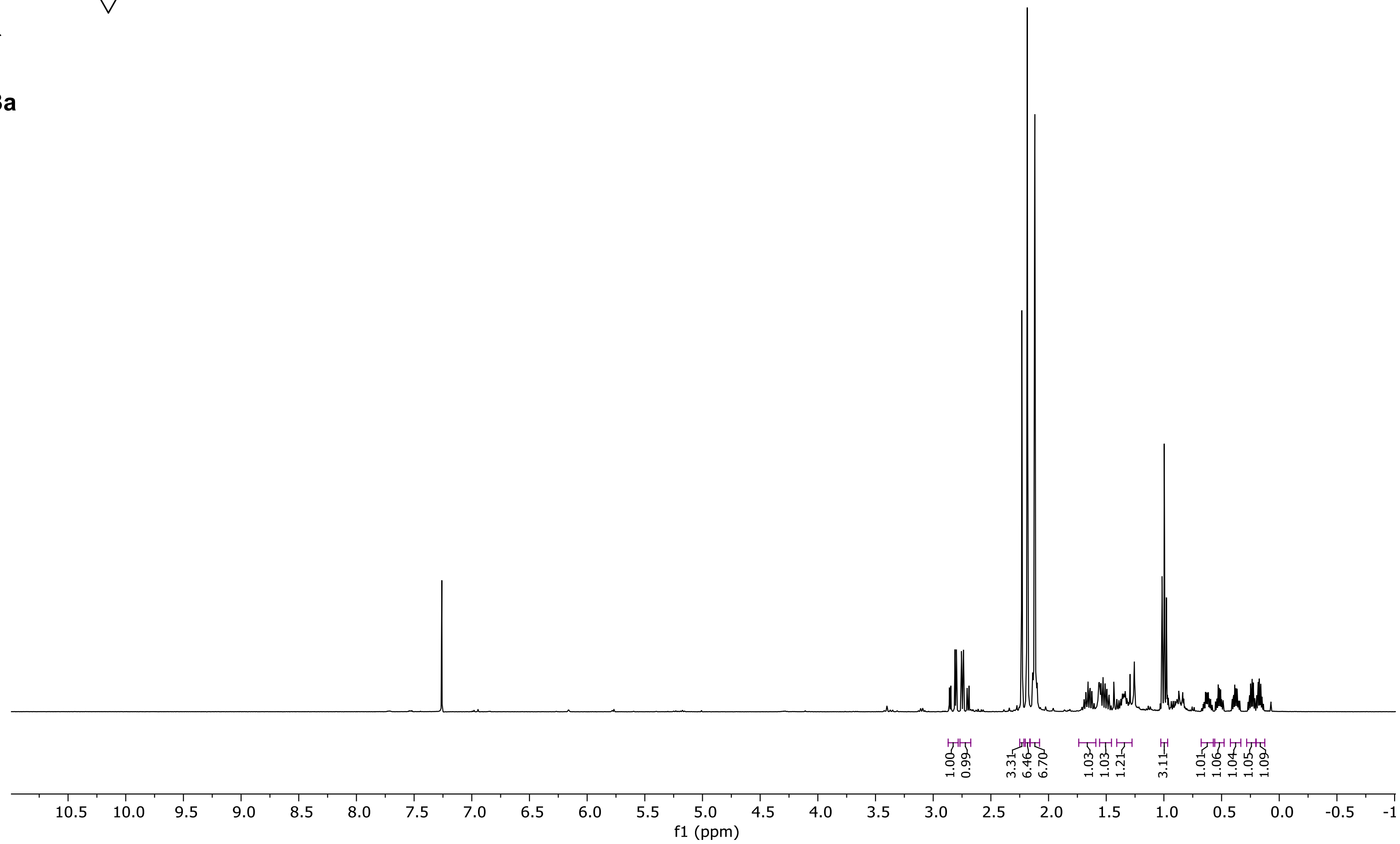

^{13}C NMR ($CDCl_3$, 101 MHz)

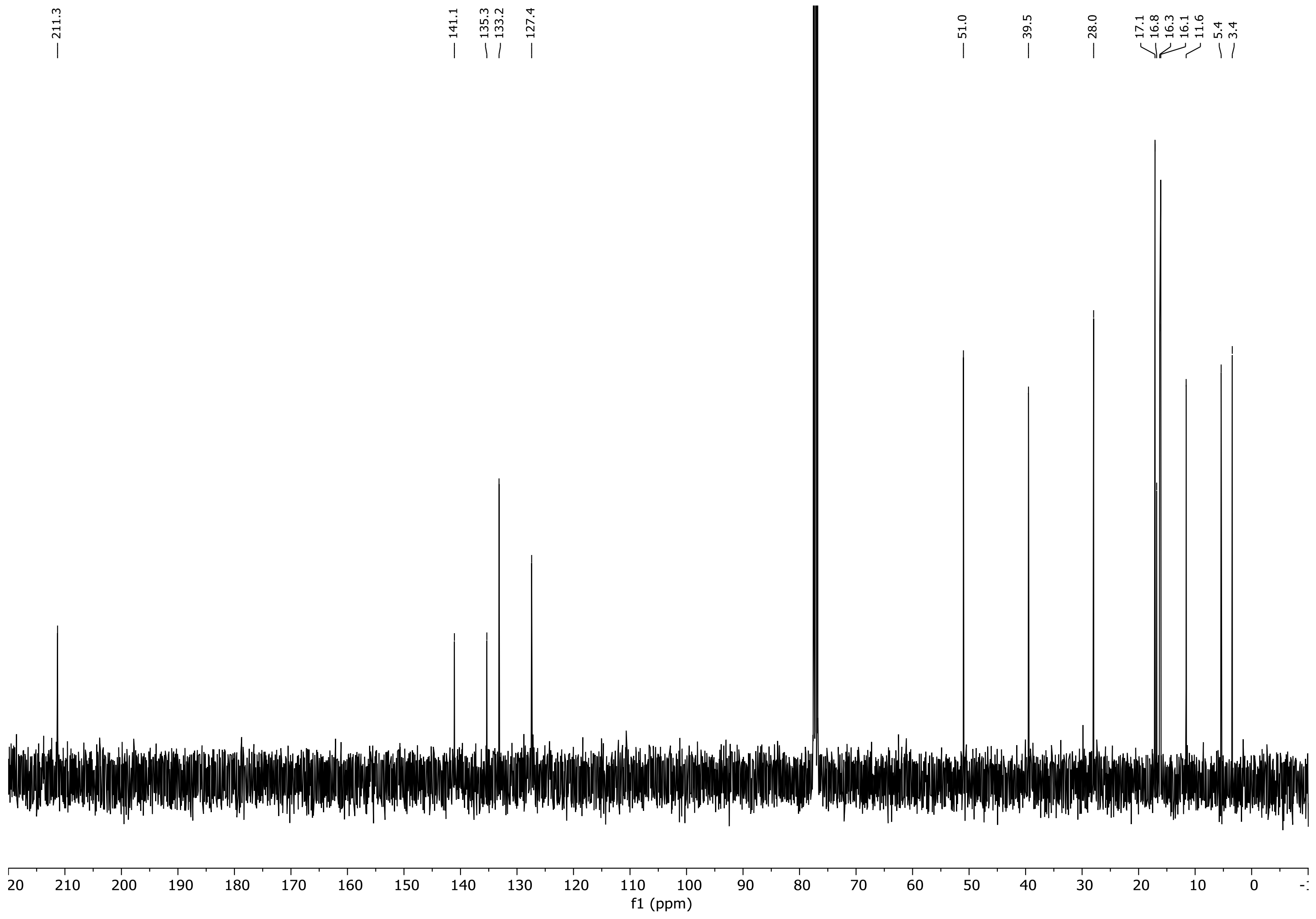

5 Single crystal X-ray diffraction for compounds 4a, 4l and 4n

Single Crystal Data for **4a**	$C_{19}H_{28}O$
	Mr =272.43
	150 K
	Monoclinic $P2_1$
	a = 8.7910(5) Å, b = 5.8022(2) Å, c = 16.0248(10) Å
	β = 93.603(5)°
	V = 815.76(7) Å^3
	Data/restraints/parameters – 3346/159/240
	Rint = 0.033
	Final R1 = 0.0995, wR2 = 0.2441 (I>2σ(I))

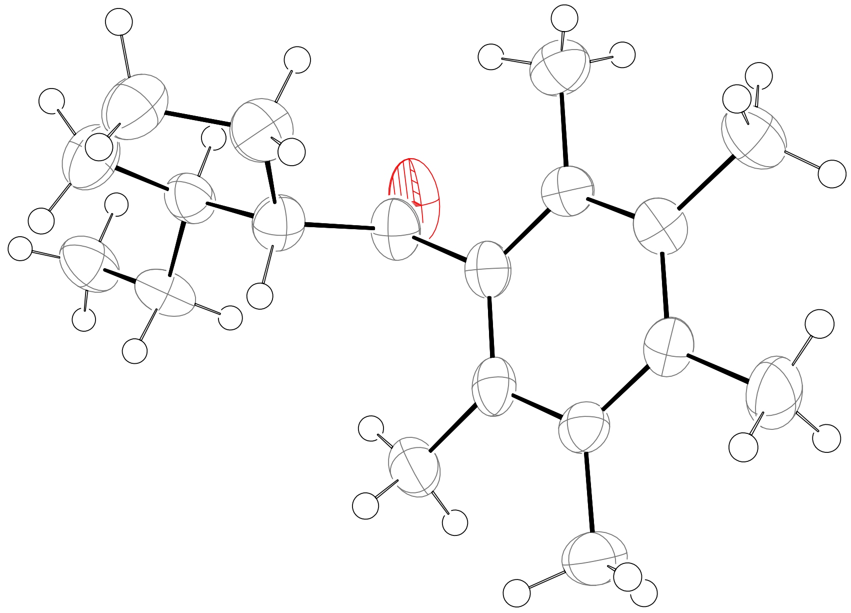

Figure S1. X-ray crystal structure (ellipsoid representation, probability level of 50%) for **4a**. Disorder are omitted for clarity.

Single Crystal Data for **4l**: $C_{22}H_{32}O$

Mr =312.50

100 K

Orthorhombic *P*$2_12_12_1$

a = 34.8779(8) Å, b = 8.8009(2) Å, c = 5.79370(10) Å

α = β = γ = 90º

V = 1778.42(7) Å^3

Data/restraints/parameters – 2820/0/210

Rint = 0.093

Final R1 = 0.0564, wR2 = 0.1298 (I>2σ(I))

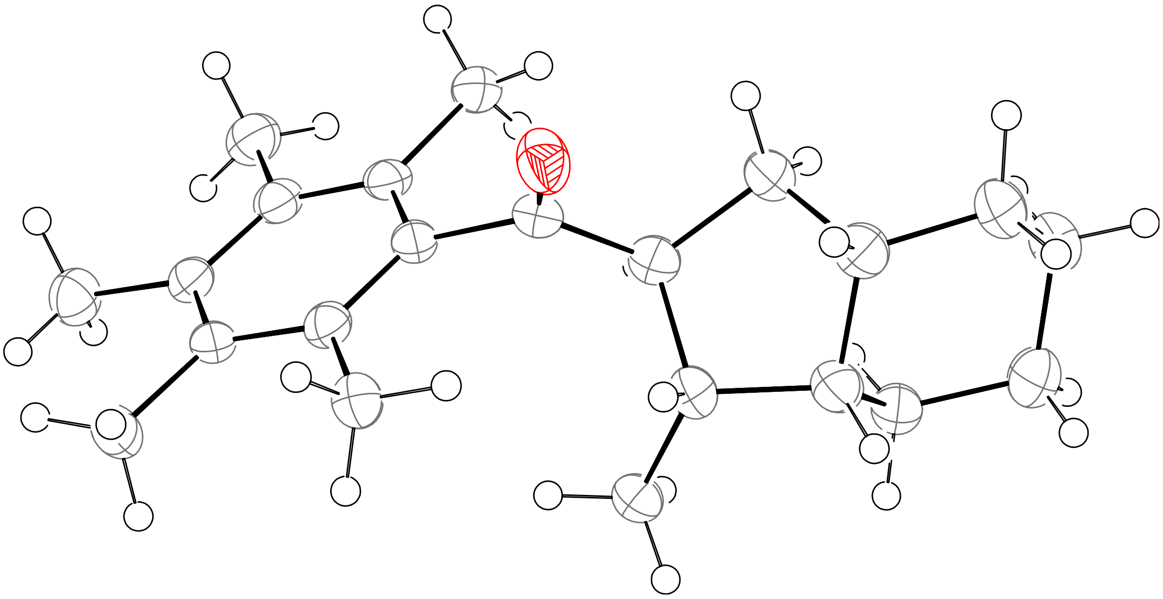

Figure S2. X-ray crystal structure (ellipsoid representation, probability level of 50%) for **4l**

Single Crystal Data for **4n**: $C_{22}H_{32}O$

Mr =393.49

150 K

Triclinic *P*-1

a = 6.441(4) Å, b = 10.5093(7) Å, c = 13.4425(10) Å

α = 88.956(6) °, β = 80.581(6) °, γ = 86.019(5)º

V = 895.90(11) Å^3

Data/restraints/parameters – 2543/260/288

Rint = 0.072

Final R1 = 0.1034, wR2 = 0.2333 (I>2σ(I))

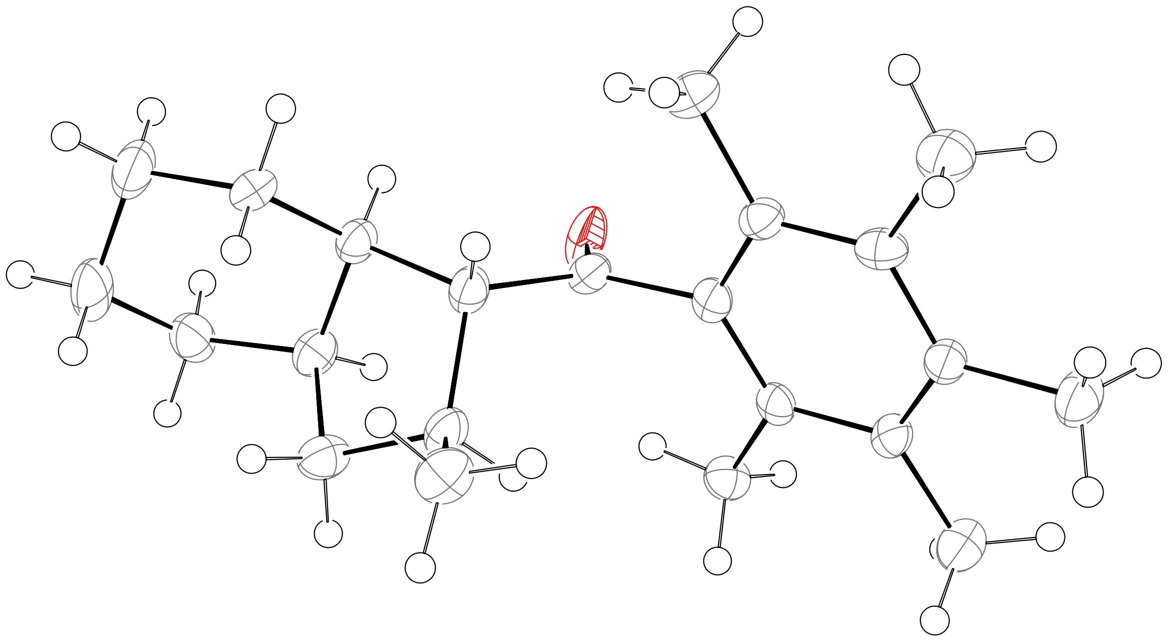

Figure S3. X-ray crystal structure (ellipsoid representation, probability level of 50%) **4n**.

6. References

[1] Y. Yamamoto, K. Maruyama, *J. Am. Chem. Soc.* **1985**, *107*, 6411–6413.

[2] J. R. Frost, C. B. Cheong, W. M. Akhtar, D. F. J. Caputo, N. G. Stevenson, T. J. Donohoe, *J. Am. Chem. Soc.* **2015**, *137*, 15664–15667.

[3] A. L. J. Beckwith, G. Moad, *J. Chem. Soc., Perkin Trans. 2* **1980**, 1473–1482.

[4] *Commissariat à l'Energie Atomique* **2008**, EP1997805 (A1), 88–59.

[5] R. J. Armstrong, W. M. Akhtar, T. A. Young, F. Duarte, T. J. Donohoe, *Angew. Chem. Int. Ed.,* **2019**, *58*, 12558–12562.

[6] W.C. Still, M. Kahn, A. Mitra, *J. Org. Chem.* **1978**, *43*, 2923–2925.

[7] R. K. Harris, E. D. Becker, S. M. Cabral de Menezes, P. Granger, R. E. Hoffman, K. W. Zilm, *Pure Appl. Chem.* **2008**, *80*, 59–84.

[8] J. Cosier, A. M. Glazer, *J. Appl. Cryst*., **1986**, *19*, 105–107.

[9] L. Palatinus, G. Chapuis, *J. Appl. Cryst.*, **2007**, *40*, 786–790.

[10] (a) P. Parois, R. I. Cooper, A. L. Thompson, *Chem. Cent. J.*, **2015**, 9:30. (b) R. I. Cooper, A. L. Thompson, D. J. Watkin, *J. Appl. Cryst.* **2010**, *43*, 1100–1107.

[11] P. W. Betteridge, J. R. Carruthers, R. I. Cooper, K. Prout, D. J. Watkin, *J. Appl. Cryst.* **2003**, *36*, 1487.

[12] C. Ferreri, M. Ambrosone, C. Chatgilialoglu *Synth. Commun.* **1995**, *25*, 3351–3356.

[13] H. Lebel, A. Charette, *J. Org. Chem.* **1995**, *60*, 2966–2967.

[14] *Oxazolidinone as Modulators of mGluR5* **2015,** WO2015054103 (A1).

[15] L. Alcaraz, J. Harnett, C. Mioskowski, J. P. Martel, T. L. Gall, D.-S. Shin, J. R. Falck *Tetrahedron Lett.* **1994**, *35*, 5449–5452.

[16] L. A lcaraz, A. Cridl, E. Kinchin, *Org. Lett.* **2001**, *3*, 4051–4053.

[17] J. Furukawa, N. Kawabata, J. Nishimura, *Tetrahedron* **1968**, *24*, 53–58.

[18] S. Yamada, H. Suginome, *Tetrahedron* **1987**, *43*, 3371–3386.
